# Fair and Square: Design, Synthesis and Biological Evaluations of Squaric Acid Derivatives as Novel HDAC8 Inhibitors

**DOI:** 10.1002/cmdc.70270

**Published:** 2026-04-25

**Authors:** Nathan Long, Franz‐Josef Meyer‐Almes, Aleksandra Kopranovic, Stephen P. Wren

**Affiliations:** ^1^ School of Life Sciences, Pharmacy and Chemistry Faculty of Health, Science, Social Care and Education Kingston University London Kingston UK; ^2^ Department of Chemical Engineering and Biotechnology University of Applied Sciences Darmstadt Darmstadt Germany

**Keywords:** bioisostere, HDAC, squaramide, squaric acid

## Abstract

Histone deacetylase 8 (HDAC8) is a clinically validated target in neuroblastoma, where isoform selective inhibition offers a strategy to suppress tumour growth while limiting off‐target toxicity. Hydroxamic acids remain the dominant zinc‐binding group (ZBG) in HDAC inhibitors but are also associated with metabolic instability, suboptimal pharmacokinetics and nonspecific metal chelation. In this study, we report the biological evaluation of a focussed library of 51 squaric acid derivatives as alternative, non‐hydroxamic HDAC inhibitors. While a subset of these compounds has been described previously in a synthetic context, their HDAC inhibitory activity and selectivity have not been reported to date. The compounds organised into four structural subfamilies were screened against HDAC8 with moderate isoform selectivity. Structure activity relationships, supported by molecular docking and in silico techniques, highlight the influence of substitution pattern, electronic properties and molecular rigidity on HDAC8 inhibition. Collectively, these findings establish squaric acid derivatives as a viable non‐hydroxamic acid scaffold for the development of selective HDAC inhibitors.

## Graphical abstract

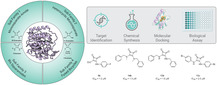

## 1 Introduction

The histone deacetylases (HDACs) are a family of enzymes first discovered in the 1960s, recognised for their fundamental role in regulating gene expression via post‐translational modification of chromatin [1]. By removing acetyl groups from lysine residues on histone proteins, the chromatin structure is tightened and transcription is suppressed. Beyond histones, HDACs also act on a variety of non‐histone proteins, influencing processes such as cell cycle progression, apoptosis and metabolism [2]. In humans, 18 lysine deactylases have been identified and classified based on their structural features and cofactor requirements. HDACs are categorised into four classes: Class I (HDAC's 1, 2, 3 and 8), Class II (HDAC's 4, 5, 6, 7, 9 and 10), Class III (NAD^+^‐dependent sirtuins) and Class IV (HDAC11, which shares properties with both Classes I and II) [3]. HDACs belonging to Classes I, II and IV, are recognised to be zinc‐dependent metalloenzymes [3, 4].

**FIGURE 1:**
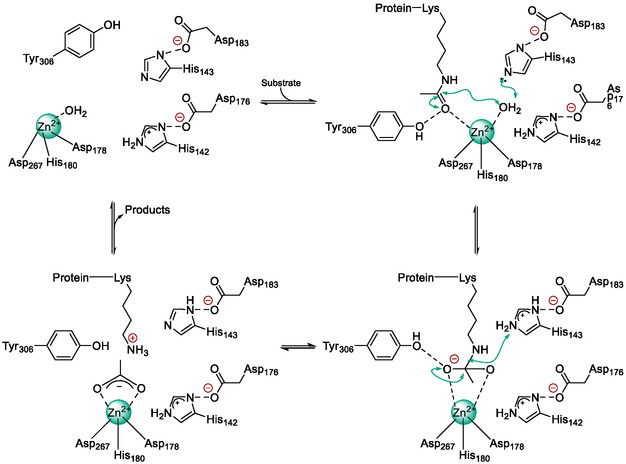
Simplified illustration of the deacetylation mechanism carried out by HDACs. The numbering of amino acid residues according to UniProt Q9By41‐1 (human HDAC8) [5].

HDAC8 catalyses lysine deacetylation through a Zn^2+^‐dependent hydrolytic mechanism in which the acetyl‐lysine carbonyl is activated by direct coordination to Zn^2+^ and hydrogen bonding with the catalytic tyrosine Y306. In the Michaelis complex, a Zn^2+^‐bound water molecule is positioned and activated for nucleophilic attack by the tandem histidine dyad: H143 functions as a general base to deprotonate the water and later as a general acid to protonate the leaving amine, while H142 provides electrostatic stabilisation and optimal water orientation. Nucleophilic attack generates a tetrahedral intermediate stabilised by Zn^2+^ coordination and hydrogen bonds to Y306, H142 and H143, as evidenced by crystal structures with substrate analogues and transition‐state mimics. Collapse of this intermediate yields deacetylated lysine and acetate, with product release potentially occurring through a backdoor tunnel in the enzyme; see Figure 1.

Belonging to the Class I group, HDAC8 is distributed across the cytoplasm, nucleus and mitochondria, where it modulates gene expression and cytoskeletal dynamics [6, 7]. HDAC8 is particularly abundant in smooth muscle, brain tissue and tumour cells, making it a promising target in cancer therapy [8]. It also plays a critical role in genomic stability by deacetylating the structural maintenance of chromosomes 3 (SMC3) in the cohesion complex, an essential component for chromosome segregation during mitosis and the early G1 phase [9].

HDAC dysregulation is implicated in a vast variety of pathological states including interstitial fibrosis, inflammation, immunomodulation, diabetes mellitus and cancer [10]. In oncology, HDAC8 is frequently overexpressed, notably in childhood neuroblastoma, as well as in T‐cell lymphomas, breast and colorectal cancers [11]. Selective inhibition of HDAC8 has been shown to directly suppress tumour growth, induce apoptosis and enhance sensitivity to conventional chemotherapy efficacy [12]. Additionally, mutations in HDAC8 are linked to Cornelia de Lange syndrome (CdLS), a rare neurodevelopmental disorder marked by intellectual disability and physical malformations [9].

**FIGURE 2:**
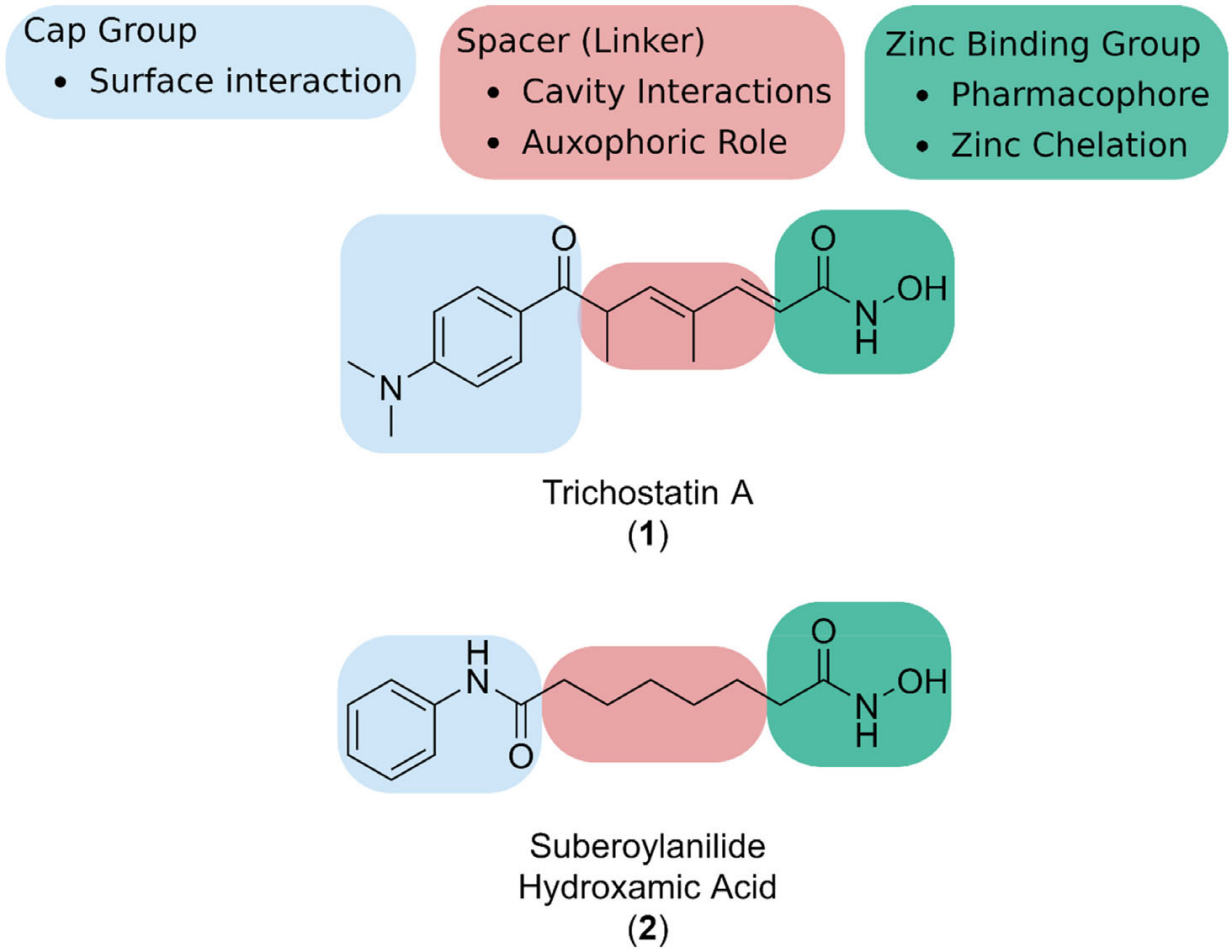
The structure of Trichostatin (TSA) and Suberoylanilide (SAHA, Vorinostat) highlighting the three parts of the HDAC inhibitor pharmacophore.

Most histone deacetylase inhibitors (HDACIs) follow a modular design based on the endogenous aliphatic acetyl‐lysine substrate. Structural motifs require the presence of a suitable Zn^2+^ binding group which can bond with the catalytic ion at the narrow bottom of the active site and a cap group which is linked by a straight chain alkyl, vinyl or aryl framework (Figure 2 single colum) [13, 14, 15].

**FIGURE 3:**
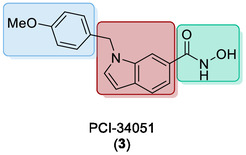
Chemical structure of PCI‐34051, a HDACI synthesised in 2008 showing selectivity for HDAC8.

To improve selectivity and reduce toxicity associated with broad‐spectrum HDAC inhibition, recent efforts have focussed on developing isoenzyme‐specific HDACIs. PCI‐34051 (**3**) (Figure 3) is a potent and selective HDAC8 inhibitor with demonstrated efficacy against T‐cell lymphoma in preclinical models [16, 17].

A notable structural feature of HDAC8 is pronounced flexibility of regions surrounding the active site, which enables accommodation of a chemically diverse range of inhibitors and contributes to its distinct substrate and inhibitor recognition profile [18]. Isoform selectivity among zinc‐dependent HDACs is commonly achieved by exploiting structural differences in secondary binding pockets adjacent to the conserved catalytic zinc site, including the acetate release channel and flexible subpockets that differ in size, shape and conformational adaptability between isoenzymes. Such features have been shown to play a key role in selective ligand recognition, particularly in HDAC8 and Class IIa HDACs [19].

In HDAC8, the acetate release channel leads to an internal cavity of approximately 14 Å, which is accessible from the catalytic site and has been proposed to facilitate product egress during catalysis. Importantly, this cavity can be directly exploited for isoenzyme selective inhibition, with HDAC8 selective ligands binding within this region and achieving selectivity through favourable π‐stacking interactions with Trp141 [20]. Trp141 is uniquely aromatic in HDAC8 (and conserved only as Phe141 in HDAC11), whereas this position is occupied by non‐aromatic residues such as Leu or Gly in most other HDAC isoenzymes [20].

By contrast, although HDAC1 also features a hydrophobic ∼11 Å channel terminating in an internal cavity, substitutions within this region alter their physicochemical environment and reduce its ability to support analogous aromatic stacking interactions [13, 21, 22]. These structural differences are believed to play a crucial role in distinguishing HDAC8 selective inhibitors from compounds that bind more broadly across Class 1 HDACs.

The crystal structure of PCI‐34051 bound to HDAC8 (PDB: 6HQY) reveals a characteristic bidentate interaction between oxygen atoms of the hydroxamic acid moiety and the catalytic Zn^2+^ cation, consistent with its potent inhibitory profile. Notably, this structure corresponds to HDAC8 from *Schistosoma mansoni* (smHDAC8), which has been widely used as a structural surrogate due to its high degree of homology with the human enzyme [23]. In the same study, PCI‐34051 was also co‐crystallised with a humanised variant of *sm*HDAC8 (PDB: 6HSF), exhibiting a comparable binding pose and coordination geometry. Both structures reinforce the critical role of bidentate Zn^2+^ coordination in inhibitor binding and provide a valuable reference model for the development of new HDAC8 selective inhibitors [23].

While hydroxamic acids remain the dominant zinc‐binding warhead in HDAC inhibitors, they suffer from several well documented drawbacks, including metabolic instability (leading to toxicity), poor pharmacokinetics and binding to other biologically relevant cations such as Fe^2+^ [24, 25, 26]. In addition, hydroxamate‐based HDAC inhibitors have been shown to inhibit other metalloenzymes as off‐targets, contributing to unwanted biological effects limiting therapeutic selectivity [27]. These limitations have prompted considerable interest in the development of alternative zinc‐binding groups (ZBGs), including benzamides [28], ethanolamines [29] and hydrated aryl ketones [28], and more recently alkyl hydrazides, which have emerged as effective warheads in several selective HDAC8 inhibitor series [30], to name but a few [31].

**FIGURE 4:**
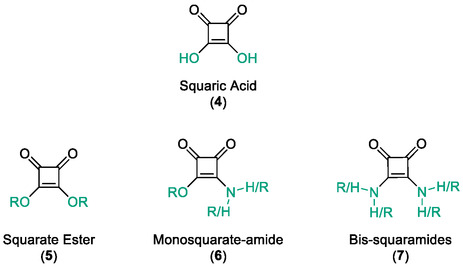
Squaric acid and functionalised squaric acid derivatives.

Inspired by the work published by Hanessian et al. in 2006, we sought to explore squaric acid derivatives as novel non‐hydroxamic acid HDACIs [32]. Squaric acid (**4**), first synthesised by Cohen in 1959, is a bench stable, planar diprotic acid featuring 2π pseudo‐aromaticity. Its unique geometry and dual hydrogen bonding capability make it a promising candidate for metal coordination [33, 34]. Squaric acid and its derivatives such as squarate esters (**5**) [35], monosquarate amides (**6**) or bis‐squaramides (**7**) are easily accessible via mild transformations (Figure 4) [35, 36, 37].

Squaric acid and its fully deprotonated squarate dianion are well‐established O‐donor ligands in coordination chemistry and are known to form stable complexes with a variety of metal cations including alkali, alkaline earth and transition metals [38, 39, 40]. Early studies demonstrated coordinated of squarate to metal ions such as Zn^2+^, Cu^2+^ and Fe^2+^ through carbonyl oxygen atoms, a property that has occasionally been exploited in organometallic synthesis and Lewis acid‐mediated transformations [40, 41]. Importantly, the metal binding behaviour of squaric acid derivatives differs substantially from that of the parent squarate anion. Conversion of squarate esters and, in particular, squaramides reduces formal charges and alters the electronic environment of the carbonyl groups, resulting in metal coordination that is highly context dependent rather than indiscriminate. In biological systems, squaramide motifs therefore act as directed zinc‐binding groups when appropriately positioned within enzyme active sites such as with the case of SNM1A [41, 42, 43].

With respect to metabolic stability, it should be noted that dedicated experimental studies evaluating the in vivo or microsomal stability of squaric acid‐derived motifs remain limited. To date, however, there is no evidence in the literature to suggest that squaric acid derivatives or squaramides are inherently unstable under physiological conditions. Squaramide‐containing compounds have been successfully employed in multiple medicinal chemistry programmes without reports of rapid degradation or instability compromising biological evaluation [41].

Beyond their emerging role in HDAC inhibition, squaric acid and squaramide motifs are increasingly recognised as privileged scaffolds in medicinal chemistry, displaying biological activity across a diverse range of molecular targets. A comprehensive review published in 2021 by Chasák and colleagues highlights the application of squaric acid derivatives for medicinal chemistry [41]. Representative examples include squaramide‐based inhibitors of the DNA repair metalloenzymes SNM1A, where the squaramide motif coordinates the catalytic metal ion and disrupts DNA repair pathways [42]. Squaric acid derivatives have also been reported as ATP synthase inhibitors and CXCR2 antagonists in inflammatory disease models, as summarised in the above review [41, 44, 45].

Squaric acid and their related derivatives (such as mono‐ and bis‐squaramides) have attracted interest in medicinal chemistry as scaffold motifs capable of mimicking the electronic and hydrogen bonding features of carboxylic acid and hydroxamic acids. While the parent molecule squaric acid itself has been proposed as a potential carboxylic acid bioisostere in works by Ballatore and Lassalas, our study focuses on its functionalised amide derivatives [46, 47].

## 2 Results

### 2.1 Chemical Synthesis

A focussed library of 51 squaric acid derivatives was assembled and evaluated for inhibition of HDAC4 and HDAC8. To enable a systematic structure activity relationship (SAR) analysis, compounds were organised into four distinct subfamilies based on their structural features. Subfamily 1 comprises monosquarate amides derived from the nucleophilic addition elimination reaction of diethyl squarate (**8**) and corresponding functionalised amine. With the exception of a single analogue **11**, all members of this subfamily have been synthesised and reported previously by our group and are employed here as a minimalist probe based on simple aromatic amines to establish a baseline contribution of aryl substitution to HDAC8 recognition [48]. Subfamily 2 builds upon this scaffold by introducing functionalised or unfunctionalised heterocyclic amines; all compounds in this series have likewise been reported previously by us and are included to assess the impact of heterocycle‐driven electronic modulation and conformational rigidity [49].

In contrast, Subfamily 3 consists of symmetrical squaramides bearing two aryl or benzylamine substituents and is developed specifically for this study. These compounds had not been synthesised or reported by our group in prior work and were designed to explore the effect of hybridisation at the nitrogen centre designed to explore bidentate binding. Subfamily 4 features fused squaric acid ring systems, in which the squarate core is embedded within a rigid, planar, heteroatomic framework. These derivatives were likewise prepared for the first time in this study and were included to probe the influence of increased molecular rigidity, conformational restriction and extended conjugation on HDAC inhibition.

As part of this study, three derivatives from Subfamily 3 (**14b**, **14c** and **15**) were determined to be novel adducts previously unpublished in the literature. Compound novelty was assessed using SciFinder^n^ (CAS) [50]. For each synthesised derivative, an exact compound structure search was conducted. A compound was considered novel if no matching structures were identified within the database. Conversely, compounds with associated primary literature or patent records (even where no synthetic methodology was reported) were classified as not novel [50].

#### 2.1.1 Synthesis of Monosquarate Amide Derivatives

Subfamilies 1 and 2 comprise the most extensively populated series in this study. The synthesis and full characterisation for these derivatives barring one have been reported by our group previously and are therefore not reiterated here; compound characterisation and isolated yields are provided in the Supporting Information [48, 49].

**FIGURE 5:**
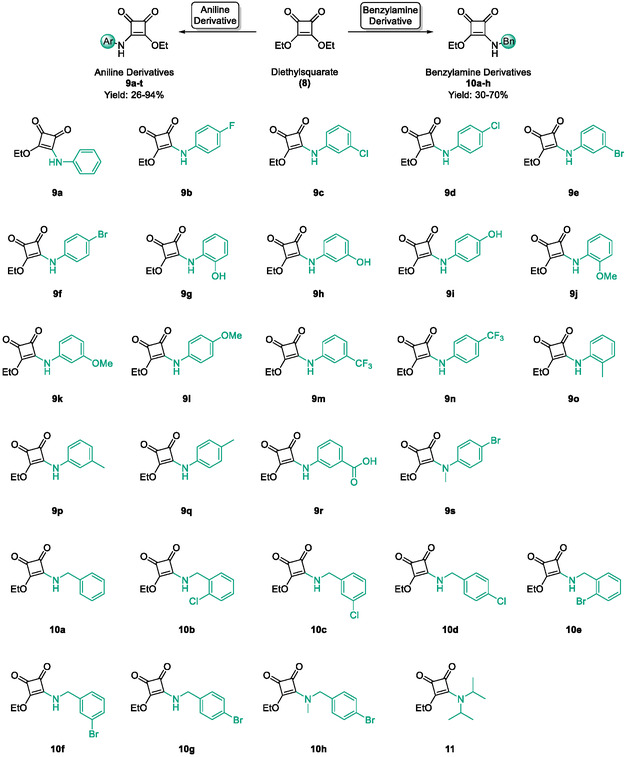
Structures of monosquarate amide derivatives belonging to Subfamily 1 that were evaluated in this study. The majority of these compounds have been reported previously [48].

In brief, selective mono‐substitution of diethyl squarate (DES) (**8**) with aniline, benzylamine or heterocyclic amines afforded the corresponding monosquarate amides, as described previously. Benzylamines generally reacted more readily than anilines, while *ortho* halogenated anilines were not successfully coupled, consistent with steric and electronic constraints. Representative structures are shown below in Figure 5 [48].

In selected cases, Zn(OTf)_2_ was employed to suppress the formation of the undesirable 1,3‐squarine by‐product and hence increase the yield of the desired product [43]. The ability of Zn^2+^ to coordinate squarate carbonyl groups and modulate their reactivity is noteworthy in the context of HDAC inhibition, where a catalytic Zn^2+^ plays a central role in the HDAC8 binding site.

**SCHEME 1:**
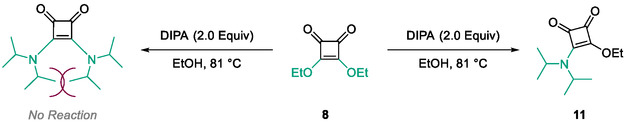
Observed steric influence for the coupling of a bulky aliphatic secondary amine with DES.

Aliphatic monosquarate amide **11** represents the only previously unpublished member of Subfamily 1 by us. Coupling of DES (**8**) with the sterically hindered secondary amine diisopropylamine resulted exclusively in mono‐substitution, even under conditions employing excess amine, highlighting the steric limitations associated with bulky aliphatic nucleophiles (Scheme 1) [37].

**FIGURE 6:**
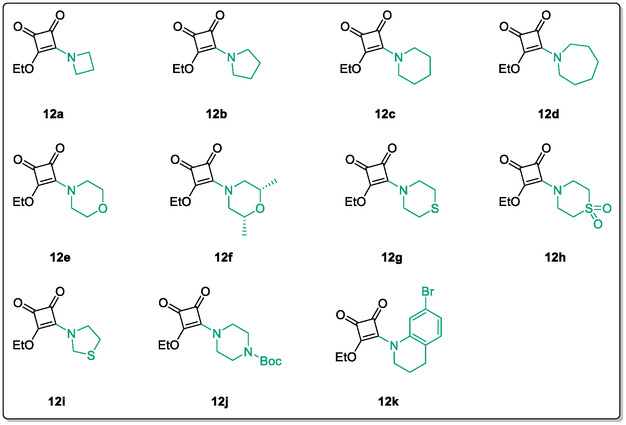
Structures of monosquarate amide derivatives belonging to Subfamily 2 that were evaluated in this study. Synthetic details and characterisation have been reported previously [49].

Monosquarate amides bearing heterocyclic substituents were prepared following the same general mono‐substitution strategy, and their synthesis and characterisation have been described previously. Representative structures are shown in Figure 6 [49].

The inclusion of nitrogen‐containing heterocycles in Subfamily 2 was guided by their prevalence in bioactive compounds and their known influence on solubility, permeability and target interactions [51, 52]. In this study, heterocyclic moieties were introduced to explore how increased conformational flexibility, hydrogen bonding capacity and basicity might affect HDAC8 binding. Unlike the rigid, planar aromatic nature of amines illustrated in Subfamily 1, these saturated heterocycles introduce non‐planarity and electron‐rich nitrogen atoms that may interact differently. This subset was designed to assess whether such structural features support productive binding or disrupt key interactions needed for inhibition.

#### 2.1.2 Synthesis of Squaramides

**SCHEME 2:**
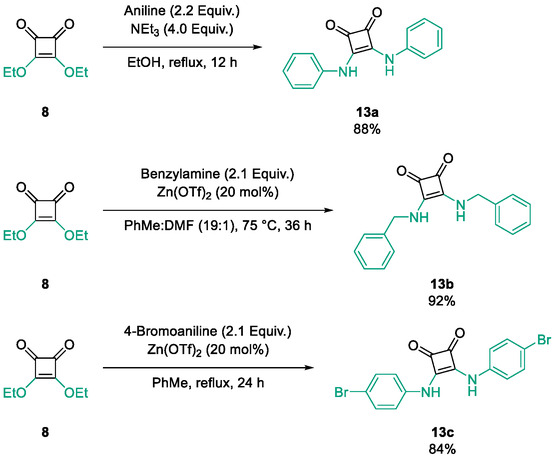
Synthesis of *bis*‐squaramides; coupling DES with aromatic amines [43, 53, 54].

Subfamily 3 comprises nine *bis*‐functionalised squaramides synthesised by introducing two equivalents of amine into the reaction. Initial derivatives **13a** and **13b** were prepared by reacting **8** with two equivalents of aniline or benzylamine, respectively, affording the corresponding *bis*‐squaramide in excellent yields (88% and 92%). The higher yield observed for **13b** is consistent with the greater nucleophilicity of benzylamine relative to aniline and mirror trends observed in our previous published work [48, 53, 54]. To probe the impact of electron‐withdrawing substituents, 4‐bromoaniline was employed to furnish compound **13c** in 84% yield (Scheme 2) [43]. The presence of the bromine atom was intended to explore the potential halogen‐mediated effects on HDAC8 binding by altering the dipole moment across the arene ring or to engage in halogen bonding with electron‐rich residues in the binding pocket. Incorporating a halogen substituent also allows for the assessment of steric effects within the HDAC8 binding pocket

**SCHEME 3:**
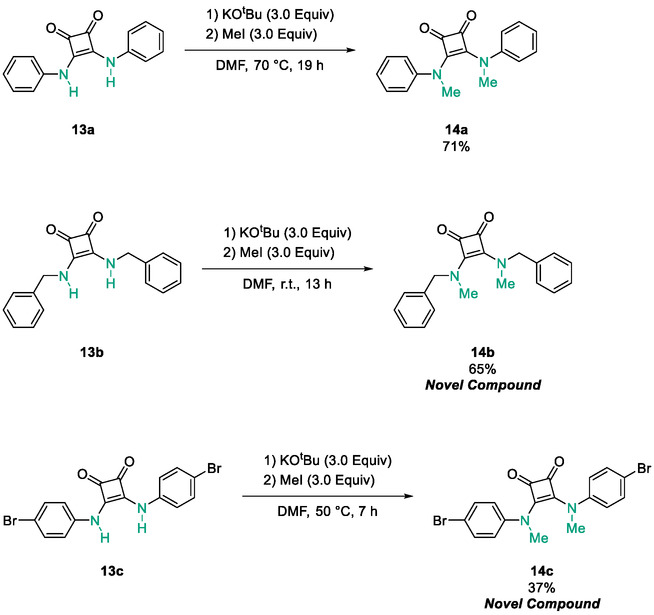
*N*‐Methylation of bis‐squaramides **13a**‐**c** using KO^t^Bu and MeI.

Following the successful preparation of *bis*‐squaramides **13a**‐**c**, late‐stage *N*‐alkylation was investigated to access tertiary squaramide derivatives. This approach was designed to probe potential hydrogen bonding interactions with residues such as Gly151, introduce steric bulk to engage the acetate release channel and the hydrophobic active site pocket and modulate conformational behaviour. Initial alkylation attempts using MeI in the presence of K_2_CO_3_ or NaH in DMF did not result in observable conversion. Although NaH is a strong base, its heterogenous nature and limited solubility under these conditions may restrict effective deprotonation of the amidic NH. Furthermore, it was thought that the rigid and planar nature of the *bis*‐squaramide may hinder deprotonation due to intramolecular hydrogen bonding or steric shielding. Treatment with KO^t^Bu enabled efficient deprotonation, and subsequent addition of MeI afforded clean *N*‐methylation. Using this protocol, the 3° squaramides **14a**‐**c** were isolated in moderate to good yields (37%–71%) (Scheme 3) [54].

**SCHEME 4:**
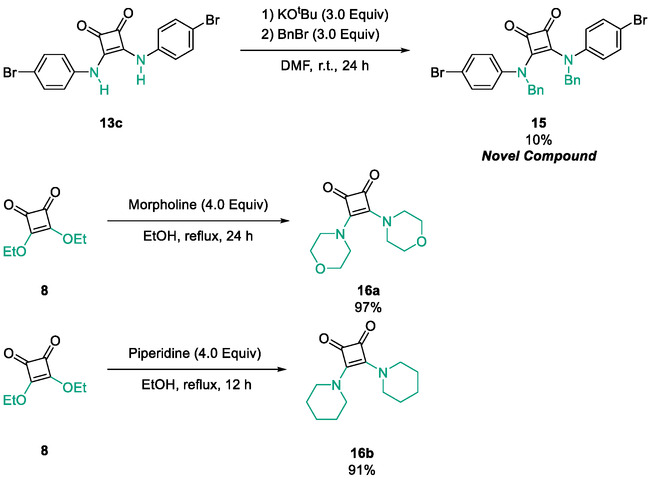
Synthesis of additional *bis*‐squaramide derivatives belonging to Subfamily 3.

To further diversify the series, we carried out benzylation of **13c** using BnBr under the same deprotonation conditions. This transformation was designed to introduce additional steric bulk and hydrophobicity, potentially enhancing interactions with apolar residues in the binding. The *N*‐benzylated product **15** was successfully obtained in modest yield (10%). The reduced efficiency is likely attributed to the intrinsically low nucleophilicity of the squaramide N**H**, diminished by the electron‐withdrawing *para*‐bromoaniline substituent (Scheme 4).

In parallel, two additional *bis*‐squaramides incorporating unfunctionalised heterocyclic amines (morpholine and piperidine) were synthesised allowing for a direct comparison of biological activity between symmetric *bis*‐heterocyclic squaramides and their counterparts in Subfamily 2 (Scheme 4) [54].

#### 2.1.3 Synthesis of Fused Bicycles

**SCHEME 5:**
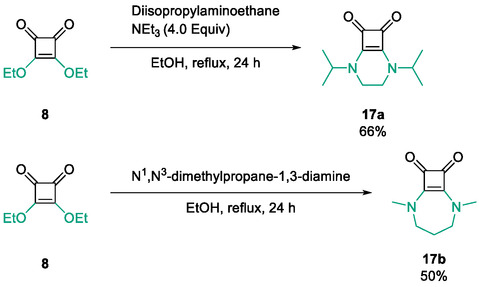
Synthesis of fused squaric acid bicycles belonging to Subfamily 4 [55].

The final compounds prepared in this study comprise two fused squaric acid bicycles, accessed through condensation of **8** with diamine linkers to generate rigid heterobicyclic frameworks [55]. Reactions with *N*,*N*′‐diisopropylethane‐1,2‐diamine afforded compound **17a** (66%), while *N* ^1^,*N* ^3^‐dimethylpropane‐1,3‐diamine yielded compound **17b** (50%) (Scheme 5). These fused derivatives represent the most structurally rigid members of the library of compounds tested and were included to explore how molecular planarity, conformational restriction and nitrogen functionalisation affect protein binding.

### 2.2 Biological Evaluation HDAC Inhibition

Biological profiling commenced with the objective of defining the structural requirements for effective HDAC inhibition by small squaric acid‐derived scaffolds. In line with our design rationale, the squaramide‐based derivatives were considered potential bioisosteric surrogates capable of reproducing the bidentate metal binding interaction typically associated with hydroxamic acid warheads.

In this context, we postulated that the squaric acid scaffold, bearing two carbonyl oxygen atoms capable of coordinating metal centres, could mimic the chelating behaviour of hydroxamates while providing enhanced physicochemical stability. The squaric acid group takes up more space than the relatively small hydroxamate group. Since HDAC8 has a particularly flexible binding pocket and can accommodate structurally diverse inhibitors, we considered this isoenzyme to be a possible target enzyme for squaric acids. HDAC8 is a Class I member characterised by an auxiliary cavity positioned approximately at a right angle to the catalytic pocket. This secondary pocket is proposed to serve as an acetate release channel, offering additional spatial capacity that could accommodate the planar, electron‐deficient squaric acid ring.

Therefore, the first stage of biological evaluation focused on determining inhibitory concentration (IC_50_) values of the squaric acid derivatives against HDAC8 and HDAC4 using purified recombinant enzymes. Enzymatic inhibition was quantified using 10‐point dose–response assays, from which IC_50_ values were calculated (Table 1). HDAC8 and HDAC4 were employed at concentrations of 10 nM and 1 nM, respectively, while other zinc‐dependent HDAC isoenzymes were used at approximate concentrations of ∼1 nM, reflecting the purity of the commercially available preparations. Reference IC_50_ values for SAHA (**2**) and PCI‐34051 (**3**) were taken from previously published studies conducted by our group using identical assay protocols [56, 57].

**TABLE 1:** The structures and inhibitory activities of compounds against HDAC4 and HDAC8.

Compound	**Compound structure**	IC_50_ a ^,^ b, µM	
		**HDAC8**	**HDAC4**
**SAHA** c **2**	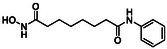	1.9 ± 0.3 [57]	40 ± 2 [57]
**PCI‐34** **051** c **3**	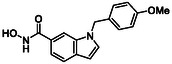	0.024 ± 0.002 [58]	10 ± 1 [58]
**9b**	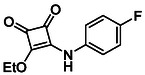	1.20 ± 0.03	8.5 ± 5.3
**14b**	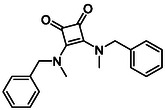	1.3 ± 0.2	14 ± 3
**13b**	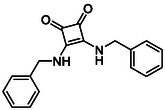	1.8 ± 0.7	4.6 ± 0.9
**13c**	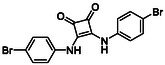	2.0 ± 1.0	11 ± 4
**9a**	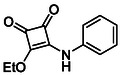	>35	>35
**9c**	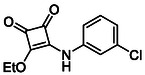	>35	>35
**9d**	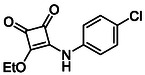	>35	>35
**9e**	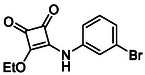	>35	>35
**9f**	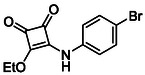	>35	>35
**9g**	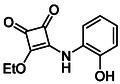	>35	>35
**9** **h**	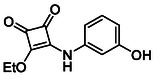	>35	>35
**9i**	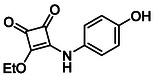	>35	>35
**9j**	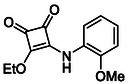	>35	>35
**9k**	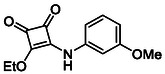	>35	>35
**9l**	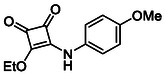	>35	>35
**9m**	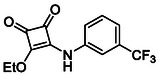	>35	>35
**9n**	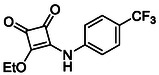	>35	>35
**9o**	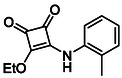	>35	>35
**9p**	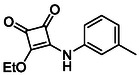	>35	>35
**9q**	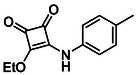	>35	>35
**9r**	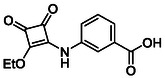	>35	>35
**9s**	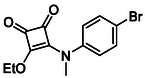	>35	>35
**10a**	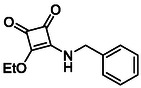	>35	>35
**10b**	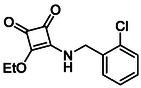	>35	>35
**10c**	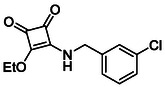	>35	>35
**10d**	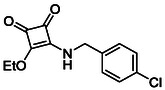	>35	>35
**10e**	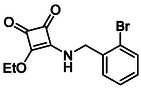	>35	>35
**10f**	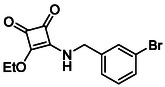	>35	>35
**10g**	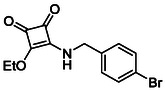	>35	>35
**10** **h**	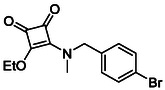	>35	>35
**11**	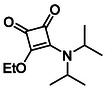	>35	>35
**12a**	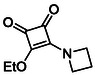	>35	>35
**12b**	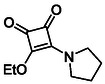	>35	>35
**12c**	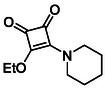	>35	>35
**12d**	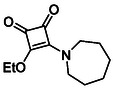	>35	>35
**12e**	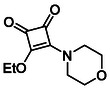	>35	>35
**12f**	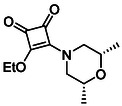	>35	>35
**12g**	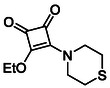	>35	>35
**12** **h**	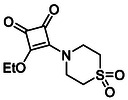	>35	>35
**12i**	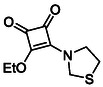	>35	>35
**12j**	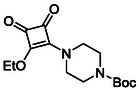	>35	>35
**12k**	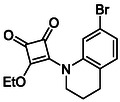	>35	>35
**13a**	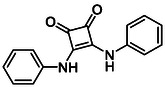	>35	14 ± 2
**14a**	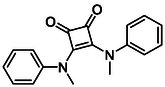	>35	>35
**14c**	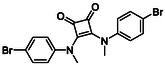	>35	>35
**15**	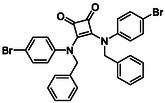	>35	>35
**16a**	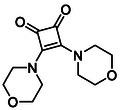	>35	>35
**16b**	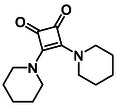	>35	>35
**17a**	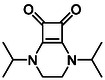	>35	>35
**17b**	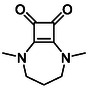	>35	>35

a Data represents mean values of three independent experiments. Standard deviations are calculated based on the results of these experiments.

b Weak inhibitors are indicated by an IC_50_ value greater than 35 µM.

c Included as reference inhibitors for direct comparison.

Following these determinations, the reference inhibitors PCI‐34051 and SAHA displayed IC_50_ values of 0.024 µM and 1.9 µM for HDAC8, respectively [56, 57]. These data are consistent with literature reports and validate the assay performance. Among the synthesised analogues, four compounds **(9b**, **13b**, **13c** and **14b**) showed single‐digit micromolar IC_50_ values against HDAC8 (1.2–2.0 µM), placing them within the same order of magnitude as SAHA for this specific isoenzyme. In contrast, the majority of the remaining analogues displayed IC_50_ values above 35 µM for both isoenzymes.

The active squaric acid derivatives demonstrated consistent selectivity for HDAC8 over HDAC4, with selectivity ratios ranging from five‐ to ten‐fold. Although this selectivity is moderate, the squaric acid motif appears to contribute to preferential HDAC8 inhibition. This observation supports the hypothesis that the squaric acid scaffold is preferentially accommodated by the HDAC8 active site cavity which contains an auxiliary channel adjacent to the zinc‐binding site, known to act as an acetate release pocket [58]. The narrower configuration of the HDAC4 catalytic domain likely limits access from the planar squaric acid motif, resulting in reduced affinity for this specific isoform [59].

The isoenzyme selectivity of the most active compounds described herein was further assessed using a representative panel of zinc‐dependent HDACs encompassing all major classes: Class I (HDAC1 and HDAC8), Class IIa (HDAC4), Class IIb (HDAC6) and Class IV (HDAC11) (Table 2). It was evident that all four compounds tested exhibited a pronounced preference for HDAC8 compared to HDAC1, HDAC4 and particularly HDAC11, whereas selectivity against HDAC6 was less distinct. Collectively, this data confirms that the squaric acid motif is a viable alternative to the hydroxamic acid warhead, capable of producing a potent inhibitor displaying preferential selectivity for HDAC8, thereby validating its potential as a scaffold for further optimisation.

**TABLE 2:** Isoenzyme selectivity of most potent HDAC8 inhibitors tested with purified enzymes.

Compound	**Structure**	IC_50_, µM					IC_50_ (HDAC4)/IC_50_ (HDAC8)
		HDAC1	HDAC4	HDAC6	HDAC8	HDAC11	
**9b**	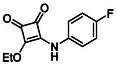	7.0 ± 2.7	8.5 ± 5.3	2.3 ± 1.6	1.20 ± 0.03	25 ± 8	7.1
**14b**	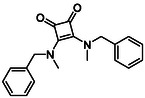	17 ± 16	14 ± 3	6.9 ± 2.5	1.3 ± 0.2	>35	10.8
**13b**	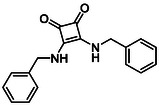	8.3 ± 3.6	4.6 ± 0.9	6.1 ± 3.5	1.8 ± 0.7	>35	2.6
**13c**	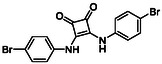	8.1 ± 4.3	11 ± 4	2.9 ± 1.3	2.0 ± 1.0	>35	5.5

### 2.3 In Silico Work

#### 2.3.1 Molecular Docking

**FIGURE 7:**
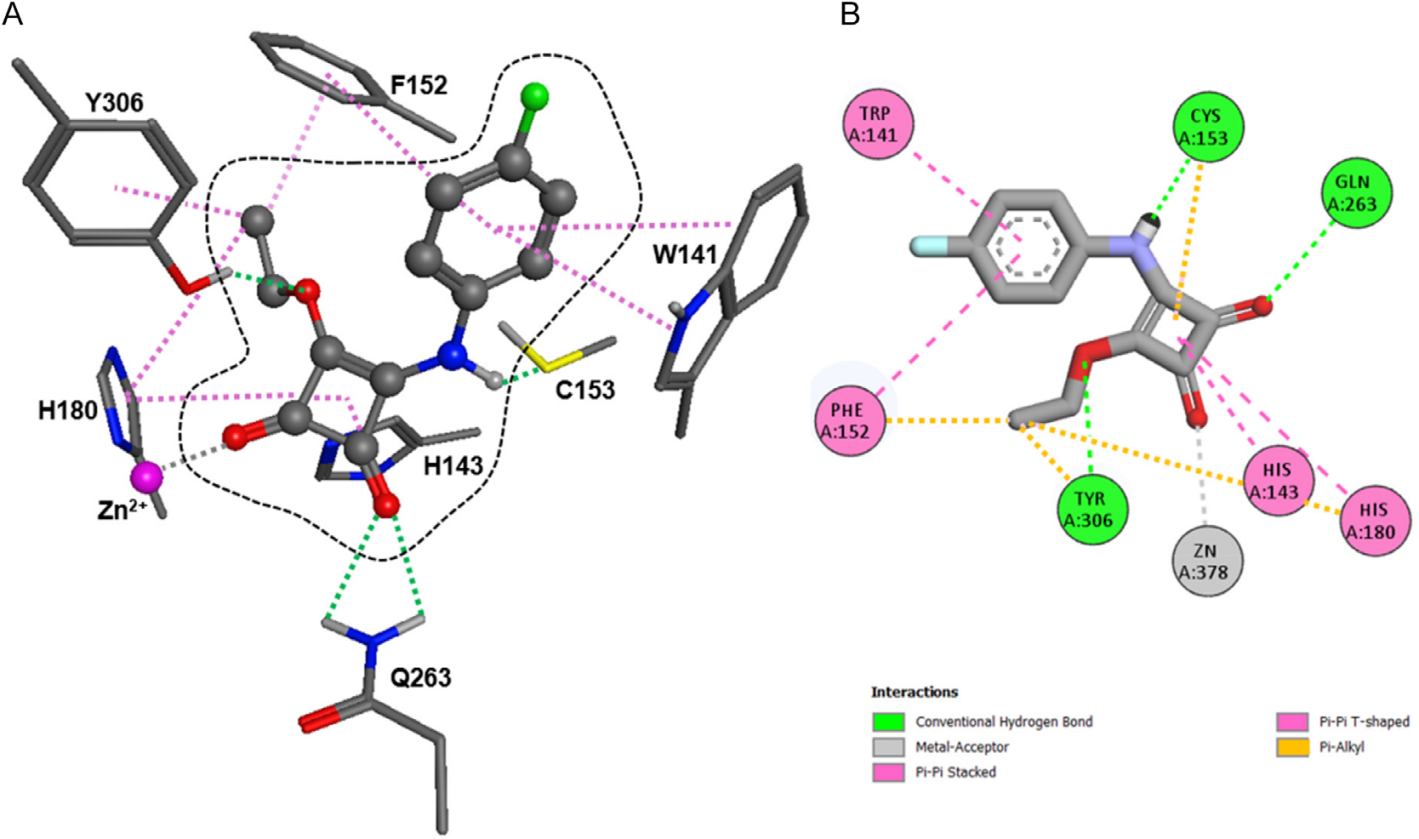
Predicted binding pose of **9b** in HDAC8 (PDB‐ID: 1T69). (A) 3D interactions of **9b** with HDAC8. The ligand is shown as ball and sticks. (B) 2D interactions for better overview. The Interactions are coloured as indicated.

To gain deeper insight into the molecular interactions responsible for HDAC8 inhibition, molecular docking simulations were performed using the most potent analogue, **9b**, in the crystal structure of HDAC8 (PDB‐ID: 1T69) using the Molecular Operating Environment (MOE 2024.0601) software suite. The predicted binding pose is illustrated in Figure 7A and shows that **9b** occupies the canonical substrate‐binding channel, positioning its squaric acid core deep within the catalytic pocket. One carbonyl oxygen atom of the squaric acid core coordinates directly with the catalytic Zn^2+^ ion, while the second carbonyl forms hydrogen bonds with His143 and Tyr306, generating a chelation motif analogous to the bidentate coordination seen in hydroxamate‐based inhibitors such as SAHA [60, 61].

Additional stabilising contacts were identified, including hydrogen bonds with Gln263 and Cys153 and extensive π–π interactions involving His143 and His180 (Figure 7B). These potential interactions firmly anchor the ligand within the hydrophobic tunnel, while π‐stacking between the aromatic ring of **9b** and Phe152 as well as Trp141 reinforces the complex stability. Notably, residues Trp141 and Phe152, which delineate the acetate release channel, appear to facilitate HDAC8 selectivity by offering additional π‐π interactions.

The key amino acid differences between HDAC8 and HDAC1 within the acetate release channel are the substitutions of Trp141 with Leu and Ile34 with Lys, which together reduce the available channel volume for ligand binding in HDAC1. The absence of Trp141's indole ring eliminates favourable π‐stacking interactions with aromatic groups, while the extended lysine side chain reaches deeper into the channel. These structural changes are believed to underlie the reduced binding affinity against HDAC1 observed for compound **9b** [20].

**FIGURE 8:**
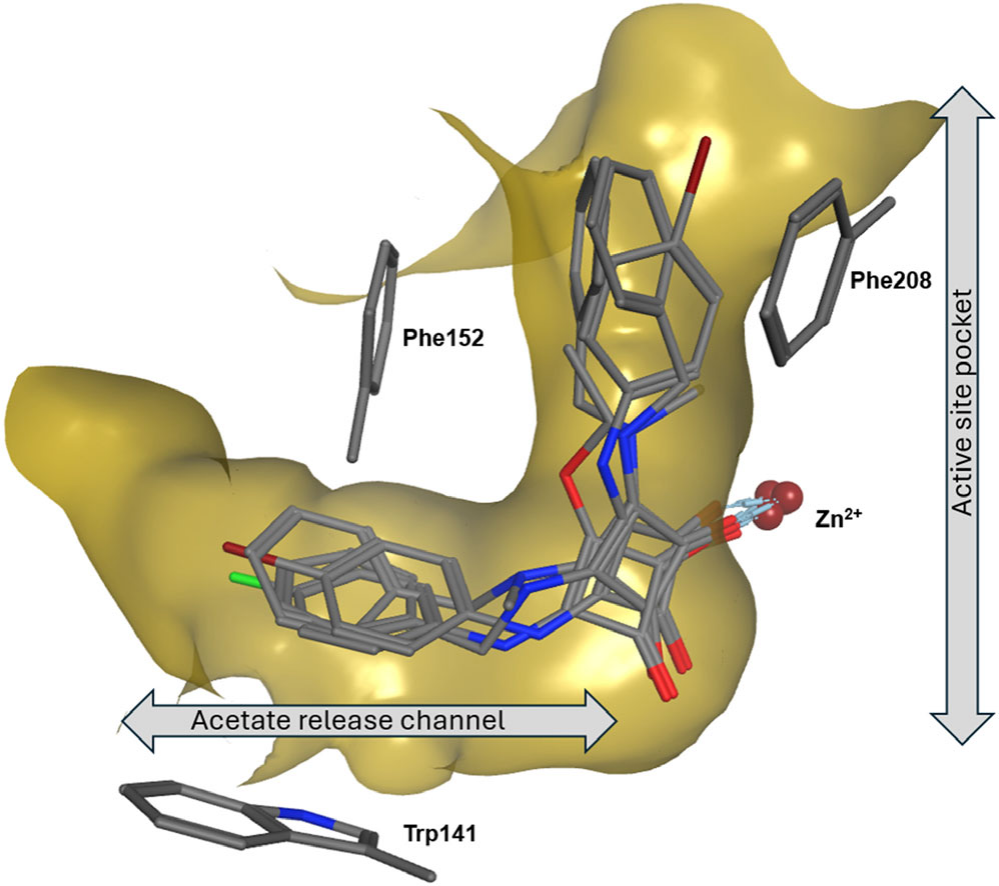
Overlay of binding poses of most potent HDAC8 inhibitors **9b**, **13b**, **13c** and **14b** in HDAC8 (PDB‐ID: 1T69).

Comparative docking of additional active analogues (**13b**, **13c** and **14b**) suggested a conserved binding orientation (Figure 8). In all cases, the squaric acid carbonyls maintain bidentate coordination to the catalytic Zn^2+^ ion, while differences in peripheral substitution alter hydrophobic contacts near the channel entrance. These subtle changes likely account for the small variations in inhibitory potency observed experimentally.

Collectively, these docking results provide a molecular rationale for the observed selectivity and potency trends. The squaric acid motif effectively mimics the canonical hydroxamate warhead, acting as a bidentate cationic Zn^2+^ binding group capable of stable coordination and hydrogen bonding within the HDAC8 catalytic pocket. Moreover, the rigid, planar geometry of the squaric acid scaffold complements the open, flexible conformation of the HDAC8 binding site, supporting the hypothesis that squaric acid derivatives can serve as promising non‐hydroxamic acid scaffolds for isoform selective HDAC inhibition.

#### 2.3.2 Evaluation of Druglikeness Using In Silico Using (SwissADME)

To complement the enzymatic inhibition and molecular docking studies, we employed the free in silico platform SwissADME to predict key druglikeness parameters for the most active squaric acid derivatives identified in this work, alongside benchmark inhibitors PCI‐34051 (**3**) and SAHA (**2**) [62, 63]. A summary of the most relevant physicochemical descriptors is presented in Table 3, while the full dataset is provided in the Supporting Information. The squaric acid derivatives (**9b, 13b**, **13c** and **14b**) satisfied the classical Lipinski criteria, displaying moderate molecular weights (235–422 Da), acceptable hydrogen bonding profiles and consensus cLogP values between 1.7 and 3.4, comparable to PCI‐34051 (cLogP: 2.25).46,47 In most cases, cLogP values were greater than those of the two reference substrates indicating a higher degree of lipophilicity relative to the hydroxamic acid containing inhibitors [64, 65]. As we continue to work on the design and synthesis of future derivatives, we will monitor the predicted lipophilicity of inhibitors [66, 67].

**TABLE 3:** Subset of in silico SwissADME druglikeness experiments.

CMPD	Structure	MW	TPSA	cLogP	BBB Permeable	Lipinski Violations	Brenk Alerts
**9b**	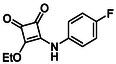	235.2	55.4	1.7	Yes	0	1
**14b**	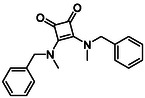	320.3	40.6	2.65	Yes	0	1
**13b**	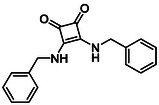	292.3	58.2	2.18	Yes	0	1
**13c**	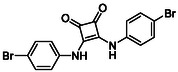	422.1	58.2	3.39	Yes	0	1
**2**	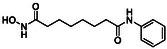	264.3	78.4	1.92	No	0	2
**3**	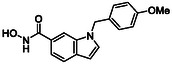	296.3	63.5	2.25	Yes	0	2

The squaric acid derivatives also exhibited moderate polarity, with TPSA values between 40 and 58 Å, markedly below SAHA (**2**) (78 Å) and similar to PCI‐34051 (**3**) (63 Å) [68]. Consistent with these values, all four compounds were predicted to be blood–brain barrier penetrable, mirroring the CNS‐penetrant reference PCI‐34051 (**3**) [69]. These predictions suggest that replacing the hydroxamic acid warhead with a squaric acid‐derived motif does not compromise key permeability‐driven druglikeness parameters and may, in some cases, offer advantages. In addition, Brenk structural alert analysis revealed minimal toxicity‐associated flags for the tested squaric acid derivatives, in contrast to the reference inhibitors, which displayed a greater degree of Brenk alerts [62, 69].

## 3 Discussion

The SAR observed across the four subfamilies reveals a clear trend governing HDAC8 inhibition by squaric acid‐derived analogues. A consistent finding across the dataset is that aliphatic squaramide derivatives show no meaningful activity indicating that hydrophobic aliphatic rings alone are insufficient to engage the HDAC8 binding channel. This suggests that π–π surface‐mediated interactions are a critical requirement for inhibition. Aromatic substitution patterns strongly influenced inhibition potency. *Para* substituted monosquarate amides particularly the *para*‐fluoro (**9b**) derivative outperformed their *meta* or *ortho* substituted counterparts, a trend that can potentially be rationalised by superior accommodation of *para* substituted rings in the narrow acetate release channel adjacent to the catalytic Zn site. Docking studies support this interpretation; the aromatic ring of compound **9b**, for example, adopts a binding post stabilised by the π–π stacking with Trp141, a well‐known aromatic anchor within this pocket. The *para*‐fluoro analogue (**9b**) is particularly favoured, achieving low‐energy binding due to its small steric footprint and optimal electronic profile, whereas bulkier *para* substituents such as *para*‐trifluoromethyl (**9n**) and *para*‐bromo (**9f**) substantially reduce inhibition.

Notably, while halogenated aryl groups have been shown to occupy the acetate release channel in crystallographic studies of HDAC8 inhibitors, the pronounced loss of activity observed for the *para*‐chloro analogue (**9d**) and *para*‐bromo analogue (**9f**) in this series likely reflects subtle steric and dynamic effects within the highly flexible HDAC8 active site that are not readily captured by docking approaches based on static protein structures.

Structural expansion of the core squaric acid scaffold to generate squaramides proved advantageous in the number of active compounds analysed. This enhancement may arise from improved occupancy of the extended binding channel, permitting secondary π–π contacts with Phe152 and Phe208, which flank the active site. Interestingly, despite these additional stabilising interactions, the inhibitory potencies of *bis*‐squarmides **13b**, **13c** and **14b** fall within a similar range to that of monosquarate amide **9b**. One plausible explanation is that the additional aromatic interactions in the *bis*‐squaramides are offset by entropic penalties arising from increased molecular rigidity and reduced conformational freedom upon binding. Furthermore, accommodation of a second aromatic substituent within the narrow and conformational dynamic acetate release channel of HDAC8 may require subtle protein rearrangements that partially counterbalance the energetic gains from additional π–π stacking.

Interestingly, although monosquarate amide esters favoured aniline substituents over benzylamines in a single point screen, some functionalised dibenzylamine squaramides such as **13b** and **14b** were among the most potent inhibitors identified. These derivatives likely induce subtly different conformational adjustments within the active site relative to monosquarate amides enabling them to exploit alternative hydrophobic contacts with HDAC8 channel architecture.

Taken together, these SAR trends indicate that an aromatic ring is essential for HDAC8 binding, that *para* substitution optimises channel fit and π‐stacking, and that extending aromatic surface area can yield further potency gains by engaging secondary hydrophobic pockets. Based on these findings, future optimisation efforts should prioritise *para*‐fluorinated motifs as a lead scaffold, exploring systematic variation of the second substituent particularly aromatic groups on the monosquarate ester to balance binding affinity, selectivity and physicochemical properties.

## 4 Conclusion and Future Work

This work presents a comprehensive study into the design, synthesis and biological evaluation of squaric acid derivatives as potential HDAC8 inhibitors. Motivated by the limitations of hydroxamic acid warheads, including their poor metabolic stability and off‐target metal chelation, we investigated the squaric acid motif as a novel bioisostere capable of engaging the catalytic Zn^2+^ while offering improved physicochemical properties. A library of 51 compounds were prepared and organised into four subfamilies based on core structural features. Subfamilies 1 and 2, comprising monosquarate amides functionalised with aromatic amines or heterocycles, largely drew on scaffolds previously reported by our group; however, their HDAC inhibitory activity and isoform selectivity are reported here for the first time. In contrast, subfamilies 3 and 4, featuring *bis*‐squaramides and fused bicyclic architectures, were developed specifically for this study and expand the structural diversity explored for HDAC inhibition. This strategic subdivision enabled a systematic SAR analysis across a chemical series.

Biological evaluation initially began with testing for HDAC inhibition on HDAC4 and HDAC8 isoenzymes to establish if the compounds were active or inactive. Several hits were identified in this initial study with low micromolar inhibitory activity displaced against HDAC8 and HDAC4. The most notable IC_50_ values were obtained for compound **9b** (Subfamily 1) and compound **13c** (Subfamily 3). Further, inhibitory activity was also observed in the case of **13b** and **14b** also belonging to Subfamily 3. Compounds that possessed promising activity in the initial study were then subjected to isoenzyme‐specific assays which revealed promising specificity towards HDAC8 over other Zn^2+^‐dependent HDACs. Molecular docking studies have also been conducted in order to provide a mechanistic rationale for the activity observed. In such studies, it was revealed that the carbonyl groups of the squaric acid core could effectively coordinate to the catalytic Zn^2+^ ion.

The findings presented in this study confirm that the squaric acid motif can act as a viable ZBG and highlight its potential to replace the classically used hydroxamic acid warhead in the development of isoenzyme‐specific HDACIs. The results presented here provide a robust platform for next‐generation HDAC8 selective inhibitors based on a squaric acid framework. Our SAR studies highlight several promising avenues for future optimisation including the preference for *para* substituted anilines that exploit the acetate release channel, high activity associated with *bis*‐aryl squaramides capable of multiple π‐stacking interactions and the ability of the core squaric acid motif to coordinate with the catalytic Zn^2+^ species via the two carbonyls of the core ring. These insights collectively define a coherent blueprint for advancing the squaric acid series towards developing more potent, selective and pharmacologically favourable HDAC8 inhibitors. Further profiling of selected compounds in functional assays will be reported on in due course.

## Supporting Information

Additional supporting information can be found online in the Supporting Information section.

## Conflicts of Interest

The authors declare no conflicts of interest.

## Supporting information

Supplementary Material

## Data Availability

The data that supports the findings of this study are available in the supplementary material of this article.

## References

[1] T. Narita , B. T. Weinert , and C. Choudhary , “Functions and Mechanisms of Non‐Histone Protein Acetylation,” Nature Reviews Molecular Cell Biology 20 (2019): 156–174. 30467427 10.1038/s41580-018-0081-3

[2] E. Verdin and M. Ott , “50 Years of Protein Acetylation: From Gene Regulation to Epigenetics, Metabolism and beyond,” Nature Reviews Molecular Cell Biology 16 (2015): 258–264. 25549891 10.1038/nrm3931

[3] M. Yoshida , N. Kudo , S. Kosono , A. Ito , “Chemical and Structural Biology of Protein Lysine Deacetylases,” Proceedings of the Japan Academy, Series B 93 (2017): 297–321. 10.2183/pjab.93.019 PMC5489435 28496053

[4] Y. Jiang , J. Liu , D. Chen , L. Yan , and W. Zheng , “Sirtuin Inhibition: Strategies, Inhibitors, and Therapeutic Potential,” Trends in Pharmacological Sciences 38 (2017): 459–472. 28389129 10.1016/j.tips.2017.01.009

[5] T. C. S. Ho , A. H. Y. Chan , and A. Ganesan , “Thirty Years of HDAC Inhibitors: 2020 Insight and Hindsight,” Journal of Medicinal Chemistry 63 (2020): 12460–12484. 32608981 10.1021/acs.jmedchem.0c00830

[6] X. Guo , H. Ruan , X. Li , et al., “Subcellular Localization of Class I Histone Deacetylases in the Developing Xenopus Tectum,” Frontiers in Cellular Neuroscience, 10.3389/FNCEL.2015.00510. PMC4709447 26793062

[7] M. Haberland , R. L. Montgomery , and E. N. Olson , “The Many Roles of Histone Deacetylases in Development and Physiology: Implications for Disease and Therapy,” Nature Reviews Genetics 10 (2009): 32–42. 10.1038/nrg2485 PMC3215088 19065135

[8] I. Van Den Wyngaert , W. De Vries , A. Kremer , et al., “Cloning and Characterization of Human Histone Deacetylase 8,” FEBS Letters 478 (2000): 77–83. 10922473 10.1016/s0014-5793(00)01813-5

[9] M. A. Deardorff , M. Bando , R. Nakato , et al., “HDAC8 Mutations in Cornelia De Lange Syndrome Affect the Cohesin Acetylation Cycle,” Nature 489 (2012): 313–317. 22885700 10.1038/nature11316 PMC3443318

[10] J. Tang , H. Yan , and S. Zhuang , “Histone Deacetylases as Targets for Treatment of Multiple Diseases,” Clinical Science 124 (2013): 651. 23414309 10.1042/CS20120504 PMC4568123

[11] I. Oehme , H. E. Deubzer , D. Wegener , et al., “Histone Deacetylase 8 in Neuroblastoma Tumorigenesis,” Clinical Cancer Research 15 (2009): 91–99. 19118036 10.1158/1078-0432.CCR-08-0684

[12] S. Balasubramanian , J. Ramos , W. Luo , M. Sirisawad , E. Verner , and J. J. Buggy , “A Novel Histone Deacetylase 8 (HDAC8)‐Specific Inhibitor PCI‐34051 Induces Apoptosis in T‐Cell Lymphomas,” Leukemia 22 (2008): 1026–1034. 18256683 10.1038/leu.2008.9

[13] M. S. Finnin , J. R. Donigian , A. Cohen , et al., “Structures of a Histone Deacetylase Homologue Bound to the TSA and SAHA Inhibitors,” Nature 401 (1999): 188–193. 10490031 10.1038/43710

[14] K. KrennHrubec , B. L. Marshall , M. Hedglin , E. Verdin , and S. M. Ulrich , “Design and Evaluation of ‘Linkerless’ Hydroxamic Acids as Selective HDAC8 Inhibitors,” Bioorganic and Medicinal Chemistry Letters 17 (2007): 2874–2878. 17346959 10.1016/j.bmcl.2007.02.064

[15] J. R. Somoza , R. J. Skene , B. A. Katz , et al., “Structural Snapshots of Human HDAC8 Provide Insights into the Class I Histone Deacetylases,” Structure 12 (2004): 1325–1334. 15242608 10.1016/j.str.2004.04.012

[16] K. Long , D. A. Close , P. A. Johnston , and D. M. Huryn , “Replacement of the Hydroxamic Acid Group in the Selective HDAC8 Inhibitor PCI‐34051,” Bioorganic and Medicinal Chemistry Letters 108 (2024): 129810. 38782078 10.1016/j.bmcl.2024.129810 PMC11212034

[17] I. Rettig , E. Koeneke , F. Trippel , et al., “Selective Inhibition of HDAC8 Decreases Neuroblastoma Growth In Vitro and In Vivo and Enhances Retinoic Acid‐Mediated Differentiation,” Cell Death and Disease 6 (2015): e1657–e1657. 25695609 10.1038/cddis.2015.24 PMC4669789

[18] V. K. Shukla , L. Siemons , and D. F. Hansen , “Intrinsic Structural Dynamics Dictate Enzymatic Activity and Inhibition,” Proceedings of the National Academy of Sciences of the United States of America 120 (2023): e2310910120. 37782780 10.1073/pnas.2310910120 PMC10576142

[19] J. Melesina , C. V. Simoben , L. Praetorius , E. F. Bülbül , D. Robaa , and W. Sippl , “Strategies To Design Selective Histone Deacetylase Inhibitors,” ChemMedChem 16 (2021): 1336–1359. 33428327 10.1002/cmdc.202000934

[20] L. Whitehead , M. R. Dobler , B. Radetich , et al., “Human HDAC Isoform Selectivity Achieved via Exploitation of the Acetate Release Channel with Structurally Unique Small Molecule Inhibitors,” Bioorganic and Medicinal Chemistry 19 (2011): 4626–4634. 21723733 10.1016/j.bmc.2011.06.030

[21] A. Vannini , C. Volpari , G. Filocamo , et al., “Crystal Structure of a Eukaryotic Zinc‐Dependent Histone Deacetylase, Human HDAC8, Complexed with a Hydroxamic Acid Inhibitor,” Proceedings of the National Academy of Sciences of the United States of America 101 (2004): 15064–15069. 15477595 10.1073/pnas.0404603101 PMC524051

[22] D. F. Wang , O. Wiest , P. Helquist , H. Y. Lan‐Hargest , and N. L. Wiech , “On the Function of the 14 Å Long Internal Cavity of Histone Deacetylase‐Like Protein: Implications for the Design of Histone Deacetylase Inhibitors,” Journal of Medicinal Chemistry 47 (2004): 3409–3417. 15189037 10.1021/jm0498497

[23] M. Marek , T. B. Shaik , T. Heimburg , et al., “Characterization of Histone Deacetylase 8 (HDAC8) Selective Inhibition Reveals Specific Active Site Structural and Functional Determinants,” Journal of Medicinal Chemistry 61 (2018): 10000–10016. 30347148 10.1021/acs.jmedchem.8b01087

[24] P. L. Skipper , S. R. Tannenbaum , W. G. Thilly , E. E. Furth , W. W. Bishop , “Mutagenicity of Hydroxamic Acids and the Probable Involvement of Arbamoylation,” Cancer Research 40 (1980): 4704–4708. 7002295

[25] A. Friedrich , A. S. Assmann , L. Schumacher , et al., “In Vitro Assessment of the Genotoxic Hazard of Novel Hydroxamic Acid‐ and Benzamide‐Type Histone Deacetylase Inhibitors (HDACi),” International Journal of Molecular Sciences 21 (2020): 4747. 32635356 10.3390/ijms21134747 PMC7370100

[26] S. Shen and A. P. Kozikowski , “Why Hydroxamates May Not Be the Best Histone Deacetylase Inhibitors—What Some May Have Forgotten or Would Rather Forget?,” ChemMedChem 11 (2016): 15–21. 26603496 10.1002/cmdc.201500486 PMC4765907

[27] S. Lechner , M. I. P. Malgapo , C. Grätz , et al., “Target Deconvolution of HDAC Pharmacopoeia Reveals MBLAC2 as Common Off‐Target,” Nature Chemical Biology 18 (2022): 812–820. 35484434 10.1038/s41589-022-01015-5 PMC9339481

[28] J. Liu , Y. Yu , J. Kelly , et al., “Discovery of Highly Selective and Potent HDAC3 Inhibitors Based on a 2‐Substituted Benzamide Zinc Binding Group,” ACS Medicinal Chemistry Letters 11 (2020): 2476–2483. 33335670 10.1021/acsmedchemlett.0c00462 PMC7734795

[29] J. He , S. Wang , X. Liu , et al., “Synthesis and Biological Evaluation of HDAC Inhibitors With a Novel Zinc Binding Group,” Frontiers in Chemistry 8 (2020): 256. 32351936 10.3389/fchem.2020.00256 PMC7174758

[30] P. Sun , J. Wang , K. S. Khan , et al., “Development of Alkylated Hydrazides as Highly Potent and Selective Class I Histone Deacetylase Inhibitors with T Cell Modulatory Properties,” Journal of Medicinal Chemistry 65 (2022): 16313–16337. 36449385 10.1021/acs.jmedchem.2c01132

[31] S. Geurs , D. Clarisse , K. De Bosscher , and M. D’hooghe , “The Zinc‐Binding Group Effect: Lessons From Non‐Hydroxamic Acid Vorinostat Analogs,” Journal of Medicinal Chemistry 66 (2023): 7698–7729. 37276138 10.1021/acs.jmedchem.3c00226

[32] S. Hanessian , V. Vinci , L. Auzzas , M. Marzi , and G. Giannini , “Exploring Alternative Zn‐Binding Groups in the Design of HDAC Inhibitors: Squaric Acid, N‐Hydroxyurea, and Oxazoline Analogues of SAHA,” Bioorganic & Medicinal Chemistry Letters 16 (2006): 4784–4787. 16870438 10.1016/j.bmcl.2006.06.090

[33] S. Cohen and J. D. Park , “Diketocyclobutenediol,” Journal of the American Chemical Society 81 (1959): 3480.

[34] F. R. Wurm and H. A. Klok , “Be Squared: Expanding the Horizon of Squaric Acid‐Mediated Conjugations,” Chemical Society Reviews 42 (2013): 8220–8236. 23873344 10.1039/c3cs60153f

[35] H. Liu , C. S. Tomooka , and H. W. Moore , “An Efficient General Synthesis of Squarate Esters,” Synthetic Communications 27 (1997): 2177–2180.

[36] A. H. Schmidt , “Reaktionen von Quadratsäure Und Quadratsäure‐Derivaten,” Synthesis (1980): 961–994.

[37] L. F. Tietze , M. Arlt , M. Beller , K.‐H. Gl üsenkamp , E. Jähde , and M. F. Rajewsky , “Anticancer Agents, 15. Squaric Acid Diethyl Ester: A New Coupling Reagent for the Formation of Drug Biopolymer Conjugates. Synthesis of Squaric Acid Ester Amides and Diamides,” Chemische Berichte 124 (1991): 1215–1221.

[38] P. H. Tedesco and H. F. Walton , “Metal Complexes of Squaric Acid (diketocyclobutenediol) in Aqueous Solution,” Inorganic Chemistry 8 (1969): 932–937.

[39] Y. Nikolova , G. M. Dobrikov , Z. Petkova , and P. Shestakova , “Chiral Aminoalcohols and Squaric Acid Amides as Ligands for Asymmetric Borane Reduction of Ketones: Insight to In Situ Formed Catalytic System by DOSY and Multinuclear NMR Experiments,” Molecules 26 (2021): 6865. 34833957 10.3390/molecules26226865 PMC8624562

[40] H. F. Schaeffer , “Squaric Acid: Reactions with Certain Metals,” Microchemical Journal 17 (1972): 443–455.

[41] J. Chasák , V. Šlachtová , M. Urban , and L. Brulíková , “Squaric Acid Analogues in Medicinal Chemistry,” European Journal of Medicinal Chemistry 209 (2021): 112872, 10.1016/j.ejmech.2020.112872. 33035923

[42] M. Berney , W. Doherty , W. T. Jauslin , M. T. Manoj , E. M. Dürr , and J. F. McGouran , “Synthesis and Evaluation of Squaramide and Thiosquaramide Inhibitors of the DNA Repair Enzyme SNM1A,” Bioorganic and Medicinal Chemistry 46 (2021): 116369, 10.1016/j.bmc.2021.116369. 34482229 PMC8607331

[43] A. Rostami , A. Colin , X. Y. Li , M. G. Chudzinski , A. J. Lough , and M. S. Taylor , “ *N*, *N′* ‐Diarylsquaramides: General, High‐Yielding Synthesis and Applications in Colorimetric Anion Sensing,” Journal of Organic Chemistry 75 (2010): 3983–3992. 20486682 10.1021/jo100104g

[44] P. R. Palme , S. Grover , R. Abdelaziz , et al., Journal of Medicinal Chemistry, 10.1021/ACS.JMEDCHEM.5C02284/SUPPL_FILE/JM5C02284_SI_002.CSV. PMC12703743 41277442

[45] W. Gonsiorek , X. Fan , D. Hesk , et al., “Pharmacological Characterization of Sch527123, a Potent Allosteric CXCR1/CXCR2 Antagonist,” Journal of Pharmacology and Experimental Therapeutics 322 (2007): 477–485. 17496166 10.1124/jpet.106.118927

[46] C. Ballatore , D. M. Huryn , A. B. Smith , “Carboxylic Acid(bio)isosteres in Drug Design,” ChemMedChem 8 (2013): 385–395. 23361977 10.1002/cmdc.201200585 PMC3640829

[47] P. Lassalas , B. Gay , C. Lasfargeas , et al., “Structure Property Relationships of Carboxylic Acid Isosteres,” Journal of Medicinal Chemistry 59 (2016): 3183–3203. 26967507 10.1021/acs.jmedchem.5b01963 PMC4833640

[48] N. Long , A. L. Gresley , A. Solomonsz , A. Wozniak , S. Brough , S. P. Wren , “Synthesis of Squaric Acid Monoamides as Building Blocks for Drug Discovery,” SynOpen 07 (2023): 401–407.

[49] N. Long , A. L. Gresley , A. Wozniak , S. Brough , and S. P. Wren , “Synthesis and Evaluation of Druglike Parameters via in Silico Techniques for a Series of Heterocyclic Monosquarate‐Amide Derivatives as Potential Carboxylic Acid Bioisosteres,” Bioorganic and Medicinal Chemistry 98 (2024): 117565. 38142561 10.1016/j.bmc.2023.117565

[50] Search, CAS SciFinder , accessed July 15, 2025, https://scifinder‐n.cas.org/.

[51] J. Jampilek , “Heterocycles in Medicinal Chemistry,” Molecules 24 (2019): 10–13. 10.3390/molecules24213839 PMC6864827 31731387

[52] N. Kerru , L. Gummidi , S. Maddila , K. K. Gangu , S. B. Jonnalagadda , “A Review on Recent Advances in Nitrogen‐Containing Molecules and Their Biological Applications,” Molecules 25 (2020): 1909–1951, 10.3390/molecules25081909. 32326131 PMC7221918

[53] V. E. Zwicker , K. K. Y. Yuen , D. G. Smith , et al., “Deltamides and Croconamides: Expanding the Range of Dual H‐bond Donors for Selective Anion Recognition,” Chemistry – A European Journal 24 (2018): 1140–1150. 29119615 10.1002/chem.201704388

[54] H. Ehrhardt , S. Hünig , and H. Pütter , “Amide Und Thioamide Der Quadratsäure: Synthese Und Reaktionen,” Chemische Berichte 110 (1977): 2506–2523.

[55] M. E. Baumert , V. Le , P. H. Su , et al., “From Squaric Acid Amides (SQAs) to Quinoxaline‐Based SQAs–Evolution of a Redox‐Active Cathode Material for Organic Polymer Batteries,” Journal of the American Chemical Society 145 (2023): 23334–23345. 37823604 10.1021/jacs.3c09153

[56] M. Schweipert , A. Amurthavasan , F. J. Meyer‐Almes , “Continuous Enzyme Activity Assay for High‐Throughput Classification of Histone deacetylase 8 inhibitors,” Exploration of Targeted Anti‐tumor Therapy 4 (2023): 447–459. 37455831 10.37349/etat.2023.00144 PMC10344891

[57] A. Kleinschek , C. Meyners , E. Digiorgio , C. Brancolini , F. J. Meyer‐Almes , “Potent and Selective Non‐Hydroxamate Histone deacetylase 8 inhibitors,” ChemMedChem 11 (2016): 2598–2606. 27860422 10.1002/cmdc.201600528

[58] Y. Luo , Z. Yan , X. Chu , Y. Zhang , Y. Qiu , and H. Li , “Binding Mechanism and Distant Regulation of Histone Deacetylase 8 by PCI‐34051,” Communications Biology 8 (2025): 221. 39939814 10.1038/s42003-025-07649-0 PMC11821889

[59] C. A. Luckhurst , P. Breccia , A. J. Stott , et al., “Potent, Selective, and CNS‐Penetrant Tetrasubstituted Cyclopropane Class IIa Histone Deacetylase (HDAC) Inhibitors,” ACS Medicinal Chemistry Letters 7 (2016): 34–39. 26819662 10.1021/acsmedchemlett.5b00302 PMC4716601

[60] A. Vannini , C. Volpari , P. Gallinari , et al., “Substrate Binding to Histone Deacetylases as Shown by the Crystal Structure of the HDAC8–substrate Complex,” EMBO reports 8 (2007): 879. 17721440 10.1038/sj.embor.7401047 PMC1973954

[61] G. Estiu , N. West , R. Mazitschek , E. Greenberg , J. E. Bradner , and O. Wiest , “On the Inhibition of Histone Deacetylase 8,” Bioorganic and Medicinal Chemistry 18 (2010): 4103. 20472442 10.1016/j.bmc.2010.03.080 PMC3245874

[62] A. Daina , O. Michielin , and V. Zoete , Scientific Reports 7 (2017): 42717. 28256516 10.1038/srep42717 PMC5335600

[63] SwissADME , accessed November 28, 2025, https://www.swissadme.ch/.

[64] C. A. Lipinski , F. Lombardo , B. W. Dominy , and P. J. Feeney , “Experimental and Computational Approaches to Estimate Solubility and Permeability in Drug Discovery and Development Settings,” Advanced Drug Delivery Reviews 23 (1997): 3–25. 10.1016/s0169-409x(00)00129-0 11259830

[65] S. B. Bunally , C. N. Luscombe , and R. J. Young (SAGE Publications Inc, 2019), preprint, 10.1177/2472555219859845.

[66] S. B. Bunally , C. N. Luscombe , and R. J. Young , “Using Physicochemical Measurements to Influence Better Compound Design,” SLAS Discovery 24 (2019): 791–801. 31429385 10.1177/2472555219859845

[67] P. N. Mortenson , C. W. Murray , Assessing the Lipophilicity of Fragments and Early Hits, Journal of Computer‐Aided Molecular Design 25, no. 7 (2011): 663–667. 21614595 10.1007/s10822-011-9435-z

[68] P. Matsson and J. Kihlberg , “How Big Is Too Big for Cell Permeability?,” Journal of Medicinal Chemistry 60 (2017): 1662–1664. 28234469 10.1021/acs.jmedchem.7b00237

[69] C. Jamieson , E. M. Moir , Z. Rankovic , and G. Wishart , “Medicinal Chemistry of hERG Optimizations: Highlights and Hang‐Ups,” Journal of Medicinal Chemistry 49 (2006): 5029–5046. 16913693 10.1021/jm060379l

