# Supplementary material for: Fair and Square: Design, Synthesis and Biological Evaluations of Squaric Acid Derivatives as Novel HDAC8 Inhibitors
Source: ChemMedChem. 2026 Apr 25;21(8):e70270. doi: 10.1002/cmdc.70270 (PMC13110267; doi:10.1002/cmdc.70270)
Supplement: Supplementary file 1 — Supplementary Material [file CMDC-21-e70270-s001.pdf]

Fair and Square: design, synthesis and biological evaluations of squaric acid derivatives as novel HDAC8 inhibitors.

N. Long,[a*] F. J. Meyer-Almes,[b*] A. Kopranovic,[b] S. P. Wren [a*]

[a] School of Life Sciences, Pharmacy and Chemistry, Faculty of Health, Science, Social Care and Education, Kingston University London, Penrhyn Road, Kingston, Surrey, KT1 2EE

[b] Department of Chemical Engineering and Biotechnology, University of Applied Sciences Darmstadt, Haardtring 100, 64295 Darmstadt, Germany

* Email: k2059435@kingston.ac.uk (Nathan Long)
franz-josef.meyer-almes@h-da.de (Franz-Josef Meyer-Almes)
S.wren@kingston.ac.uk (Stephen P. Wren)

Supporting Information

Contents:

**1.1**	**Chemistry: General Information**	2
**1.2**	**Compound Characterisation and Synthetic Procedures**	3
**1.3**	**HDAC Assay**	22
**1.4**	**Molecular Docking**	26
**1.4.1**	**Swiss ADME *In silico* Data**	27
	**References**	28
	**NMR Data**	29-128

1 Experimental Section

1.1 Chemistry: General Information

The reactions detailed in this paper were conducted in oven-dried/flame dried glassware unless otherwise specified and under an inert atmosphere. Commercially available reagents purchased from Sigma Aldrich, Acros Organic, Fluorochem, TCl Chemicals, or Alfa Aesar were used without further purification. Reactions which are denoted to be moisture sensitive were carried out using dry solvents. Dry solvents were either purchased from commercial sources or dried *in situ* by appropriate methodology.[1] Analytical thin layer chromatography (TLC) was performed on silica gel plates (Merck 60 Å, F_{254}, aluminium backed) visualised either with aid of a UV lamp (254 nm) or by using staining solutions (Vanillin/$KMnO_4$/Anisaldehyde) followed by heating. Flash column chromatography (FCC) was performed on silica gel (60-230 mesh). Dry loading for FCC used either the afore mentioned silica gel or Celite®. Organic extracts were dried over anhydrous Na_2SO_4, $MgSO_4$ or molecular sieves (4 or 6 Å). All samples were dried *via* a high vacuum pump (Vacuubrand, Rotary vane pump RZ 2.5, 4 x 10^{-4} mbar).

^{1}H NMR spectra were recorded on a Bruker AV400/HD400 or AV600 at 400 MHz and 600 MHz respectively in an appropriate deuterated solvent. Observed signals are reported as follows: chemical shift in parts per million from tetramethylsilane with the solvent as an internal standard ($CDCl_3$ δ 7.26 ppm), ($(CD_3)_2CO$ δ 2.05 ppm), ($(CD_3)_2SO$ δ 2.50 ppm) (CD_3CN δ 1.94 ppm) (CD_3OD δ 3.31 ppm), multiplicity (s = singlet, d = doublet, t = triplet, q = quartet, dd = doublet of doublets, and m = multiplet or overlap of non-equivalent resonances, br = broad), integration. ^{13}C NMR spectra were recorded at 100 MHz in an appropriate deuterated solvent and the observed signals were reported in the same

format as that presented for ^{1}H NMR data: chemical shift in parts per million. Coupling constants (*J*) are reported in Hertz (Hz). All ^{1}H NMR spectra were obtained at rt, and ^{13}C NMR experiments when run in $(CD_3)_2SO$ were obtained at 318 K. Sample melting point was determined using Gallenkamp melting point apparatus MPD350.BM2.5. HRMS analyses were carried out on a Bruker Compass ESI POS LOW micro QTOF machine. LRMS was conducted using an Agilent Triple quadropoloe 6410.

1.2 Compound Characterisation

Synthetic procedures for compounds which have not been previously reported by us are presented herein. For compounds which have previously been reported by us and our group no procedure will be given but literature references to our previous work are provided.

1.2.1 3-Ethoxy-4-(phenylamino)cyclobut-3-ene-1,2-dione (9a)

The title compound was obtained as an off-white solid (234 mg, 37%). M. p. 112-115 °C. ^{1}H NMR (400 MHz, DMSO-*d*$_6$) δ 10.73 (s, 1H), 7.36 (d, *J* = 6.4 Hz, 4H), 7.11 (dd, *J* = 11.4 5.5 Hz, 1H), 4.76 (q, *J* = 7.1 Hz, 2H), 1.41 (t, *J* = 7.1 Hz, 3H). ^{13}C NMR (101 MHz, DMSO-*d*$_6$) δ 187.7, 183.6, 178.1, 169.5, 137.9, 128.9, 123.9, 119.5, 69.4, 15.4. HRMS (ESI) m/z: $[M+K]^+$ calcd. for $C_{12}H_{11}NO_3K$ 256.0371; found 256.0405. Synthetic procedure can be found in our previous published work.[2]

1.2.2 3-Ethoxy-4-((4-fluorophenyl)amino)cyclobut-3-ene-1,2-dione (9b)

The title compound was obtained as a pale yellow solid (523 mg, 75%). Decomposition occurred at 250 °C during melting point determination. ^{1}H NMR (400 MHz, DMSO-*d*$_6$) δ 10.77 (s, 1H), 7.37 (s, 2H), 7.22 – 7.17 (m, 2H), 4.75 (q, *J* = 7.1 Hz, 2H), 1.40 (t, *J* = 7.1 Hz, 3H). ^{13}C NMR (101 MHz, DMSO-*d*$_6$) δ 190.6, 188.2, 184.8, 181.8, 159. (d, *J* = 239.9 Hz), 135.3 (d, *J* = 2.5 Hz), 120.8 (d, *J* = 8.2 Hz), 115.7 (d, *J* = 22.8 Hz), 56.1, 18.5.

HRMS (ESI) m/z: $[M+Na]^+$ calcd. for $C_{12}H_{10}FNO_3Na$ 258.0537 found 258.0491. Synthetic procedure can be found in our previous published work.[2]

1.2.3 3-((3-Chlorophenyl)amino)-4-ethoxycyclobut-3-ene-1,2-dione (9c)

The title compound was obtained as a yellow solid (193 mg, 25%). M. p. 152-155 °C. ^1H NMR (400 MHz, DMSO-d_6) δ 10.84 (s, 1H), 7.50 (s, 1H), 7.43 – 7.28 (m, 2H), 7.18 – 7.09 (m, 1H), 4.78 (q, J = 7.1 Hz, 2H), 1.42 (t, J = 7.1 Hz, 3H). ^{13}C NMR (101 MHz, DMSO-d_6) δ 187.4, 183.9, 178.6, 169.3, 139.4, 133.4, 130.5, 123.4, 119.1, 117.8, 69.6, 15.4. HRMS (ESI) m/z: [M]+1 calcd. for $C_{13}H_{13}ClNO_3$ 266.0578; found 266.0577. Synthetic procedure can be found in our previous published work.[2]

1.2.4 3-((4-Chlorophenyl)amino)-4-ethoxycyclobut-3-ene-1,2-dione (9d)

The title compound was obtained as an off-white solid (451 mg, 60%). M. p. 189-193 °C. ^1H NMR (400 MHz, DMSO-d_6) δ 10.80 (s, 1H), 7.40 (d, J = 4.0 Hz, 4H), 4.76 (q, J = 7.1 Hz, 2H), 1.42 (t, J = 7.1 Hz, 3H). ^{13}C NMR (101 MHz, DMSO-d_6) δ 187.5, 183.7, 178.4, 169.3, 136.9, 128.8, 127.9, 121.1, 69.5, 15.4. HRMS (ESI) m/z: [M]+1 calcd. for $C_{12}H_{11}ClNO_3$ 252.0422; found 252.0379. Synthetic procedure can be found in our previous published work.[2]

1.2.5 3-((3-Bromophenyl)amino)-4-ethoxycyclobut-3-ene-1,2-dione (9e)

The title compound was obtained as an off-white solid (553 mg, 64%). M.p. 170-173 °C. ^1H NMR (400 MHz, DMSO-d_6) δ 10.85 (s, 1H), 7.64 (s, 1H), 7.39 – 7.26 (m, 4H), 4.77 (q, J = 7.1 Hz, 2H), 1.42 (t, J = 7.1 Hz, 4H). ^{13}C NMR (101 MHz, DMSO-d_6) δ 187.5, 184.0, 178.6, 169.3, 139.6, 130.9, 126.3, 122.0, 121.8, 118.3, 69.7, 15.5. HRMS (ESI) m/z: $[M+K]^+$ calcd. for $C_{12}H_{10}{}^{81}BrNO_3K$ 335.9455; found 335.9695. Synthetic procedure can be found in our previous published work.[2]

1.2.6 3-((4-Bromophenyl)amino)-4-ethoxycyclobut-3-ene-1,2-dione (9f)

The title compound was obtained as an off-white solid (572 mg, 66%). M. p. 188-191 °C. ^{1}H NMR (400 MHz, DMSO-d_6) δ 10.82 (s, 1H), 7.56 – 7.51 (m, 2H), 7.33 (d, J = 8.2 Hz, 2H), 4.76 (q, J = 7.1 Hz, 2H), 1.41 (t, J = 7.1 Hz, 3H). ^{13}C NMR (101 MHz, DMSO-d_6) δ 187.5, 183.8, 178.4, 169.3, 137.3, 131.7, 121.4, 115.9, 69.6, 15.4. HRMS (ESI) m/z: $[M+K]^+$ calcd. for $C_{12}H_{10}{}^{81}BrNO_3K$ 335.9455; found 335.9726. Synthetic procedure can be found in our previous published work.[2]

1.2.7 3-Ethoxy-4-((2-hydroxyphenyl)amino)cyclobut-3-ene-1,2-dione (9g)

The title compound was obtained as a bright yellow solid (304 mg, 44%). M. p. 215-219 °C. ^{1}H NMR (400 MHz, DMSO-d_6) δ 9.98 (s, 1H), 7.15 (dd, J = 7.8, 1.3 Hz, 1H), 7.04 (ddd, J = 8.0, 7.4, 1.6 Hz, 1H), 6.87 (dd, J = 8.1, 1.3 Hz, 1H), 6.81 – 6.73 (m, 1H), 4.66 (q, J = 7.0 Hz, 2H), 1.34 (t, J = 7.0 Hz, 2H). ^{13}C NMR (101 MHz, DMSO-d_6) δ 188.3, 183.9, 177.8, 170.9, 150.4, 126.7, 124.8, 124.5, 118.8, 115.8, 69.0, 15.6. HRMS (ESI) m/z: $[M+K]^+$ calcd. for $C_{12}H_{11}NO_4K$ 272.0320; found 272.0272. Synthetic procedure can be found in our previous published work.[2]

1.2.8 3-Ethoxy-4-((3-hydroxyphenyl)amino)cyclobut-3-ene-1,2-dione (9h)

The title compound was obtained as a yellow solid (367 mg, 54%). M. p. 207-211 °C. ^{1}H NMR (400 MHz, DMSO-d_6) δ 10.64 (s, 1H), 9.59 (s, 1H), 7.11 (t, J = 8.4 Hz, 1H), 6.82 (s, 2H), 6.51 (dd, J = 7.3, 1.4 Hz, 1H), 4.76 (q, J = 7.1 Hz, 2H), 1.41 (t, J = 7.1 Hz, 3H). ^{13}C NMR (101 MHz, DMSO-d_6) δ 187.7, 183.6, 178.0, 169.4, 157.8, 138.9, 129.6, 111.2, 110.3, 106.7, 69.3, 15.5. HRMS (ESI) m/z: $[M+Na]^+$ calcd. for $C_{12}H_{11}NO_4Na$ 256.0580; found 256.0594. Synthetic procedure can be found in our previous published work.[2]

1.2.9 3-Ethoxy-4-((4-hydroxyphenyl)amino)cyclobut-3-ene-1,2-dione (9i)

The title compound was obtained as a tan solid (311 mg, 45%). M. p. 208-213 °C. ^{1}H NMR (400 MHz, DMSO-d_6) δ 10.56 (s, 1H), 9.42 (s, 1H), 7.14 (s, 2H), 6.75 – 6.69 (m, 2H), 4.72 (q, J = 7.1 Hz, 2H), 1.44 – 1.32 (m, 3H). ^{13}C NMR (101 MHz, Acetone-d_6) δ 155.6, 131.2, 122.5, 116.5, 70.3, 16.1. HRMS (ESI) m/z: $[M+Na]^+$ calcd. for $C_{12}H_{11}NO_4Na$ 256.0580; found 256.0603. Synthetic procedure can be found in our previous published work.[2]

1.2.10 3-ethoxy-4-((2-methoxyphenyl)amino)cyclobut-3-ene-1,2-dione (9j)

The title compound was obtained as a white solid (133 mg, 18%). M. p. 132-136 °C. ^{1}H NMR (600 MHz, DMSO-d_6) δ 10.21 (s, 1H), 7.20 (dd, J = 8.3, 6.7 Hz, 2H), 7.08 – 7.05 (m, 1H), 6.93 (td, J = 7.6, 1.3 Hz, 1H), 4.66 (q, J = 7.1 Hz, 2H), 3.79 (s, 3H), 1.34 (t, J = 7.1 Hz, 3H). ^{13}C NMR (151 MHz, DMSO-d_6) δ 188.2, 184.1, 178.1, 171.0, 152.2, 127.0, 125.8, 124.3, 120.2, 111.8, 69.1, 55.6, 15.7. HRMS (ESI) m/z: [M]+1 calcd. For $C_{13}H_{14}NO_4$ 248.0917; found 248.0881. Synthetic procedure can be found in our previous published work.[2]

1.2.11 3-Ethoxy-4-((3-methoxyphenyl)amino)cyclobut-3-ene-1,2-dione (9k)

The title compound was obtained as a pale-yellow solid (684 mg 94%). M. p. 167-170 °C. ^{1}H NMR (400 MHz, DMSO-d_6) δ 10.73 (s, 1H), 7.24 (t, J = 8.2 Hz, 1H), 7.05 (s, 1H), 6.94 (d, J = 8.0 Hz, 1H), 6.67 (ddd, J = 8.3, 2.4, 0.9 Hz, 1H), 4.77 (q, J = 7.1 Hz, 2H), 3.75 (s, 3H), 1.41 (t, J = 7.1 Hz, 3H). ^{13}C NMR (101 MHz, DMSO-d_6) δ 159.9, 139.3, 130.0, 111.7, 109.8, 105.2, 69.7, 55.2, 15.7. HRMS (ESI) m/z: [M]+1 calcd. for $C_{13}H_{14}NO_4$ 248.0917; found 248.0764. Synthetic procedure can be found in our previous published work.[2]

1.2.12 3-Ethoxy-4-((4-methoxyphenyl)amino)cyclobut-3-ene-1,2-dione (9l)

The title compound was obtained as a pale-yellow solid (359 mg, 49%). M. p. 175-177 °C. ^{1}H NMR (400 MHz, DMSO-d_6) δ 10.62 (s, 1H), 7.27 (s, 2H), 6.96 – 6.89 (m, 2H), 4.74 (q, J = 7.0 Hz, 2H), 3.73 (s, 3H), 1.40 (t, J = 7.0 Hz, 3H). ^{13}C NMR (101 MHz, DMSO-d_6) δ 156.2, 131.0, 121.3, 114.2, 69.4, 55.3, 15.7. HRMS (ESI) m/z: [M]+1 calcd. for $C_{13}H_{14}NO_4$ 248.0917; found 248.0931. Synthetic procedure can be found in our previous published work.[2]

1.2.13 3-Ethoxy-4-((3-(trifluoromethyl)phenyl)amino)cyclobut-3-ene-1,2-dione (9m)

The title compound was obtained as a bright yellow solid (450 mg, 54%). M. p. 160-164 °C. ^{1}H NMR (400 MHz, DMSO) δ 10.98 (s, 1H), 7.78 (s, 1H), 7.66 – 7.54 (m, 2H), 7.44 (dt, J = 7.2, 1.7 Hz, 1H), 4.78 (q, J = 7.1 Hz, 2H), 1.41 (t, J = 7.1 Hz, 3H). ^{13}C NMR (151 MHz, DMSO) δ 138.9, 130.4, 129.9, 124.9, 123.1, 120.2, 115.9, 69.9, 15.5. HRMS (ESI) m/z: [M]+1 calcd. for $C_{13}H_{11}F_3NO_3$ 286.0686; found 286.0809. Synthetic procedure can be found in our previous published work.[2]

1.2.14 3-Ethoxy-4-((4-(trifluoromethyl)phenyl)amino)cyclobut-3-ene-1,2-dione (9n)

The title compound was obtained as a bright yellow solid (404 mg, 48%). M. p. 181-184 °C. ^{1}H NMR (400 MHz, DMSO-d_6) δ 11.00 (s, 1H), 7.72 (d, J = 8.5 Hz, 2H), 7.57 (d, J = 8.4 Hz, 2H), 4.79 (q, J = 7.1 Hz, 2H), 1.43 (t, J = 7.1 Hz, 3H). ^{13}C NMR (151 MHz, DMSO) δ 187.4, 184.4, 179.3, 169.6, 141.7, 126.5, 125.3, 123.9, 123.7, 123.5, 119.4, 70.0, 15.6. HRMS (ESI) m/z: [M]+1 calcd. for $C_{13}H_{11}F_3NO_3$ 286.0686; found 286.0683. Synthetic procedure can be found in our previous published work.[2]

1.2.15 3-Ethoxy-4-(o-tolylamino)cyclobut-3-ene-1,2-dione (9o)

The title compound was obtained as a yellow solid (197 mg, 29%). M. p. 113-117 °C. ^{1}H NMR (400 MHz, DMSO-d_6) δ 10.31 (s, 1H), 7.24 – 7.10 (m, 4H), 4.68 (q, J = 7.1 Hz, 2H), 2.27 (s, 3H), 1.34 (t, J = 7.0 Hz, 3H). ^{13}C NMR (101 MHz, DMSO-d_6) δ 188.3, 183.9, 177.8, 170.8, 135.6, 131.3, 130.3, 125.0, 125.9, 124.3, 69.0, 17.3, 15.4. HRMS (ESI) m/z: $[M+Na]^+$ calcd. for $C_{13}H_{13}NO_3Na$ 254.0788; found 254.0827. Synthetic procedure can be found in our previous published work.[2]

1.2.16 3-Ethoxy-4-(m-tolylamino)cyclobut-3-ene-1,2-dione (9q)

The title compound was obtained as a bright yellow solid (607 mg, 89%). M. p. 143-145 °C. ^{1}H NMR (600 MHz, DMSO-d_6) δ 10.70 (s, 1H), 7.29 – 7.13 (m, 3H), 6.93 (d, J = 7.4 Hz, 1H), 4.76 (q, J = 7.1 Hz, 2H), 2.28 (s, 3H), 1.41 (t, J = 7.1 Hz, 3H). ^{13}C NMR (151 MHz, DMSO-d_6) δ 138.5, 137.9, 128.9, 124.8, 120.2, 116.9, 69.5, 21.1, 15.6. HRMS (ESI) m/z: $[M+Na]^+$ calcd. for $C_{13}H_{13}NO_3Na$ 254.0788; found 254.0783. Synthetic procedure can be found in our previous published work.[2]

1.2.17 3-Ethoxy-4-(p-tolylamino)cyclobut-3-ene-1,2-dione (9q)

The title compound was obtained as a yellow solid (546 mg, 75%). M. p. 168-171 °C. ^{1}H NMR (400 MHz, DMSO-d_6) δ 10.66 (s, 1H), 7.31 – 7.07 (m, 4H), 4.75 (q, J = 7.1 Hz, 2H), 2.26 (s, 3H), 1.41 (t, J = 7.0 Hz, 3H). ^{13}C NMR (101 MHz, DMSO-d_6) δ 187.8, 183.4, 177.8, 169.4, 135.3, 133.1, 129.2, 119.6, 69.2, 20.2, 15.4. HRMS (ESI) m/z: $[M+Na]^+$ calcd. for $C_{13}H_{13}NO_3Na$ 254.0788; found 254.0787. Synthetic procedure can be found in our previous published work.[2]

1.2.18 ((2-Ethoxy-3,4-dioxocyclobut-1-en-1-yl)amino)benzoic acid (9r)

The title compound was obtained as a white solid (243 mg 31%). M. p. 256-259 °C. ^{1}H NMR (400 MHz, DMSO-d_6) δ 12.96 (s, 1H), 10.83 (s, 1H), 7.99 (t, J = 2.0 Hz, 1H), 7.67 (dt, J = 7.6, 1.3 Hz, 1H), 7.64 – 7.57 (m, 1H), 7.47 (t, J = 7.9 Hz, 1H), 4.78 (q, J = 7.1 Hz, 2H), 1.43 (t, J = 7.1 Hz, 3H). ^{13}C NMR (101 MHz, DMSO-d_6) δ 187.8, 184.1, 178.4, 169.5, 166.8, 138.2, 131.8, 129.3, 124.7, 123.7, 120.3, 69.6, 15.4. HRMS (ESI) m/z: $[M+Na]^+$ calcd. for $C_{13}H_{11}NO_5Na$ 284.0529; found 284.0389. Synthetic procedure can be found in our previous published work.[2]

1.2.19 3-((4-Bromophenyl)(methyl)amino)-4-ethoxycyclobut-3-ene-1,2-dione (9s)

The title compound was obtained as a white solid (90 mg 29%). M. p. 164-168 °C. ^{1}H NMR (400 MHz, DMSO-d_6) δ 7.64 – 7.55 (m, 2H), 7.38 – 7.15 (m, 2H), 4.68 (q, J = 7.1 Hz, 2H), 3.61 (s, 3H), 1.34 (t, J = 7.1 Hz, 3H). ^{13}C NMR (101 MHz, DMSO-d_6) δ 187.8, 186.6, 183.9, 177.7, 141.8, 131.5, 125.2, 122.9, 19.5, 38.7, 15.5. HRMS (ESI) m/z: [M]+1 calcd. for $C_{13}H_{13}{}^{79}BrNO_3$ 310.0073; found 309.9945. Synthetic procedure can be found in our previous published work.[2]

1.2.20 3-(Benzylamino)-4-ethoxycyclobut-3-ene-1,2-dione (10a)

The title compound was obtained as an off-white solid (223 mg, 33%). M. p. 96-104 °C. ^{1}H NMR (400 MHz, DMSO-d_6) δ 9.19 (d, J = 63.2 Hz, 1H), 7.43 – 7.24 (m, 5H), 4.72 – 4.60 (m, 3H), 4.47 (s, 1H), 1.35 (dt, J = 14.0, 7.1 Hz, 3H).. ^{13}C NMR (101 MHz, DMSO) δ 189.5, 189.2, 182.4, 182.1, 177.4, 176.7, 172.7, 172.0, 138.5, 138.1, 128.7, 127.5, 127.5, 127.5, 69.0, 68.9, 47.4, 46.9, 15.6. HRMS (ESI) m/z: $[M+K]^+$ calcd. for $C_{13}H_{13}NO_3K$ 270.0527; found 270.0575. Synthetic procedure can be found in our previous published work.[2]

1.2.21 3-((2-Chlorobenzyl)amino)-4-ethoxycyclobut-3-ene-1,2-dione (10b)

The title compound was obtained as an off-white solid (298 mg, 38%). M. p. 152-155 °C. ^{1}H NMR (400 MHz, DMSO-d_6) δ 9.15 (d, J = 87.1 Hz, 1H), 7.51 – 7.32 (m, 4H), 4.85 – 4.57 (m, 4H), 1.42 – 1.25 (m, 3H). ^{13}C NMR (101 MHz, Acetone-d_6) δ 136.4, 133.9, 130.6, 130.5, 130.3, 128.3, 69.9, 46.4, 16.0. HRMS (ESI) m/z: [M]+1 calcd. for $C_{13}H_{13}ClNO_3$ 266.0578; found 266.0578. Synthetic procedure can be found in our previous published work.[2]

1.2.22 3-((3-Chlorobenzyl)amino)-4-ethoxycyclobut-3-ene-1,2-dione (10c)

The title compound was obtained as a yellow solid (193 mg, 25%). M. p. 152-155 °C. ^{1}H NMR (400 MHz, DMSO-d_6) δ 9.15 (d, J = 82.8 Hz, 1H), 7.44 – 7.21 (m, 4H), 4.71 – 4.43 (m, 4H), 1.40 – 1.29 (m, 3H). ^{13}C NMR (101 MHz, DMSO-d_6) δ 141.1, 133.1, 130.3, 127.3, 127.2, 126.0, 68.8, 46.2, 15.4. HRMS (ESI) m/z: [M]+1 calcd. for $C_{13}H_{13}ClNO_3$ 266.0578; found 266.0577. Synthetic procedure can be found in our previous published work.[2]

1.2.23 3-((4-Chlorobenzyl)amino)-4-ethoxycyclobut-3-ene-1,2-dione (10d)

The title compound was obtained as a pale yellow solid (778 mg, 99 %). M. p. 132-136 °C. ^{1}H NMR (400 MHz, DMSO-d_6) δ 9.17 (d, J = 84.5 Hz, 1H), 7.38 (dd, J = 44.3, 7.9 Hz, 4H), 4.71 – 4.41 (m, 4H), 1.34 (dt, J = 15.8, 7.0 Hz, 3H). ^{13}C NMR (101 MHz, DMSO-d_6) δ 189.1, 182.2, 176.8, 172.7, 137.2, 132.2, 129.4, 128.6, 69.0, 46.6, 15.6. HRMS (ESI) m/z: [M]+1 calcd. for $C_{13}H_{13}ClNO_3$ 266.0578; found 256.0557. Synthetic procedure can be found in our previous published work.[2]

1.2.24 3-((2-Bromobenzyl)amino)-4-ethoxycyclobut-3-ene-1,2-dione (10e)

The title compound was obtained as a pale-yellow solid (308 mg, 34%). M. p. 153-155 °C. ^{1}H NMR (400 MHz, DMSO-d_6) δ 9.13 (s, 1H), 7.66 – 7.63 (m, 1H), 7.44 – 7.39 (m,

2H), 7.27 (ddd, *J* = 7.9, 6.5, 2.5 Hz, 1H), 4.79 – 4.56 (m, 4H), 1.33 (dt, *J* = 39.8, 7.1 Hz, 3H). ^{13}C NMR (151 MHz, DMSO-d_6) δ 188.9, 182.8, 177.1, 173.2, 136.5, 132.7, 129.7, 129.5, 128.1, 122.4, 69.0, 47.6, 47.1, 15.7. HRMS (ESI) m/z: [M]+1 calcd. for $C_{13}H_{13}{}^{79}BrNO_3$ 310.0073; found 310.0088. Synthetic procedure can be found in our previous published work.[2]

1.2.25 3-((3-Bromobenzyl)amino)-4-ethoxycyclobut-3-ene-1,2-dione (10f)

The title compound was obtained as a yellow solid (516 mg, 57%). M. p. 110-114 °C. ^{1}H NMR (400 MHz, DMSO-d_6) δ 9.16 (d, *J* = 85.4 Hz, 1H), 7.50 (dd, *J* = 9.2, 1.7 Hz, 2H), 7.32 (p, *J* = 7.8 Hz, 2H), 4.70 – 4.44 (m, 4H), 1.41 – 1.27 (m, 3H). ^{13}C NMR (101 MHz, Acetone-d_6) δ 142.1, 131.7, 131.6, 127.6, 127.5, 123.1, 70.0, 47.9, 16.1. HRMS (ESI) m/z: [M]+1 calcd. for $C_{13}H_{12}{}^{79}BrNO_3$ 310.0073; found 309.9859. Synthetic procedure can be found in our previous published work.[2]

1.2.26 3-((4-Bromobenzyl)amino)-4-ethoxycyclobut-3-ene-1,2-dione (10g)

The title compound was obtained as a white solid (256 mg, 41%). M. p. 202-205 °C. ^{1}H NMR (400 MHz, DMSO-d_6) δ 9.15 (d, *J* = 84.3 Hz, 1H), 7.56 (dd, *J* = 8.5, 2.2 Hz, 2H), 7.26 (d, *J* = 8.1 Hz, 2H), 4.54 (dd, *J* = 80.5, 5.0 Hz, 4H), 1.43 – 1.28 (m, 3H). ^{13}C NMR (101 MHz, DMSO-d_6) δ 138.3, 131.3, 129.5, 120.4, 68.8, 46.0, 15.4. HRMS (ESI) m/z: $[M+K]^+$ calcd. for $C_{13}H_{12}{}^{81}BrNO_3K$ 349.9632; found 349.9765. Synthetic procedure can be found in our previous published work.[2]

1.2.27 3-((4-Bromobenzyl)(methyl)amino)-4-ethoxycyclobut-3-ene-1,2-dione (10h)

The title compound was obtained as a white solid (870 mg, 91%). M. p. 110-114 °C. ^{1}H NMR (400 MHz, DMSO-d_6) δ 7.59 (d, *J* = 8.4 Hz, 2H), 7.28 (d, *J* = 8.4 Hz, 2H), 4.75 (s,

1H), 4.67 (p, J = 6.8 Hz, 2H), 4.52 (s, 1H), 3.05 (d, J = 63.5 Hz, 3H), 1.35 (q, J = 7.3 Hz, 3H). ^{13}C NMR (151 MHz, DMSO-d_6) δ 188.8, 181.6, 176.5, 171.4, 134.9, 131.7, 130.3, 130.2, 121.2, 69.2, 53.0, 36.0, 15.5. HRMS (ESI) m/z: [M]+1 calcd. for $C_{14}H_{15}{}^{81}BrNO_3$ 326.02298; found 326.0203. Synthetic procedure can be found in our previous published work.[2]

1.2.28 3-(Diisopropylamino)-4-ethoxycyclobut-3-ene-1,2-dione (11)

To 3,4-diethoxycyclobut-3-ene-1,2-dione (750 mg, 0.65 mL, 4.41 mmol, 1.0 Equiv.) dissolved in EtOH (10 mL) was added DIPA (937 mg, 1.30 mL, 9.26 mmol, 2.1 Equiv.). The reaction was refluxed for a period of 24 h before being concentrated under reduced pressure and purified via column chromatography (40% EtOAc : Hexane). The title compound was obtained as an off white solid following trituration with hexane (595 mg, 60%). M. p. 187-190 °C. ^{1}H NMR (600 MHz, Chloroform-d) δ 4.83 (q, J = 7.1 Hz, 2H), 4.64 (s, 1H), 3.95 (d, J = 7.3 Hz, 1H), 1.46 (t, J = 7.1 Hz, 3H), 1.30 (t, J = 7.2 Hz, 12H). ^{13}C NMR (101 MHz, Chloroform-d) δ 189.0, 182.6, 175.7, 171.3, 69.5, 49.9, 48.8, 22.0, 21.8, 16.0. LRMS (ESI) m/z: $[M+Na]^+$ calcd. for $C_{12}H_{19}NO_3Na$ 248.1; found 248.0. Spectroscopic data was in accordance with literature data.[3]

1.2.29 3-(Azetidin-1-yl)-4-ethoxycyclobut-3-ene-1,2-dione (12a)

The title compound was obtained as a white solid (215 mg, 40%). M. p. 77-79 °C. ^{1}H NMR (400 MHz, DMSO-d_6) δ 4.56 (q, J = 7.1 Hz, 2H), 4.38 (s, 4H), 2.48 – 2.40 (m, 2H), 1.34 (t, J = 7.0 Hz, 3H). ^{13}C NMR (101 MHz, Acetone-d_6) δ 189.9, 183.4, 178.2, 172.4, 69.9, 55.3, 19.4, 16.3. HRMS (ESI) m/z: $[M+Na]^+$ calcd. for $C_9H_{11}NO_3Na$ 204.0631; found 204.0648. Synthetic procedure can be found in our previous published work.[4]

1.2.30 3-Ethoxy-4-(pyrrolidin-1-yl)cyclobut-3-ene-1,2-dione (12b)

The title compound was obtained as a white solid (57 mg, 10%). M. p. 108-111 °C. ^{1}H NMR (400 MHz, DMSO-d_6) δ 4.63 (q, J = 7.0 Hz, 2H), 3.61 (m, 4H), 1.89 – 1.83 (m, 4H), 1.36 (t, J = 7.1 Hz, 3H). ^{13}C NMR (101 MHz, DMSO-d_6) δ 188.8, 181.3, 176.4, 169.7, 68.5, 49.8, 48.1, 24.7, 24.4, 15.4. HRMS (ESI) m/z: $[M+K]^+$ calcd. for $C_{10}H_{13}NO_3K$ 234.0527; found 234.0547. Synthetic procedure can be found in our previous published work.[4]

1.2.31 3-Ethoxy-4-(piperidin-1-yl)cyclobut-3-ene-1,2-dione (12c)

The title compound was obtained as an off-white solid (334 mg, 54%). M. p. 73-75 °C. ^{1}H NMR (600 MHz, DMSO-d_6) δ 4.66 (q, J = 7.1 Hz, 2H), 3.75 (s, 2H), 3.50 (s, 2H), 1.60 (s, 6H), 1.37 (t, J = 7.1 Hz, 3H). ^{13}C NMR (101 MHz, DMSO-d_6) δ 188.8, 181.1, 175.5, 169.6, 68.9, 46.9, 25.4, 22.7, 15.4. HRMS (ESI) m/z: [M]+1 calcd. for $C_{11}H_{16}NO_3$ 210.1125; found 210.1115. Synthetic procedure can be found in our previous published work.[4]

1.2.32 3-(Azepan-1-yl)-4-ethoxycyclobut-3-ene-1,2-dione (12d)

The title compound was obtained as an off-white solid (139 mg, 21%). M. p. 69-72 °C. ^{1}H NMR (400 MHz, Chloroform-d) δ 4.76 (q, J = 7.1 Hz, 2H), 3.89 – 3.82 (m, 2H), 1.84 – 1.71 (m, 4H), 1.65 – 1.58 (m, 4H), 1.44 (t, J = 7.1 Hz, 3H). ^{13}C NMR (101 MHz, Chloroform-d) δ 189.2, 182.5, 176.6, 172.1, 69.5, 50.6, 50.0, 29.2, 28.5, 27.2, 26.7, 15.9. LRMS (ESI) m/z: $[M+Na]^+$ calcd. for $C_{12}H_{17}NO_3Na$ 246.1; found 246.1. Synthetic procedure can be found in our previous published work.[4]

1.2.33 3-Ethoxy-4-morpholinocyclobut-3-ene-1,2-dione (12e)

The title was obtained as a white solid (449 mg, 72%). M. p. 177-180 °C. ^{1}H NMR (400 MHz, DMSO-d_6) δ 4.66 (q, J = 7.1 Hz, 2H), 3.82 – 3.67 (m, 6H), 3.53 (s, 2H), 1.37 (t, J

= 7.1 Hz, 3H). ^{13}C NMR (101 MHz, DMSO-d_6) δ 188.5, 181.5, 175.9, 169.7, 69.1, 65.6, 15.4. HRMS (ESI) m/z: $[M+Na]^+$ calcd. for $C_{10}H_{13}NO_4$ 234.0737; found 234.0580. Synthetic procedure can be found in our previous published work.[4]

1.2.34 3-((2R,6S)-2,6-Dimethylmorpholino)-4-ethoxycyclobut-3-ene-1,2-dione (12f)

The title compound was obtained as a white solid (532 mg 76%). M. p. 139-142 °C. ^{1}H NMR (400 MHz, Chloroform-d) δ 4.77 (p, J = 7.0 Hz, 2H), 4.48 (d, J = 13.1 Hz, 1H), 3.80 (d, J = 12.9 Hz, 1H), 3.76 – 3.62 (m, 2H), 2.85 (ddd, J = 16.0, 13.0, 10.6 Hz, 2H), 1.46 (t, J = 7.1 Hz, 3H), 1.21 (t, J = 5.4 Hz, 6H). ^{13}C NMR (101 MHz, DMSO-d_6) δ 188.5, 181.5, 175.8, 169.5, 70.7, 69.1, 51.2, 50.7, 18.1, 15.4. HRMS (ESI) m/z: [M]+1 calcd. for $C_{12}H_{18}NO_4$ 240.1236; found 240.1561. Synthetic procedure can be found in our previous published work.[4]

1.2.35 3-Ethoxy-4-thiomorpholinocyclobut-3-ene-1,2-dione (12g)

The title was obtained as a white solid (638 mg, 95%). M. p. 176-179 °C. ^{1}H NMR (400 MHz, Chloroform-d) δ 4.77 (q, J = 7.1 Hz, 2H), 4.20 – 4.11 (m, 2H), 3.90 – 3.77 (m, 2H), 2.77 – 2.69 (m, 4H), 1.46 (t, J = 7.1 Hz, 3H). ^{13}C NMR (101 MHz, DMSO-d_6) δ 188.5, 181.6, 175.9, 170.2, 69.1, 48.9, 26.4, 15.4. HRMS (ESI) m/z: $[M+Na]^+$ calcd. for $C_{10}H_{13}NO_3SNa$ 250.0508; found 250.0499. Synthetic procedure can be found in our previous published work.[4]

1.2.36 3-(1,1-Dioxidothiomorpholino)-4-ethoxycyclobut-3-ene-1,2-dione (12h)

The title compound was obtained as a white solid (208 mg, 27%). M. p. 189-193 °C. ^{1}H NMR (400 MHz, Chloroform-d) δ 4.79 (q, J = 7.1 Hz, 2H), 4.40 (s, 2H), 4.09 (s, 2H), 3.20 (t, J = 5.6 Hz, 4H), 1.48 (t, J = 7.1 Hz, 3H). ^{13}C NMR (101 MHz, DMSO-d_6) δ 188.2,

182.3, 176.6, 170.9, 69.4, 50.6, 45.4, 15.5. HRMS (ESI) m/z: [M]+1 calcd. for $C_{10}H_{14}NO_5S$ 260.0587; found 260.0449. Synthetic procedure can be found in our previous published work.[4]

1.2.37 3-Ethoxy-4-(thiazolidin-3-yl)cyclobut-3-ene-1,2-dione (12i)

The title compound was obtained as a yellow solid (180 mg, 21%). M. p. 94-96 °C. ^{1}H NMR (400 MHz, Chloroform-*d*) δ 4.89 (s, 1H), 4.77 (q, J = 7.1 Hz, 2H), 4.65 (s, 1H), 4.15 (s, 1H), 3.90 (s, 1H), 3.07 (t, J = 6.4 Hz, 2H), 1.46 (t, J = 7.1 Hz, 3H). ^{13}C NMR (101 MHz, DMSO-d_6) δ 179.6, 175.1, 168.7, 162.2, 61.6, 42.5, 41.9, 21.2, 6.5. LRMS (ESI) m/z: $[M+Na]^+$ calcd. for $C_9H_{11}NO_3SNa$ 236.0; found 236.1. Synthetic procedure can be found in our previous published work.[4]

1.2.38 Tert-butyl 4-(2-ethoxy-3,4-dioxocyclobut-1-en-1-yl)piperazine-1-carboxylate (12j)

The title compound was obtained as a white solid (685 mg, 73%). M. p. 131-134 °C. ^{1}H NMR (400 MHz, Chloroform-*d*) δ 4.77 (q, J = 7.1 Hz, 2H), 3.86 (s, 2H), 3.55 (s, 6H), 1.46 (t, J = 5.5 Hz, 12H). ^{13}C NMR (101 MHz, DMSO-d_6) δ 188.5, 181.6, 176.0, 170.0, 153.5, 79.3, 69.1, 46.0, 42.8, 27.8, 15.4. HRMS (ESI) m/z: $[M+K]^+$ calcd. for $C_{15}H_{22}N_2O_5K$ 349.1166; found 349.1518. Synthetic procedure can be found in our previous published work.[4]

1.2.39 3-(7-Bromo-3,4-dihydroquinolin-1(2H)-yl)-4-ethoxycyclobut-3-ene-1,2-dione (12k)

The title compound was obtained as a white solid (144 mg 21%). M. p. 187-190 °C. ^{1}H NMR (400 MHz, Methylene Chloride-d_2) δ 7.35 (dd, J = 8.1, 2.1 Hz, 1H), 7.29 (d, J = 9.9

Hz, 1H), 7.07 (d, J = 7.8 Hz, 1H), 5.02 (s, 1H), 4.75 (q, J = 6.5 Hz, 3H), 4.14 – 3.78 (m, 2H), 2.93 (t, J = 6.1 Hz, 2H), 1.46 (t, J = 7.1 Hz, 3H). ^{13}C NMR (101 MHz, Acetone-*d6*) δ 183.2, 171.8, 133.8, 132.0, 130.7, 129.8, 120.4, 70.1, 48.5, 30.1, 16.1, 14.5. LRMS (ESI) m/z: $[M+Na]^+$ calcd. for $C_{15}H_{14}{}^{81}BrNO_3$ 360.0; found 360.0. Synthetic procedure can be found in our previous published work.[4]

1.2.40 3,4-Bis(phenylamino)cyclobut-3-ene-1,2-dione (13a)

To 3,4-diethoxycyclobut-3-ene-1,2-dione (1.50 g, 1.3 mL, 8.82 mmol, 1.0 Equiv.) and NEt_3 (3.57 g, 4.9 mL 35.3 mmol, 4.0 Equiv.) dissolved in EtOH (10 mL) was added aniline (1.81 g, 1.77 mL, 19.40 mmol, 2.2 Equiv.). The reaction was then heated at 80 °C for 12 h. The reaction was then cooled to r.t., filtered and the precipitate washed with cold EtOH. The title compound was obtained as a white solid (2.04 g, 88%). Decomposition occurred at 265 °C during melting point determination. ^{1}H NMR (600 MHz, DMSO-*d6*) δ 9.90 (s, 2H), 7.50 (d, J = 7.9 Hz, 4H), 7.39 (t, J = 7.7 Hz, 4H), 7.09 (t, J = 7.4 Hz, 2H). ^{13}C NMR (151 MHz, DMSO) δ 181.5, 165.6, 138.4, 129.2, 123.1, 118.4. LRMS (ESI) m/z: $[M+K]^+$ calcd. for $C_{16}H_{12}N_2O_2K$ 303.1; found 304.1. Spectroscopic data was in accordance with literature data.[3]

1.2.41 3,4-Bis(benzylamino)cyclobut-3-ene-1,2-dione (13b)

To 3,4-diethoxycyclobut-3-ene-1,2-dione (1.5 g, 1.3 mL, 8.82 mmol, 1.0 Equiv.) and $Zn(OTf)_2$ (641 mg, 1.76 mmol, 0.2 Equiv.) dissolved in toluene/DMF (19:1) (26 mL) was added benzylamine (1.98 g, 2.0 mL, 18.51 mmol, 2.1 Equiv.). The reaction was then heated at 75 °C for 36 h. The reaction was then cooled to r.t., filtered and the precipitate washed with Et_2O. The title compound was obtained as a white solid (2.39 g, 92%). M. p. 203-206 °C**.** ^{1}H NMR (600 MHz, DMSO-d_6) δ 7.71 (s, 2H), 7.41 – 7.26 (m, 10H), 4.71 (s, 4H). ^{13}C

NMR (151 MHz, DMSO-d_6) δ 182.5, 167.4, 138.6, 128.3, 127.1, 127.0, 46.6. LRMS (ESI) m/z: $[M+Na]^+$ calcd. for $C_{18}H_{16}N_2O_2Na$ 315.1; found 315.0. Spectroscopic data was in accordance with literature data.[5]

1.2.42 3,4-Bis((4-bromophenyl)amino)cyclobut-3-ene-1,2-dione (13c)

To 3,4-diethoxycyclobut-3-ene-1,2-dione (1.3 g, 1.2 mL, 7.8 mmol, 1.0 Equiv.) and $Zn(OTf)_2$ (567 mg, 1.44 mmol, 0.2 Equiv.) suspended in PhMe (20 mL) was added 4-bromoaniline (2.82 g, 16.4 mmol, 2.1 Equiv.). The reaction was then heated to reflux for a period of 24 h. After allowing the reaction to cool to r.t., the precipitate was filtered and washed with ice cold PhMe. The title compound was obtained as an off-white solid (2.76 g, 84%). M. p. 318-321 °C. ^{1}H NMR (600 MHz, DMSO-d_6) δ 9.95 (s, 2H), 7.55 (d, J = 8.8 Hz, 4H), 7.42 (d, J = 8.8 Hz, 4H). ^{13}C NMR (151 MHz, DMSO-d_6) δ 181.8, 165.5, 137.8, 132.0, 120.6, 115.2. LRMS (ESI) m/z: [M-2Br+K]+1 calcd. for $C_{16}H_{13}N_2O_2K$ 304.1; found 304.2. Spectroscopic data was in accordance with literature data.[6]

1.2.43 3,4-Bis(methyl(phenyl)amino)cyclobut-3-ene-1,2-dione (14a)

To 3,4-bis(phenylamino)cyclobut-3-ene-1,2-dione (**13a**) (1.0 g, 3.78 mmol, 1.0 Equiv.) dissolved in dry DMF (15 mL) was added KOtBu (1.27 g, 11.4 mmol, 3.0 Equiv.) and the reaction was allowed to stir at r.t., for 1 h upon addition the reaction turned fluorescent green. Following this MeI (1.62 g, 0.71 mL, 11.4 mmol, 3.0 Equiv.) was added dropwise to the reaction and stirred at 80 °C for 18 h. After this period, H_2O (30 mL) was added, and the resulting precipitate was filtered and washed with ice cold water (3 x 35 mL). The title compound was obtained as a light tan solid (783 mg, 71%). M. p. 177-180 °C. ^{1}H NMR (600 MHz, Methylene Chloride-d_2) δ 7.01 (t, J = 7.8 Hz, 4H), 6.88 (t, J = 7.4 Hz, 2H), 6.64 (d, J = 7.8 Hz, 4H), 3.62 (s, 6H). ^{13}C NMR (151 MHz, CD_2Cl_2) δ 187.2, 168.1, 143.3,

129.1, 125.2, 121.5, 39.2. LRMS (ESI) m/z: [M]+1 calcd. for $C_{18}H_{17}N_2O_2$ 293.1; found 293.0. Spectroscopic data was in accordance with literature data.[3]

1.2.44 3,4-Bis(benzyl(methyl)amino)cyclobut-3-ene-1,2-dione (14b)

To 3,4-bis(benzylamino)cyclobut-3-ene-1,2-dione (**13b**) (300 mg, 1.03 mmol, 1.0 Equiv.) dissolved in dry DMF (5 mL) was added KO^tBu (345 mg, 3.08 mmol, 3.0 Equiv.) and the reaction was allowed to stir at r.t., for 1 h upon addition the reaction turned fluorescent green. Following this MeI (437 mg, 0.20 mL, 3.08 mmol, 3.0 Equiv.) was added dropwise to the reaction and stirred at r.t., for 12 h. After this period, H_2O (15 mL) was added and the resulting precipitate was filtered and washed with ice cold water (3 x 12 mL). The title compound was obtained as a bright yellow solid following triturating the product with Et_2O (189 mg, 65%). M. p. 131-133 °C. ^1H NMR (400 MHz, Methylene Chloride-d_2) δ 7.39 – 7.34 (m, 4H), 7.34 – 7.30 (m, 2H), 7.30 – 7.25 (m, 4H), 4.86 (s, 4H), 3.01 (s, 6H). ^{13}C NMR (151 MHz, Methylene Chloride-d_2) δ 184.4, 169.3, 136.4, 129.2, 128.3, 56.5, 40.5, 39.8. LRMS (ESI) m/z: $[M+Na]^+$ calcd. for $C_{20}H_{20}N_2O_2Na$ 343.1; found 343.2.

1.2.45 3,4-Bis((4-bromophenyl)(methyl)amino)cyclobut-3-ene-1,2-dione (14c)

To 3,4-bis((4-bromophenyl)amino)cyclobut-3-ene-1,2-dione (**13c**) (750 mg, 1.79 mmol, 1.0 Equiv.) dissolved in dry DMF (12 mL) was added KO^tBu (602 mg, 5.37 mmol, 3.0 Equiv.) and the reaction was allowed to stir at r.t., for 1 h upon addition the reaction turned dark red. Following this MeI (762 mg, 0.33 mL, 3.08 mmol, 3.00 Equiv.) was added dropwise to the reaction and stirred at 50 °C for 6 h. After this period, H_2O (15 mL) was added and the resulting precipitate was filtered and washed with ice cold water (3 x 12 mL). The crude product was then purified via column chromatography (40% EtOAc : Hexane) to afford the title compound as a beige solid (300 mg, 37%). M. p. 260-264 °C. ^1H NMR (600

MHz, DMSO-d_6) δ 7.19 (d, J = 8.8 Hz, 4H), 6.79 (d, J = 8.8 Hz, 4H), 3.55 (s, 6H). ^{13}C NMR (151 MHz, DMSO-d_6) δ 186.5, 167.1, 141.8, 131.0, 122.8, 116.8, 37.9. LRMS (ESI) m/z: [M-2Br-2CH$_3$+K]+1 calcd. for $C_{16}H_{13}N_2O_2K$ 304.06; found 304.2.

1.2.46 3,4-Bis(benzyl(4-bromophenyl)amino)cyclobut-3-ene-1,2-dione (15)

To 3,4-bis((4-bromophenyl)amino)cyclobut-3-ene-1,2-dione (**13c**) (600 mg, 1.43 mmol, 1.0 Equiv.) dissolved in dry DMF (9 mL) was added KO^{t}Bu (481 mg, 4.30 mmol, 3.0 Equiv.) and the reaction was allowed to stir at r.t., for 1 h upon addition the reaction turned dark green. Following this BnBr (735 mg, 0.50 mL, 4.30 mmol, 3.0 Equiv.) was added dropwise to the reaction and stirred at r.t., for 24 h. After this period H_2O (50 mL) was added and the product was extracted with DCM (3 x 30 mL), washed with water and brine (4 x 35 mL), dried over anhydrous $MgSO_4$ and concentrated under reduced pressure. The crude product was then purified via column chromatography (30% EtOAc : Hexane) and triturated with Et_2O to afford the title compound as a pale orange solid (90 mg, 10%). M. p. 235-237 °C. ^{1}H NMR (400 MHz, DMSO-d_6) δ 7.3 – 7.1 (m, 10H), 7.0 – 6.9 (m, 4H), 6.8 – 6.6 (m, 4H), 5.3 (s, 4H). ^{13}C NMR (101 MHz, Chloroform-d) δ 186.5, 167.6, 140.6, 136.3, 131.6, 128.9, 128.5, 128.3, 124.7, 119.2, 56.0. LRMS (ESI) m/z: [M-2Br-2Bn+K]+H calcd. for $C_{16}H_{13}N_2O_2K$ 304.1; found 304.3.

1.2.47 3,4-Dimorpholinocyclobut-3-ene-1,2-dione (16a)

To 3,4-diethoxycyclobut-3-ene-1,2-dione (1.5 g, 1.30 mL, 8.81 mmol, 1.0 Equiv.) dissolved in EtOH (20 mL) was added morpholine (3.1 g, 3.04 mL, 35.3 mmol, 4.0 Equiv.). The reaction was then refluxed for a period of 24 h. The reaction was then cooled to r.t., filtered and the precipitate washed with cold hexane. The title compound was obtained as a white crystalline solid (2.17 g, 97%). M. p. 273-276 °C. ^{1}H NMR (600 MHz, Chloroform-d)

δ 3.8 – 3.8 (m, 8H), 3.7 – 3.6 (m, 8H). ^{13}C NMR (151 MHz, DMSO-*d₆*) δ 183.7, 167.3, 65.9, 48.1. LRMS (ESI) m/z: $[M+Na]^+$ calcd. for $C_{12}H_{16}N_2O_4Na$ 275.1; found 275.1. Spectroscopic data was in accordance with literature data.[3]

1.2.48 3,4-Di(piperidin-1-yl)cyclobut-3-ene-1,2-dione (16b)

To 3,4-diethoxycyclobut-3-ene-1,2-dione (1.3 g, 1.1 mL, 7.64 mmol, 1.0 Equiv.) dissolved in EtOH (10 mL) was added piperidine (2.6 g, 3.0 mL, 30.6 mmol, 4.0 Equiv.). The reaction was then refluxed for a period of 12 h. The reaction was then cooled to r.t., filtered and the precipitate washed with cold hexane. The title compound was obtained as a white solid (1.72 g, 91%). M. p. 169-172 °C. ^{1}H NMR (600 MHz, Chloroform-*d*) δ 3.56 (s, 8H), 1.68 (s, 12H). ^{13}C NMR (151 MHz, DMSO-d_6) δ 183.5, 167.6, 48.8, 25.5, 23.0. HRMS (ESI) m/z: $[M+Na]^+$ calcd. for $C_{14}H_{20}N_2O_2Na$ 271.1; found 271.1. Spectroscopic data was in accordance with literature data.[3]

1.2.49 2,5-Diisopropyl-2,5-diazabicyclo[4.2.0]oct-1(6)-ene-7,8-dione (17a)

To 3,4-diethoxycyclobut-3-ene-1,2-dione (755 mg, 0.66 mL, 4.43 mmol, 1.0 Equiv.) and diisopropylaminoethane (640 mg, 0.80 mL, 4.43 mmol, 1.0 Equiv.) dissolved in EtOH (12 mL) was added NEt_3 (1.79 g, 2.5 mL, 17.3 mmol, 4.0 Equiv.). The reaction was refluxed for a period of 24 h before being cooled to r.t., concentrated under reduced pressure and purified via column chromatography (100% EtOAc). The title compound was obtained as a crystalline white solid after recrystalising from water (650 mg, 66%). M. p. 171-173 °C. ^{1}H NMR (600 MHz, Chloroform-*d*) δ 3.92 (dq, *J* = 12.9, 6.7 Hz, 2H), 3.43 (s, 4H), 1.34 (d, *J* = 6.7 Hz, 12H). ^{13}C NMR (151 MHz, Chloroform-*d*) δ 180.0, 167.3, 53.1, 42.9, 21.0. LRMS (ESI) m/z: $[M+Na]^+$ calcd. for $C_{12}H_{18}N_2O^2Na$ 245.1; found 245.1. Spectroscopic data was in accordance with literature data.[3]

1.2.50 2,6-Dimethyl-2,6-diazabicyclo[5.2.0]non-1(7)-ene-8,9-dione (17b)

To 3,4-diethoxycyclobut-3-ene-1,2-dione (1.00 g, 0.88 mL, 5.88 mmol, 1.0 Equiv.) dissolved in EtOH (11 mL) was added N^1,N^3-dimethylpropane-1,3-diamine (660 mg, 0.80 mL, 5.88 mmol, 1.0 Equiv.) The reaction was refluxed for a period of 24 h before being cooled to r.t. The formed precipitate was filtered and washed with cold EtOH and then allowed to dry under reduced pressure. The title compound was obtained a white crystalline solid following recrystallisation from water (531 mg, 50%). Decomposition occurred at 265 °C during melting point determination. ^{1}H NMR (600 MHz, Chloroform-*d*) δ 3.39 (s, 6H), 3.30 (s, 4H), 2.08 – 2.01 (m, 3H). ^{13}C NMR (151 MHz, Chloroform-*d*) δ 181.8, 167.3, 55.0, 38.3, 29.2. LRMS (ESI) m/z: $[M+Na]^+$ calcd. for $C_9H_{12}N_2O_2Na$ 203.1; found 203.1246.1. Spectroscopic data was in accordance with literature data.[3]

1.3 HDAC Assay

1.3.1 HDAC Assay General Information

The activity of HDAC1, HDAC4, HDAC6, HDAC8, and HDAC11 was determined using a fluorometric assay in black half-area 96-well microplates (Greiner Bio-One, Germany). HDAC1 (#50051) and HDAC6 (#50006) were purchased from BPS Bioscience. HDAC4, HDAC8 and HDAC11 were produced in house as previously described.[7-9] The enzymes were pre-incubated with serial dilutions of the compounds for 1 hour at 30 °C in either assay buffer (25 mM Tris-HCl, 75 mM KCl and 0.001% (v/v) Pluronic F-127, pH 8.0) or MAL buffer (137 mM NaCl, 50 mM Tris-HCl, 2.7 mM KCl, 1 mM $MgCl_2$, 0.5 mg/ml bovine serum albumin (BSA), pH 8.0b) for HDAC11. The enzyme reaction was initiated by the addition of the following substrates: 20 µM Boc-Lys(TFA)-AMC for HDAC4 and HDAC8; 80 µM Boc-Lys(TFA)-AMC for HDAC11; and 50 µM Boc-Lys(Ac)-AMC for HDAC1 and HDAC6. After an incubation period of 60 minutes, the reaction was terminated by the addition of 1.7 µM 9,9,9-trifluoro-8-oxo-N-phenyl-nonanamide (SATFMK) for HDAC4 and HDAC8, 4.2 µM suberoylanilide hydroxamic acid (SAHA) for HDAC1 and HDAC6, and 1 µM 1,1-dimethyl-2-[5-(trifluoromethyl)pyrazin-2-yl]isoindoline-4-carbohydroxamic acid (FT895) for HDAC11. The deacetylated substrate was then cleaved into fluorescent 7-amino-4-methylcoumarin (AMC) by the addition of 0.42 mg/mL trypsin. Measurements were performed using a PHERAstar FS fluorescence microplate reader (BMG LABTECH) with excitation at 360 nm and emission at 460 nm. The normalised enzyme activities were plotted against the respective ligand concentration and fitted with a four-parameter logistic function using GraphPad Prism 6 software.

1.3.2 HDAC Compound Identification and Numbering Convention

Table S1. Conversion of Compound ID to Manuscript ID with Structures.

Compound ID	Manuscript Compound ID	Structure
**14**	**9h**	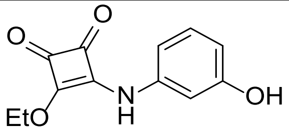
**15**	**9i**	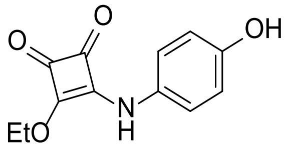
**21**	**9f**	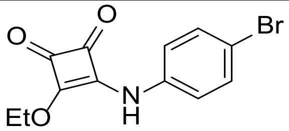
**41**	**9b**	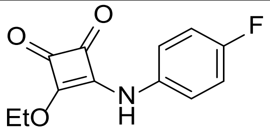
**77**	**12i**	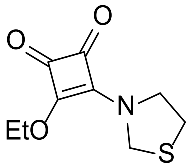
**92**	**9n**	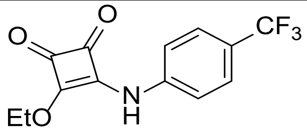
**99**	**9j**	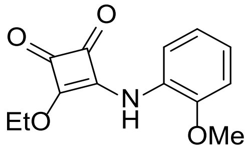
**101**	**9s**	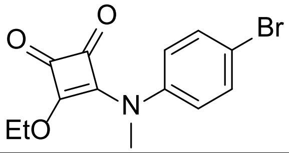
**115**	Not Included in Manuscript Omitted For Future Publication	Not Included in Manuscript Omitted For Future Publication
**116**	**13b**	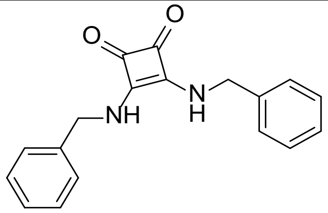

**123**	**14a**	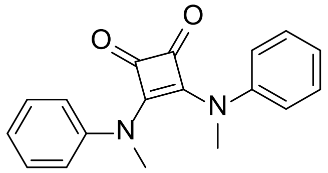
**126**	**14b**	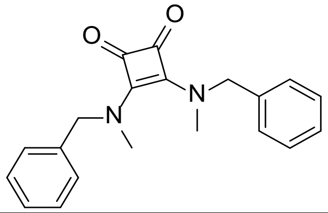
**135**	**13c**	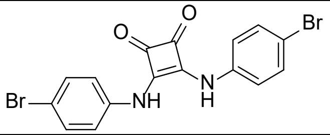
**137**	**14c**	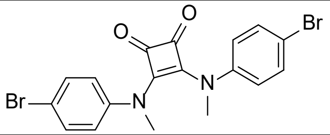
**138**	**15**	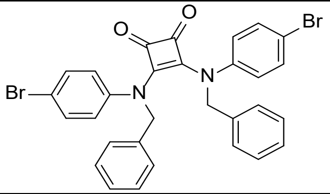

1.3.3 HDAC IC_{50} Biological Data

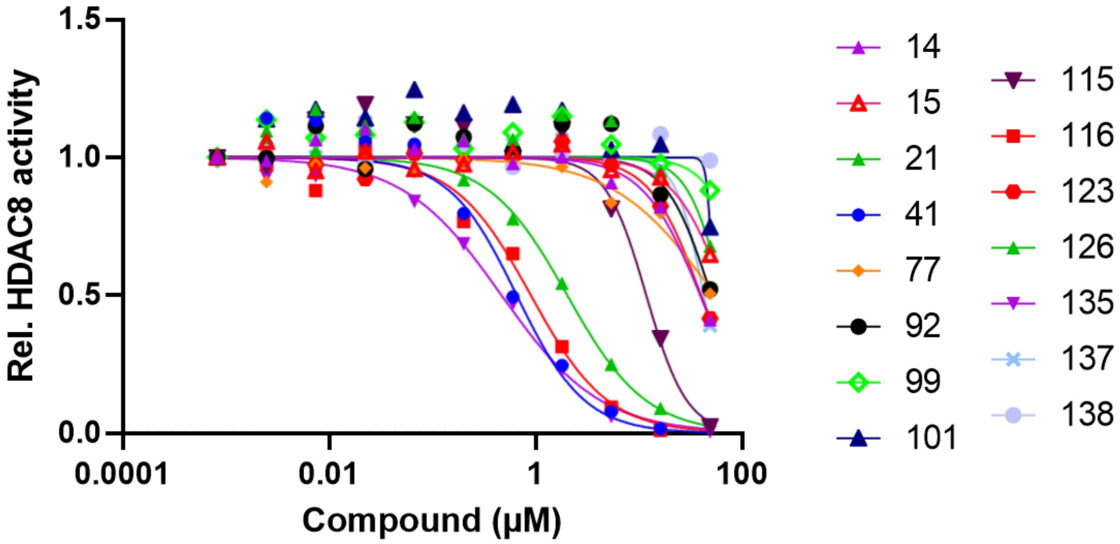

Figure S1. Dose-response curves of HDAC8 activity assay for indicated compounds (see Table 2).

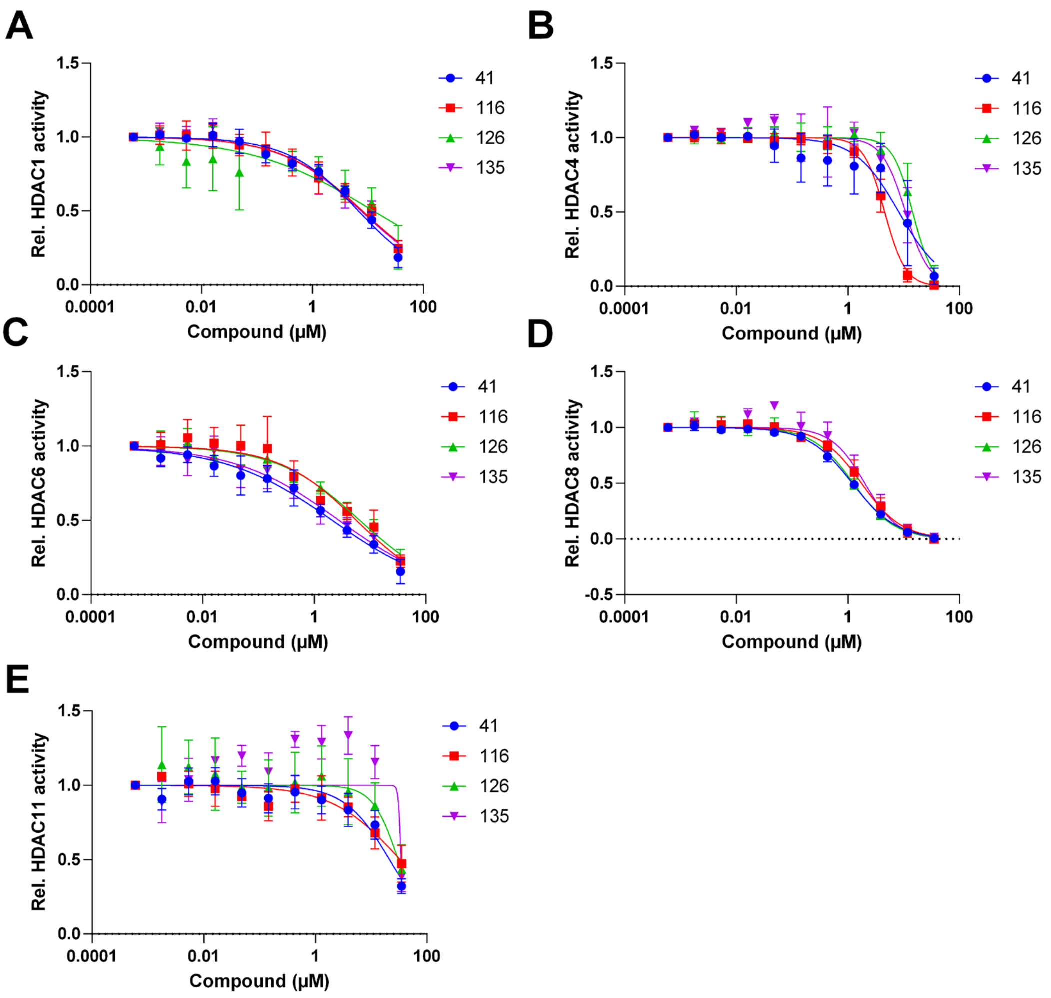

Figure S2. Dose-response curves of HDAC activity assays for indicated best compounds (see Table 2). The HDAC isoenzyme is indicated on the y-axis. Data are shown as means ± standard deviation; N = 3.

1.4 Molecular Docking

Modelling, preparation, and visualisation of structural data, as well as molecular docking, were performed using MOE 2024.0601 software (Chemical Computing Group ULC, Montreal, QC, Canada). The crystal structure of HDAC8 was taken from the Protein Data Bank (PDB-ID: 1T69).[10] All receptor structures were subjected to the Quickprep procedure of MOE 2024, including 3D protonation for subsequent docking. The partial charges of all protein and ligand atoms were calculated using the implemented Amber EHT force field. The docking site was defined by the co-crystallised ligand within the binding pocket of the protein structure. Molecular docking was performed choosing the triangle matcher for placement of the ligand in the binding site and ranked with the London dG scoring function. The best 50 poses were passed onto refinement and energy minimisation in the pocket using the induced fit method, and the 10 best poses were rescored using the GBVI/WSA dG scoring function.

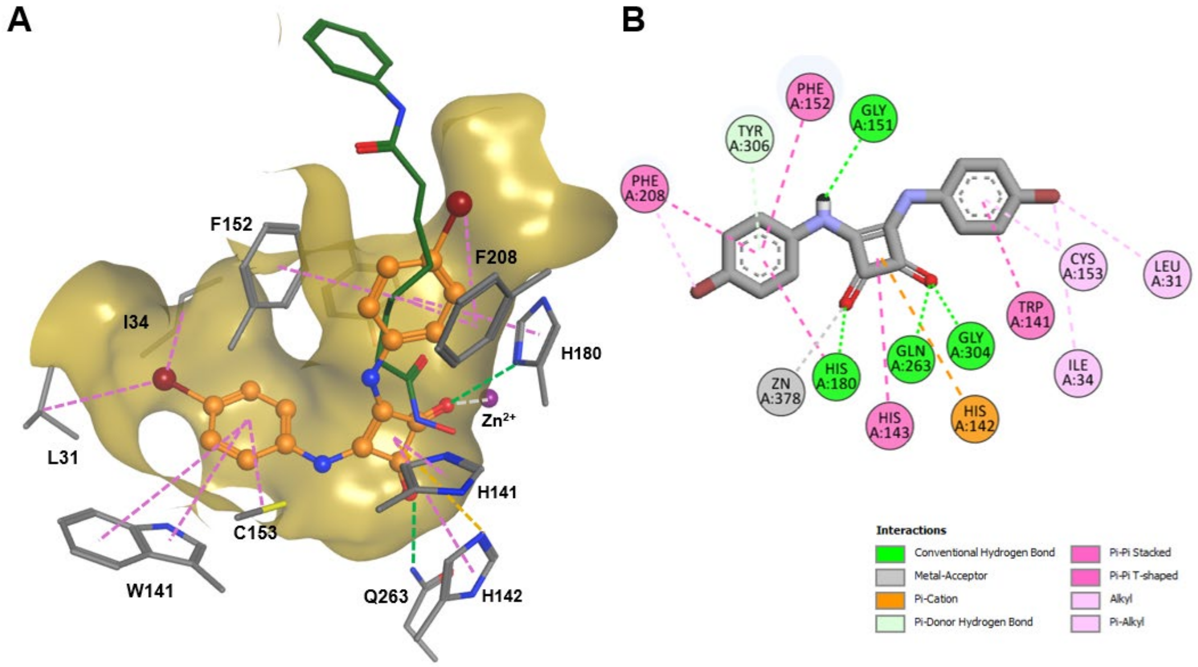

Figure S3. Predicted binding pose of **13c** in HDAC8 (PDB-ID: 1T69). A) 3D-interactions of **13c** with HDAC8. The ligand is shown as ball and sticks. B) 2D-interactions for better overview. The Interactions are coloured as indicated.

1.4.1 Evaluation of Druglikness Using SwissADME [11,12]

Molecule	9C	14B	13B	13C	PCI-34051	SAHA
Structure	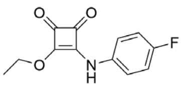	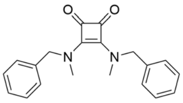	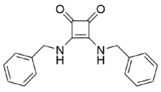	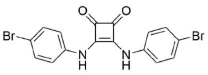	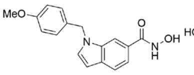	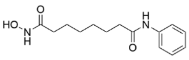
MW	235.21	320.29	292.33	422.07	296.32	264.32
Fraction Csp3	0.17	0.2	0.11	0	0.12	0.43
#Rotatable bonds	4	6	6	4	5	10
#H-bond acceptors	4	2	2	2	3	3
#H-bond donors	1	0	2	2	2	3
MR	58.64	93.2	83.4	95.2	83.1	73.3
TPSA	55.4	40.6	58.2	58.2	63.5	78.4
Consensus Log P	1.7	2.65	2.18	3.39	2.25	1.52
ESOL Solubility (mg/ml)	6.84E-01	4.75E-02	8.86E-02	1.66E-03	1.38E-01	1.58E+00
ESOL Solubility (mol/l)	2.91E-03	1.48E-04	3.03E-04	3.92E-06	4.65E-04	5.97E-03
ESOL Class	Soluble	Soluble	Soluble	Moderately soluble	Soluble	Soluble
GI absorption	High	High	High	High	High	High
BBB permeant	Yes	Yes	Yes	Yes	Yes	No
Pgp substrate	No	No	No	No	Yes	No
CYP1A2 inhibitor	Yes	No	Yes	Yes	Yes	No
CYP2C19 inhibitor	No	Yes	Yes	Yes	No	No
CYP2C9 inhibitor	No	Yes	No	Yes	No	No
CYP2D6 inhibitor	No	Yes	Yes	Yes	Yes	No
CYP3A4 inhibitor	No	Yes	Yes	Yes	No	No
Lipinski #violations	0	0	0	0	0	0
Ghose #violations	0	0	0	0	0	0
Veber #violations	0	0	0	0	0	0
Bioavailability Score	0.85	0.55	0.55	0.55	0.55	0.55
PAINS #alerts	1	1	1	1	0	0
Brenk #alerts	1	1	1	1	2	2
Leadlikeness #violations	1	0	0	2	0	1

References

1 John. R. Dean, *Practical Skills In Chemistry*, Pearson, Third., 2017.

2 N. Long, A. Le Gresley, A. Solomonsz, A. Wozniak, S. Brough and S. P. Wren, *SynOpen*, 2023, 07, 401–407.

3 M. E. Baumert, V. Le, P. H. Su, Y. Akae, D. Bresser, P. Théato and M. M. Hansmann, *J. Am. Chem. Soc.*, 2023, 145, 23334–23345.

4 N. Long, A. Le Gresley, A. Wozniak, S. Brough and S. P. Wren, *Bioorg. Med. Chem.*, 2024, 98, 117565.

5 V. E. Zwicker, K. K. Y. Yuen, D. G. Smith, J. Ho, L. Qin, P. Turner and K. A. Jolliffe, *Chemistry – A European Journal*, 2018, 24, 1140–1150.

6 A. Rostami, A. Colin, X. Y. Li, M. G. Chudzinski, A. J. Lough and M. S. Taylor, *Journal of Organic Chemistry*, 2010, 75, 3983–3992.

7 N. Upadhyay, K. Tilekar, S. Safuan, A. P. Kumar, M. Schweipert, F. J. Meyer-Almes and C. S. Ramaa, *Bioorg. Chem.*, 2021, 116, 105350.

8 N. Jänsch, C. Meyners, M. Muth, A. Kopranovic, O. Witt, I. Oehme and F. J. Meyer-Almes, *Redox Biol.*, 2019, 20, 60–67.

9 A. Kopranovic and F. J. Meyer-Almes, *Int. J. Mol. Sci.*, 2025, 26, 5950.

10 J. R. Somoza, R. J. Skene, B. A. Katz, C. Mol, J. D. Ho, A. J. Jennings, C. Luong, A. Arvai, J. J. Buggy, E. Chi, J. Tang, B. C. Sang, E. Verner, R. Wynands, E. M. Leahy, D. R. Dougan, G. Snell, M. Navre, M. W. Knuth, R. V. Swanson, D. E. McRee and L. W. Tari, *Structure*, 2004, 12, 1325–1334.

11 A. Daina, O. Michielin and V. Zoete, *Scientific Reports 2017 7:1*, 2017, 7, 42717-.

12 SwissADME, https://www.swissadme.ch/, (accessed 28 November 2025).

NL22 - Aniline with Zn(OTf)2 - Pure

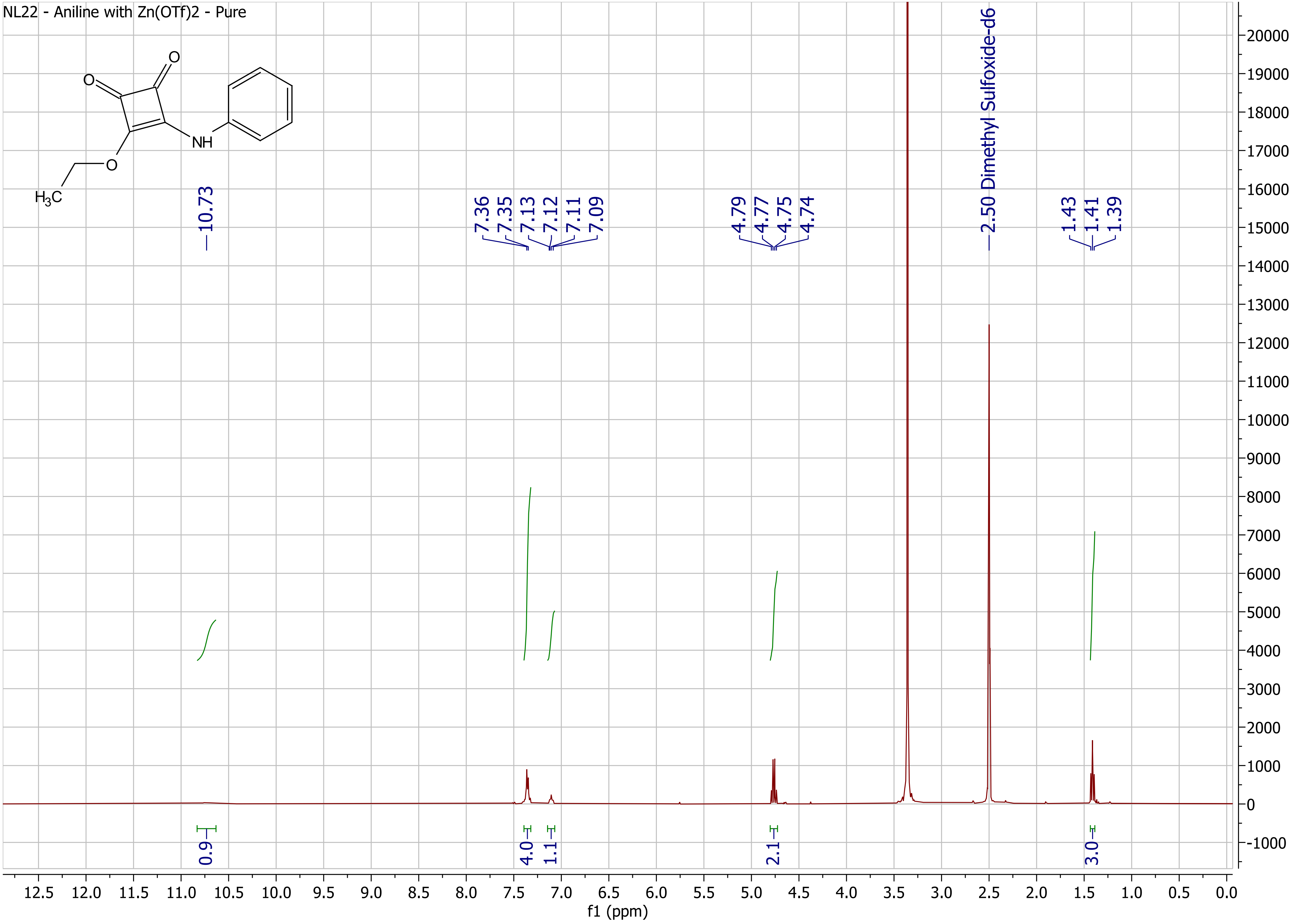

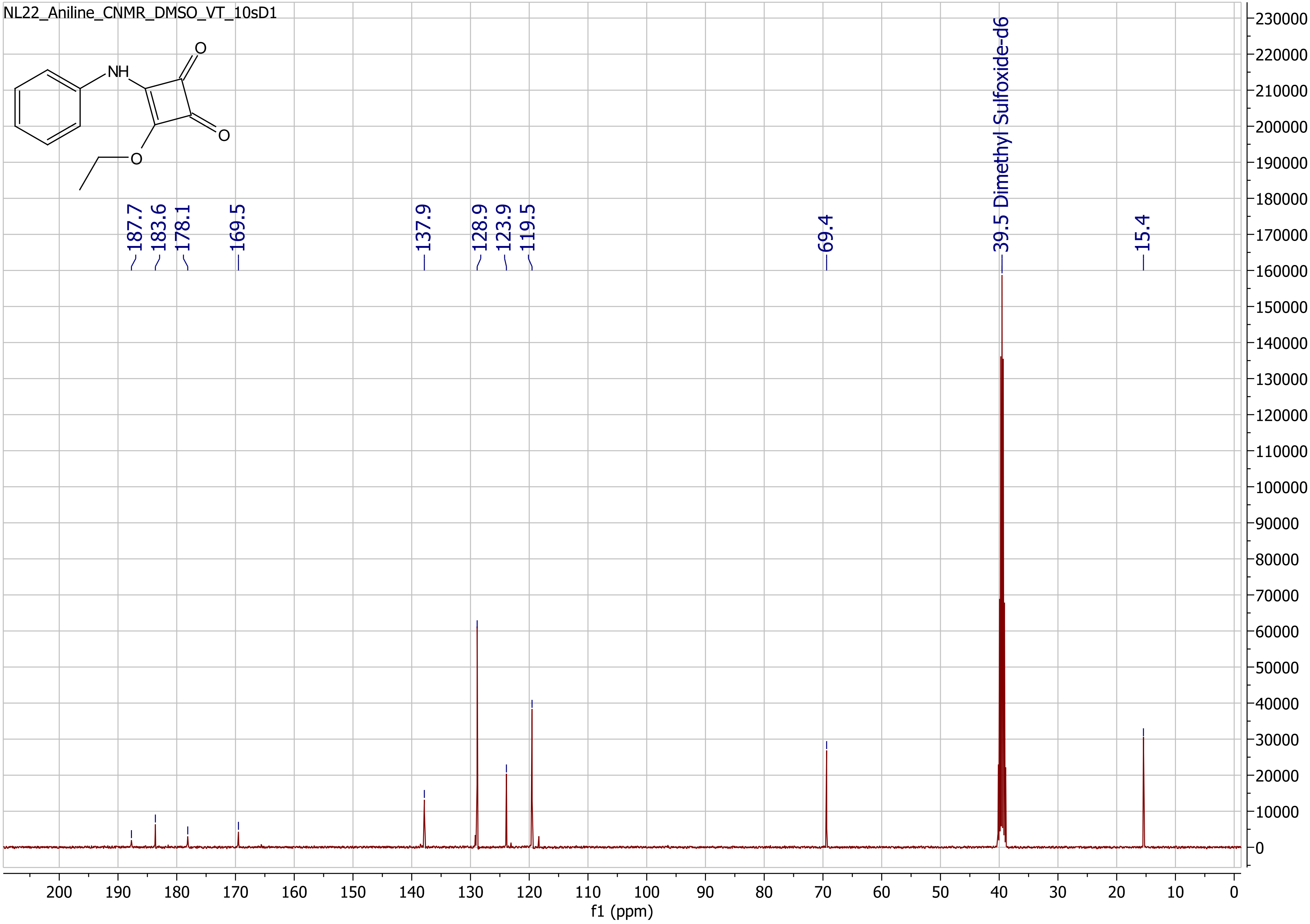
NL22_Aniline_CNMR_DMSO_VT_10sD1
NH
O
O
O
187.7
183.6
178.1
169.5
137.9
128.9
123.9
119.5
69.4
39.5 Dimethyl Sulfoxide-d6
15.4
230000
220000
210000
200000
190000
180000
170000
160000
150000
140000
130000
120000
110000
100000
90000
80000
70000
60000
50000
40000
30000
20000
10000
0
200
190
180
170
160
150
140
130
120
110
100
90
80
70
60
50
40
30
20
10
0
f1 (ppm)

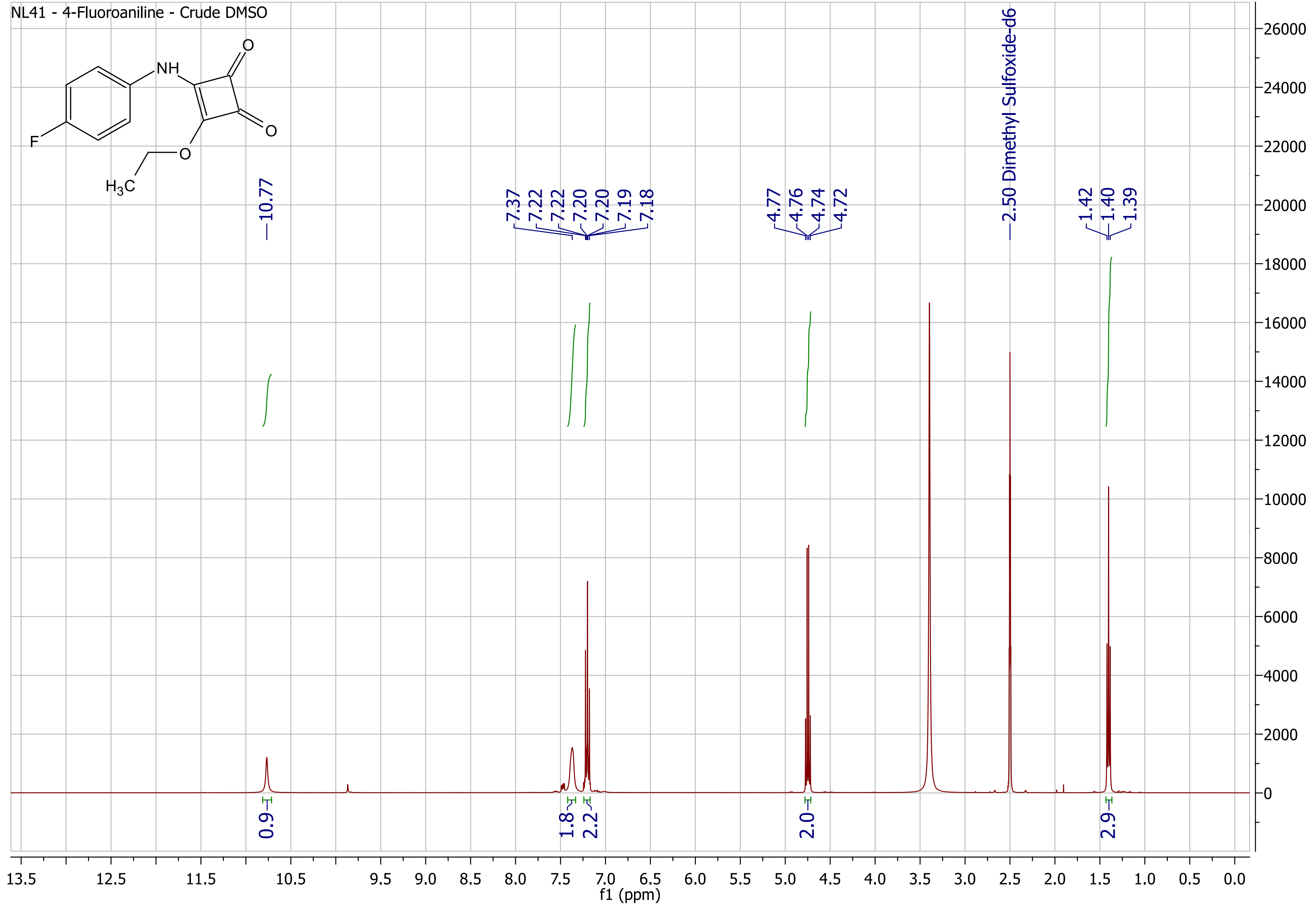
NL41 - 4-Fluoroaniline - Crude DMSO
NH
O
O
F
O
H3C
10.77
7.37
7.22
7.22
7.20
7.20
7.19
7.18
4.77
4.76
4.74
4.72
2.50 Dimethyl Sulfoxide-d6
1.42
1.40
1.39
0.9
1.8
2.2
2.0
2.9
f1 (ppm)

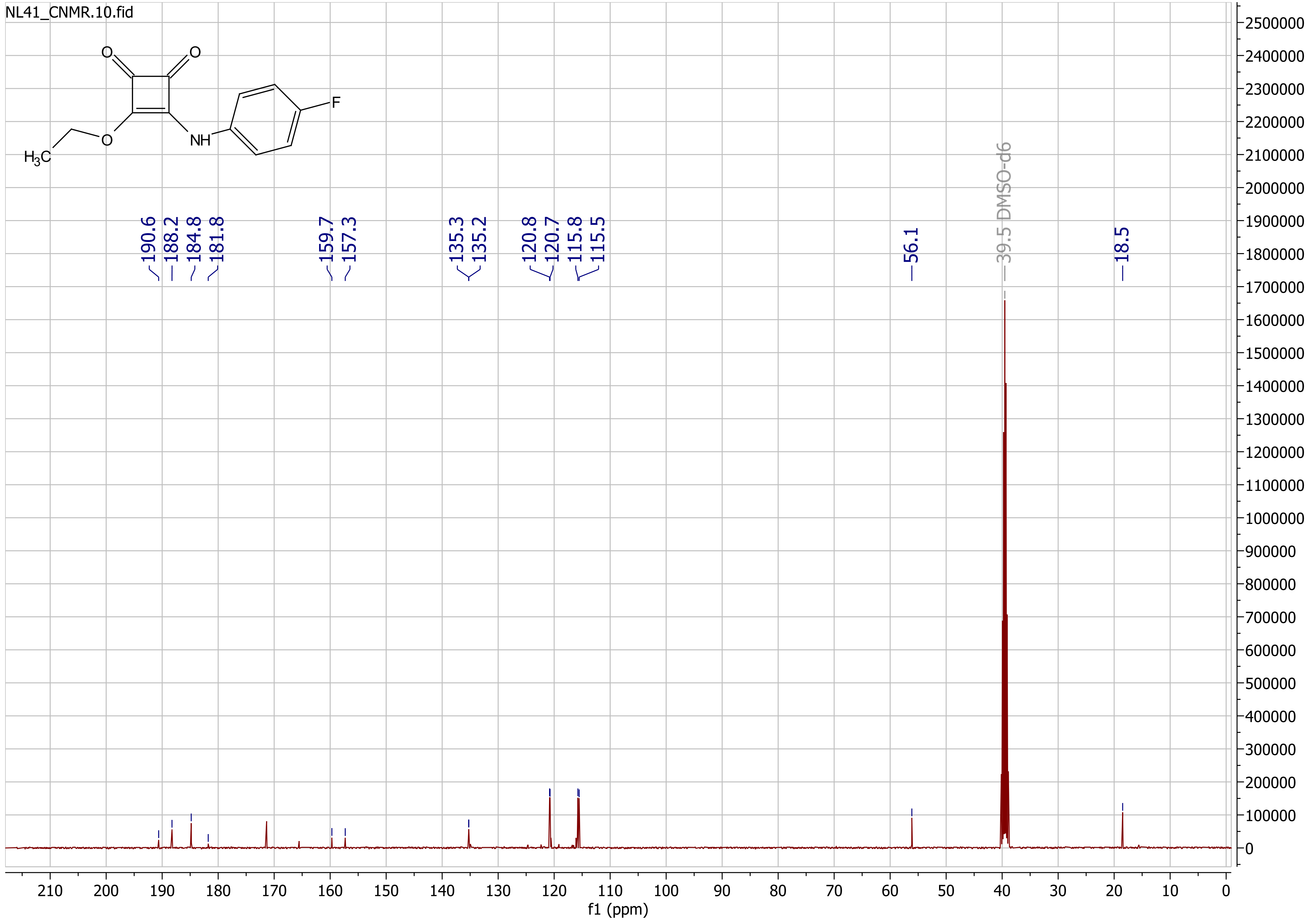

NL41_CNMR.10.fid
O
O
F
NH
O
H3C
190.6
188.2
184.8
181.8
159.7
157.3
135.3
135.2
120.8
120.7
115.8
115.5
56.1
39.5 DMSO-d6
18.5
f1 (ppm)

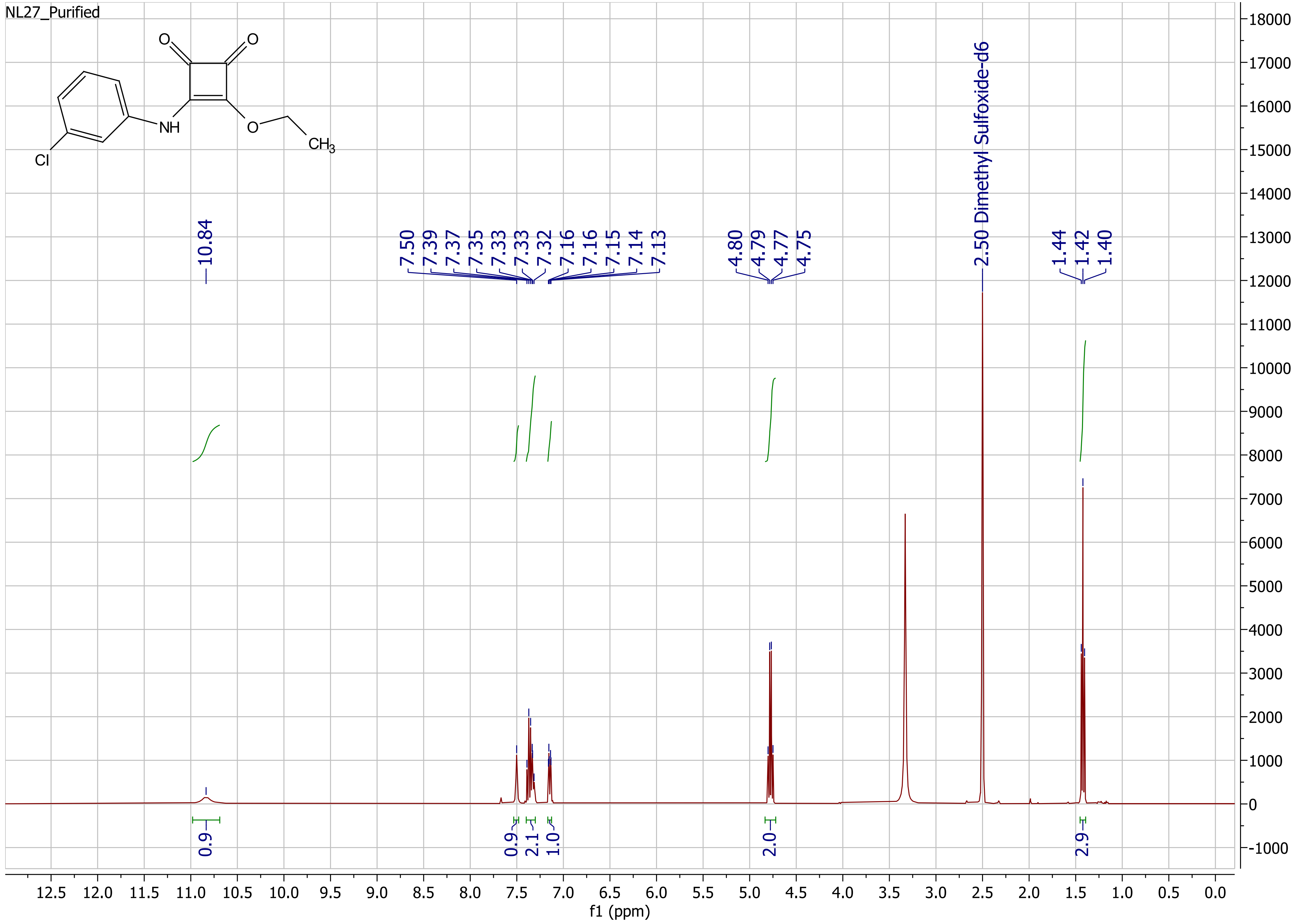

NL27_Purified
O
O
NH
O
CH3
Cl
10.84
7.50
7.39
7.37
7.35
7.33
7.33
7.32
7.16
7.16
7.15
7.14
7.13
4.80
4.79
4.77
4.75
2.50 Dimethyl Sulfoxide-d6
1.44
1.42
1.40
0.9
0.9
2.1
1.0
2.0
2.9
f1 (ppm)

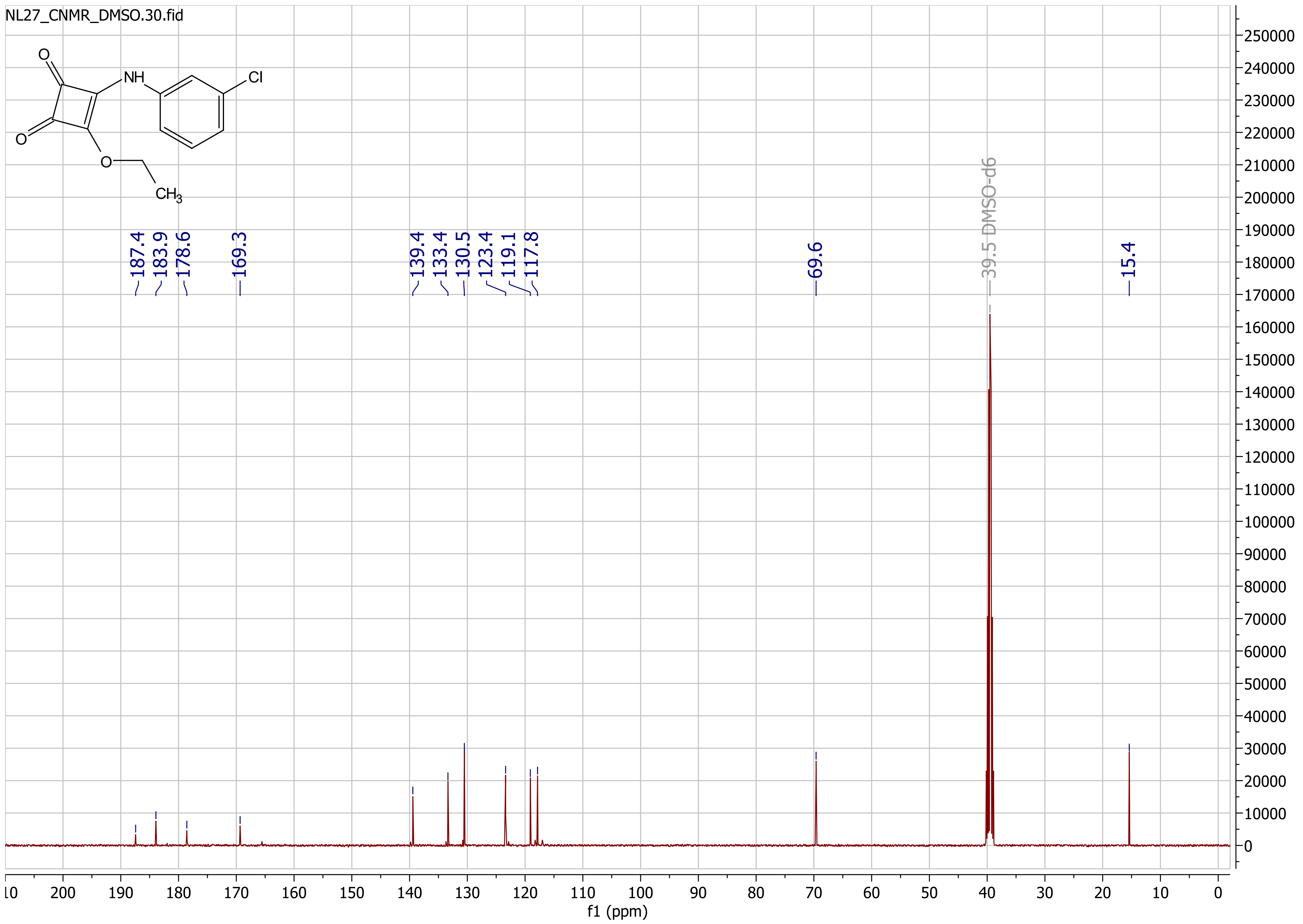
NL27_CNMR_DMSO.30.fid
O
NH
Cl
O
O
CH3
187.4
183.9
178.6
169.3
139.4
133.4
130.5
123.4
119.1
117.8
69.6
39.5 DMSO-d6
15.4
f1 (ppm)

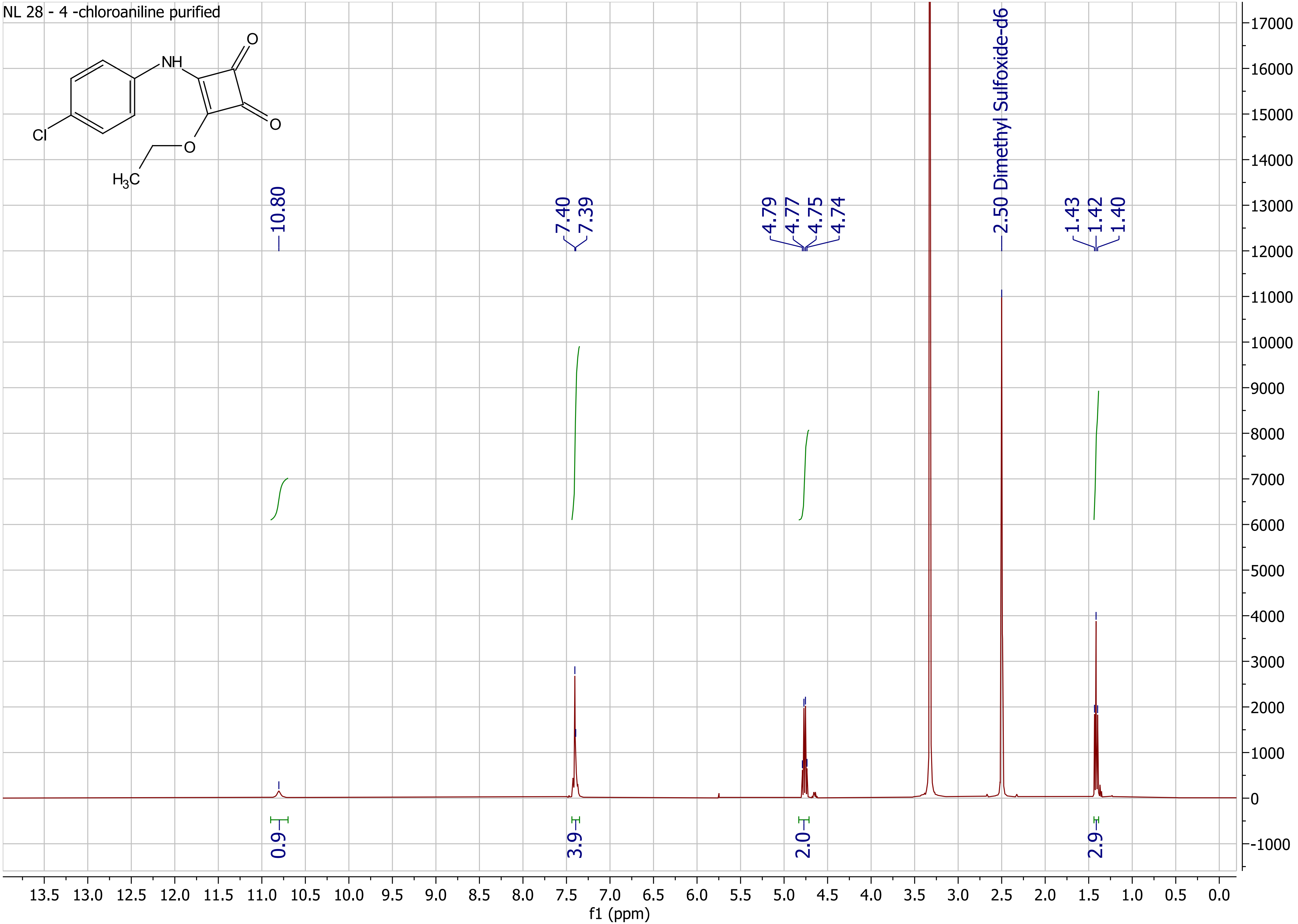
NL 28 - 4 -chloroaniline purified
NH
O
O
O
Cl
H_3C
10.80
7.40
7.39
4.79
4.77
4.75
4.74
2.50 Dimethyl Sulfoxide-d6
1.43
1.42
1.40
0.9
3.9
2.0
2.9
13.5 13.0 12.5 12.0 11.5 11.0 10.5 10.0 9.5 9.0 8.5 8.0 7.5 7.0 6.5 6.0 5.5 5.0 4.5 4.0 3.5 3.0 2.5 2.0 1.5 1.0 0.5 0.0
f1 (ppm)
17000 16000 15000 14000 13000 12000 11000 10000 9000 8000 7000 6000 5000 4000 3000 2000 1000 0 -1000

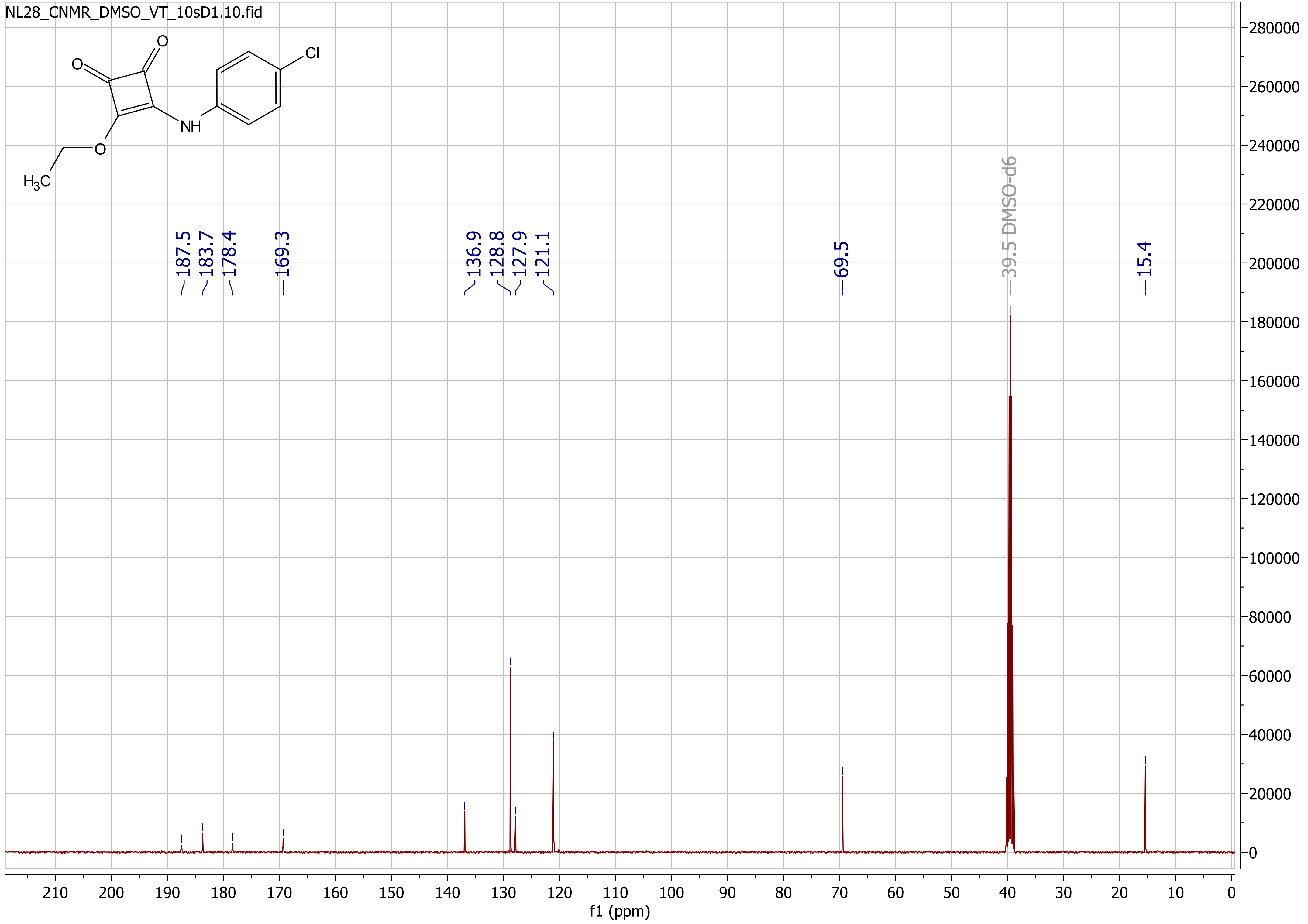
NL28_CNMR_DMSO_VT_10sD1.10.fid
O
O
Cl
NH
O
H3C
187.5
183.7
178.4
169.3
136.9
128.8
127.9
121.1
69.5
39.5 DMSO-d6
15.4
280000
260000
240000
220000
200000
180000
160000
140000
120000
100000
80000
60000
40000
20000
0
210
200
190
180
170
160
150
140
130
120
110
100
90
80
70
60
50
40
30
20
10
0
f1 (ppm)

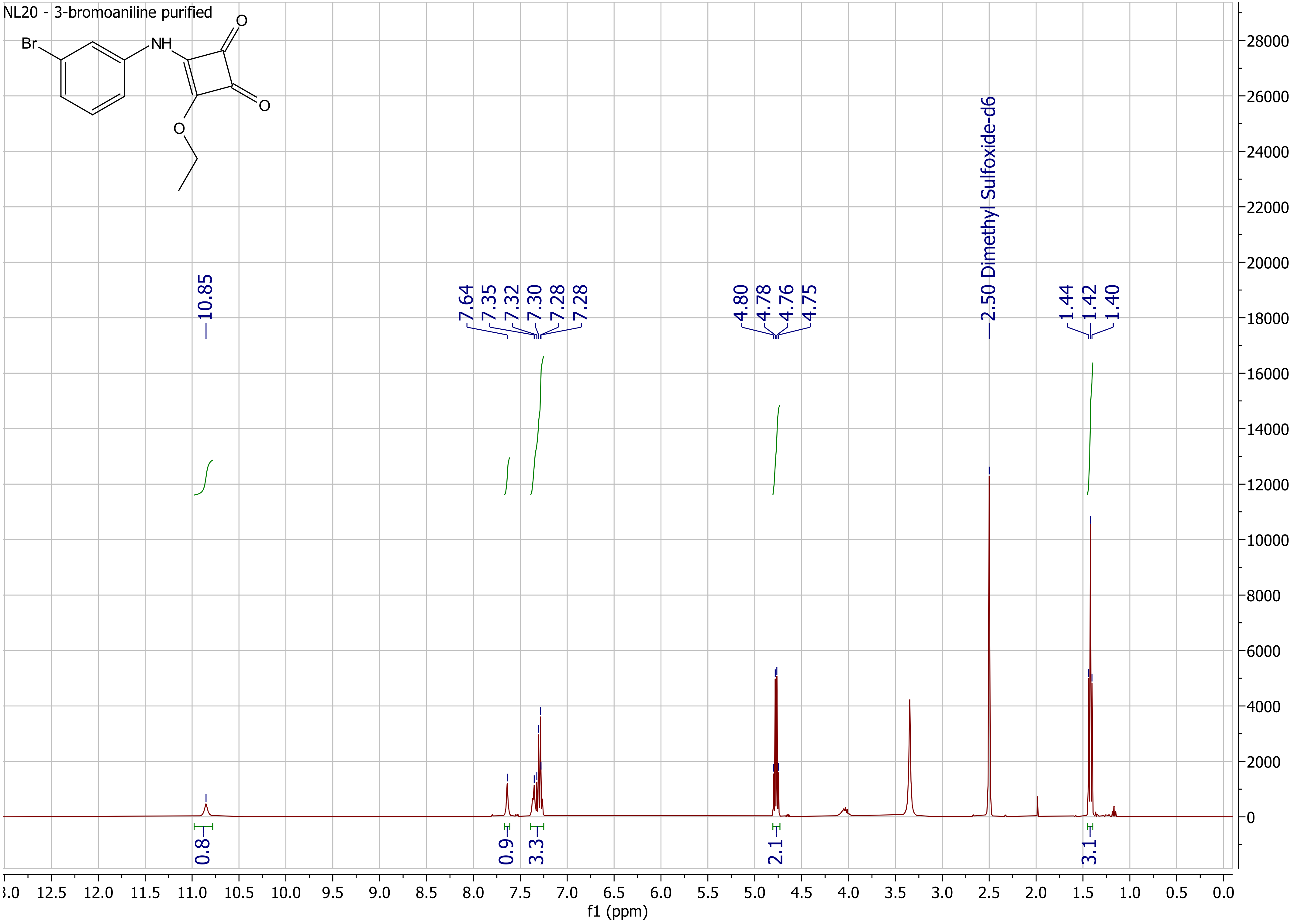
NL20 - 3-bromoaniline purified
Br
NH
O
O
O
10.85
7.64
7.35
7.32
7.30
7.28
7.28
4.80
4.78
4.76
4.75
2.50 Dimethyl Sulfoxide-d6
1.44
1.42
1.40
0.8
0.9
3.3
2.1
3.1
f1 (ppm)

NL20 3-Bromoaniline_CNMR

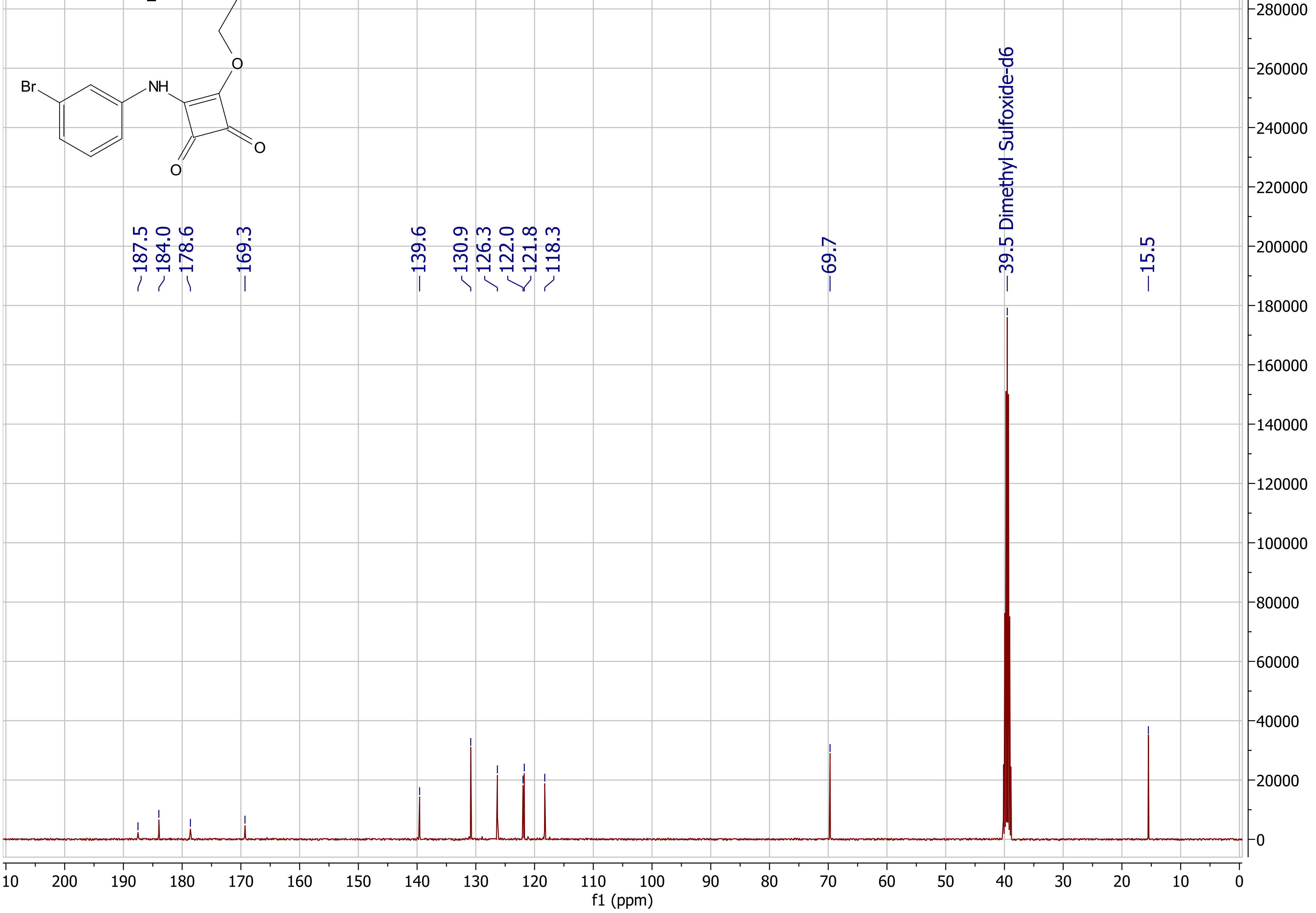

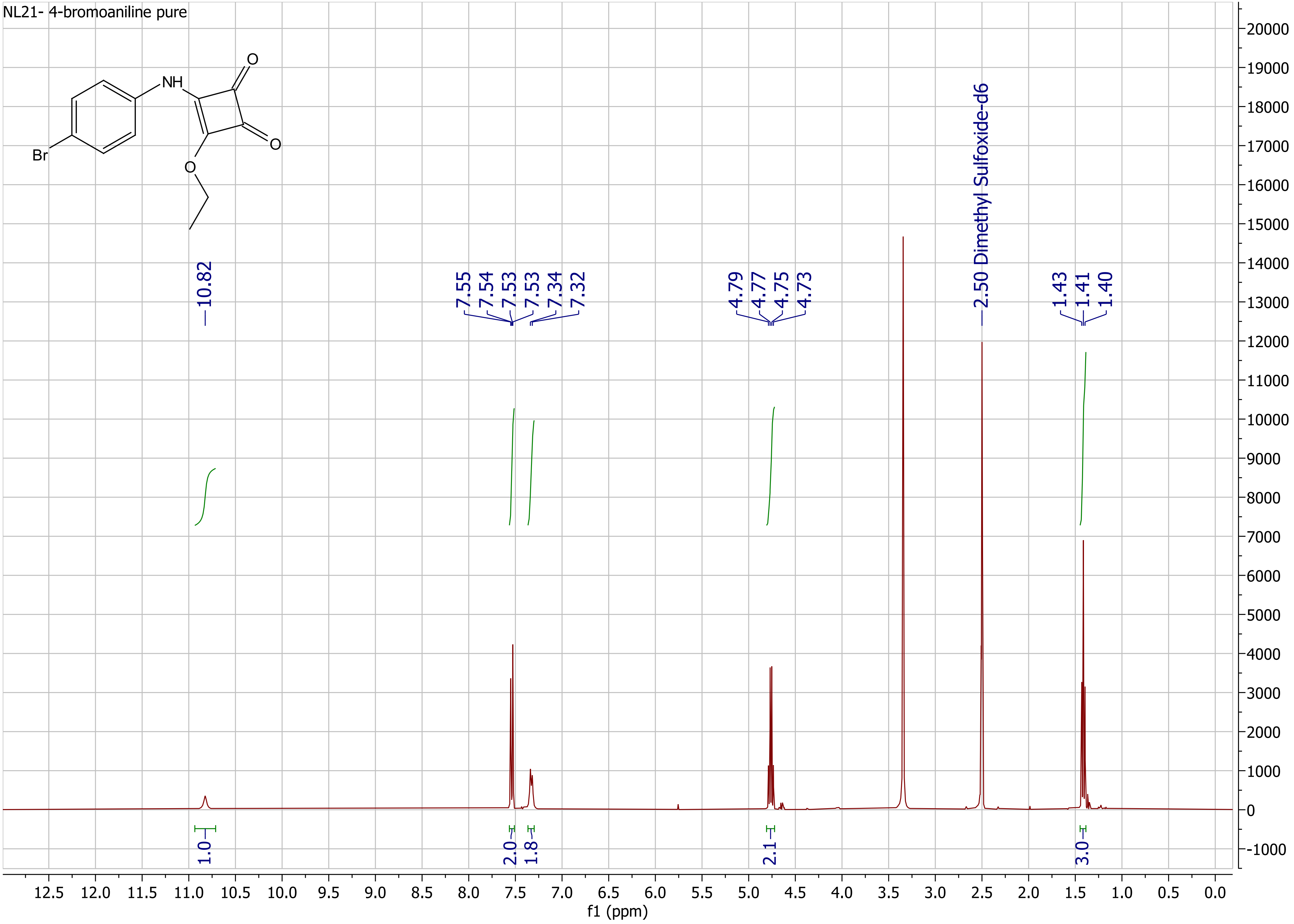
NL21- 4-bromoaniline pure
NH
O
O
Br
O
10.82
7.55
7.54
7.53
7.53
7.34
7.32
4.79
4.77
4.75
4.73
2.50 Dimethyl Sulfoxide-d6
1.43
1.41
1.40
1.0
2.0
1.8
2.1
3.0
f1 (ppm)

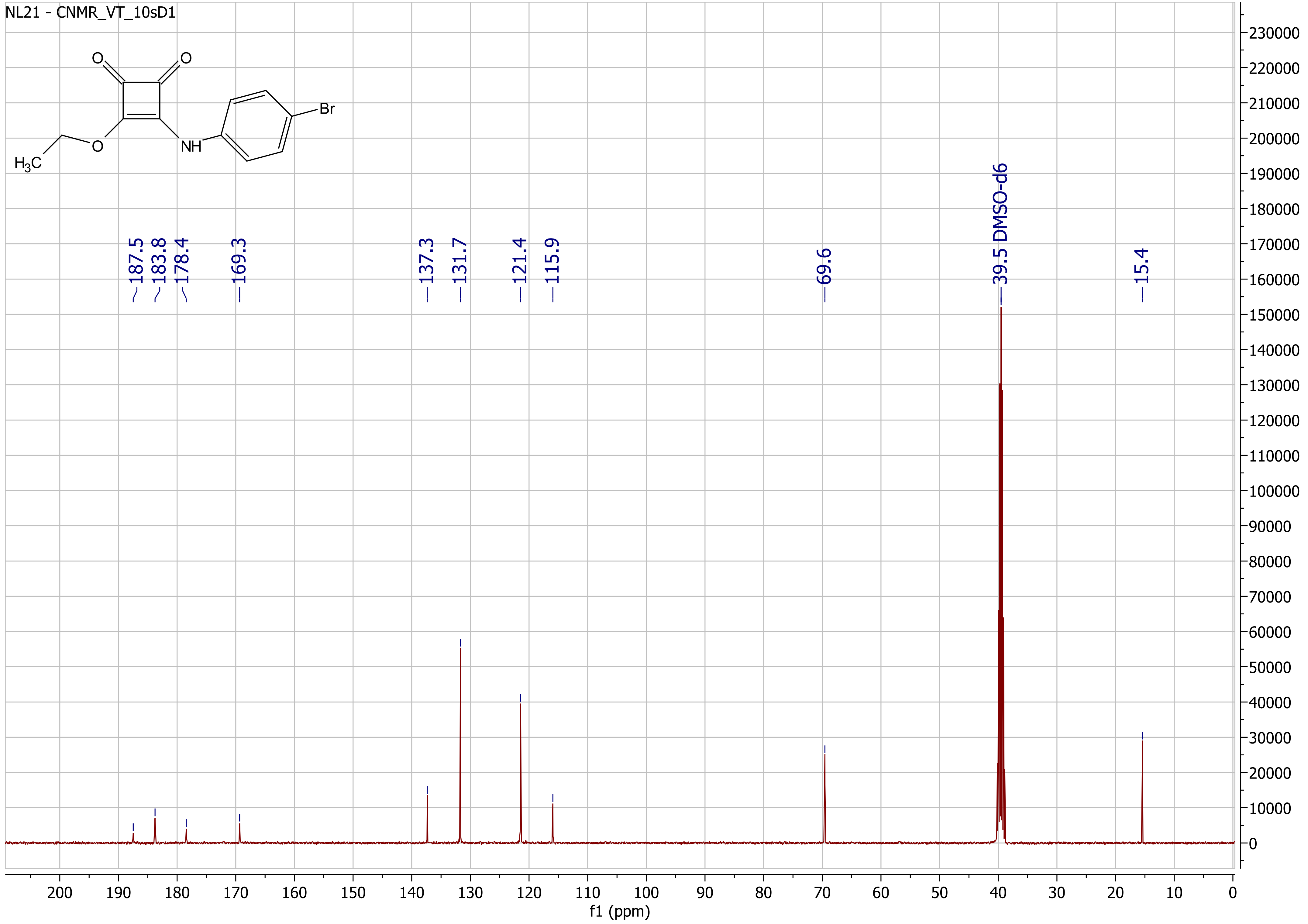

NL21 - CNMR_VT_10sD1
O
O
Br
O
NH
H3C
187.5
183.8
178.4
169.3
137.3
131.7
121.4
115.9
69.6
39.5 DMSO-d6
15.4
230000
220000
210000
200000
190000
180000
170000
160000
150000
140000
130000
120000
110000
100000
90000
80000
70000
60000
50000
40000
30000
20000
10000
0
200
190
180
170
160
150
140
130
120
110
100
90
80
70
60
50
40
30
20
10
0
f1 (ppm)

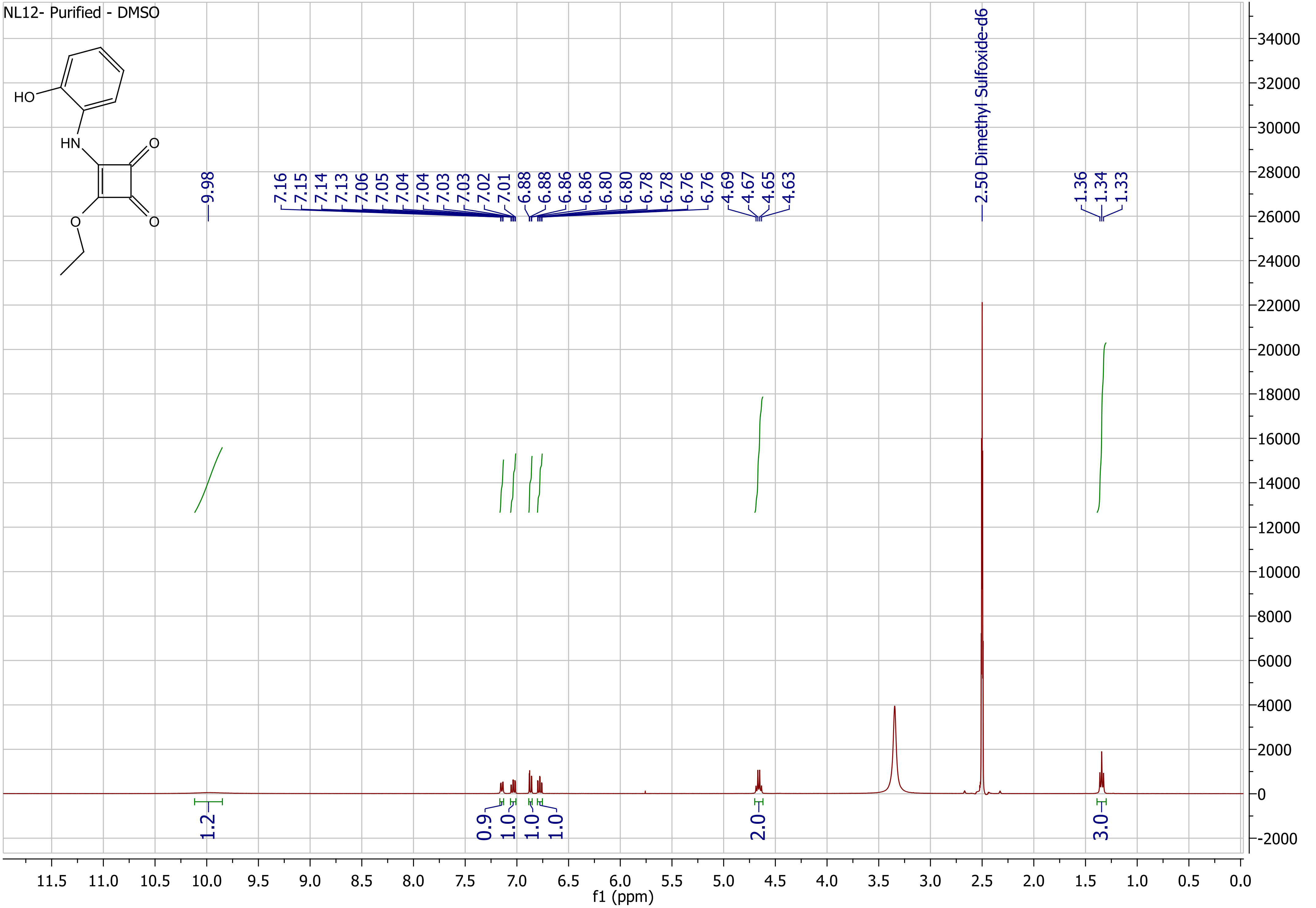
NL12- Purified - DMSO
HO
HN
O
O
O
9.98
7.16
7.15
7.14
7.13
7.06
7.05
7.04
7.04
7.03
7.03
7.02
7.01
6.88
6.88
6.86
6.86
6.80
6.80
6.78
6.78
6.76
6.76
4.69
4.67
4.65
4.63
2.50 Dimethyl Sulfoxide-d6
1.36
1.34
1.33
1.2
0.9
1.0
1.0
1.0
2.0
3.0
f1 (ppm)

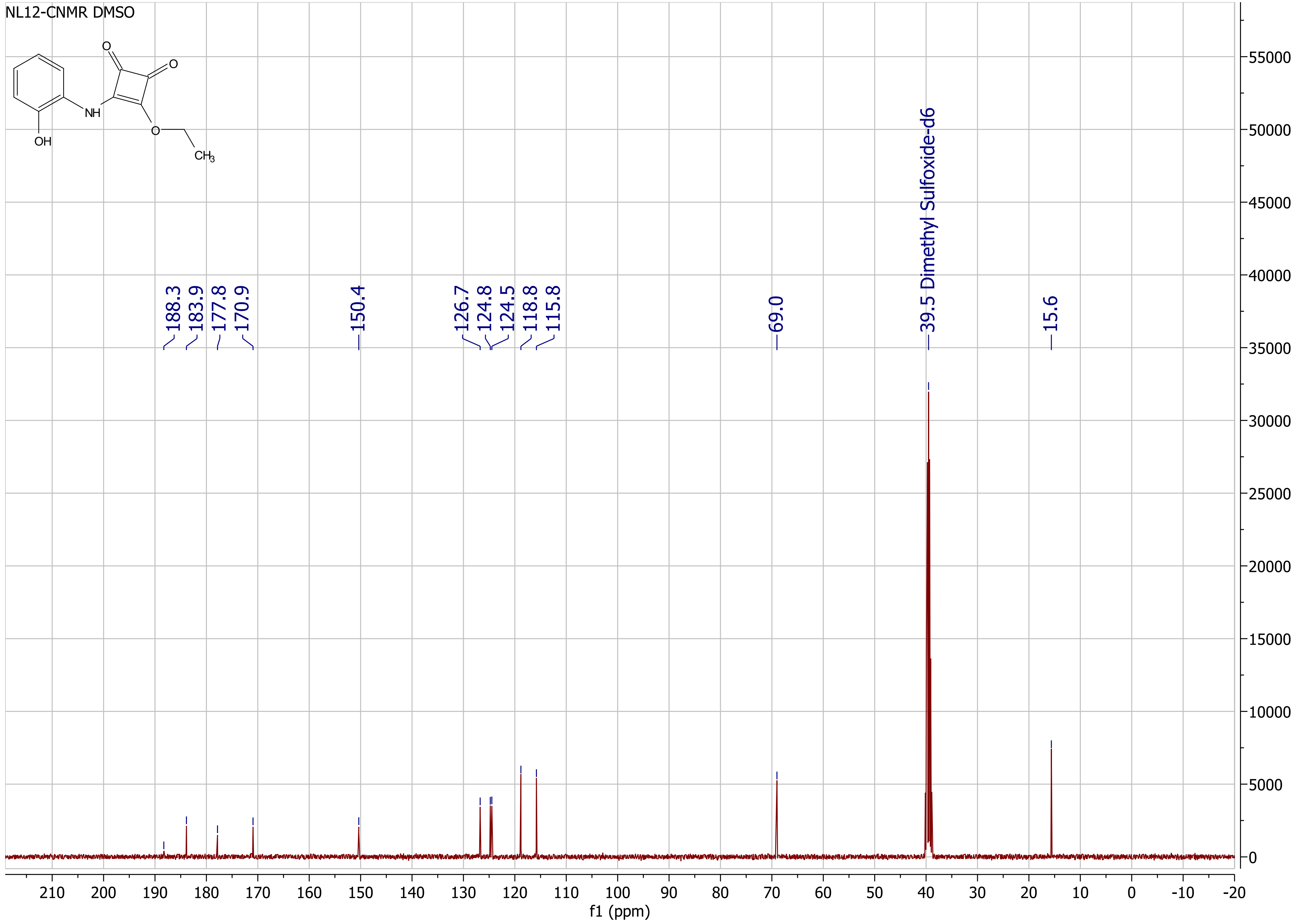
NL12-CNMR DMSO
O
O
NH
O
OH
CH3
188.3
183.9
177.8
170.9
150.4
126.7
124.8
124.5
118.8
115.8
69.0
39.5 Dimethyl Sulfoxide-d6
15.6
55000
50000
45000
40000
35000
30000
25000
20000
15000
10000
5000
0
210
200
190
180
170
160
150
140
130
120
110
100
90
80
70
60
50
40
30
20
10
0
-10
-20
f1 (ppm)

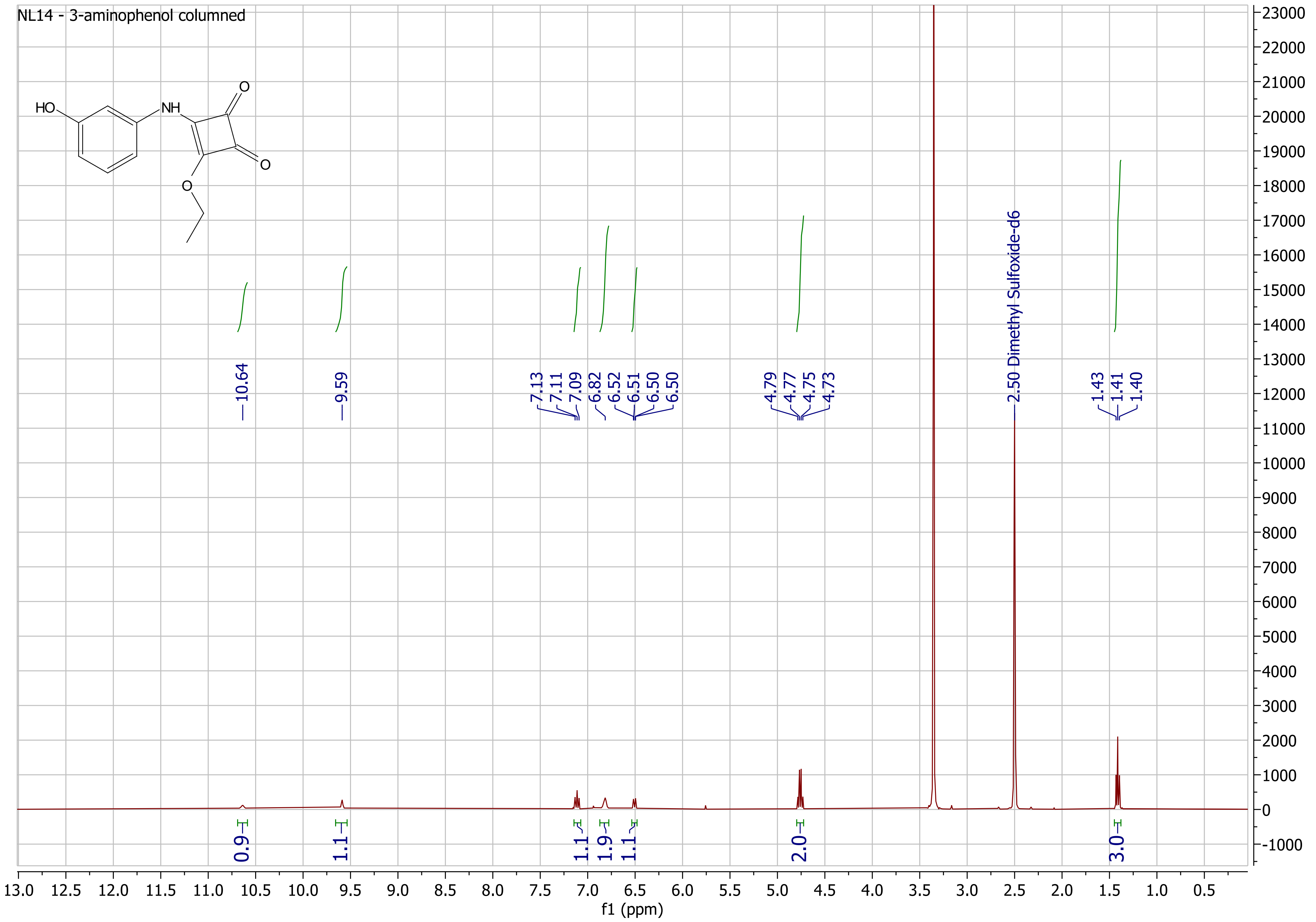

NL14 - 3-aminophenol columned
HO
NH
O
O
O
10.64
9.59
7.13
7.11
7.09
6.82
6.52
6.51
6.50
6.50
4.79
4.77
4.75
4.73
2.50 Dimethyl Sulfoxide-d6
1.43
1.41
1.40
0.9
1.1
1.1
1.9
1.1
2.0
3.0
f1 (ppm)

NL14 CNMR

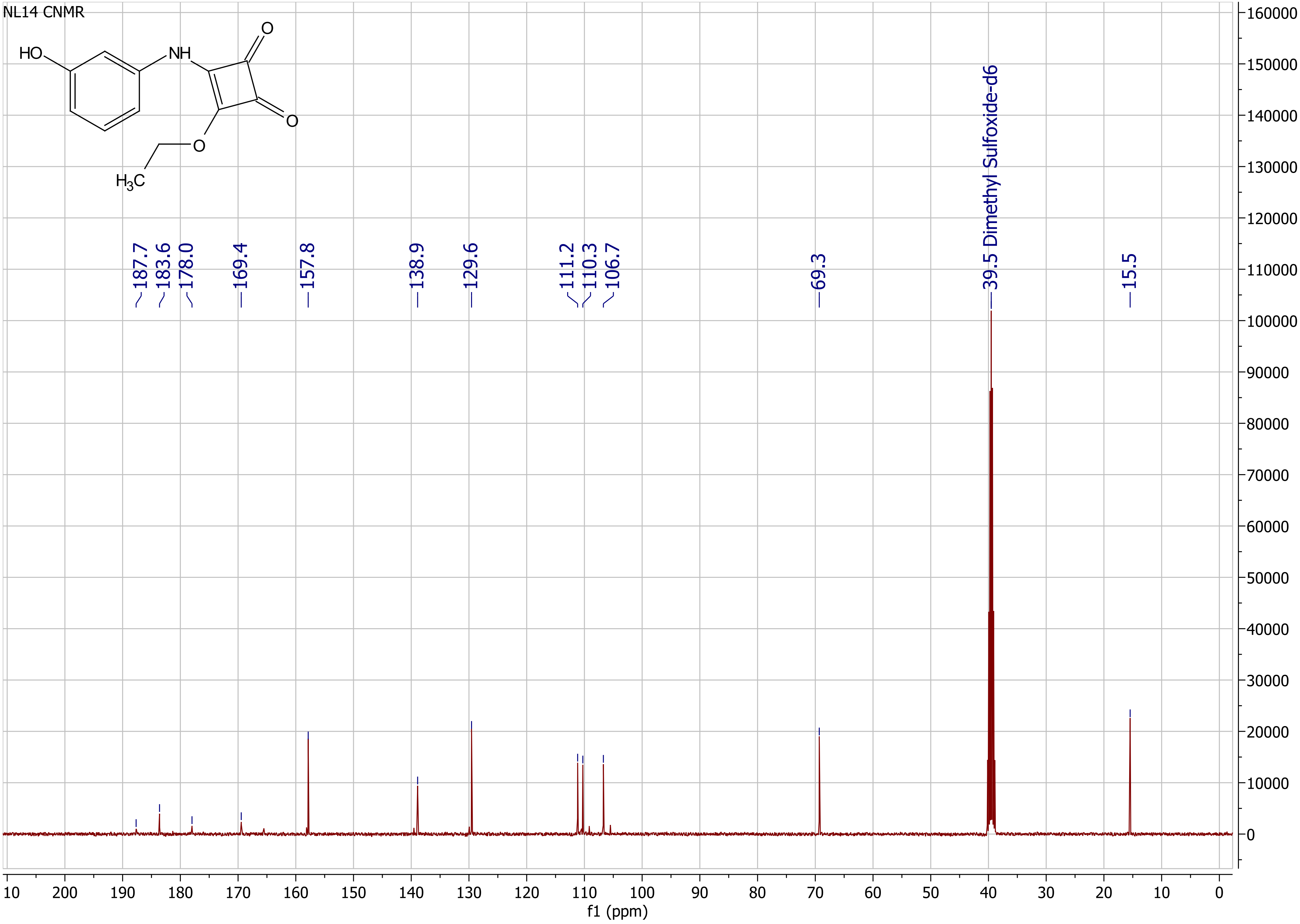

HO
NH
O
O
O
H3C
187.7
183.6
178.0
169.4
157.8
138.9
129.6
111.2
110.3
106.7
69.3
39.5 Dimethyl Sulfoxide-d6
15.5
f1 (ppm)

NL15 - 4-hydroxyaniline - Purified

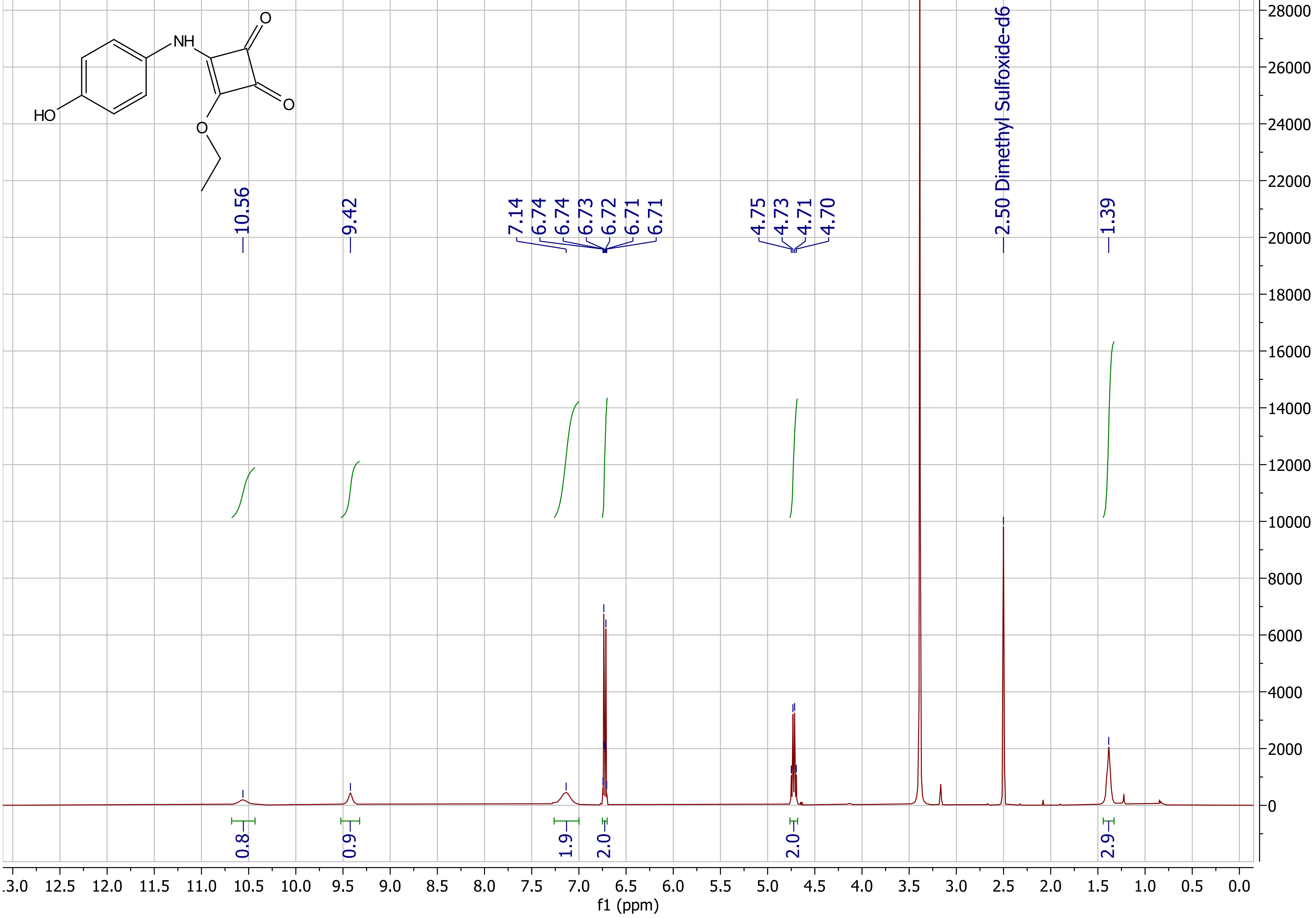

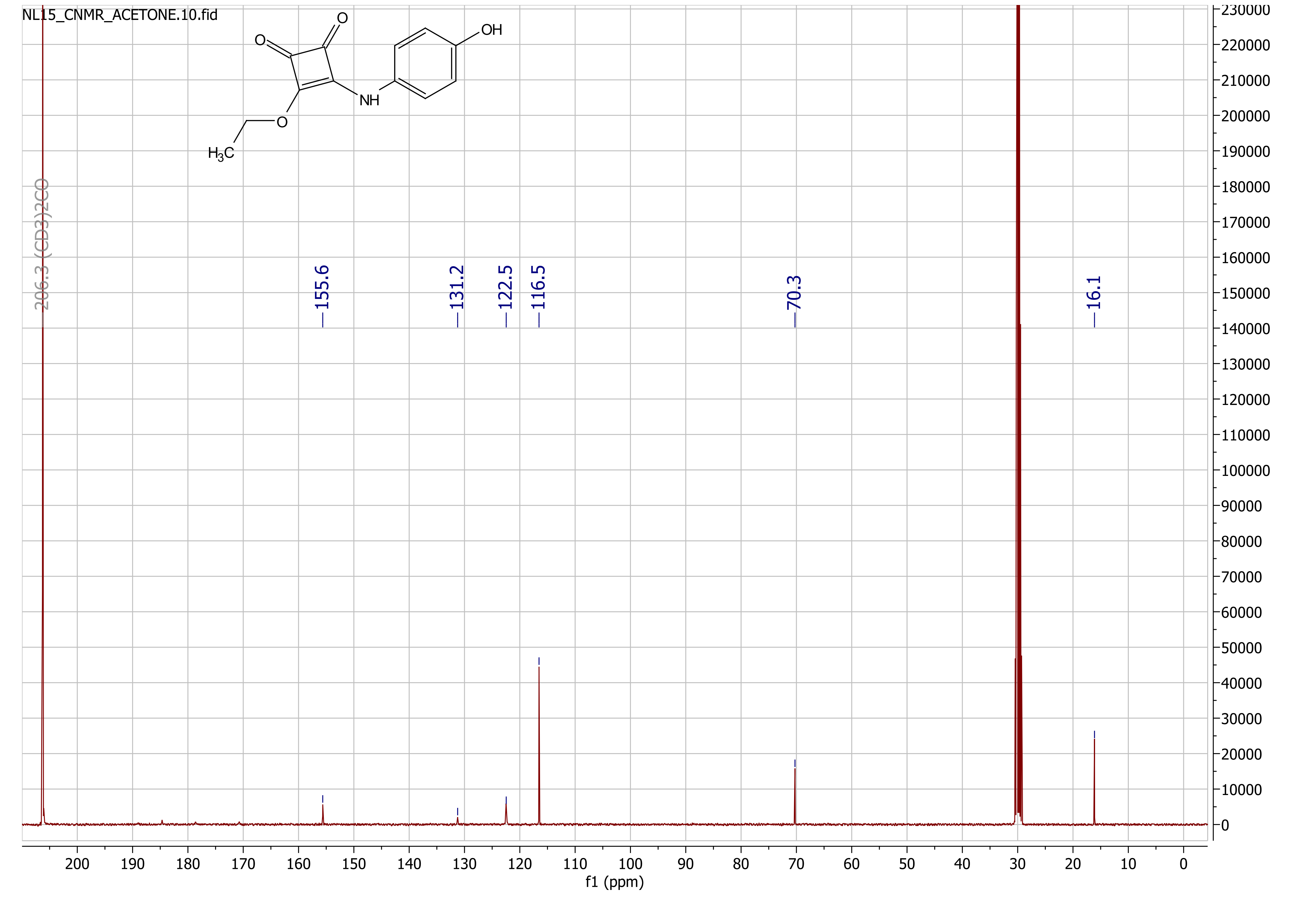
NL15_CNMR_ACETONE.10.fid
O
O
OH
NH
O
H3C
206.3 (CD3)2CO
155.6
131.2
122.5
116.5
70.3
16.1
f1 (ppm)

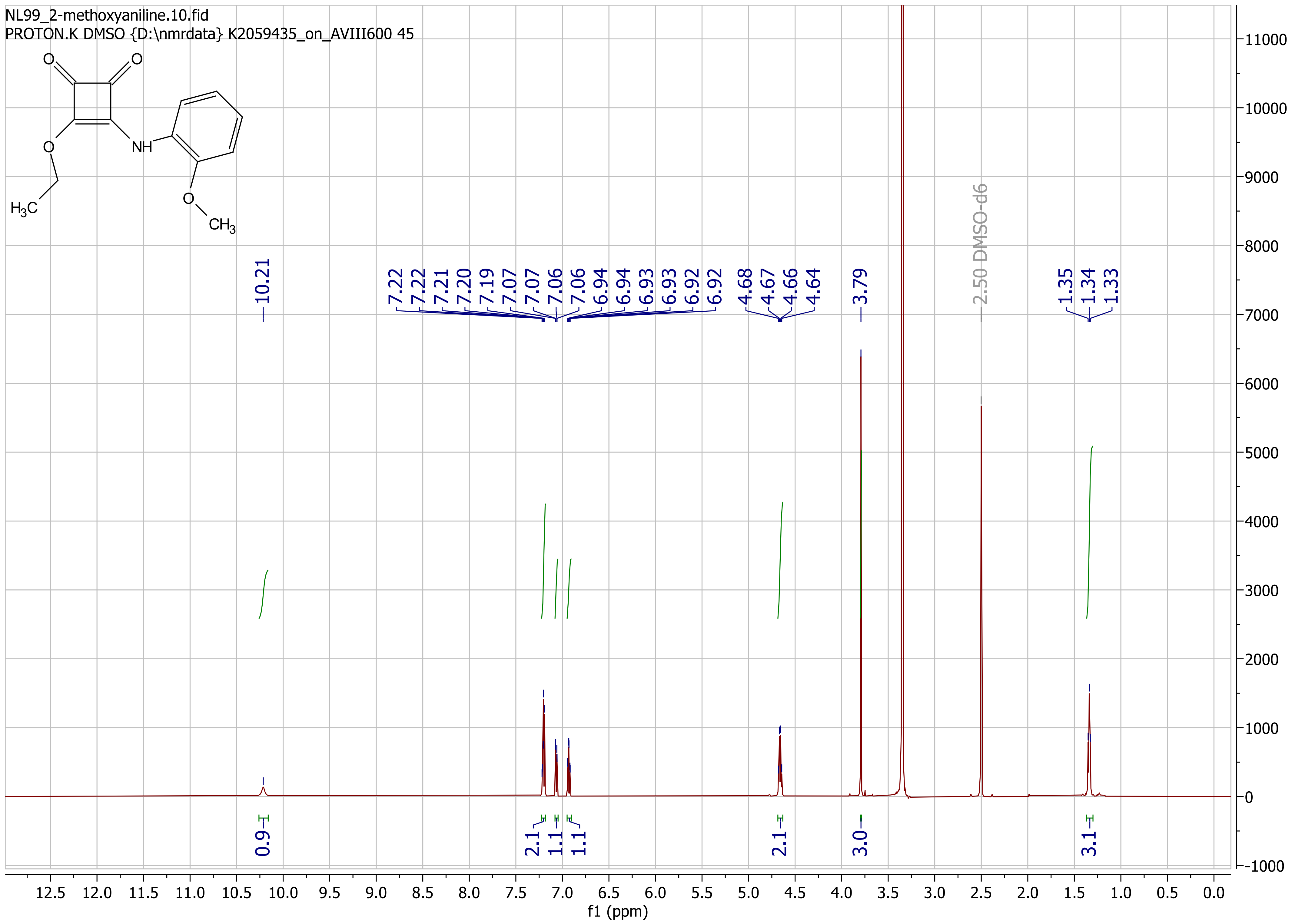
NL99_2-methoxyaniline.10.fid
PROTON.K DMSO {D:\nmrdata} K2059435_on_AVIII600 45
O
O
O
NH
H3C
O
CH3
10.21
7.22
7.22
7.21
7.20
7.19
7.07
7.07
7.06
7.06
6.94
6.94
6.93
6.93
6.92
6.92
4.68
4.67
4.66
4.64
3.79
2.50 DMSO-d6
1.35
1.34
1.33
0.9
2.1
1.1
1.1
2.1
3.0
3.1
11000
10000
9000
8000
7000
6000
5000
4000
3000
2000
1000
0
-1000
12.5 12.0 11.5 11.0 10.5 10.0 9.5 9.0 8.5 8.0 7.5 7.0 6.5 6.0 5.5 5.0 4.5 4.0 3.5 3.0 2.5 2.0 1.5 1.0 0.5 0.0
f1 (ppm)

NL99_2-Methoxyaniline CNMR.30.fid
13C setup
C13CPDVT.K DMSO {D:\nmrdata} K2059435_on_AVIII600 34

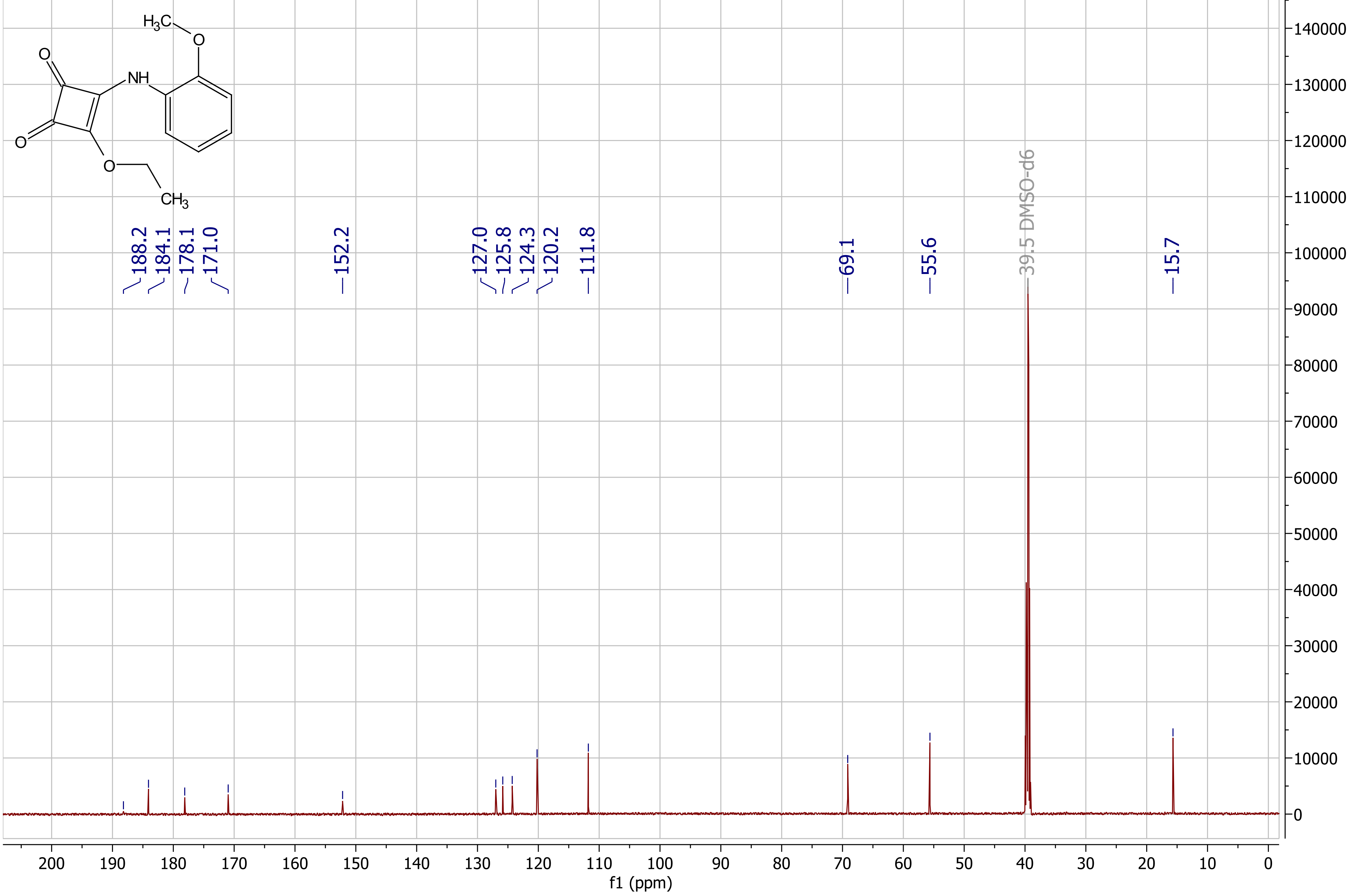

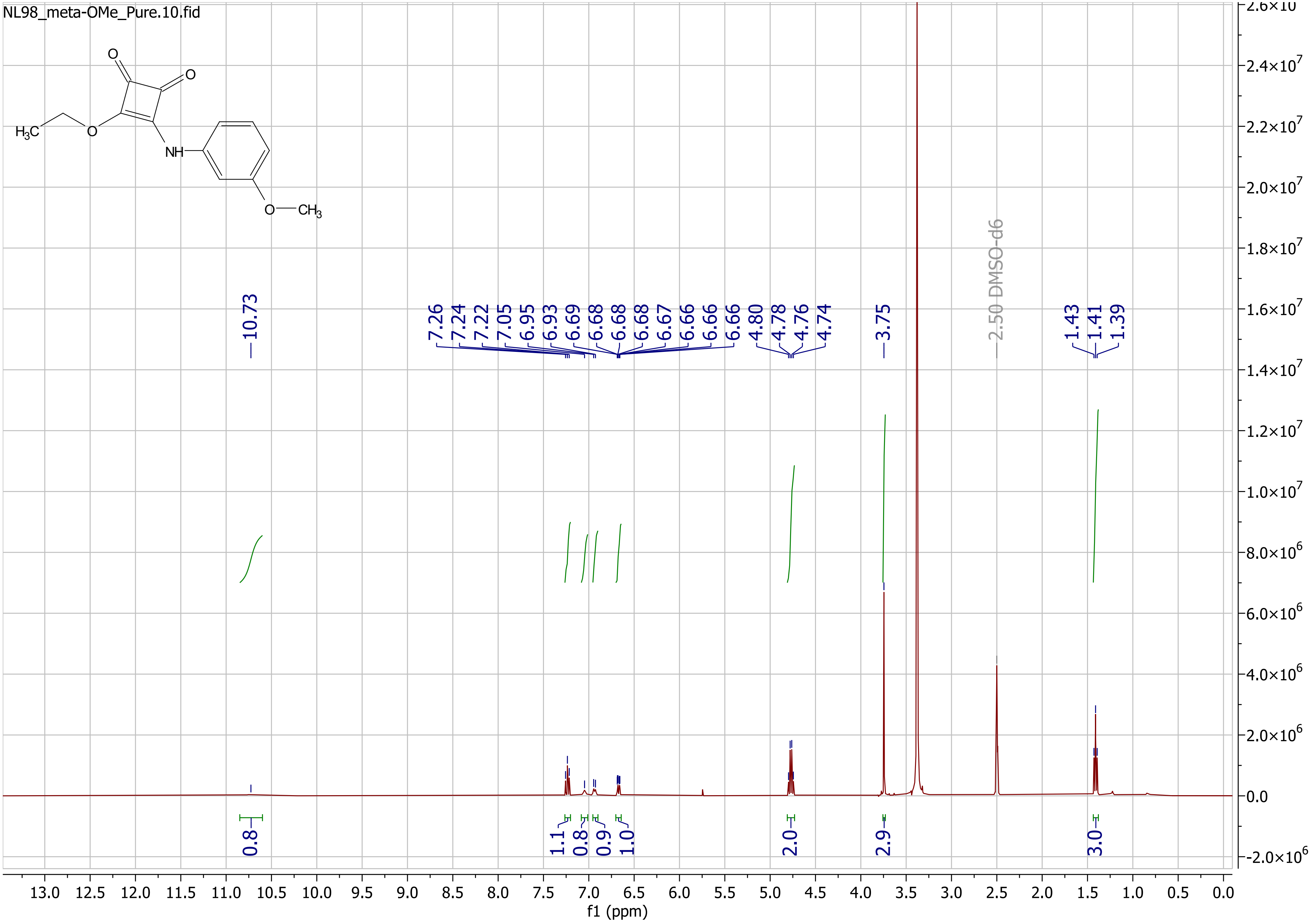

NL98_meta-OMe_Pure.10.fid
O
O
H3C
O
NH
O
CH3
10.73
7.26
7.24
7.22
7.05
6.95
6.93
6.69
6.68
6.68
6.68
6.67
6.66
6.66
6.66
4.80
4.78
4.76
4.74
3.75
2.50 DMSO-d6
1.43
1.41
1.39
0.8
1.1
0.8
0.9
1.0
2.0
2.9
3.0
f1 (ppm)

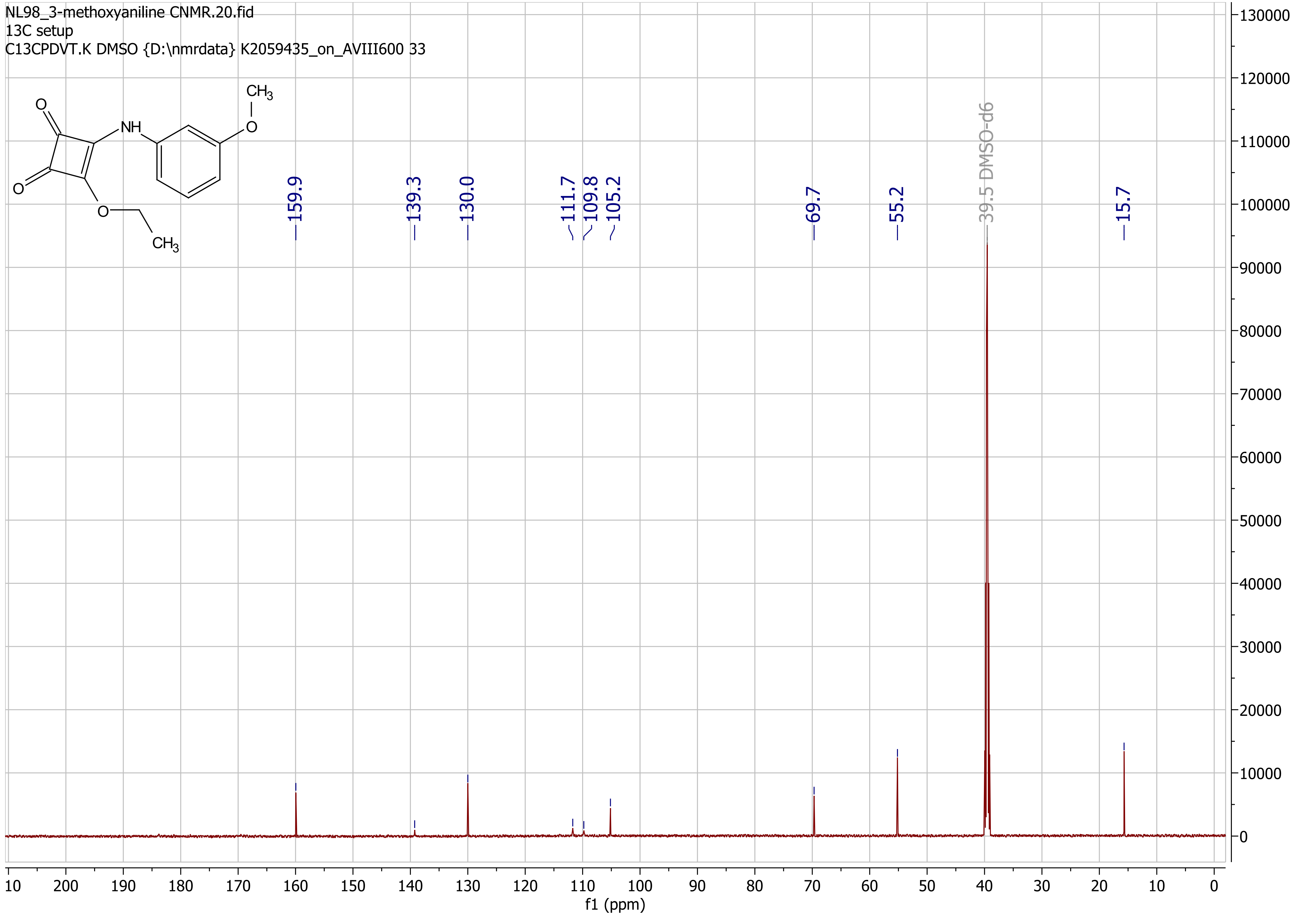
NL98_3-methoxyaniline CNMR.20.fid
13C setup
C13CPDVT.K DMSO {D:\nmrdata} K2059435_on_AVIII600 33
O
O
NH
O
CH3
O
CH3
159.9
139.3
130.0
111.7
109.8
105.2
69.7
55.2
39.5 DMSO-d6
15.7
130000
120000
110000
100000
90000
80000
70000
60000
50000
40000
30000
20000
10000
0
10
200
190
180
170
160
150
140
130
120
110
100
90
80
70
60
50
40
30
20
10
0
f1 (ppm)

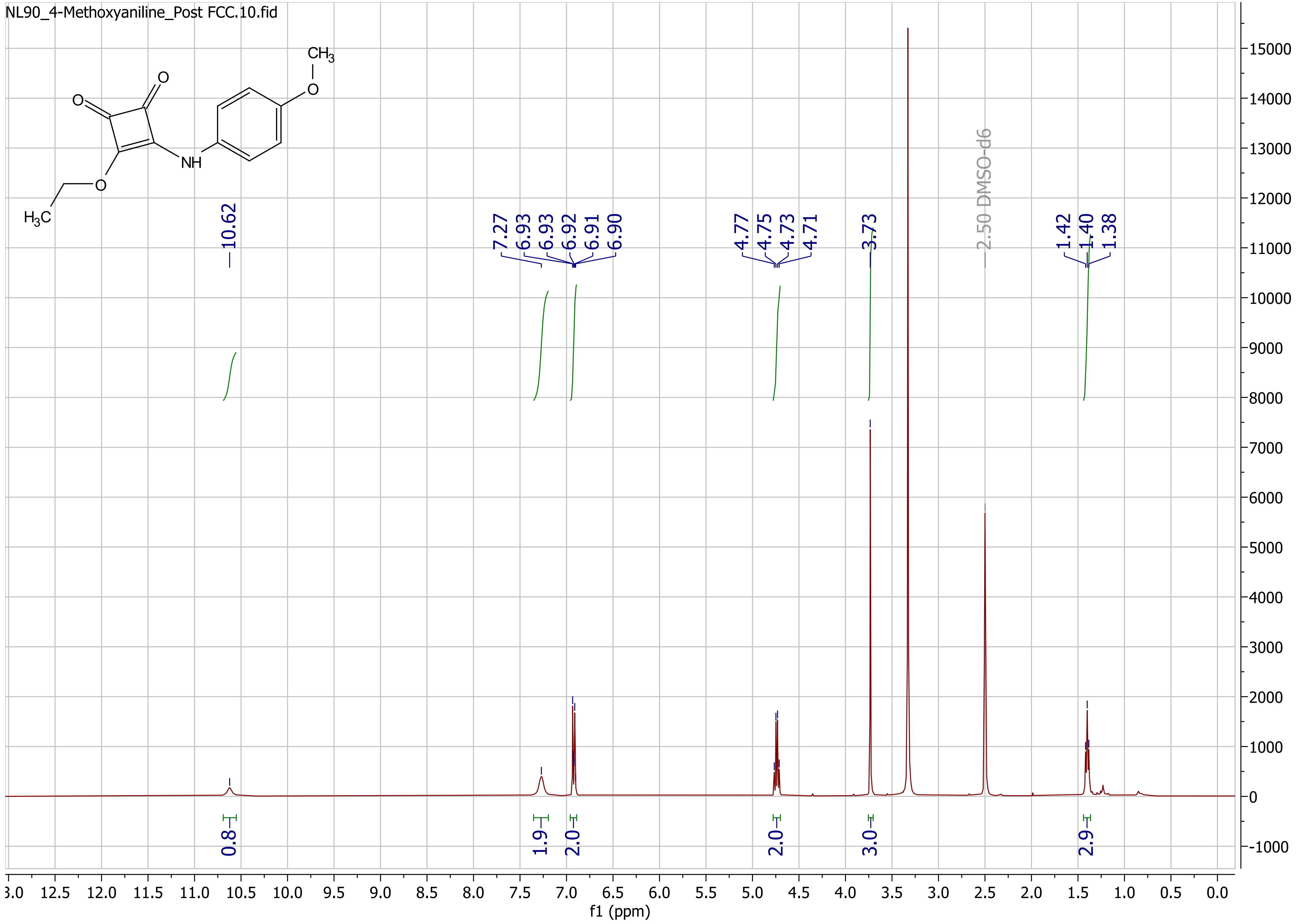

NL90_4-Methoxyaniline_Post FCC.10.fid
CH3
O
O
O
NH
O
H3C
10.62
7.27
6.93
6.93
6.92
6.91
6.90
4.77
4.75
4.73
4.71
3.73
2.50 DMSO-d6
1.42
1.40
1.38
0.8
1.9
2.0
2.0
3.0
2.9
f1 (ppm)

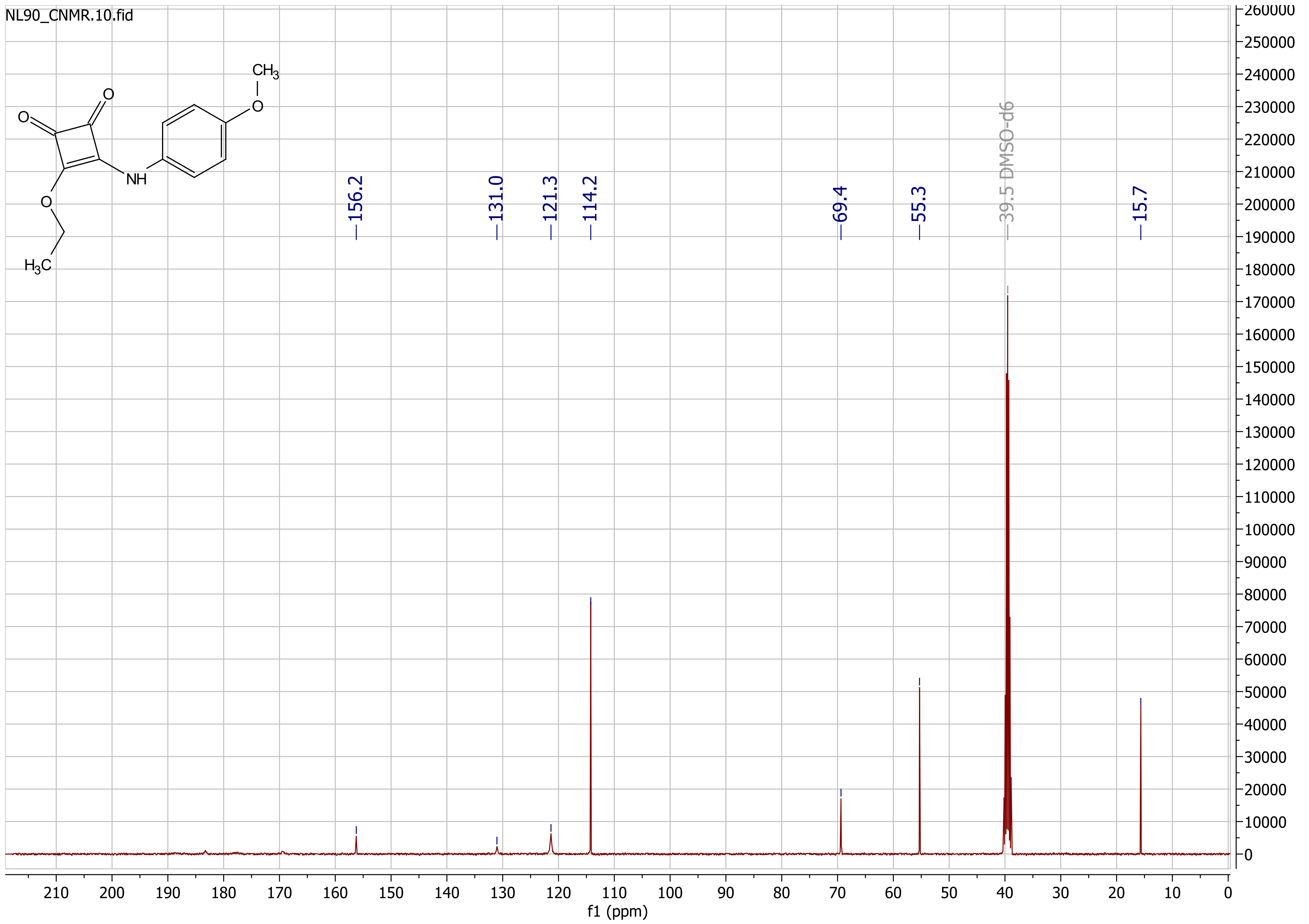
NL90_CNMR.10.fid
CH3
O
O
O
NH
O
H3C
156.2
131.0
121.3
114.2
69.4
55.3
39.5 DMSO-d6
15.7
f1 (ppm)

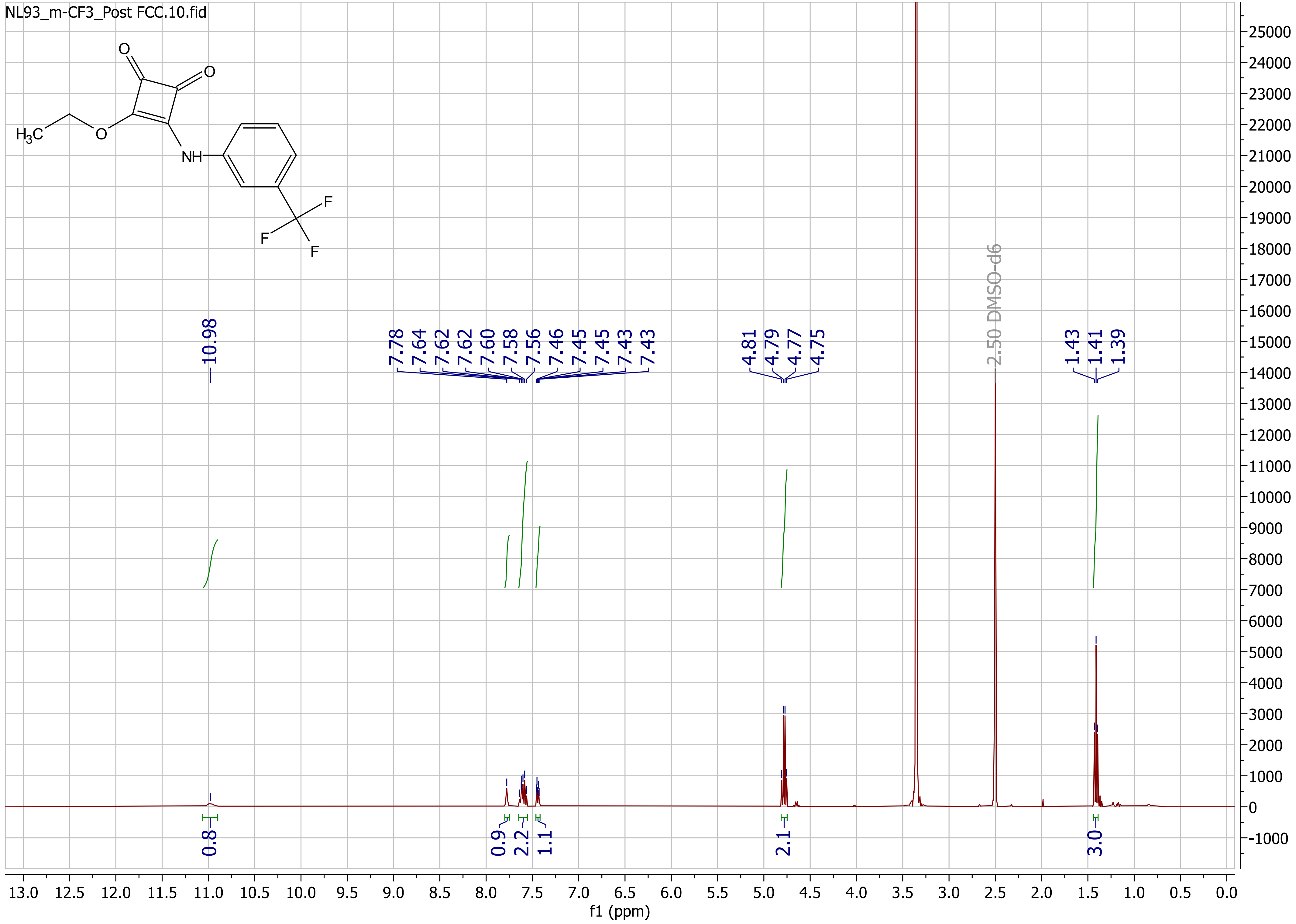
NL93_m-CF3_Post FCC.10.fid
O
O
H3C
O
NH
F
F
F
10.98
7.78
7.64
7.62
7.62
7.60
7.58
7.56
7.46
7.45
7.45
7.43
7.43
4.81
4.79
4.77
4.75
2.50 DMSO-d6
1.43
1.41
1.39
0.8
0.9
2.2
1.1
2.1
3.0
f1 (ppm)

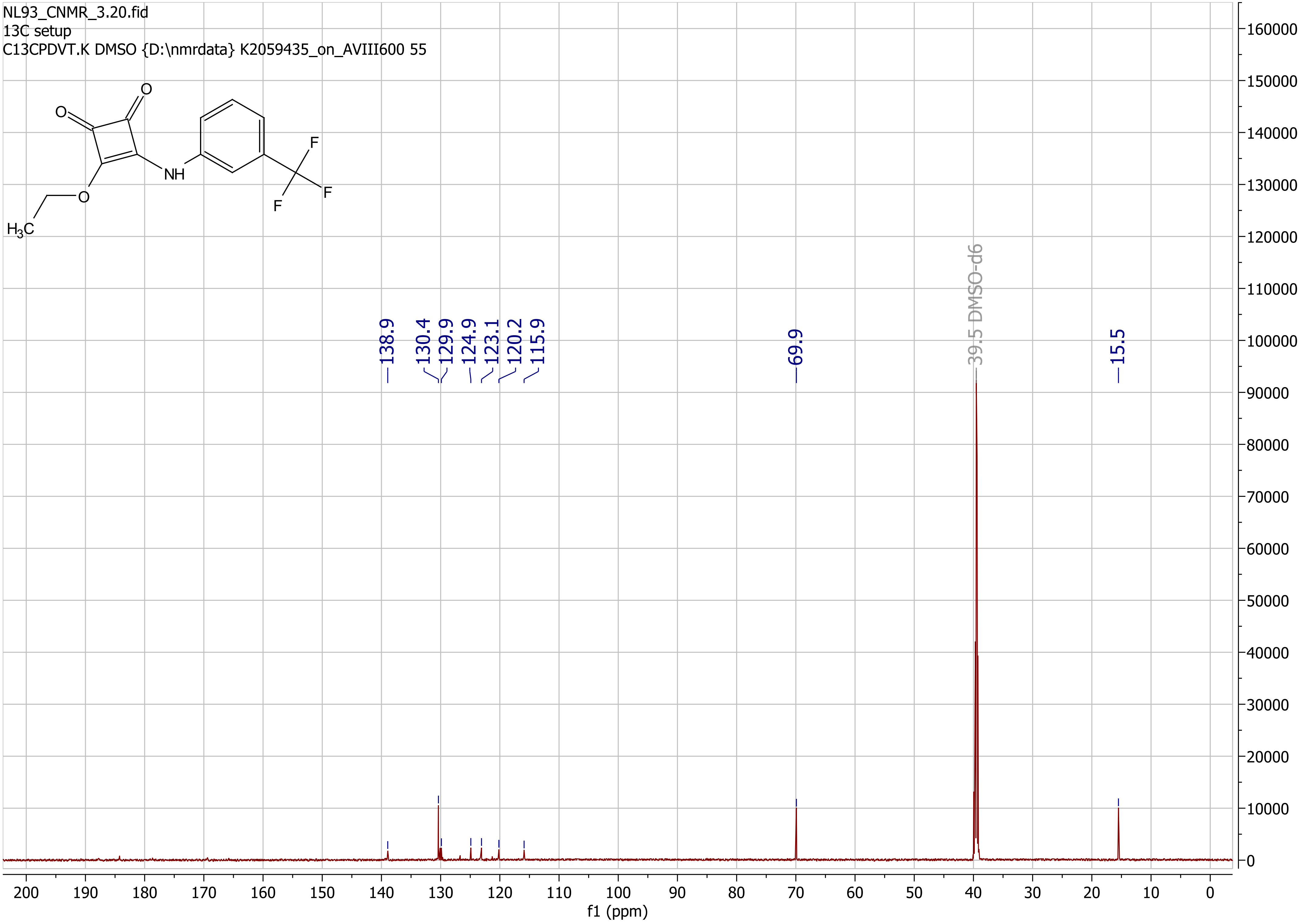
NL93_CNMR_3.20.fid
13C setup
C13CPDVT.K DMSO {D:\nmrdata} K2059435_on_AVIII600 55
O
O
NH
F
F
F
O
H_3C
138.9
130.4
129.9
124.9
123.1
120.2
115.9
69.9
39.5 DMSO-d6
15.5
f1 (ppm)

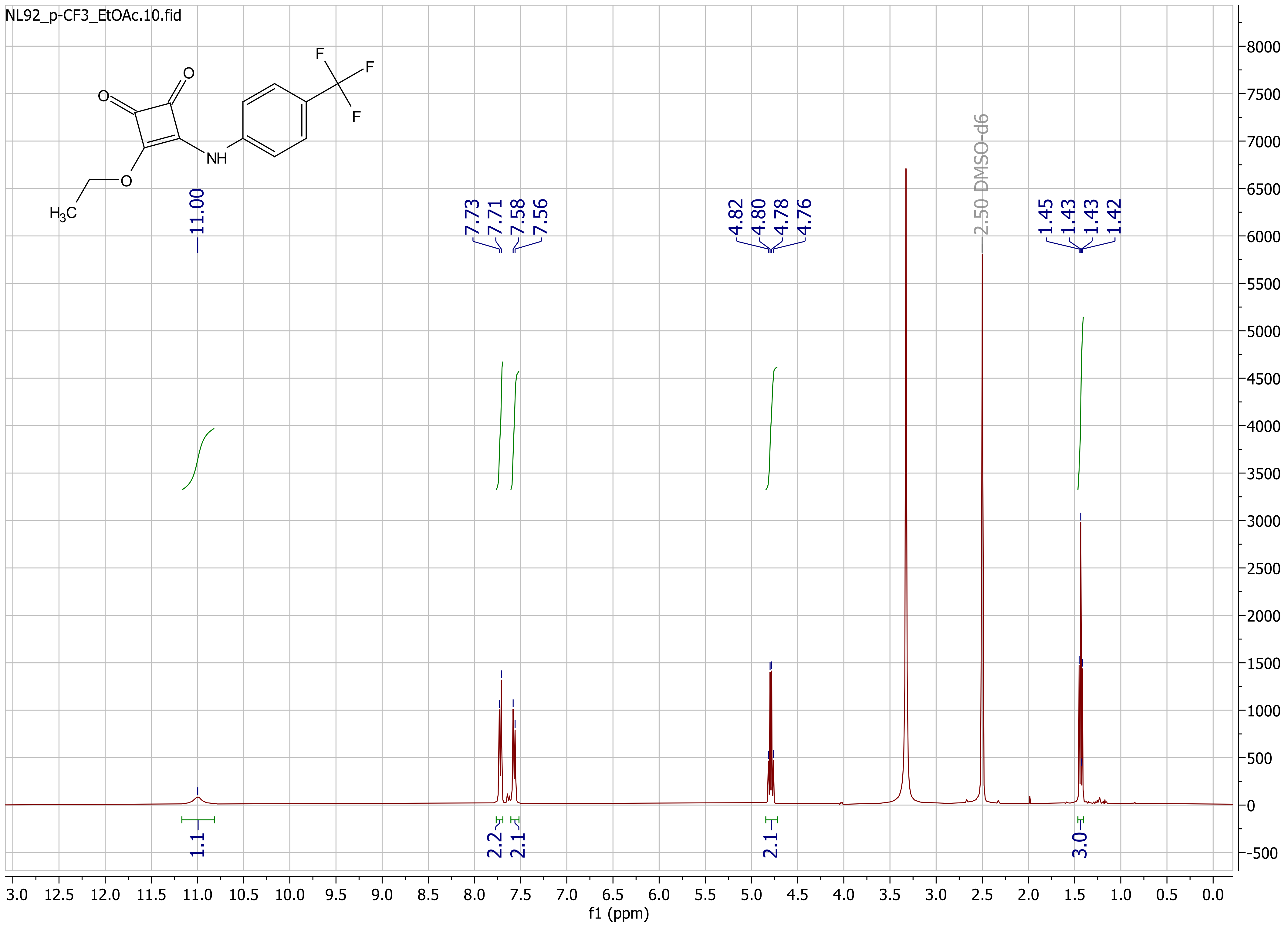
NL92_p-CF3_EtOAc.10.fid
F
F
F
O
O
NH
O
H_3C
11.00
7.73
7.71
7.58
7.56
4.82
4.80
4.78
4.76
2.50 DMSO-d6
1.45
1.43
1.43
1.42
1.1
2.2
2.1
2.1
3.0
f1 (ppm)

NL92_CNMR_2.10.fid
13C setup
C13CPDVT.K DMSO {D:\nmrdata} K2059435_on_AVIII600 54

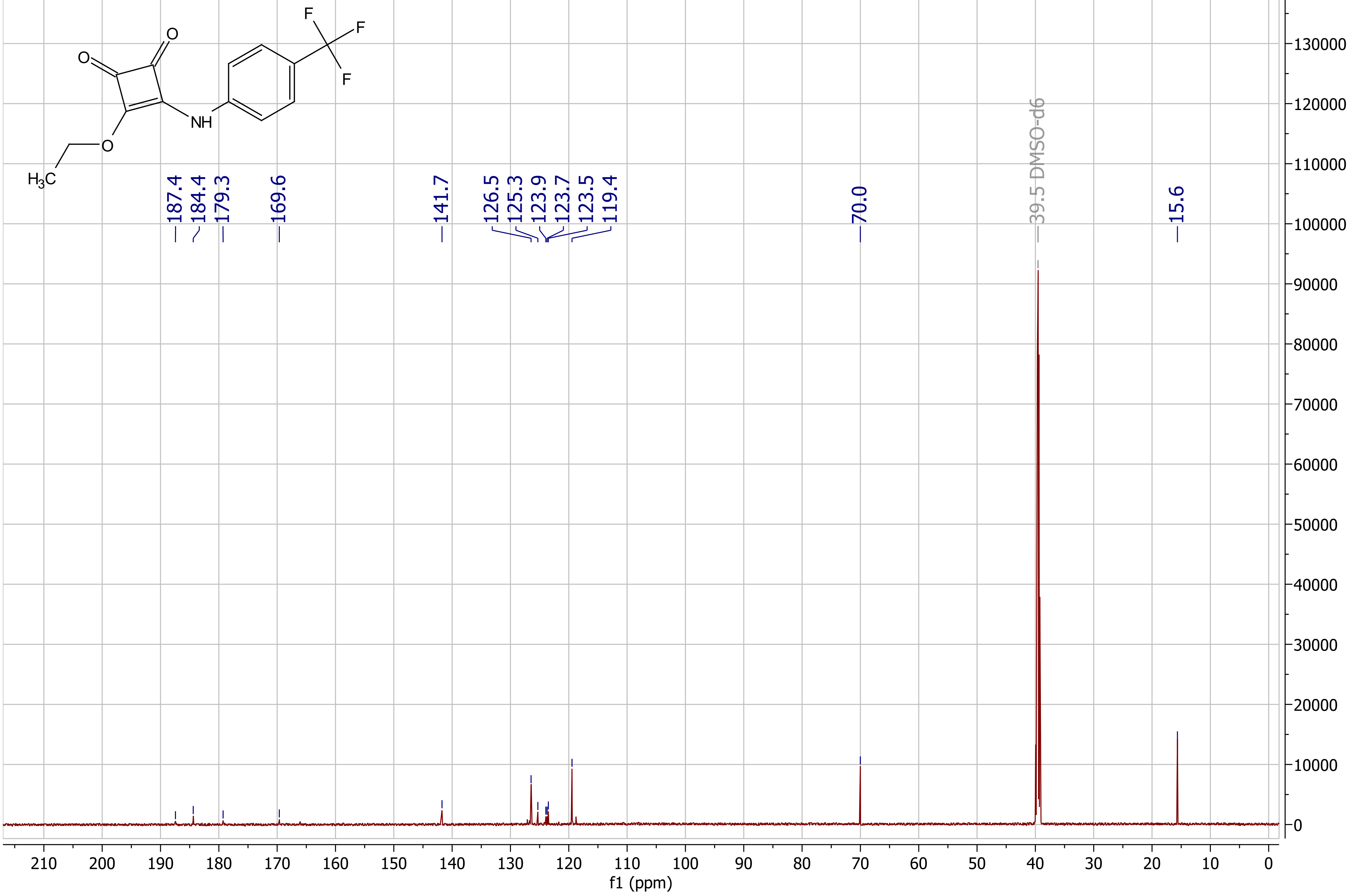

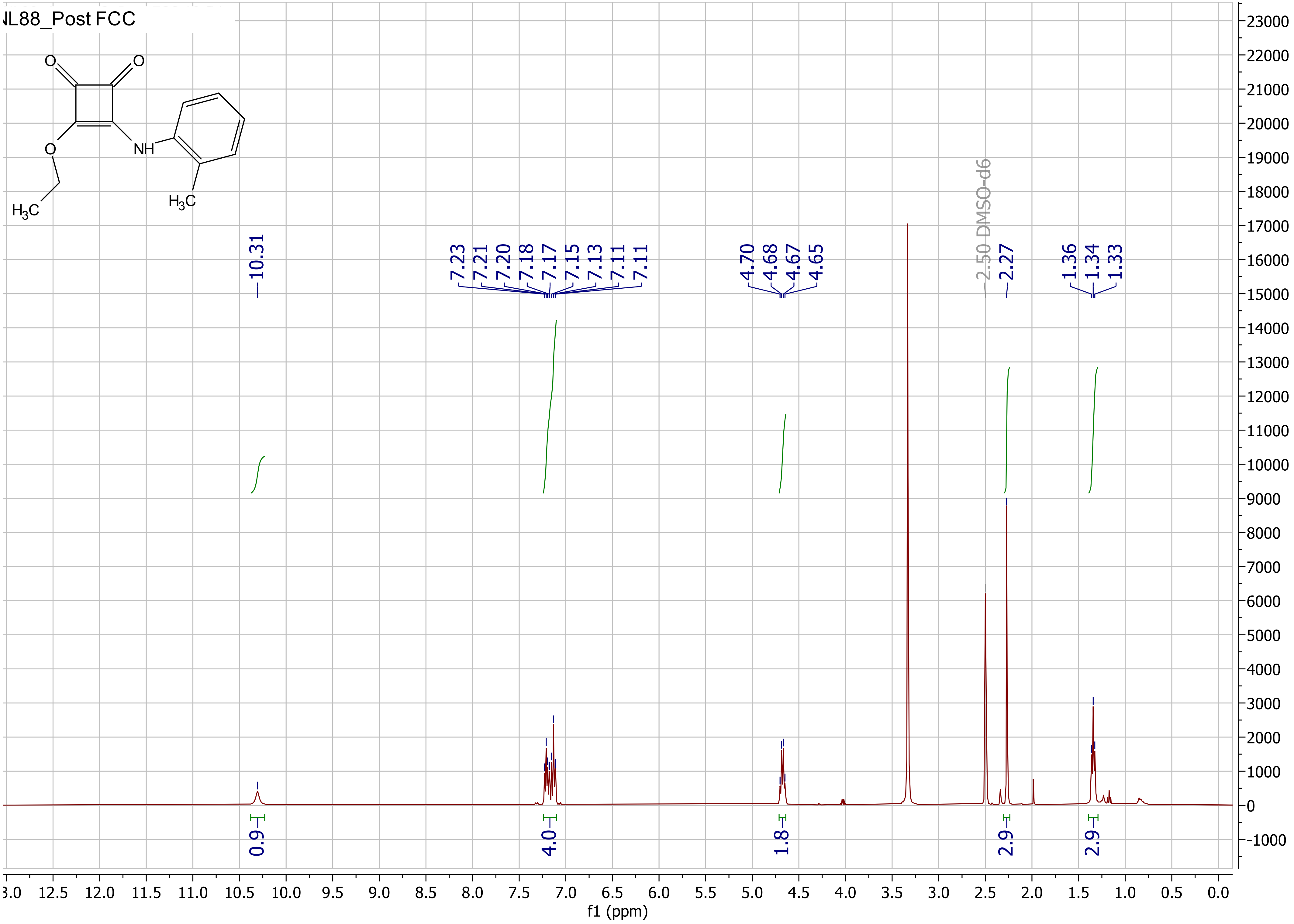

NL88_Post FCC
O
O
O
NH
H3C
H3C
10.31
7.23
7.21
7.20
7.18
7.17
7.15
7.13
7.11
7.11
4.70
4.68
4.67
4.65
2.50 DMSO-d6
2.27
1.36
1.34
1.33
0.9
4.0
1.8
2.9
2.9
13.0 12.5 12.0 11.5 11.0 10.5 10.0 9.5 9.0 8.5 8.0 7.5 7.0 6.5 6.0 5.5 5.0 4.5 4.0 3.5 3.0 2.5 2.0 1.5 1.0 0.5 0.0
f1 (ppm)
23000 22000 21000 20000 19000 18000 17000 16000 15000 14000 13000 12000 11000 10000 9000 8000 7000 6000 5000 4000 3000 2000 1000 0 -1000

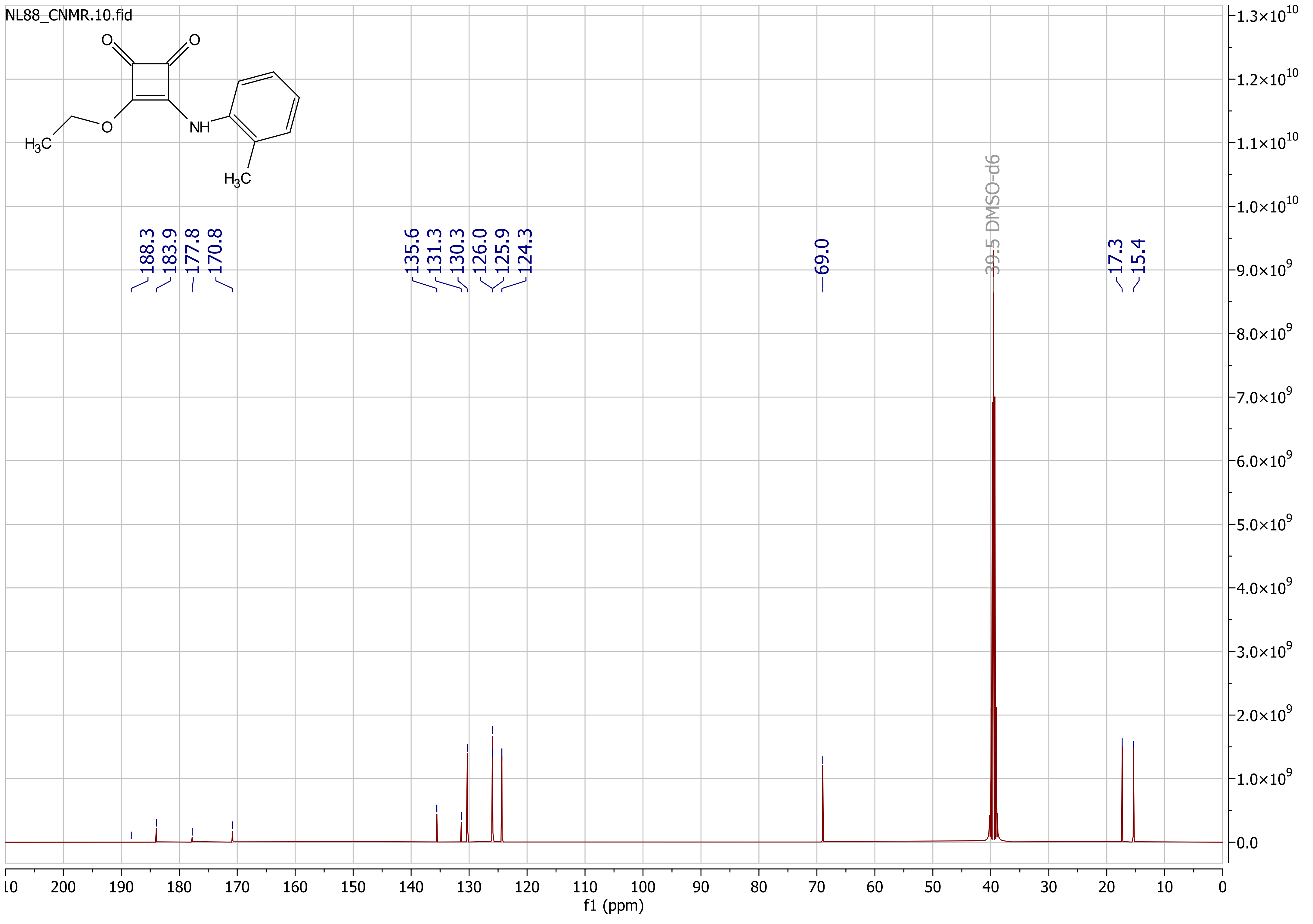

NL88_CNMR.10.fid
O
O
H3C
O
NH
H3C
188.3
183.9
177.8
170.8
135.6
131.3
130.3
126.0
125.9
124.3
69.0
39.5 DMSO-d6
17.3
15.4
f1 (ppm)

NL87 - M-Toluidine Purified

PROTON.K DMSO {D:\nmrdata} K2059435_on_AVIII600 52

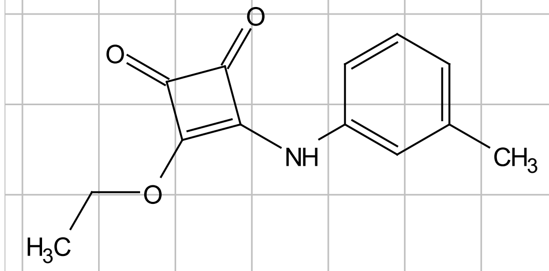

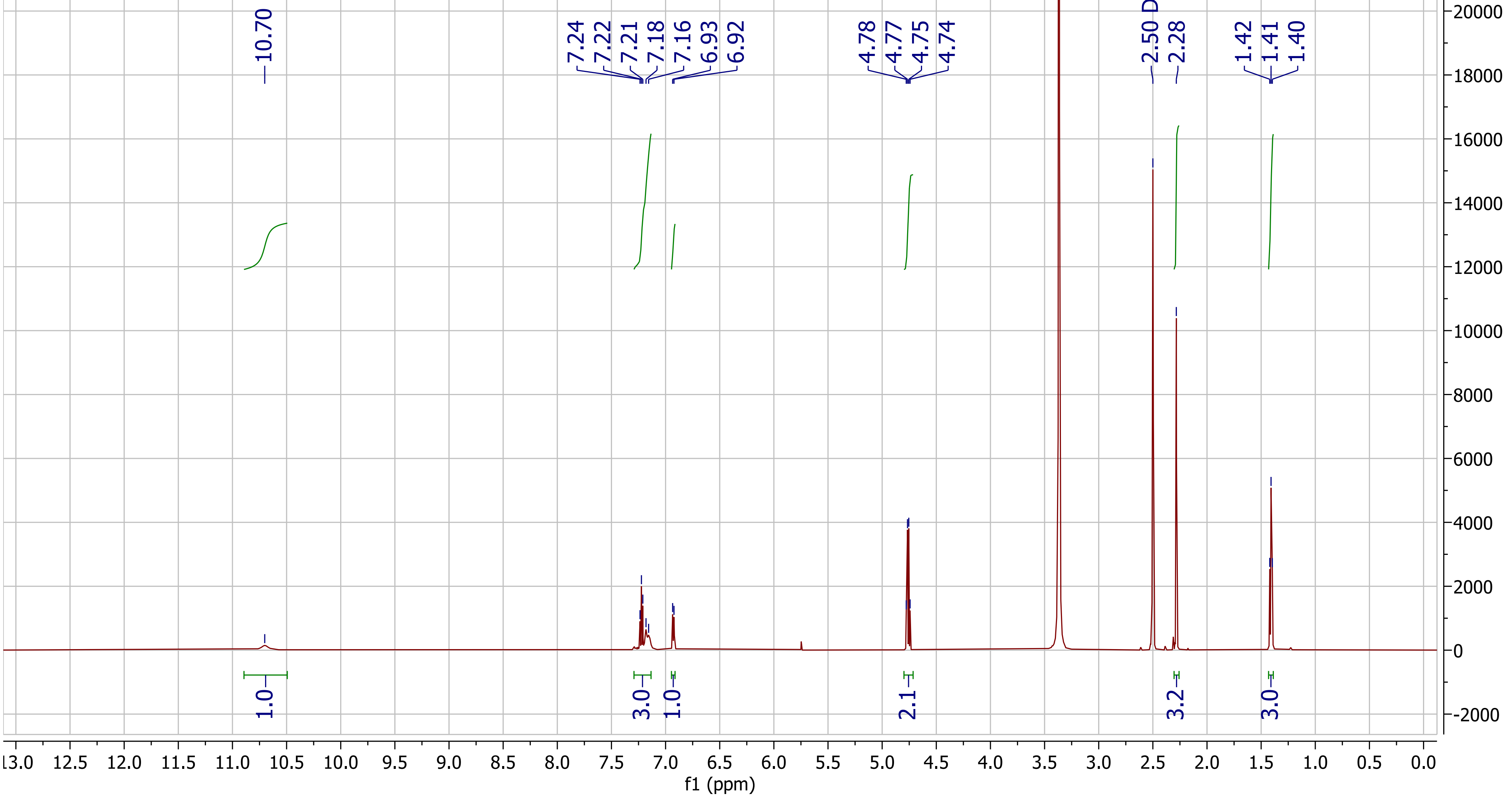

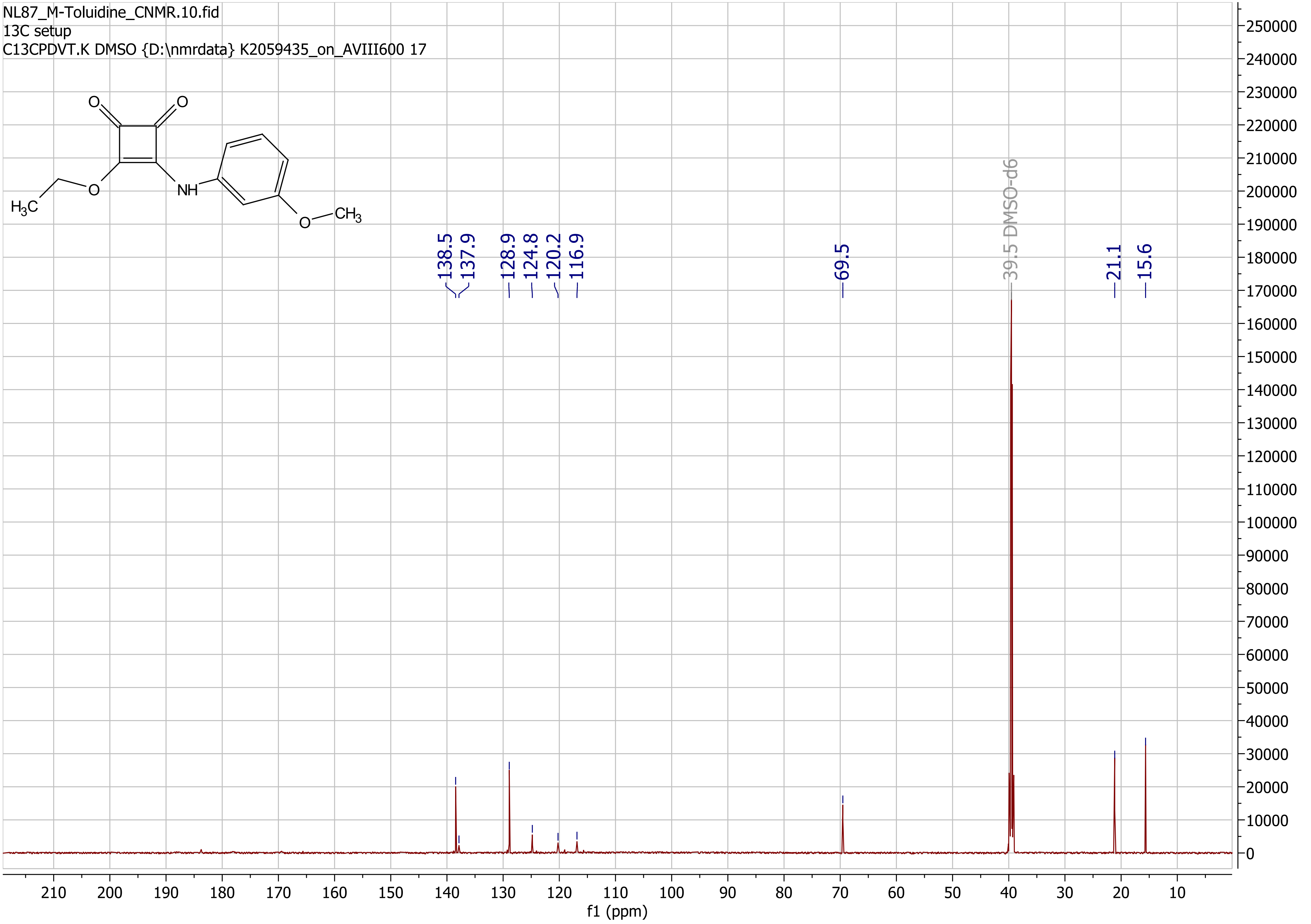
NL87_M-Toluidine_CNMR.10.fid
13C setup
C13CPDVT.K DMSO {D:\nmrdata} K2059435_on_AVIII600 17
O
O
NH
O
H_3C
O
CH_3
138.5
137.9
128.9
124.8
120.2
116.9
69.5
39.5 DMSO-d6
21.1
15.6
f1 (ppm)

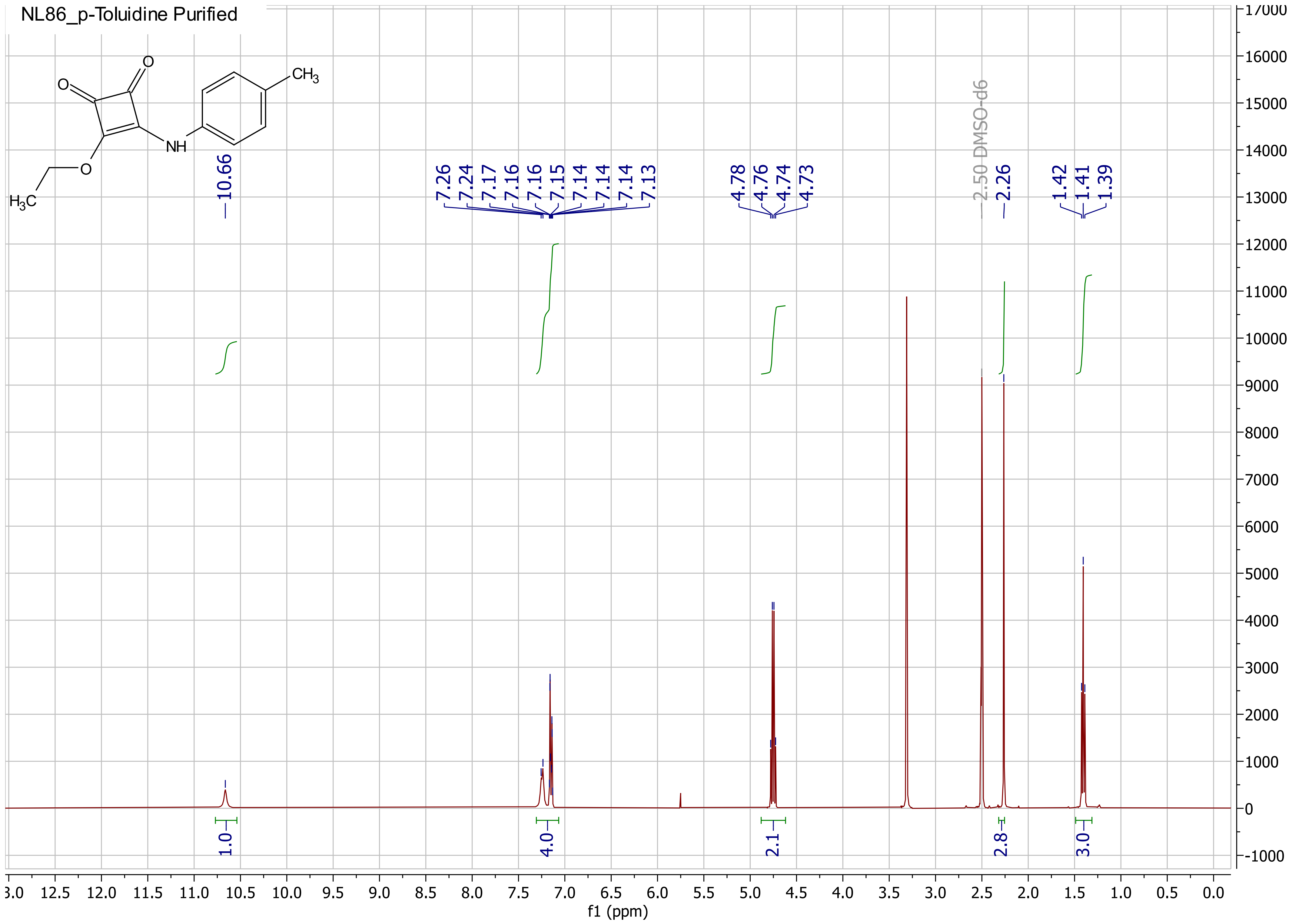

NL86_p-Toluidine Purified
O
O
CH3
NH
O
H3C
10.66
7.26
7.24
7.17
7.16
7.16
7.15
7.14
7.14
7.14
7.13
4.78
4.76
4.74
4.73
2.50 DMSO-d6
2.26
1.42
1.41
1.39
1.0
4.0
2.1
2.8
3.0
17000
16000
15000
14000
13000
12000
11000
10000
9000
8000
7000
6000
5000
4000
3000
2000
1000
0
-1000
13.0
12.5
12.0
11.5
11.0
10.5
10.0
9.5
9.0
8.5
8.0
7.5
7.0
6.5
6.0
5.5
5.0
4.5
4.0
3.5
3.0
2.5
2.0
1.5
1.0
0.5
0.0
f1 (ppm)

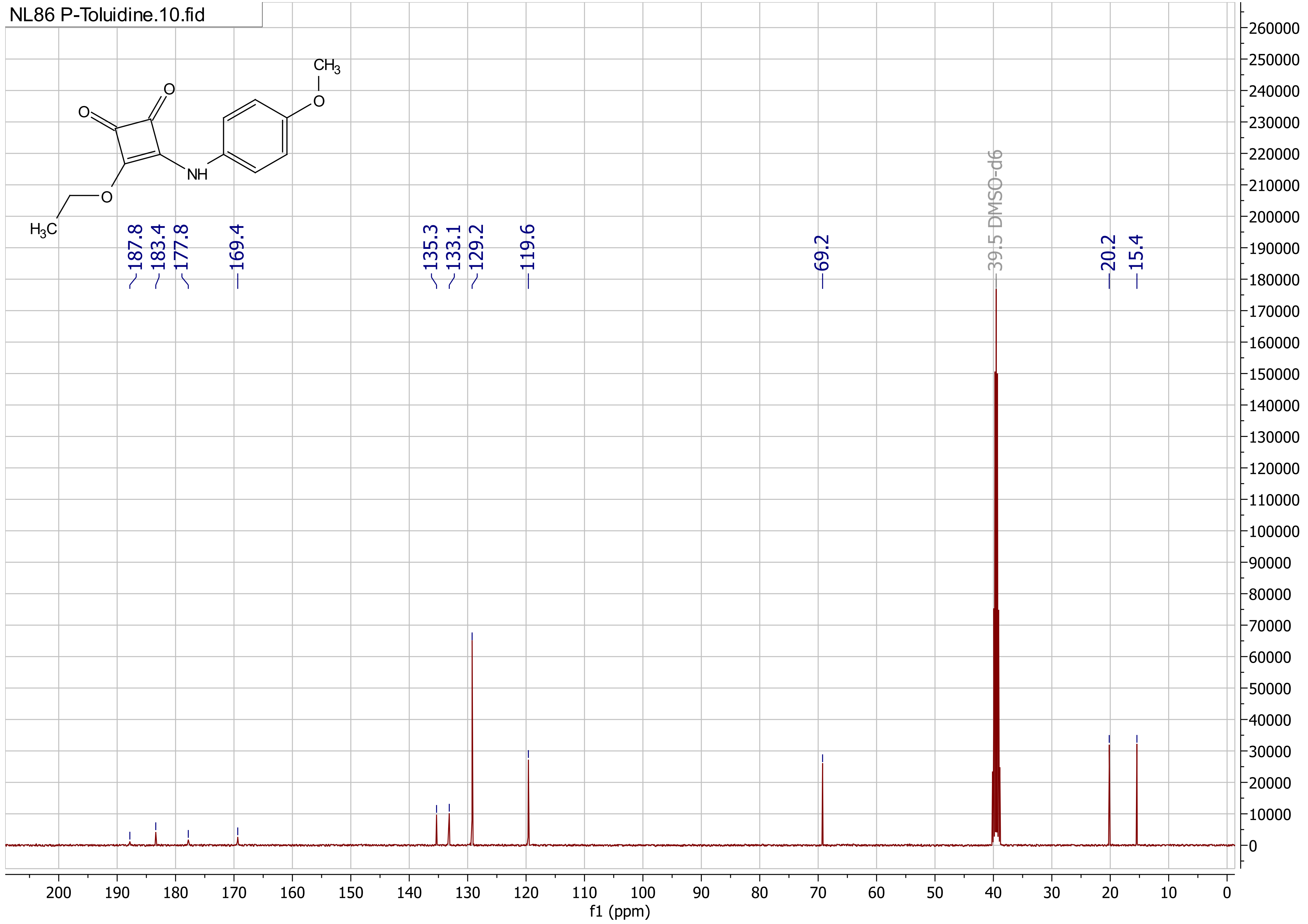
NL86 P-Toluidine.10.fid
CH3
O
O
O
NH
O
H3C
187.8
183.4
177.8
169.4
135.3
133.1
129.2
119.6
69.2
39.5 DMSO-d6
20.2
15.4
f1 (ppm)

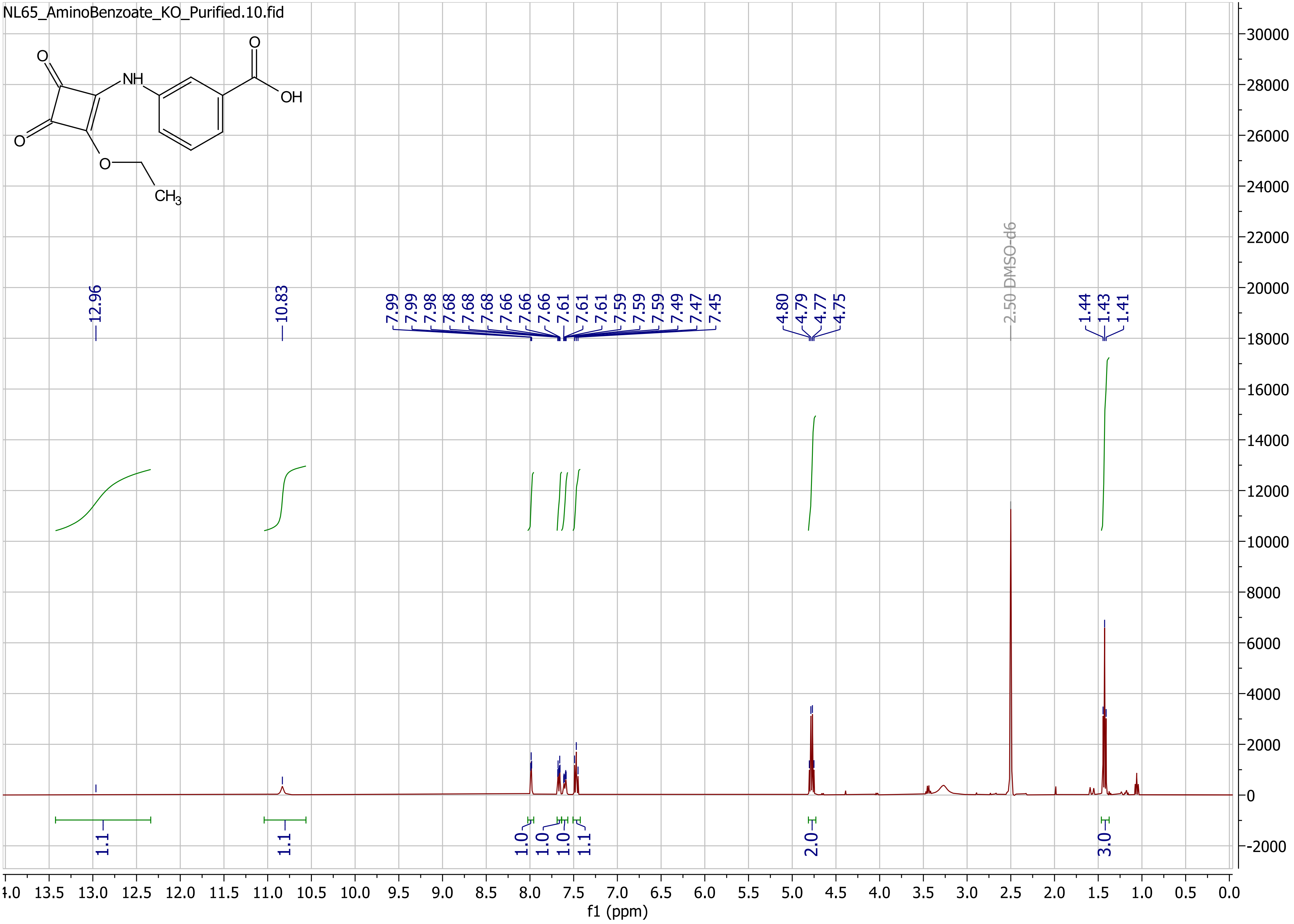
NL65_AminoBenzoate_KO_Purified.10.fid
O
O
NH
O
OH
O
O
CH3
12.96
10.83
7.99
7.99
7.98
7.68
7.68
7.68
7.66
7.66
7.66
7.61
7.61
7.61
7.59
7.59
7.59
7.49
7.47
7.45
4.80
4.79
4.77
4.75
2.50 DMSO-d6
1.44
1.43
1.41
1.1
1.1
1.0
1.0
1.0
1.1
2.0
3.0
f1 (ppm)

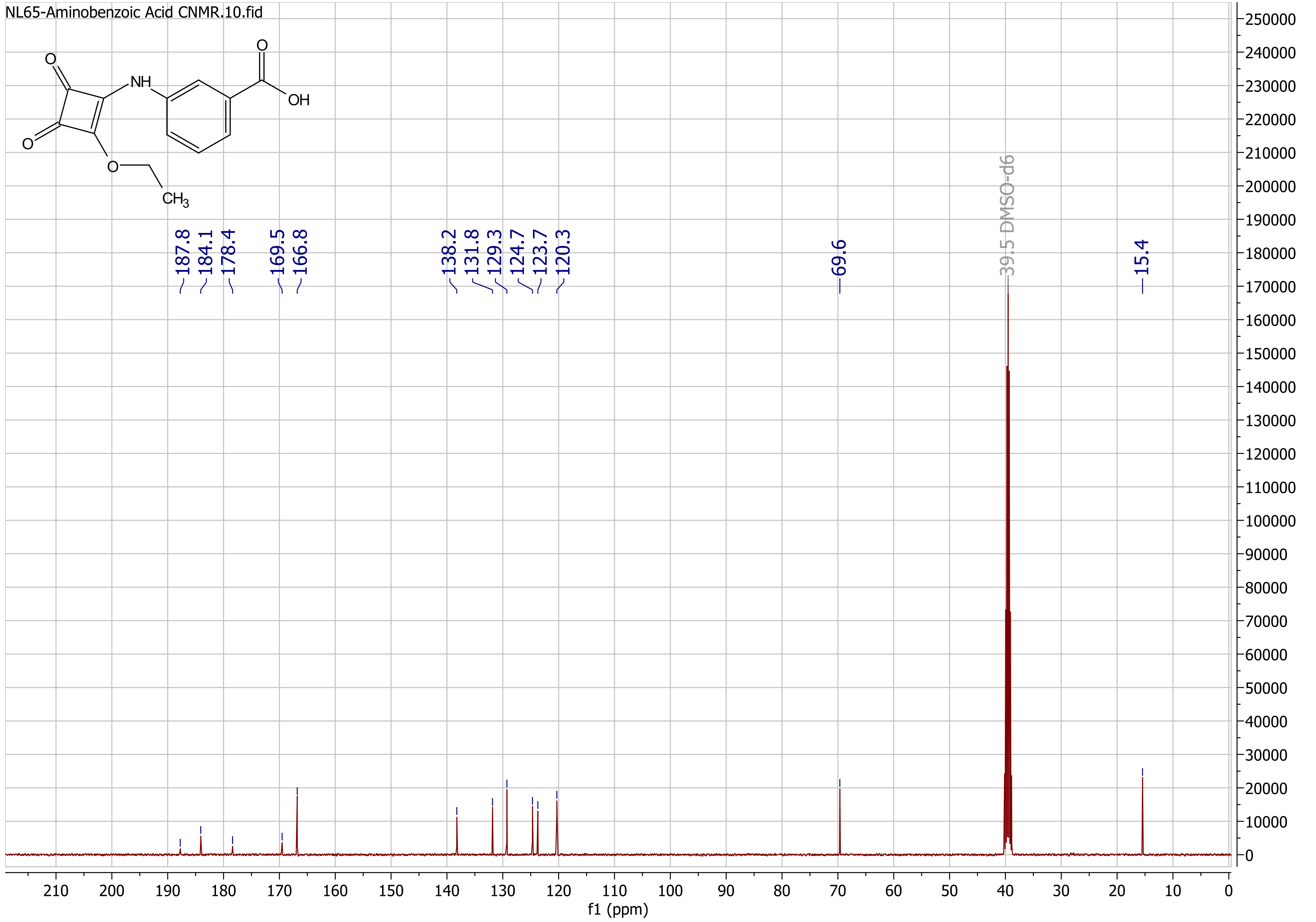

NL65-Aminobenzoic Acid CNMR.10.fid
O
O
NH
O
OH
O
CH3
187.8
184.1
178.4
169.5
166.8
138.2
131.8
129.3
124.7
123.7
120.3
69.6
39.5 DMSO-d6
15.4
f1 (ppm)

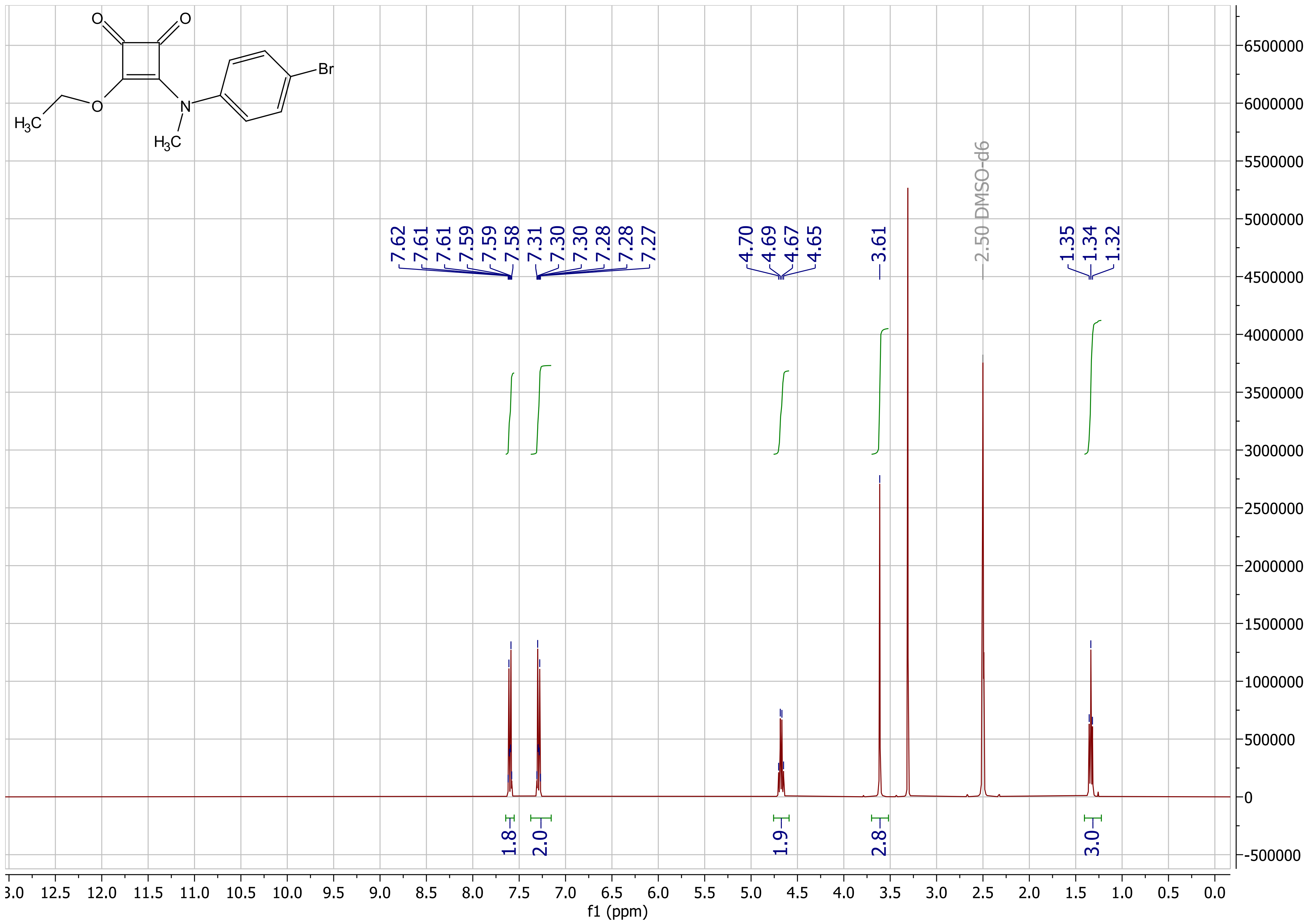

Br
O
O
N
O
H3C
H3C
7.62
7.61
7.61
7.59
7.59
7.58
7.31
7.30
7.30
7.28
7.28
7.27
4.70
4.69
4.67
4.65
3.61
2.50 DMSO-d6
1.35
1.34
1.32
1.8
2.0
1.9
2.8
3.0
f1 (ppm)

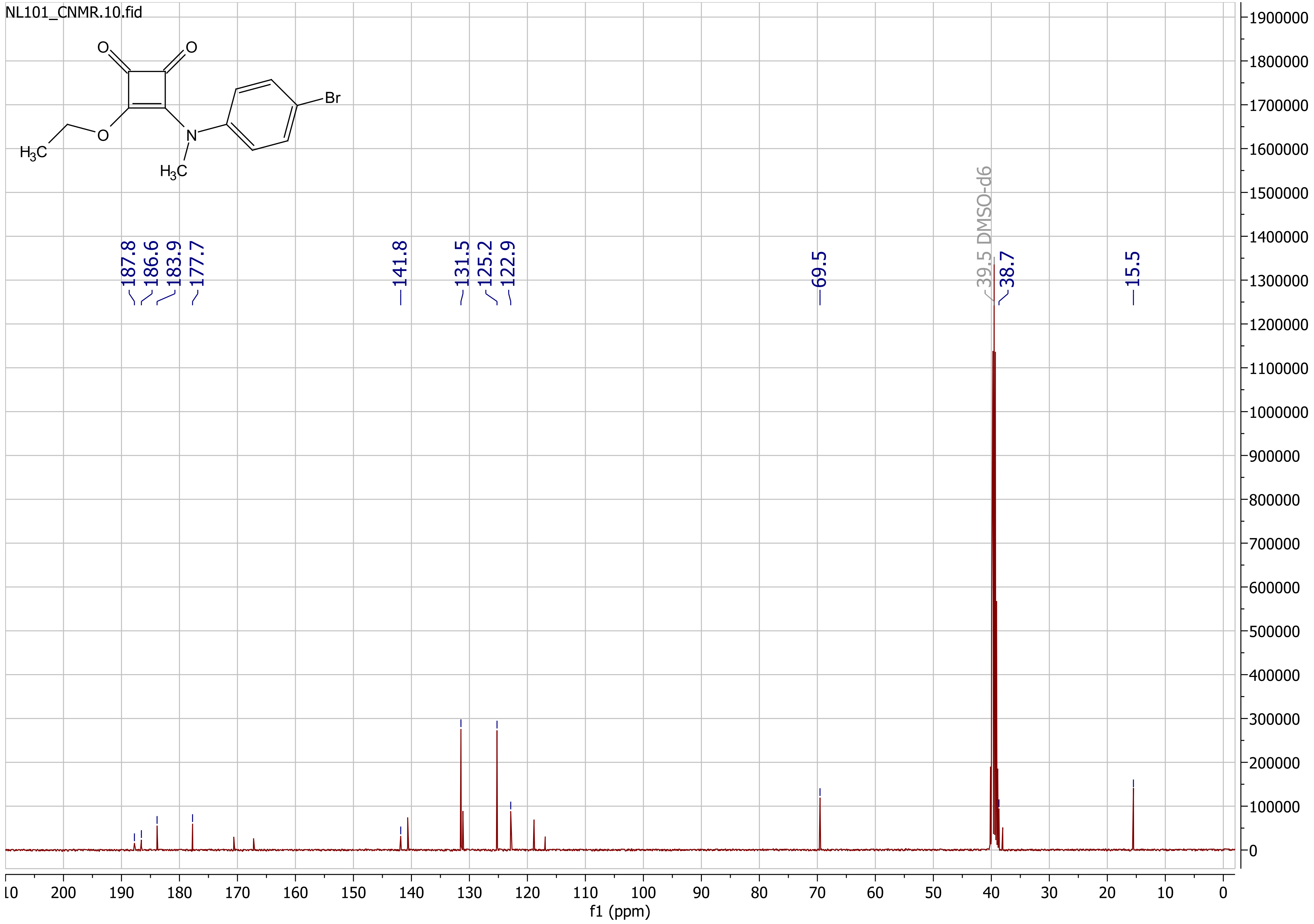

NL101_CNMR.10.fid
O
O
Br
O
N
H_3C
H_3C
187.8
186.6
183.9
177.7
141.8
131.5
125.2
122.9
69.5
39.5 DMSO-d6
38.7
15.5
1900000
1800000
1700000
1600000
1500000
1400000
1300000
1200000
1100000
1000000
900000
800000
700000
600000
500000
400000
300000
200000
100000
0
200
190
180
170
160
150
140
130
120
110
100
90
80
70
60
50
40
30
20
10
0
f1 (ppm)

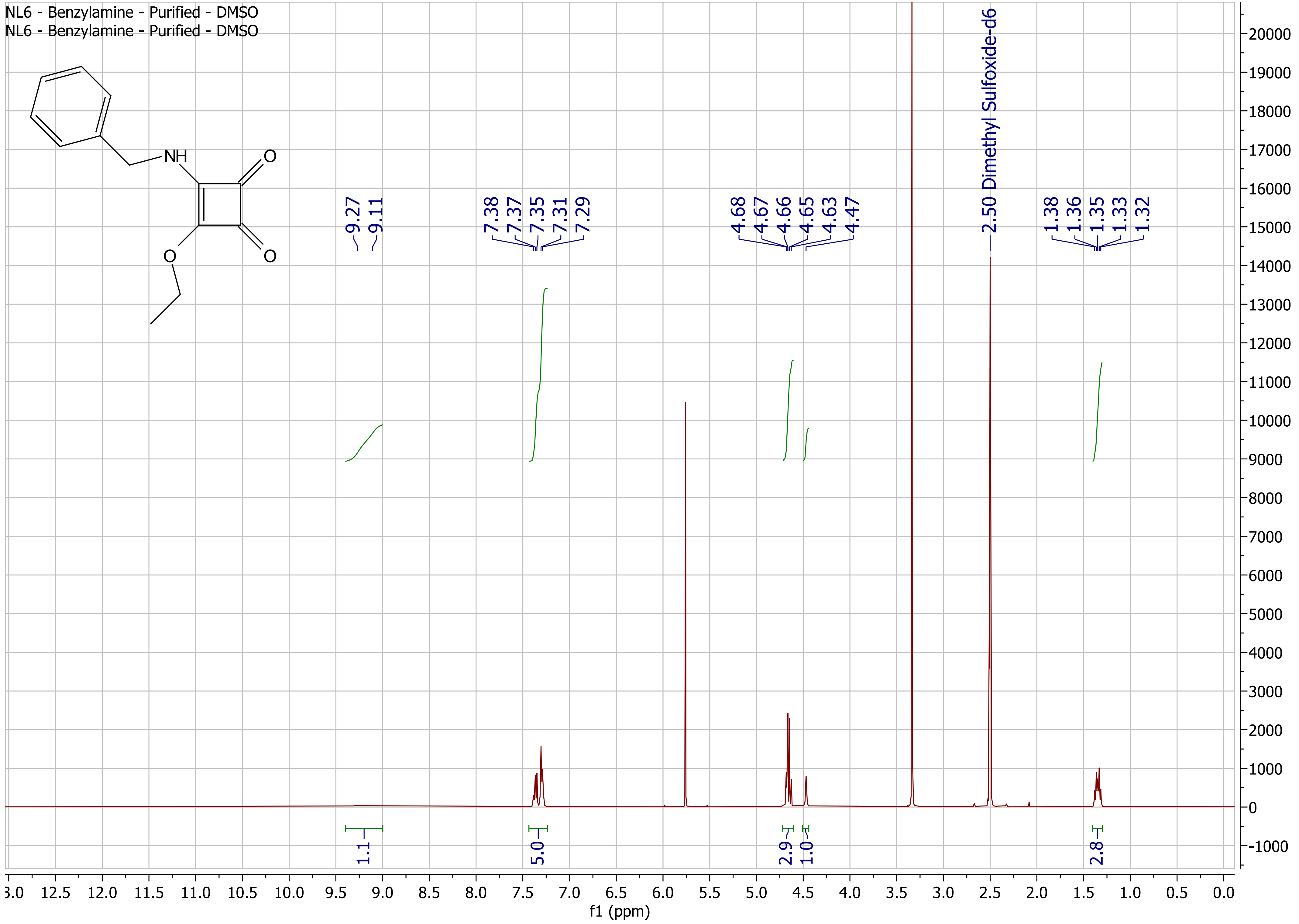
NL6 - Benzylamine - Purified - DMSO
NL6 - Benzylamine - Purified - DMSO
NH
O
O
O
9.27
9.11
7.38
7.37
7.35
7.31
7.29
4.68
4.67
4.66
4.65
4.63
4.47
2.50 Dimethyl Sulfoxide-d6
1.38
1.36
1.35
1.33
1.32
1.1
5.0
2.9
1.0
2.8
f1 (ppm)

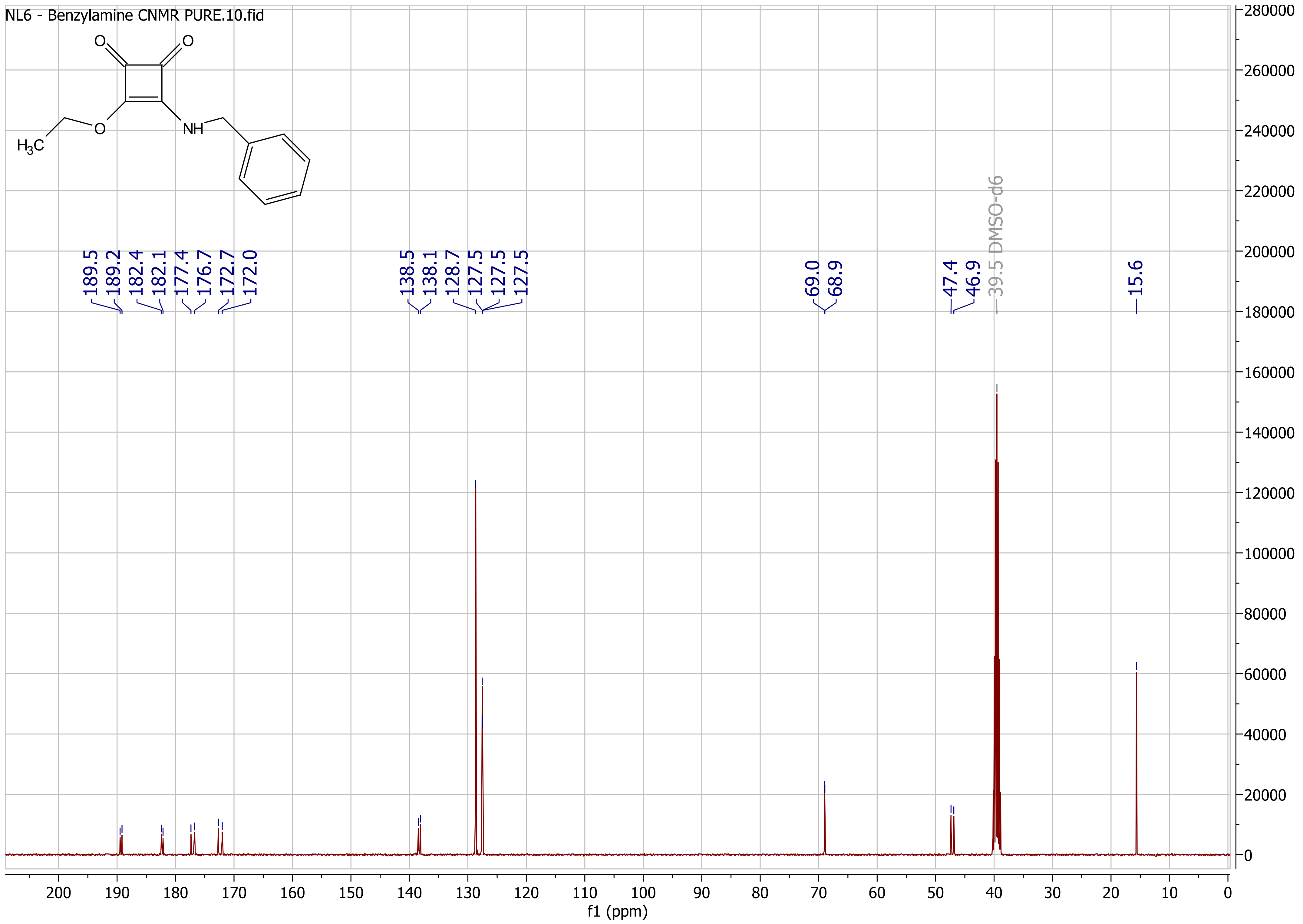

NL6 - Benzylamine CNMR PURE.10.fid
O
O
O
NH
H3C
189.5
189.2
182.4
182.1
177.4
176.7
172.7
172.0
138.5
138.1
128.7
127.5
127.5
127.5
69.0
68.9
47.4
46.9
39.5 DMSO-d6
15.6
280000
260000
240000
220000
200000
180000
160000
140000
120000
100000
80000
60000
40000
20000
0
200
190
180
170
160
150
140
130
120
110
100
90
80
70
60
50
40
30
20
10
0
f1 (ppm)

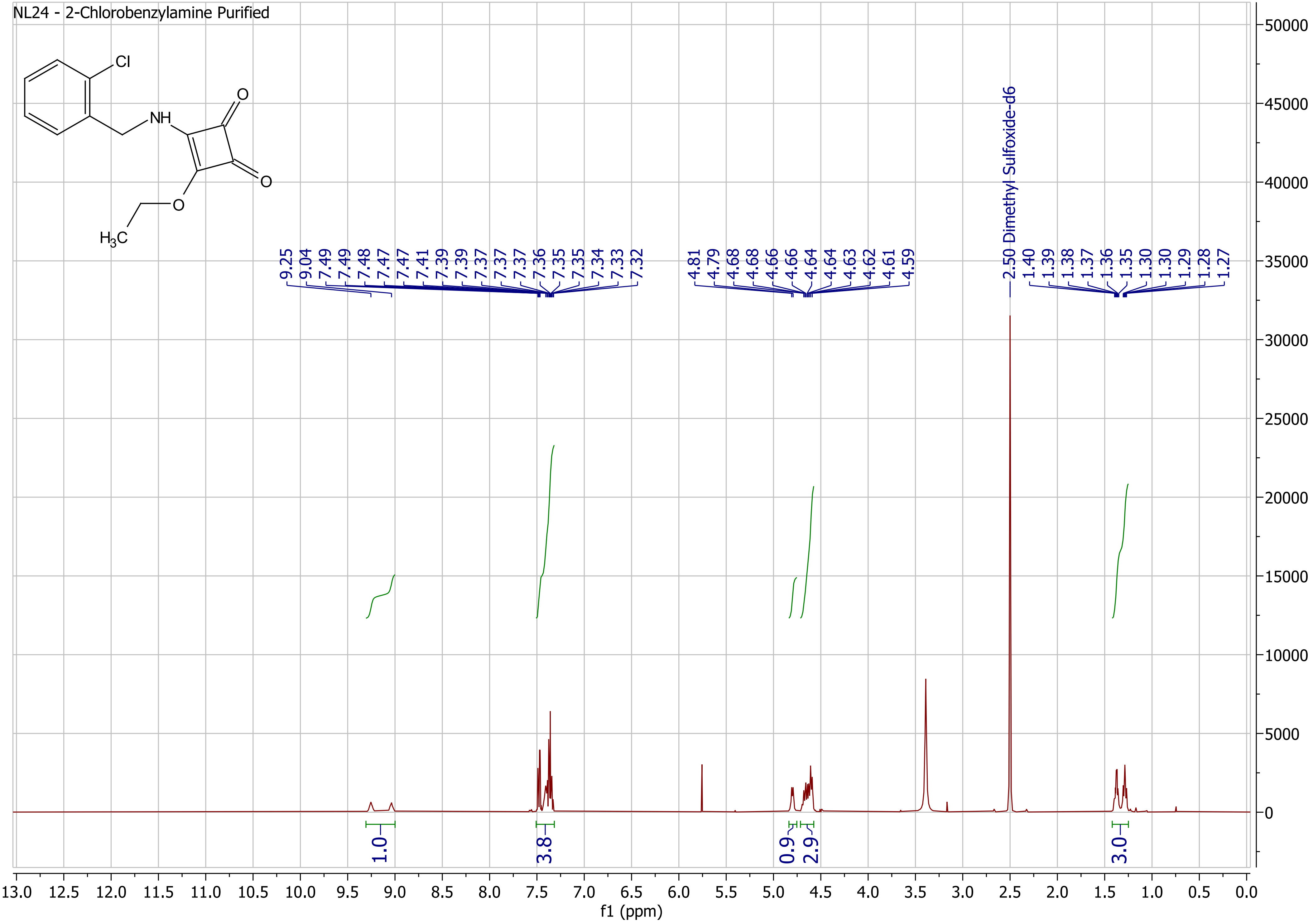
NL24 - 2-Chlorobenzylamine Purified
Cl
NH
O
O
O
H3C
9.25
9.04
7.49
7.49
7.48
7.47
7.47
7.41
7.39
7.39
7.37
7.37
7.37
7.36
7.35
7.35
7.34
7.33
7.32
4.81
4.79
4.68
4.68
4.66
4.66
4.64
4.64
4.63
4.62
4.61
4.59
2.50 Dimethyl Sulfoxide-d6
1.40
1.39
1.38
1.37
1.36
1.35
1.30
1.30
1.29
1.28
1.27
1.0
3.8
0.9
2.9
3.0
50000
45000
40000
35000
30000
25000
20000
15000
10000
5000
0
13.0
12.5
12.0
11.5
11.0
10.5
10.0
9.5
9.0
8.5
8.0
7.5
7.0
6.5
6.0
5.5
5.0
4.5
4.0
3.5
3.0
2.5
2.0
1.5
1.0
0.5
0.0
f1 (ppm)

NL24_CNMR_ACETONE.20.fid

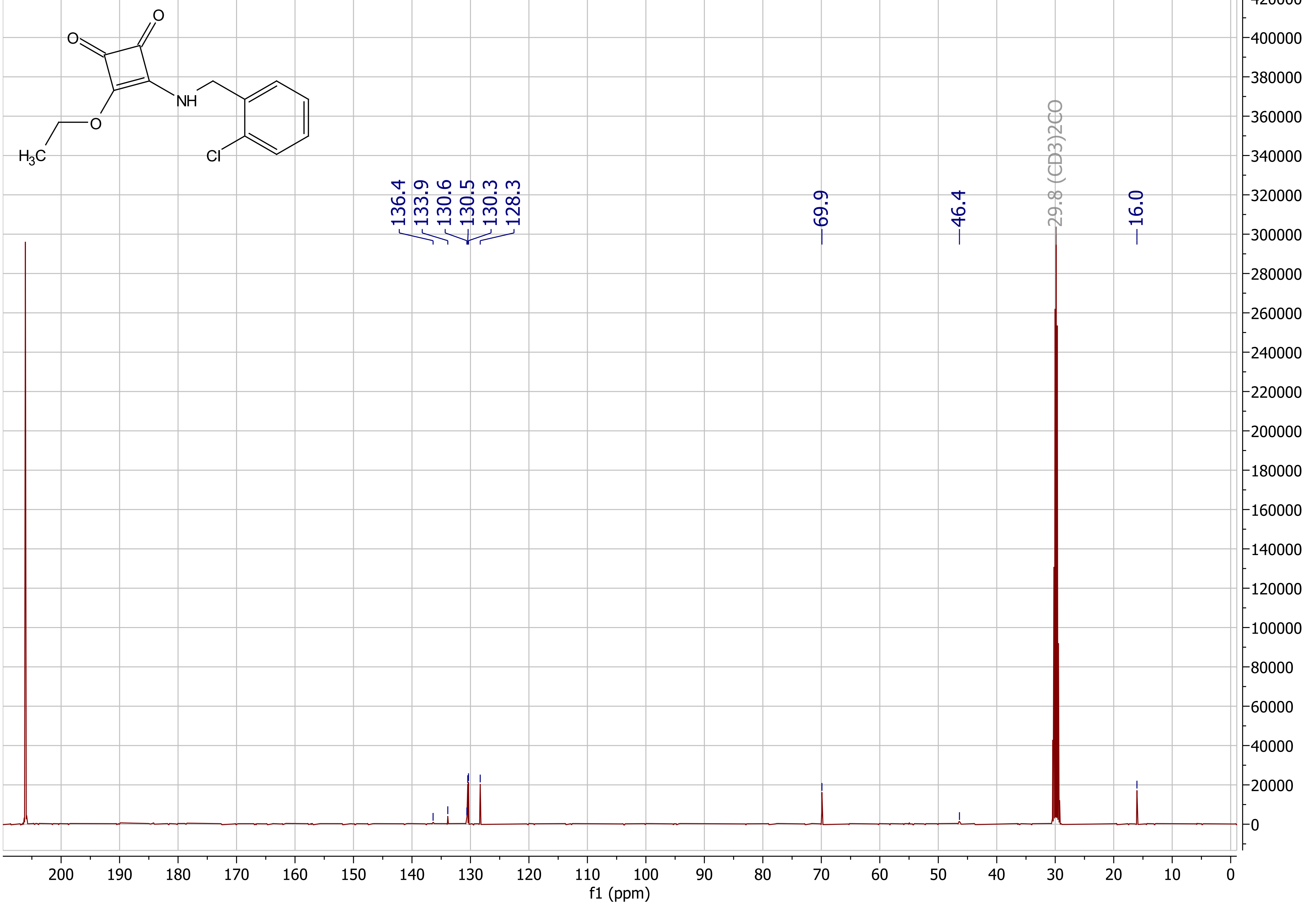

NL50 - 3-chlorobenzylamine purified.10.fid

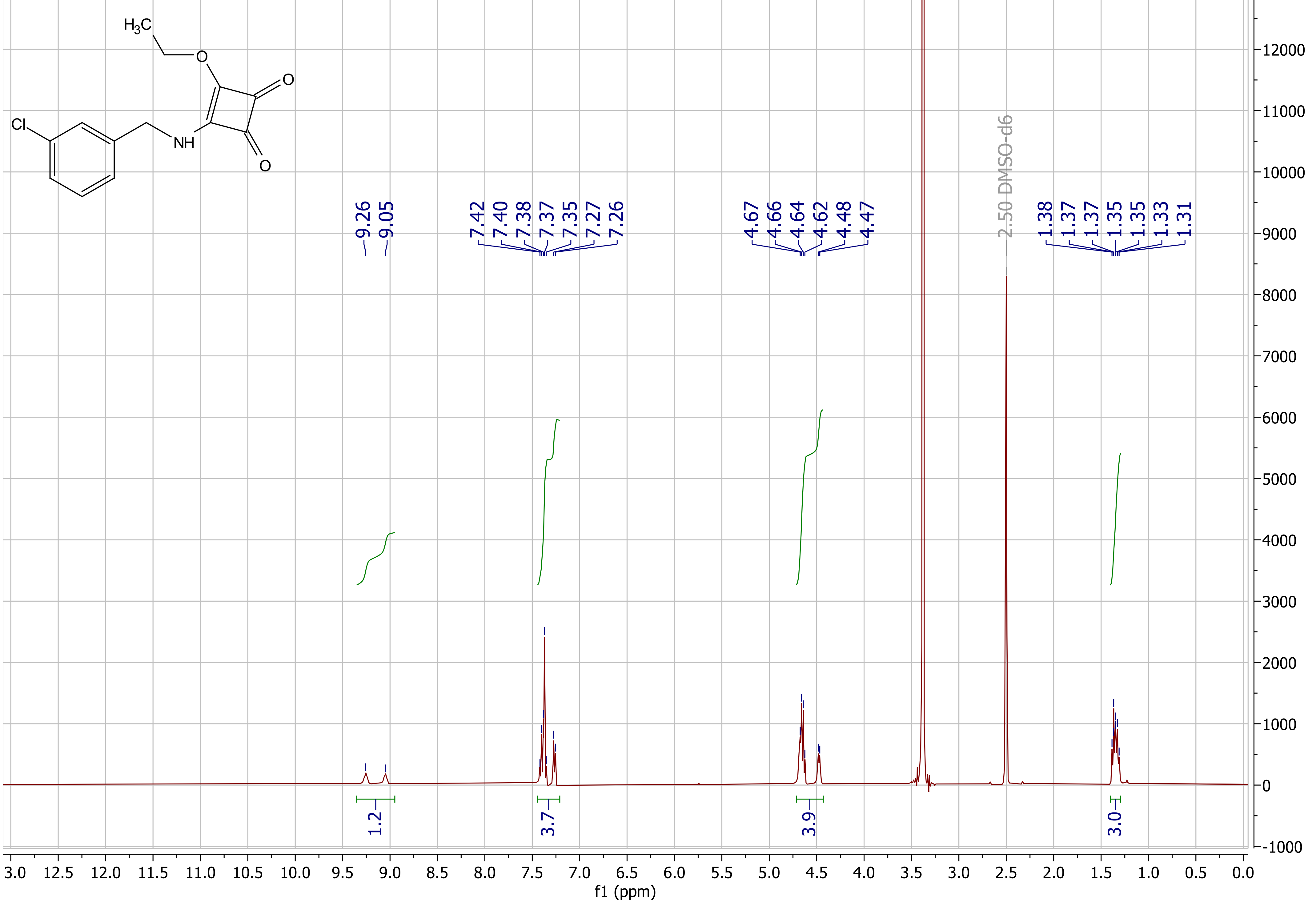

NL_50_3-Chlorobenzylamine_CNMR_DMSO_VT_10sD3.30.fid

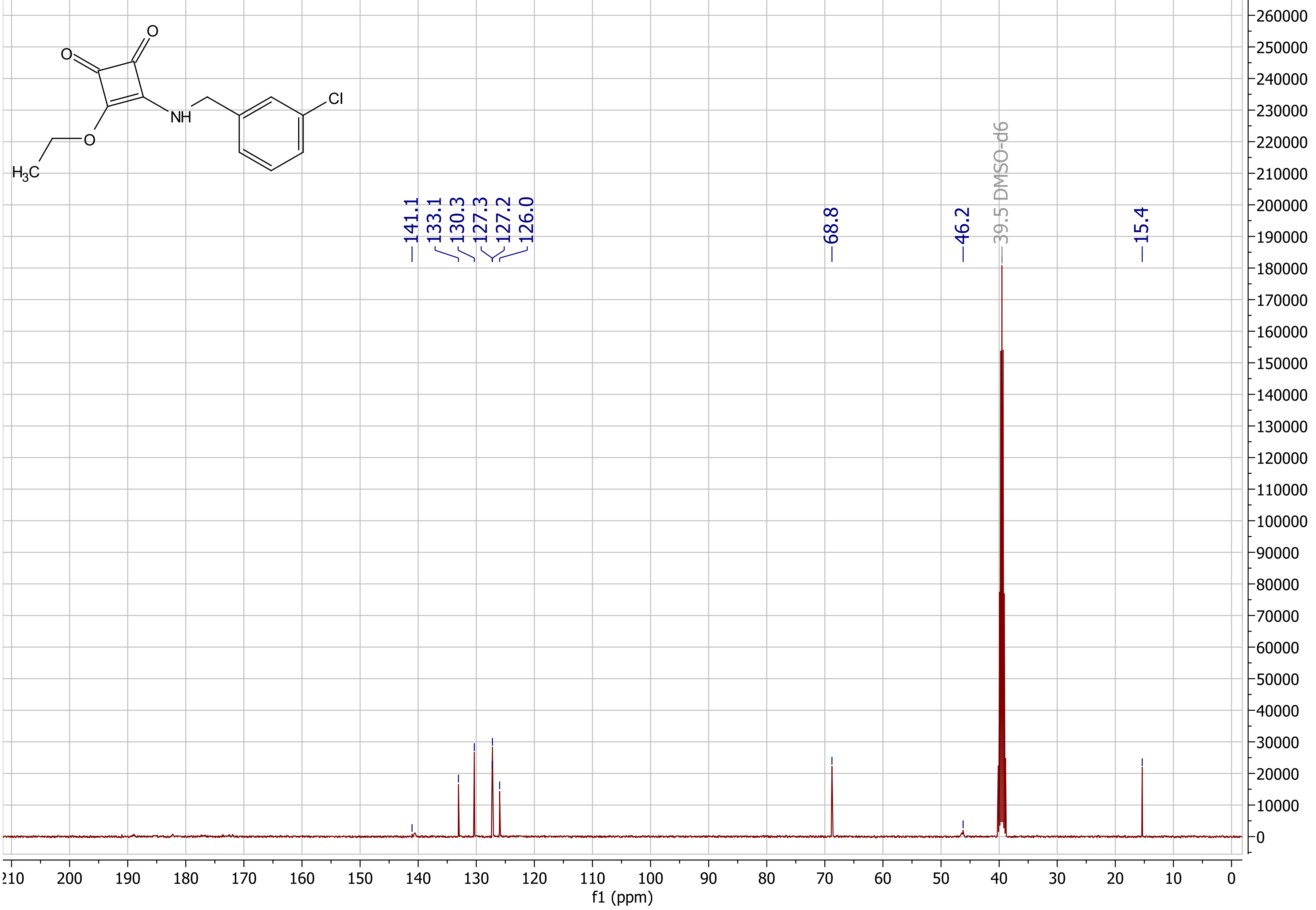

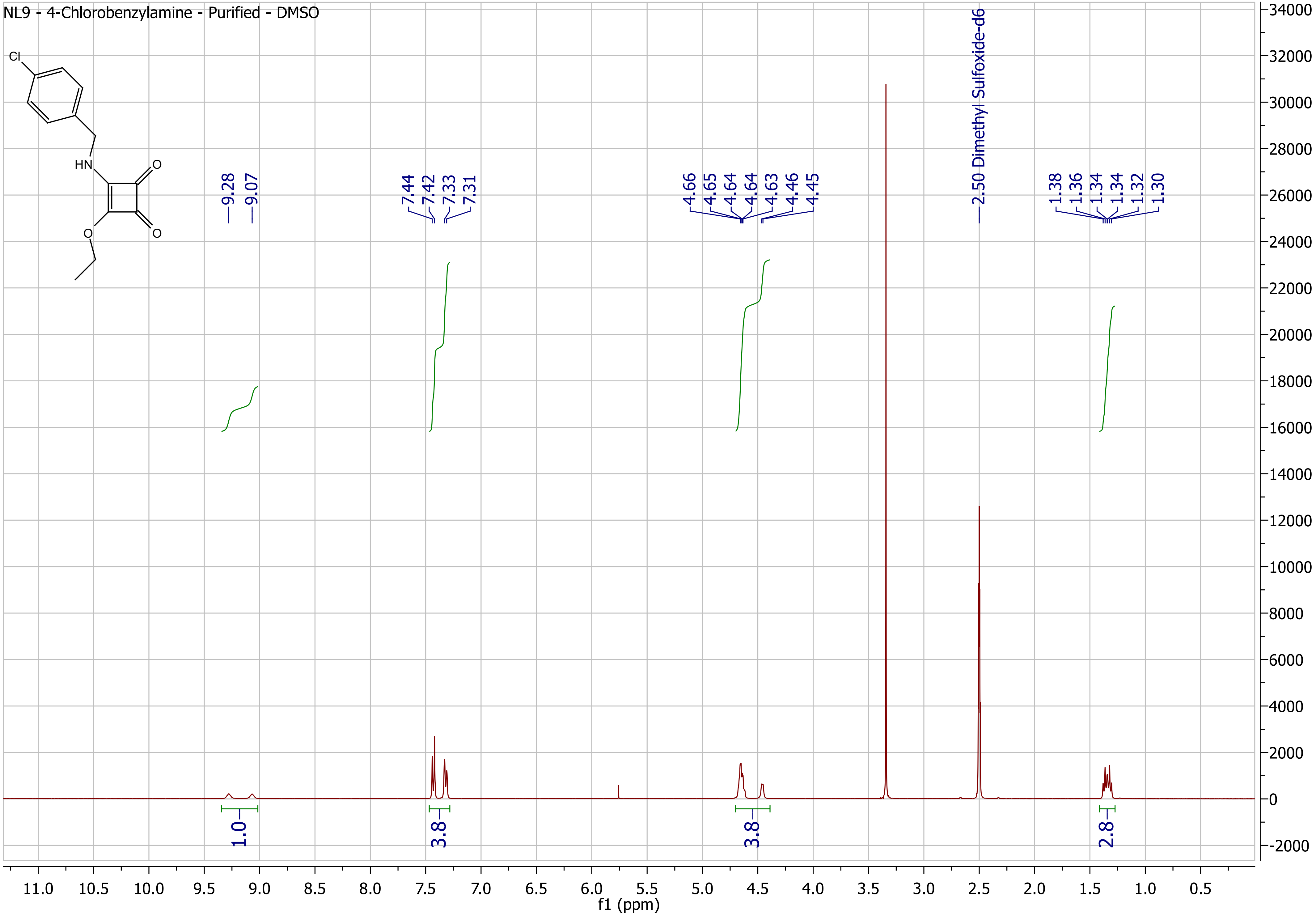
NL9 - 4-Chlorobenzylamine - Purified - DMSO
Cl
HN
O
O
O
9.28
9.07
7.44
7.42
7.33
7.31
4.66
4.65
4.64
4.64
4.63
4.46
4.45
2.50 Dimethyl Sulfoxide-d6
1.38
1.36
1.34
1.34
1.32
1.30
1.0
3.8
3.8
2.8
f1 (ppm)

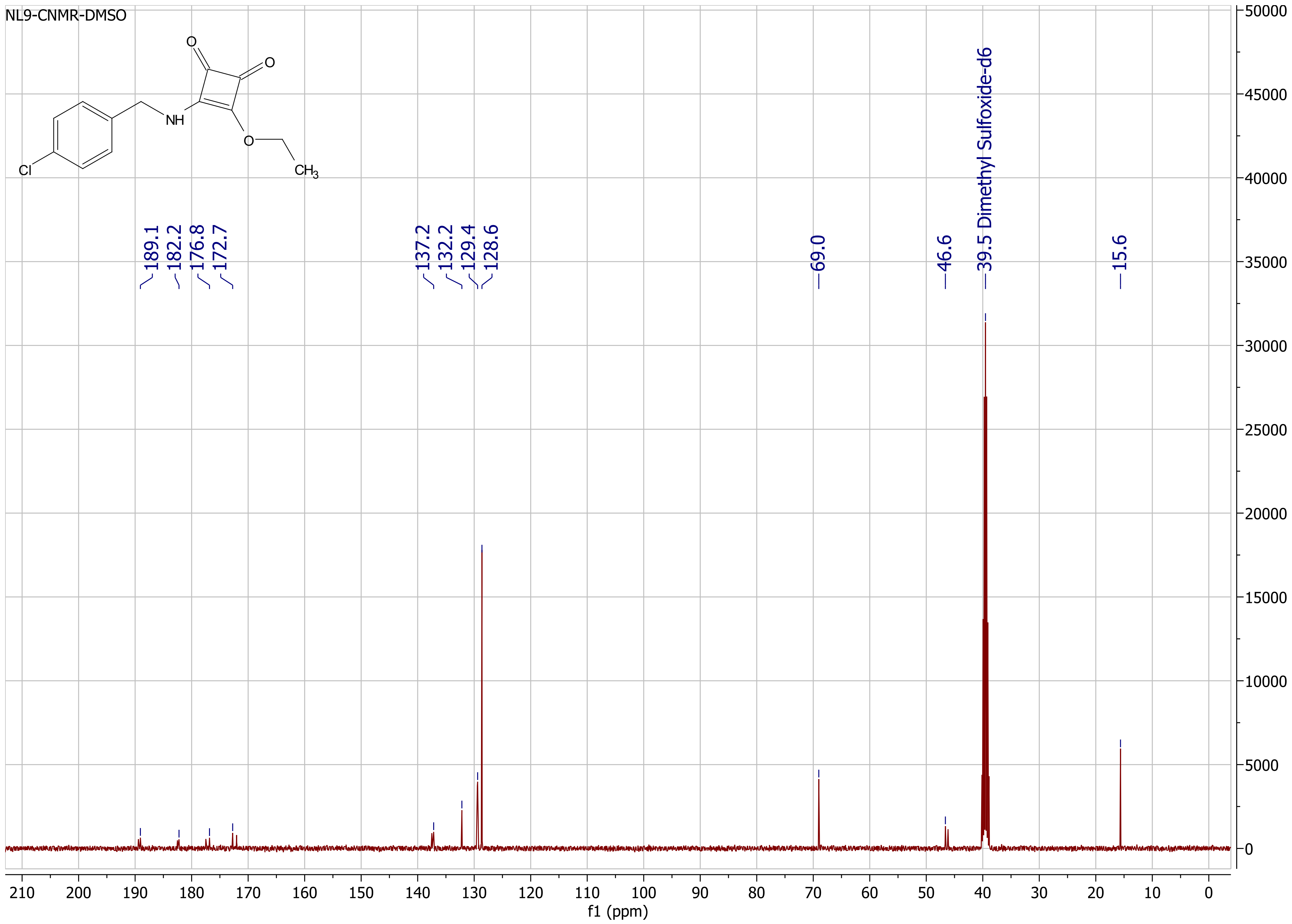

NL9-CNMR-DMSO
O
O
NH
O
Cl
CH3
189.1
182.2
176.8
172.7
137.2
132.2
129.4
128.6
69.0
46.6
39.5 Dimethyl Sulfoxide-d6
15.6
50000
45000
40000
35000
30000
25000
20000
15000
10000
5000
0
210 200 190 180 170 160 150 140 130 120 110 100 90 80 70 60 50 40 30 20 10 0
f1 (ppm)

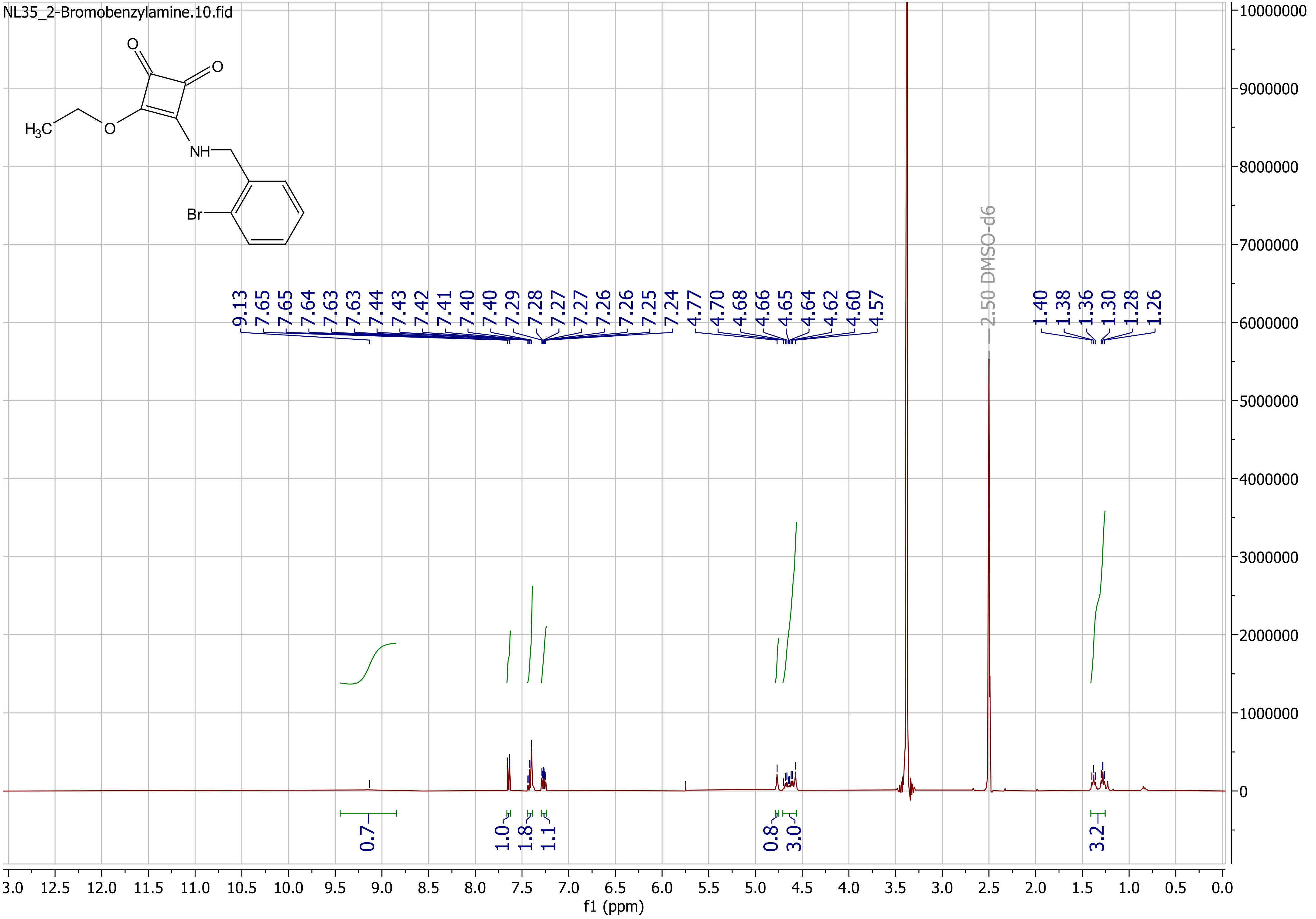

NL35_2-Bromobenzylamine.10.fid
O
O
H3C
O
NH
Br
9.13 7.65 7.65 7.64 7.63 7.63 7.44 7.43 7.42 7.41 7.40 7.40 7.29 7.28 7.27 7.27 7.26 7.26 7.25 7.24 4.77 4.70 4.68 4.66 4.65 4.64 4.62 4.60 4.57
2.50 DMSO-d6
1.40 1.38 1.36 1.30 1.28 1.26
0.7
1.0
1.8
1.1
0.8
3.0
3.2
10000000
9000000
8000000
7000000
6000000
5000000
4000000
3000000
2000000
1000000
0
3.0 12.5 12.0 11.5 11.0 10.5 10.0 9.5 9.0 8.5 8.0 7.5 7.0 6.5 6.0 5.5 5.0 4.5 4.0 3.5 3.0 2.5 2.0 1.5 1.0 0.5 0.0
f1 (ppm)

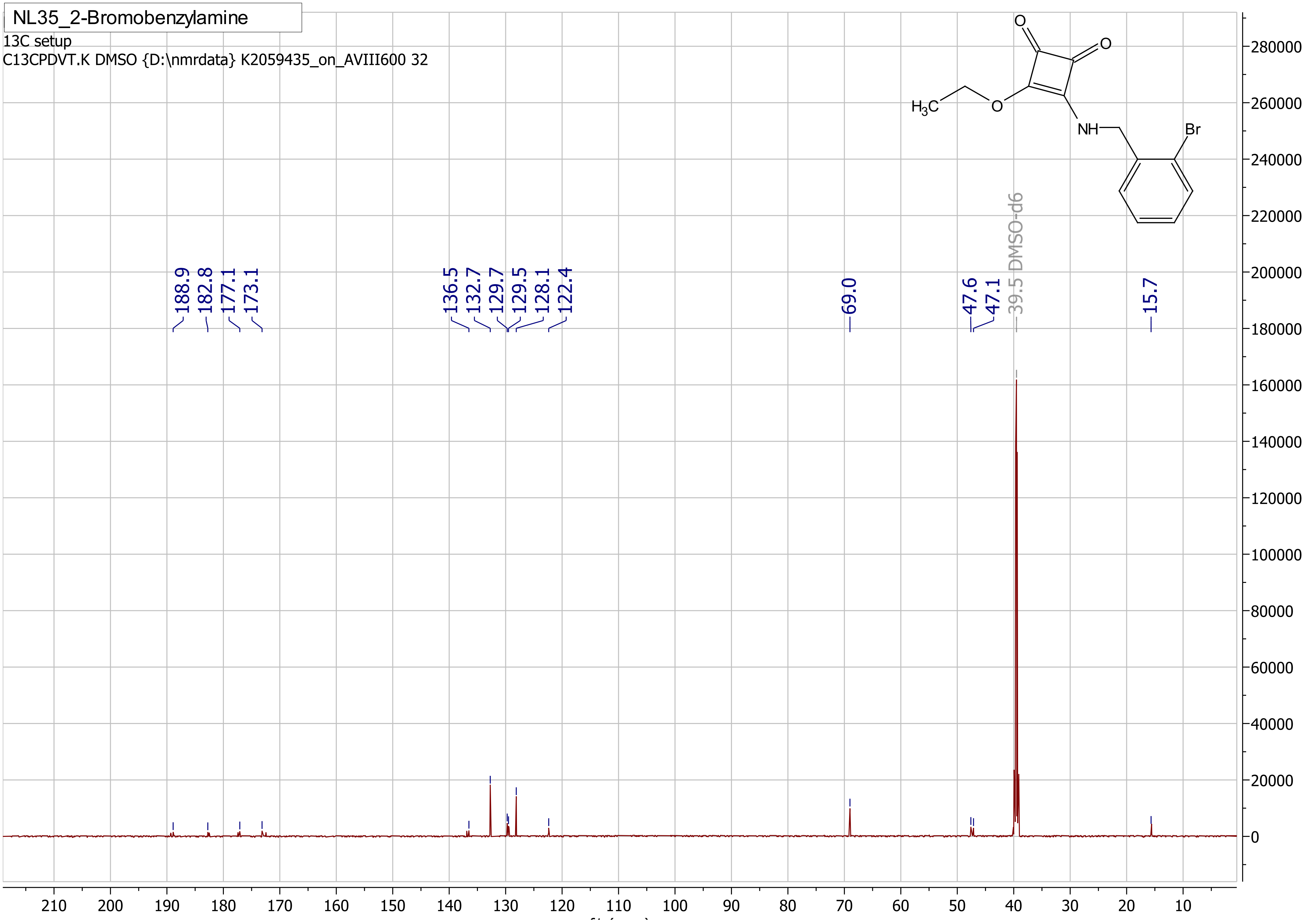

NL35_2-Bromobenzylamine
13C setup
C13CPDVT.K DMSO {D:\nmrdata} K2059435_on_AVIII600 32
188.9
182.8
177.1
173.1
136.5
132.7
129.7
129.5
128.1
122.4
69.0
47.6
47.1
39.5 DMSO-d6
15.7
O
O
H3C
O
NH
Br
280000
260000
240000
220000
200000
180000
160000
140000
120000
100000
80000
60000
40000
20000
0
210
200
190
180
170
160
150
140
130
120
110
100
90
80
70
60
50
40
30
20
10

NL13 - 3-Bromobenzylamine HNMR

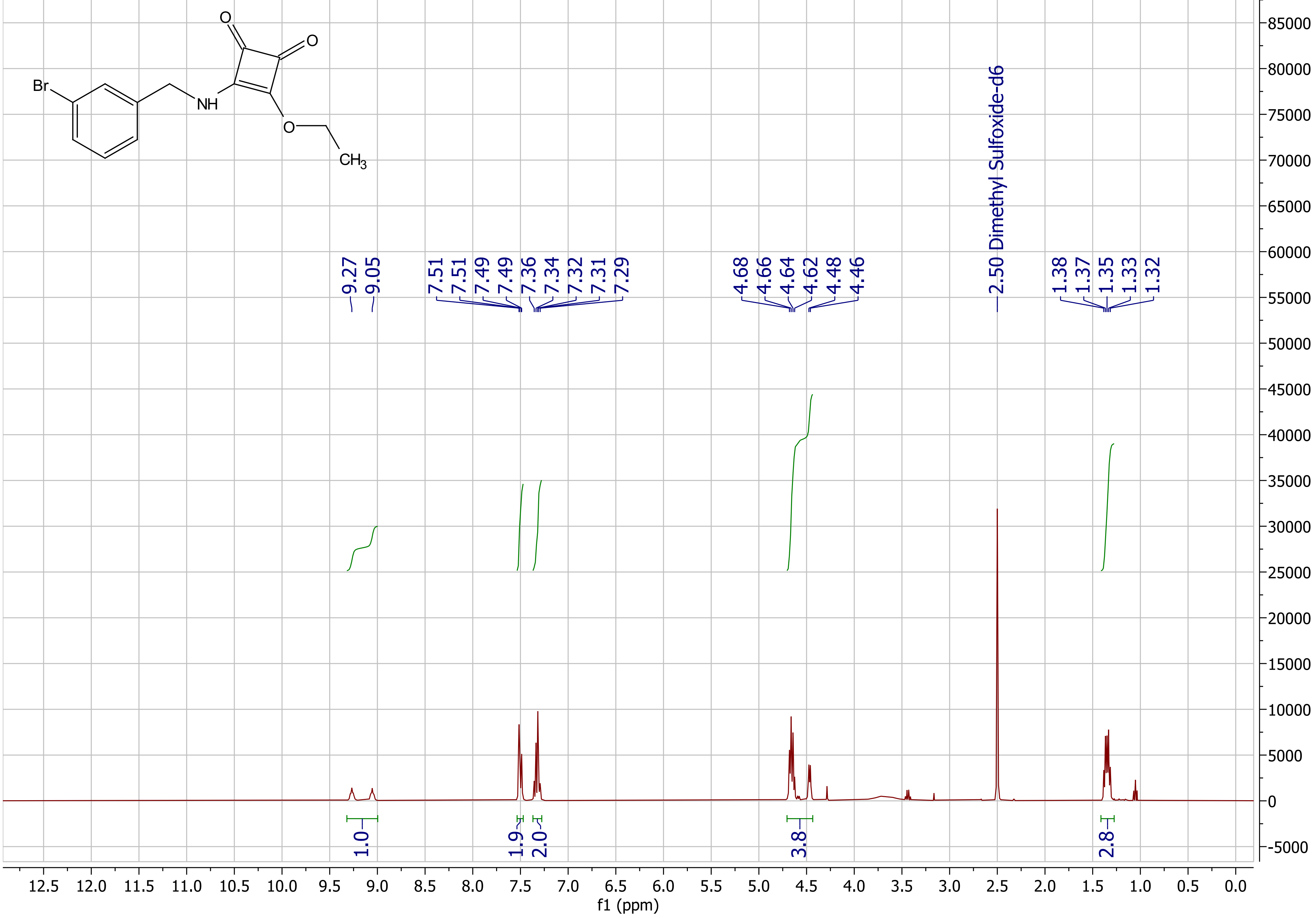

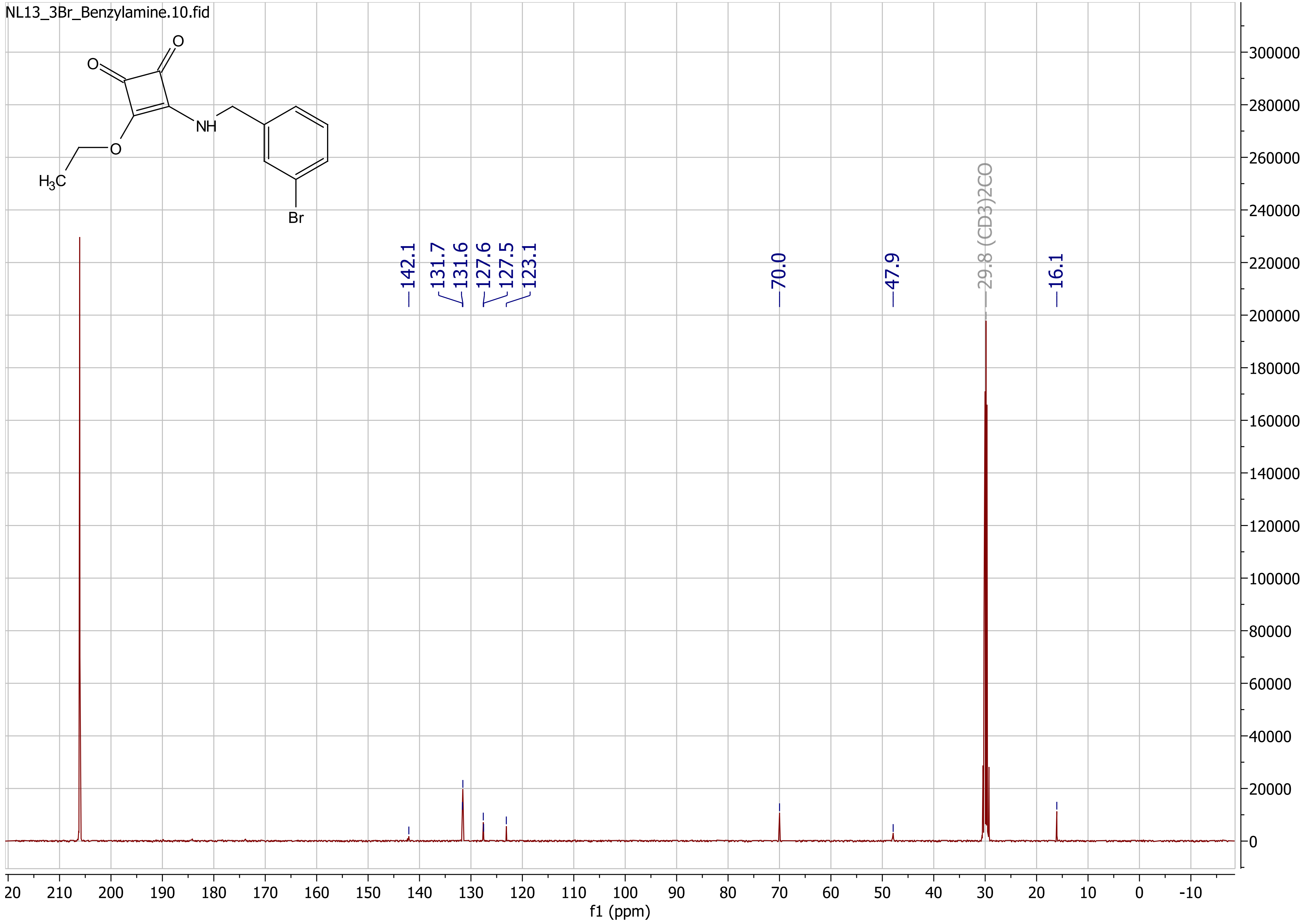

NL13_3Br_Benzylamine.10.fid
O
O
NH
O
H3C
Br
142.1
131.7
131.6
127.6
127.5
123.1
70.0
47.9
29.8 (CD3)2CO
16.1
f1 (ppm)

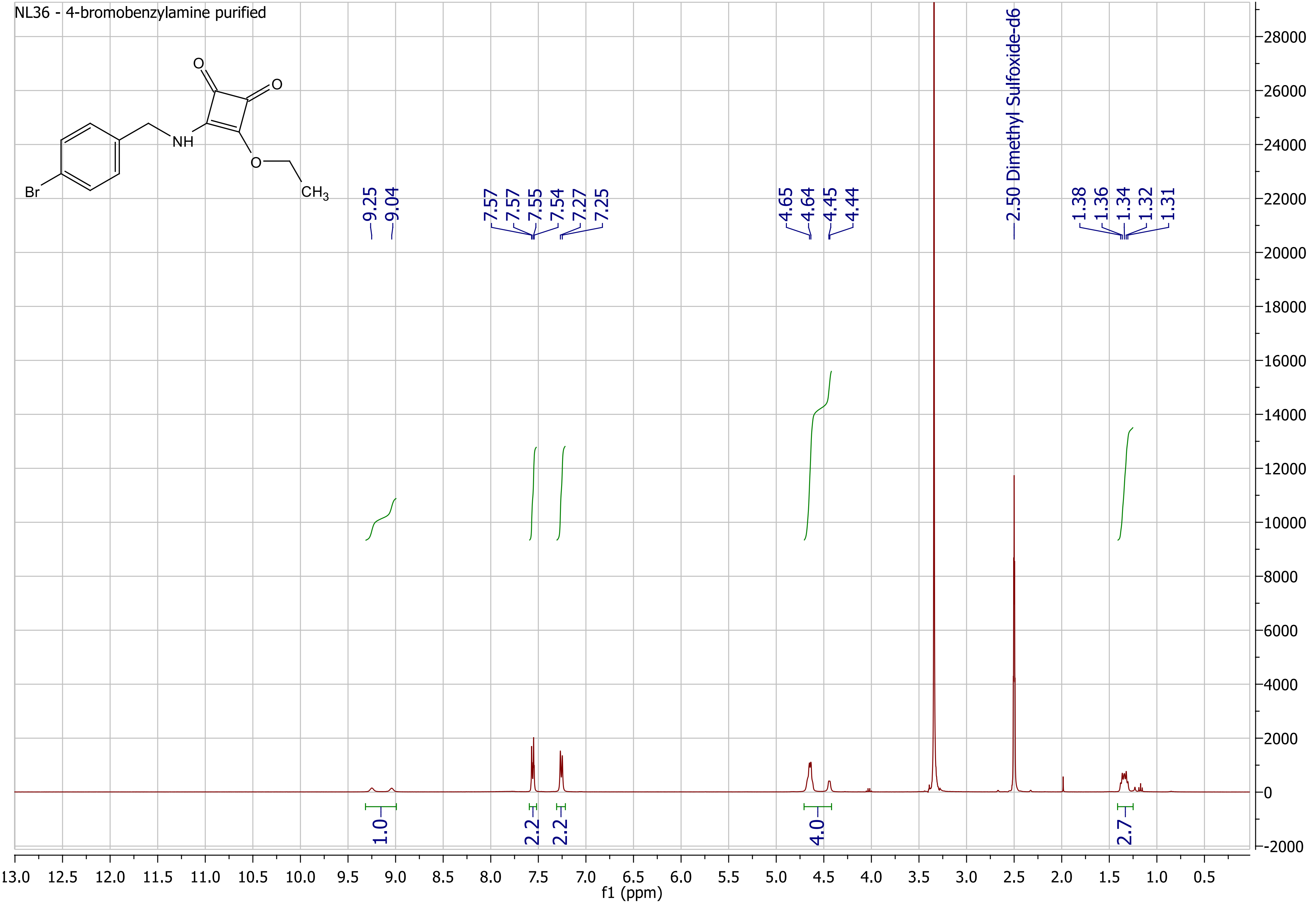
NL36 - 4-bromobenzylamine purified
O
O
NH
O
CH3
Br
9.25
9.04
7.57
7.57
7.55
7.54
7.27
7.25
4.65
4.64
4.45
4.44
2.50 Dimethyl Sulfoxide-d6
1.38
1.36
1.34
1.32
1.31
1.0
2.2
2.2
4.0
2.7
f1 (ppm)

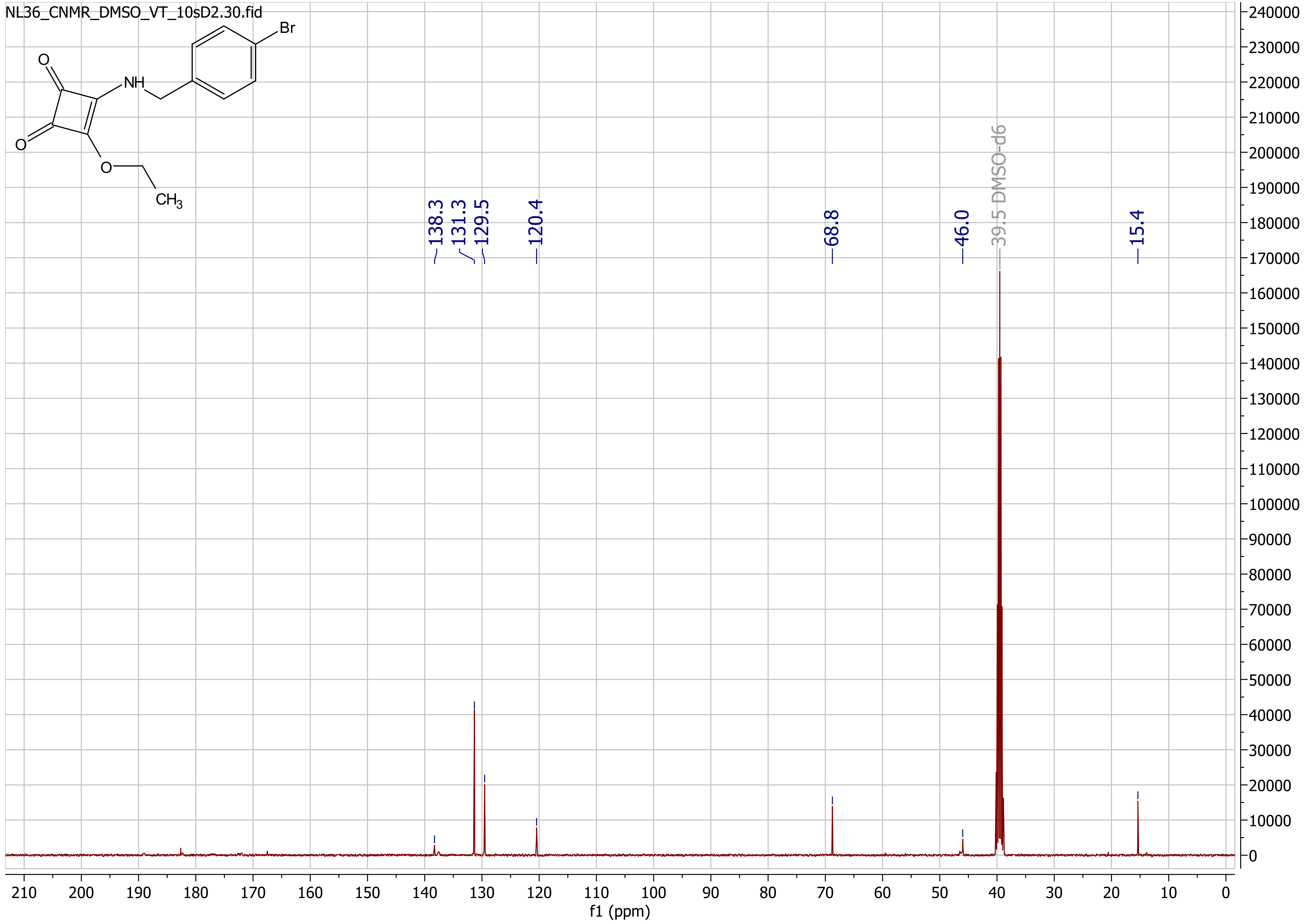
NL36_CNMR_DMSO_VT_10sD2.30.fid
Br
O
O
NH
O
CH3
138.3
131.3
129.5
120.4
68.8
46.0
39.5 DMSO-d6
15.4
f1 (ppm)

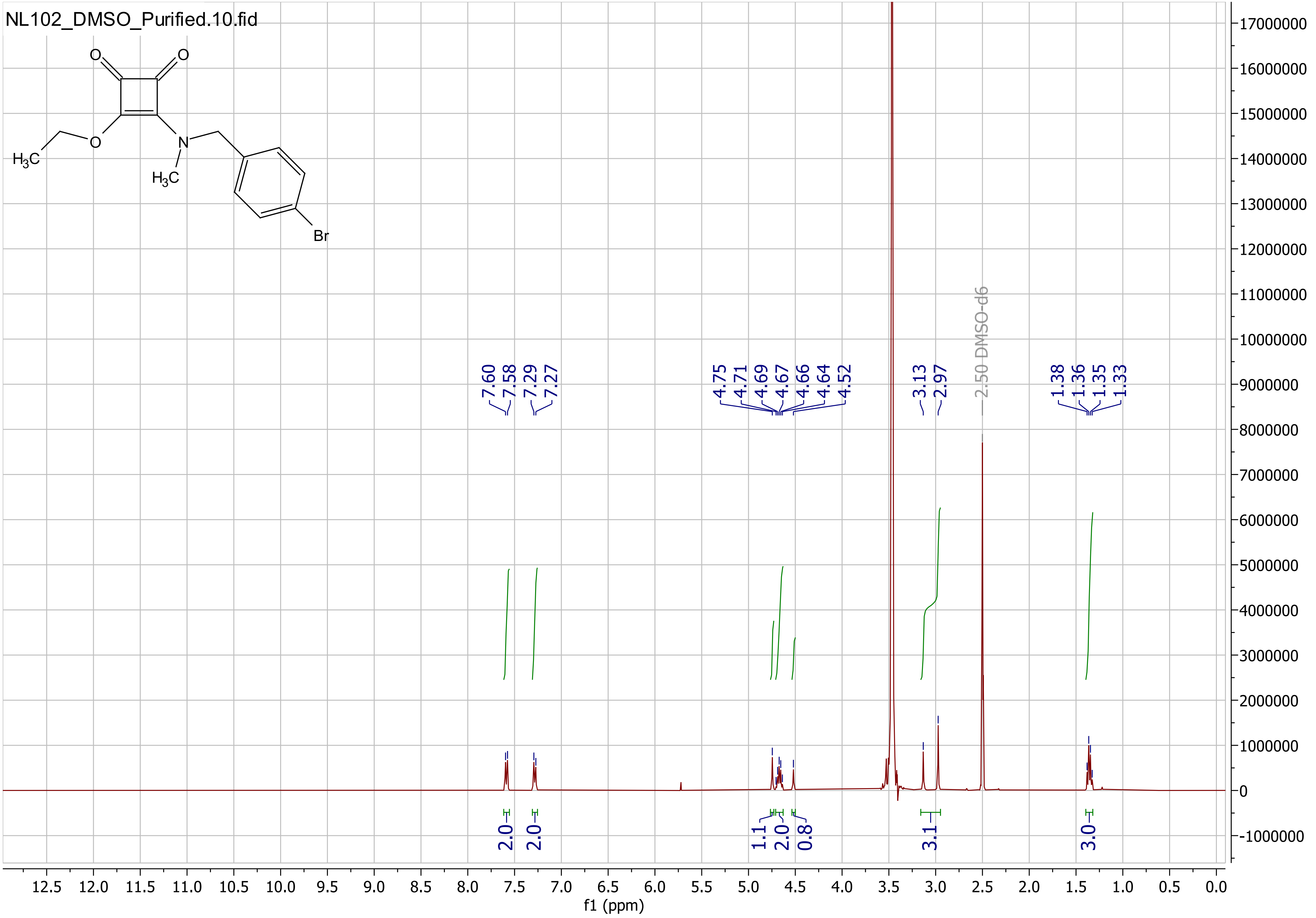
NL102_DMSO_Purified.10.fid
O
O
O
N
H_3C
H_3C
Br
7.60
7.58
7.29
7.27
4.75
4.71
4.69
4.67
4.66
4.64
4.52
3.13
2.97
2.50 DMSO-d6
1.38
1.36
1.35
1.33
2.0
2.0
1.1
2.0
0.8
3.1
3.0
f1 (ppm)

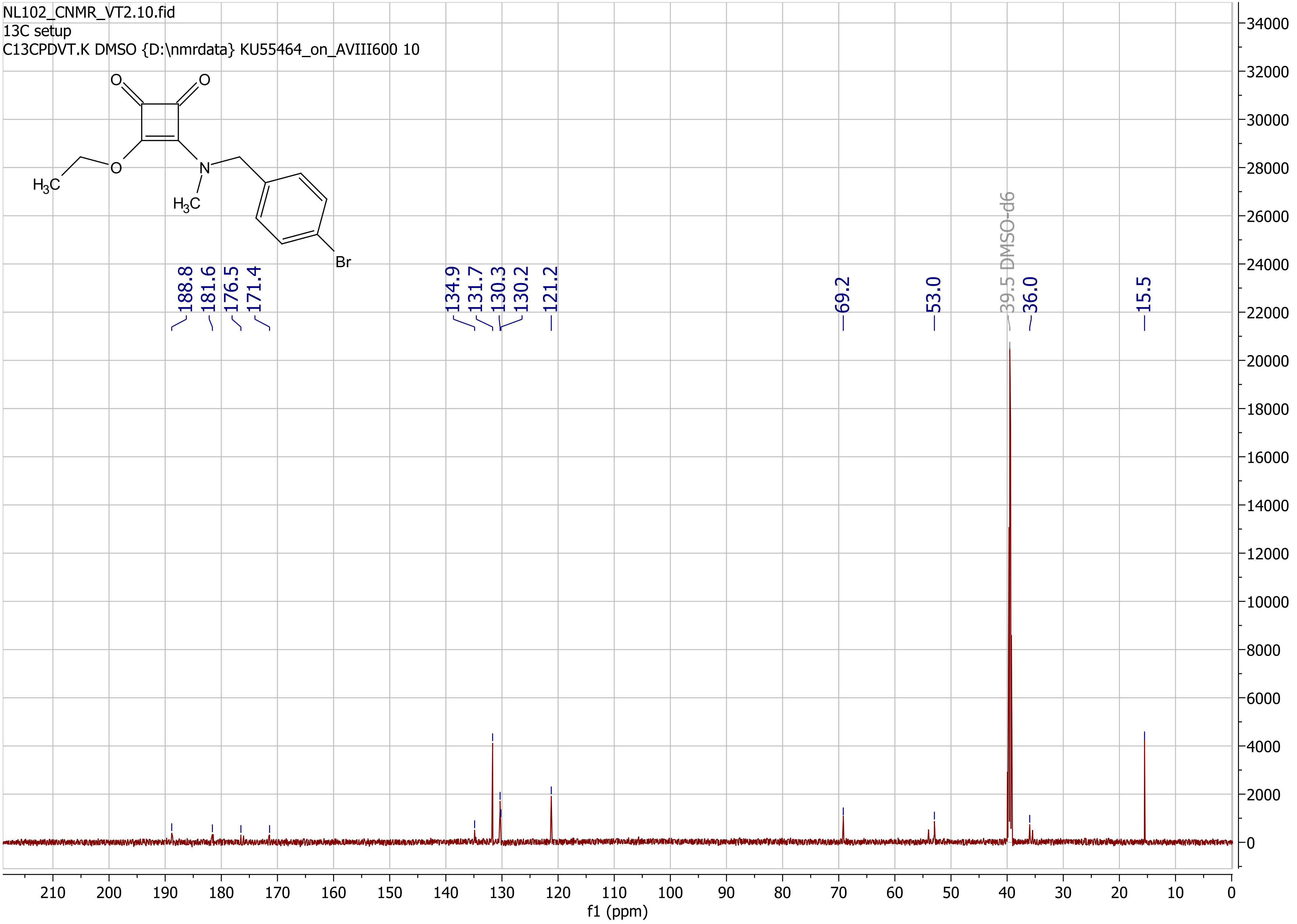
NL102_CNMR_VT2.10.fid
13C setup
C13CPDVT.K DMSO {D:\nmrdata} KU55464_on_AVIII600 10
O
O
O
N
H3C
H3C
Br
188.8
181.6
176.5
171.4
134.9
131.7
130.3
130.2
121.2
69.2
53.0
39.5 DMSO-d6
36.0
15.5
f1 (ppm)

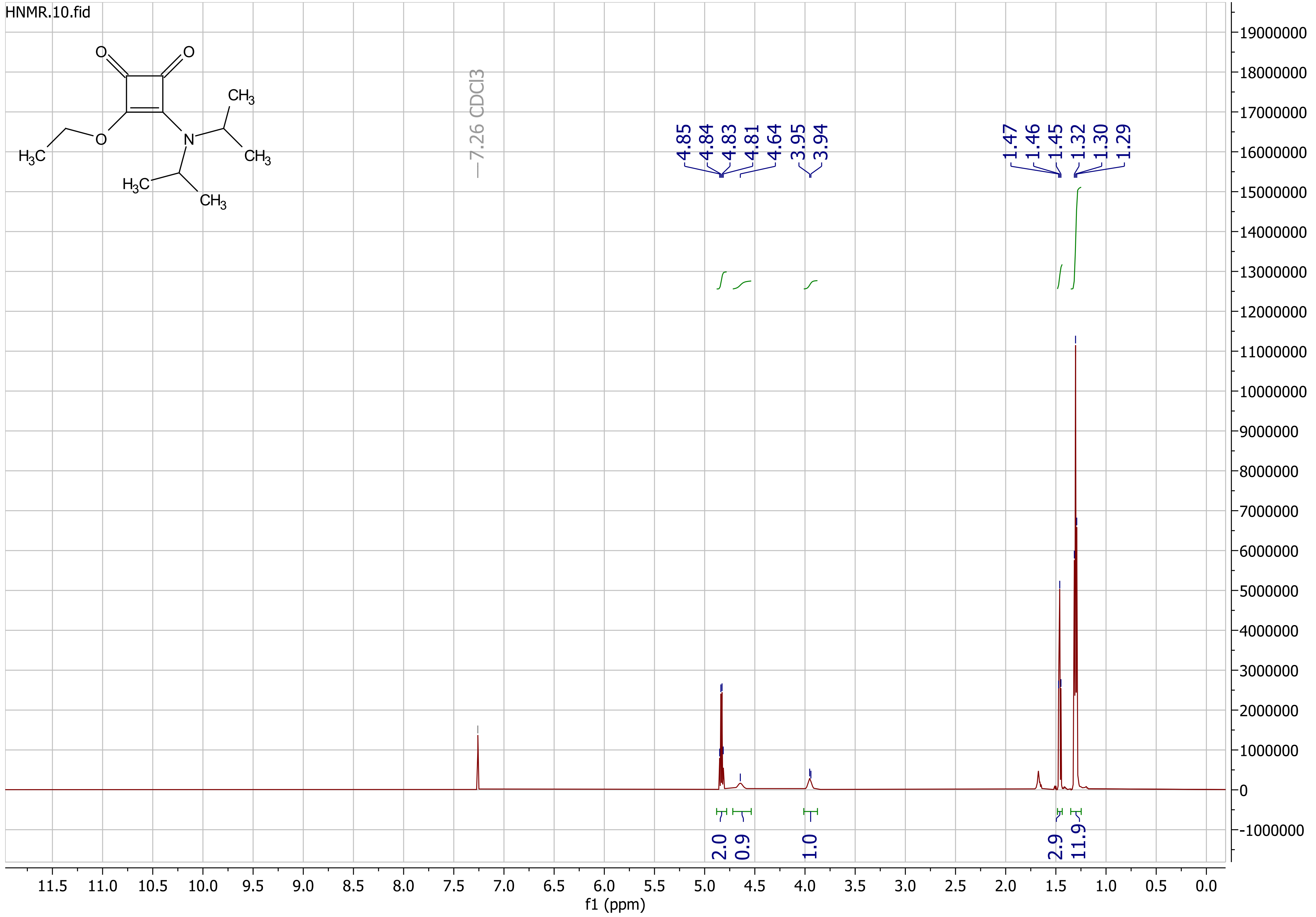
HNMR.10.fid
7.26 CDCl3
4.85
4.84
4.83
4.81
4.64
3.95
3.94
1.47
1.46
1.45
1.32
1.30
1.29
2.0
0.9
1.0
2.9
11.9
f1 (ppm)

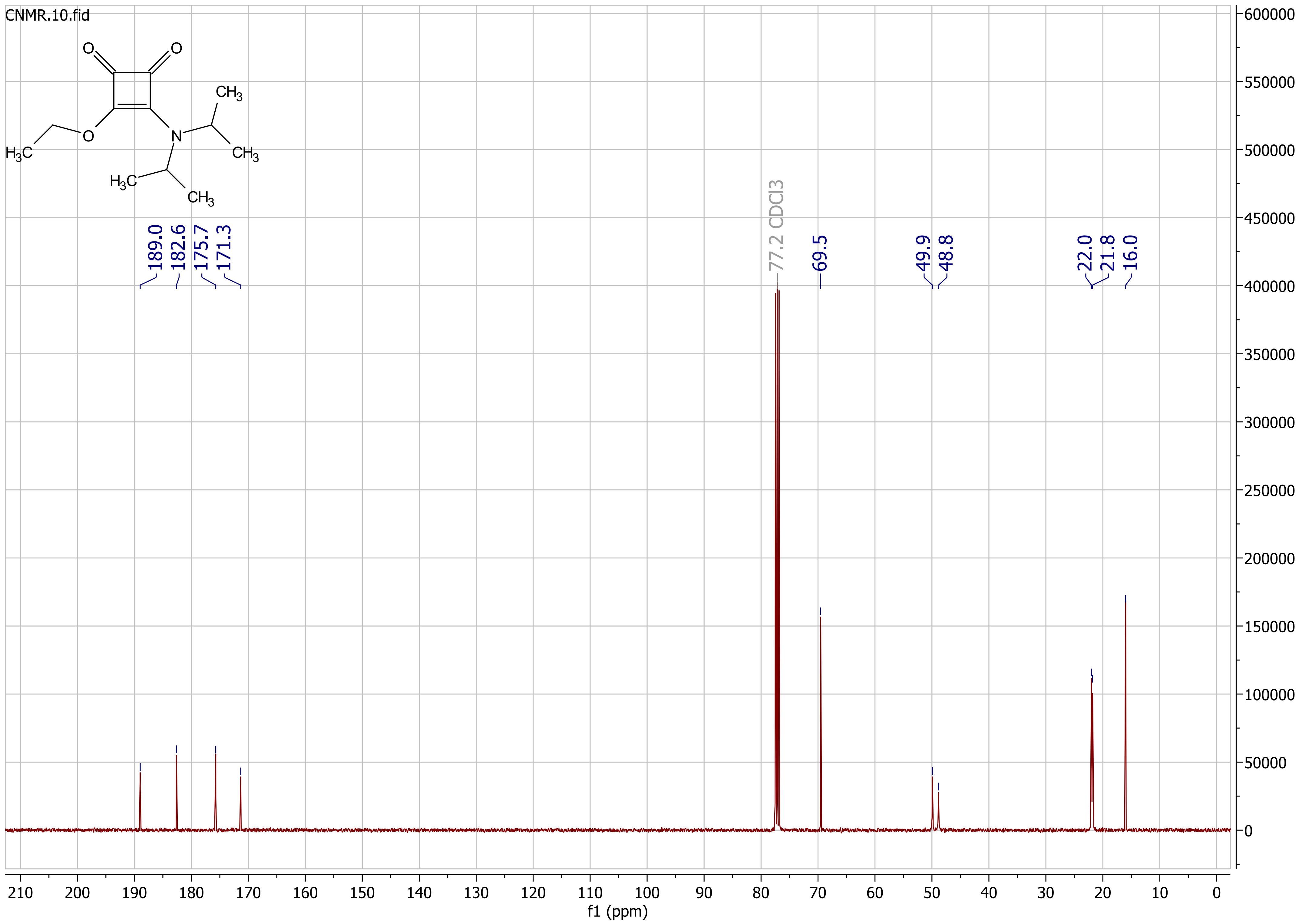
CNMR.10.fid
189.0
182.6
175.7
171.3
77.2 CDCl3
69.5
49.9
48.8
22.0
21.8
16.0
f1 (ppm)

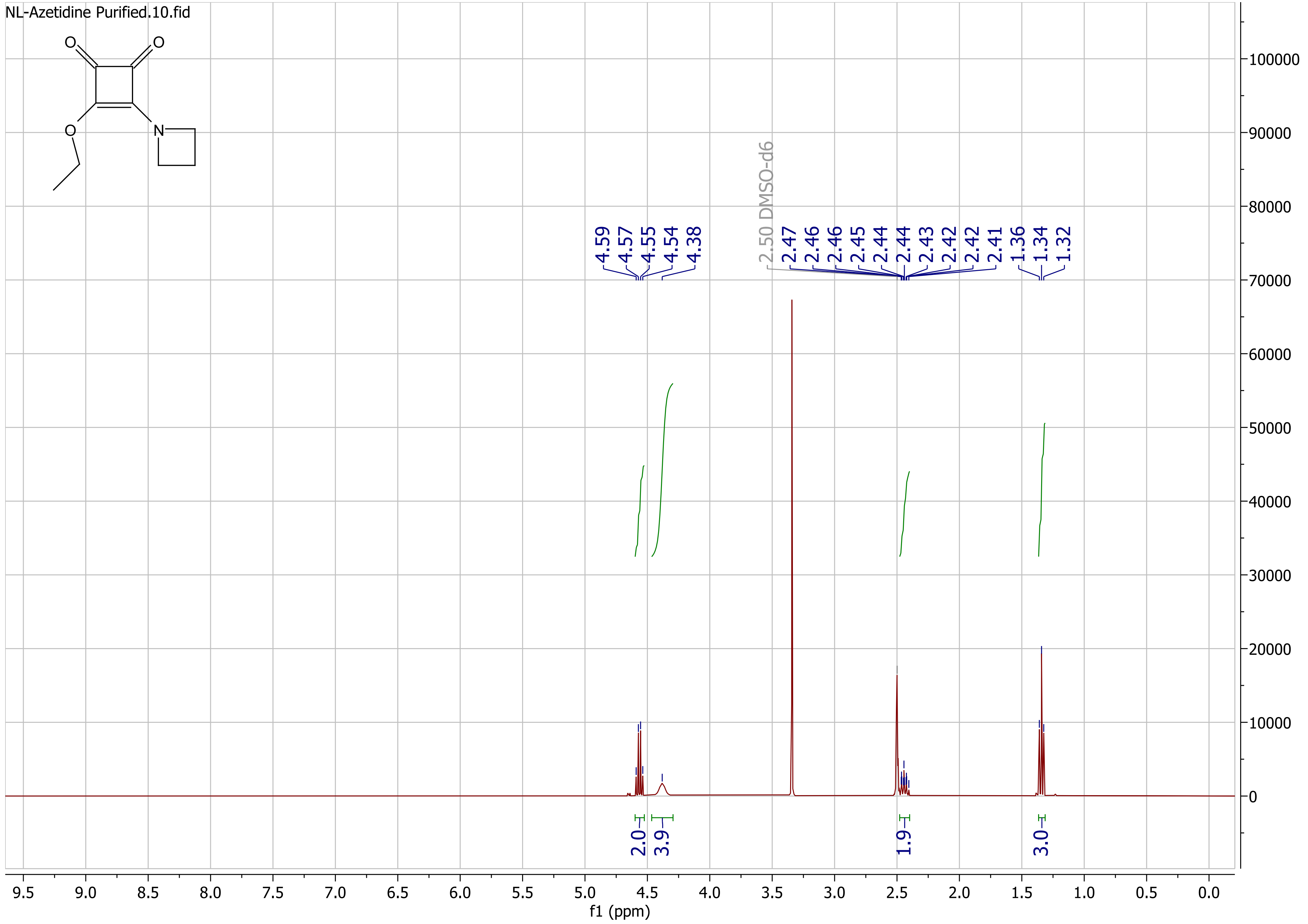
NL-Azetidine Purified.10.fid
O
O
O
N
2.50 DMSO-d6
4.59
4.57
4.55
4.54
4.38
2.47
2.46
2.46
2.45
2.44
2.44
2.43
2.42
2.42
2.41
1.36
1.34
1.32
2.0
3.9
1.9
3.0
100000
90000
80000
70000
60000
50000
40000
30000
20000
10000
0
9.5
9.0
8.5
8.0
7.5
7.0
6.5
6.0
5.5
5.0
4.5
4.0
3.5
3.0
2.5
2.0
1.5
1.0
0.5
0.0
f1 (ppm)

NL59_Azetidine_CNMR.10.fid

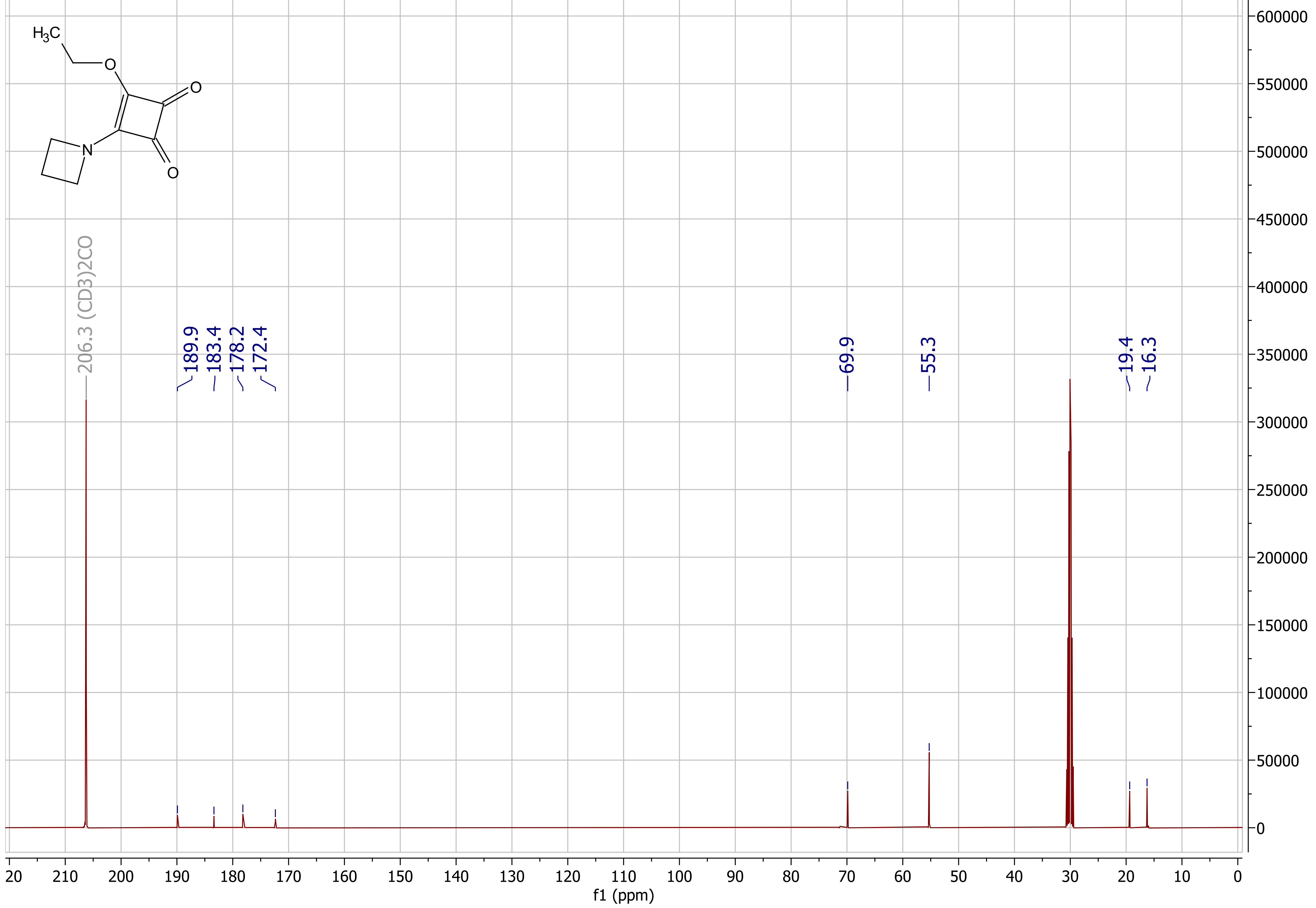

NL33 - Pyrrolidine Pure

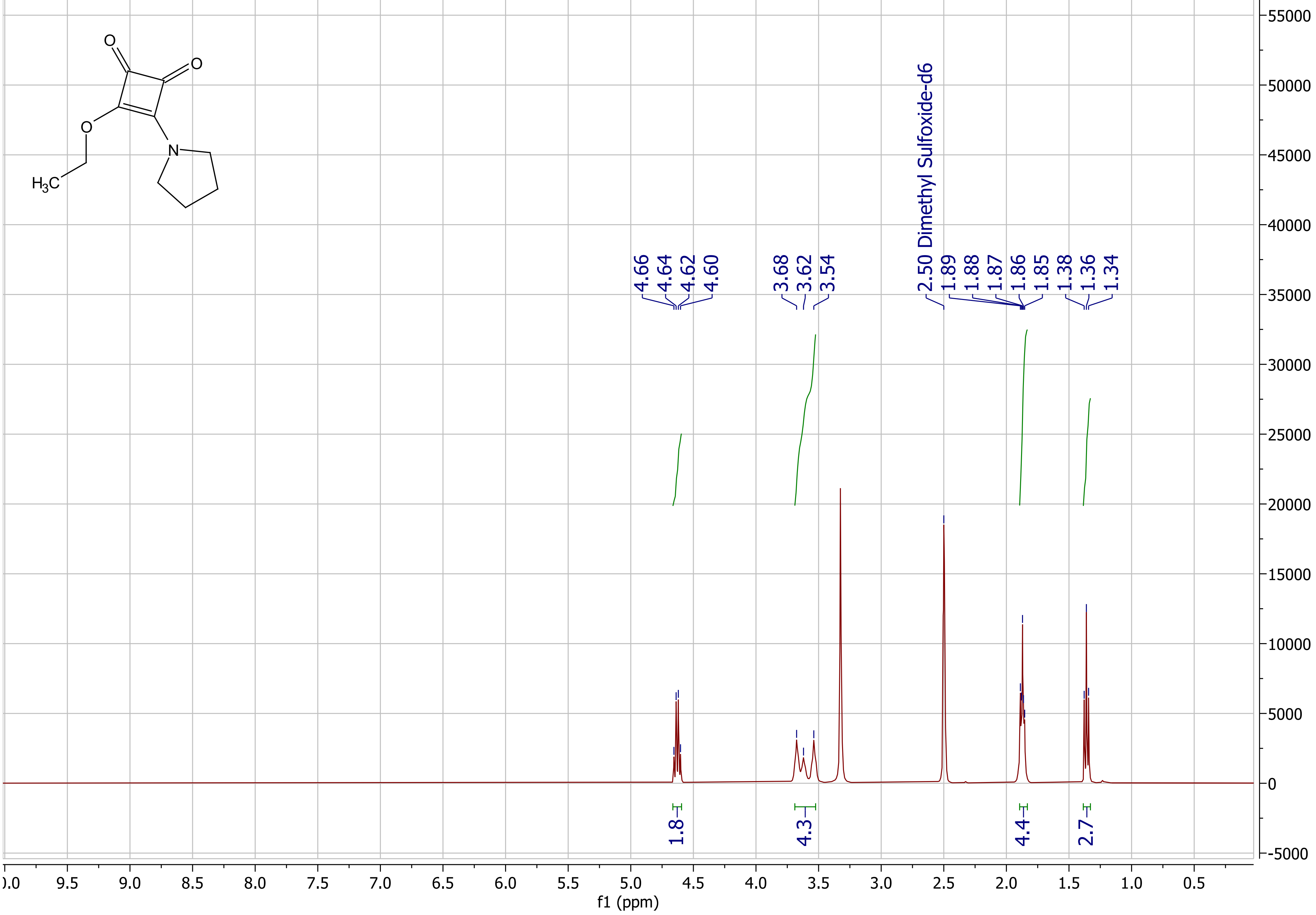

NL33_CNMR_DMSO_VT_10sD1.20.fid

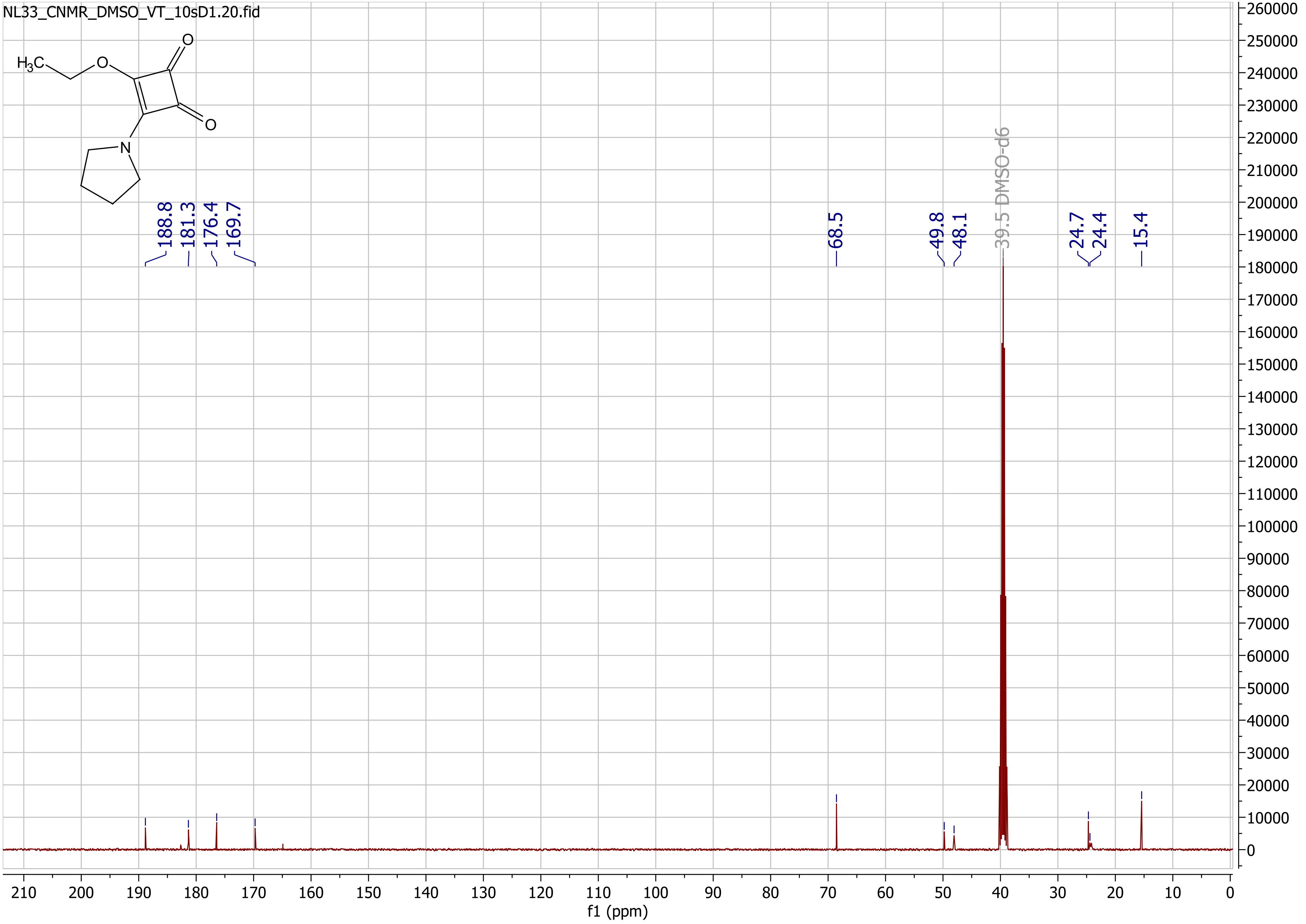

NL44 - Piperidine Pure.10.fid
PROTON.K DMSO {D:\nmrdata} K2059435_on_AVIII600 44

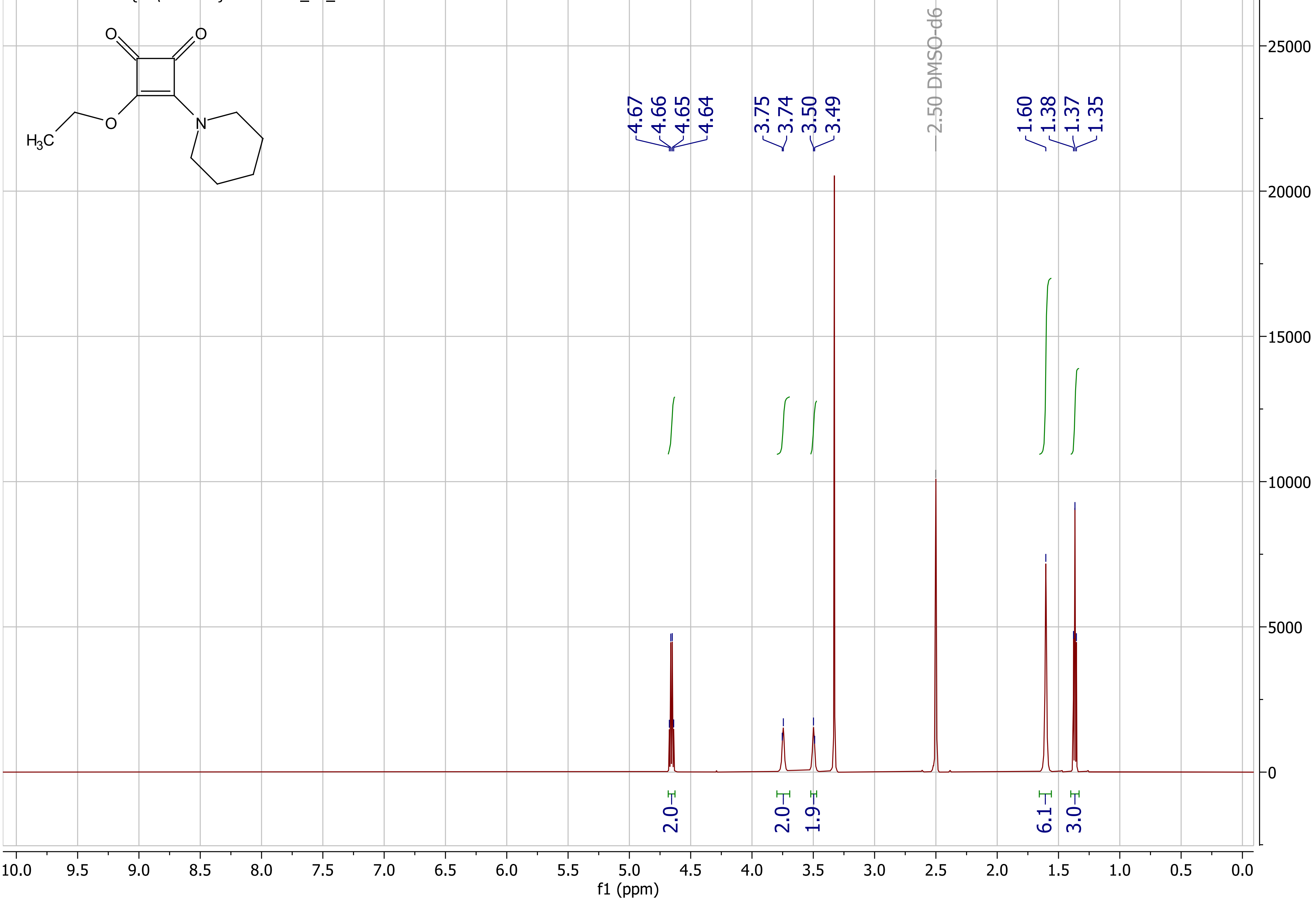

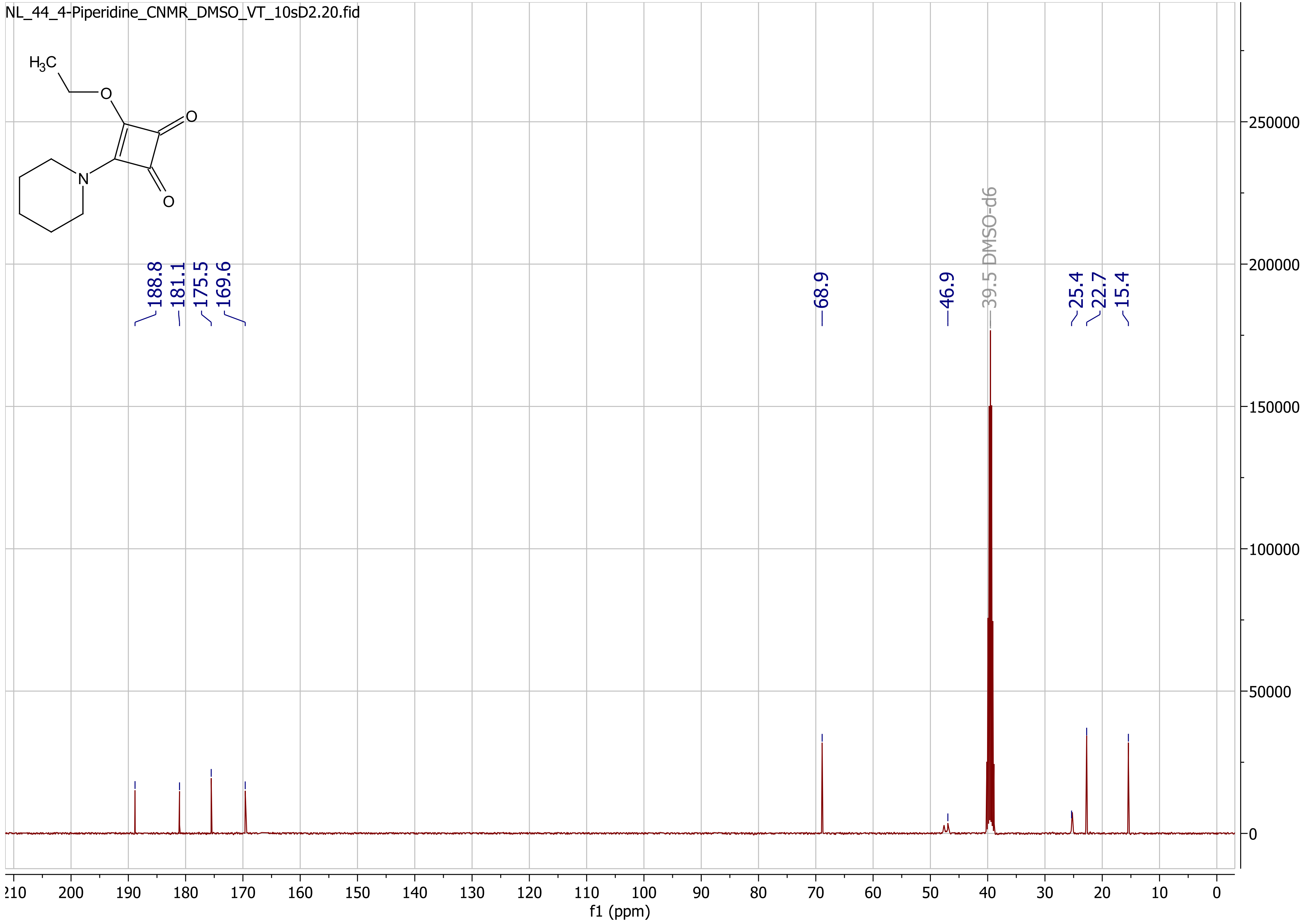
NL_44_4-Piperidine_CNMR_DMSO_VT_10sD2.20.fid
H3C
O
O
O
N
188.8
181.1
175.5
169.6
68.9
46.9
39.5 DMSO-d6
25.4
22.7
15.4
250000
200000
150000
100000
50000
0
210
200
190
180
170
160
150
140
130
120
110
100
90
80
70
60
50
40
30
20
10
0
f1 (ppm)

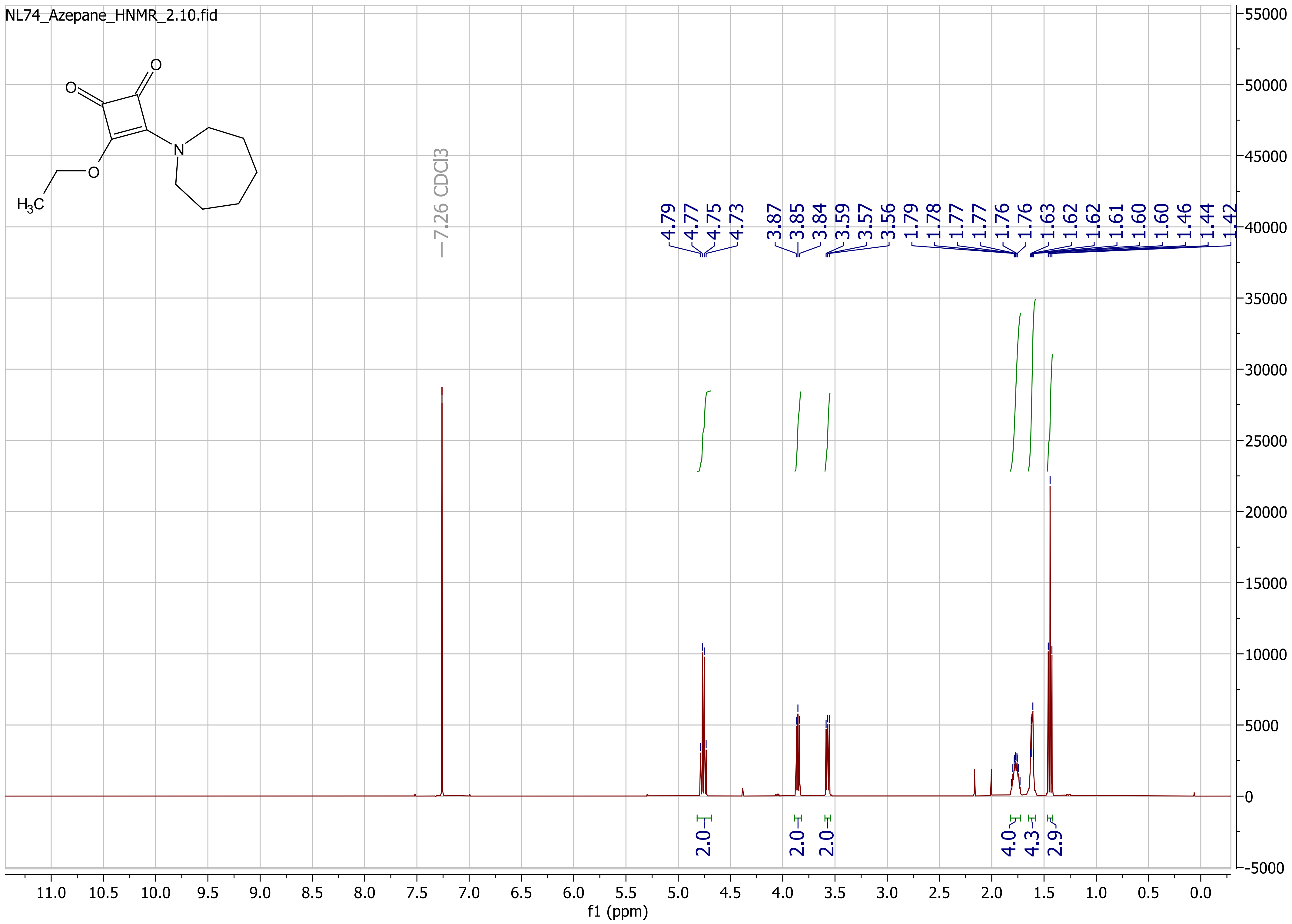
NL74_Azepane_HNMR_2.10.fid
O
O
O
N
H_3C
O
7.26 CDCl3
4.79
4.77
4.75
4.73
3.87
3.85
3.84
3.59
3.57
3.56
1.79
1.78
1.77
1.77
1.76
1.76
1.63
1.62
1.62
1.61
1.60
1.60
1.46
1.44
1.42
2.0
2.0
2.0
4.0
4.3
2.9
f1 (ppm)

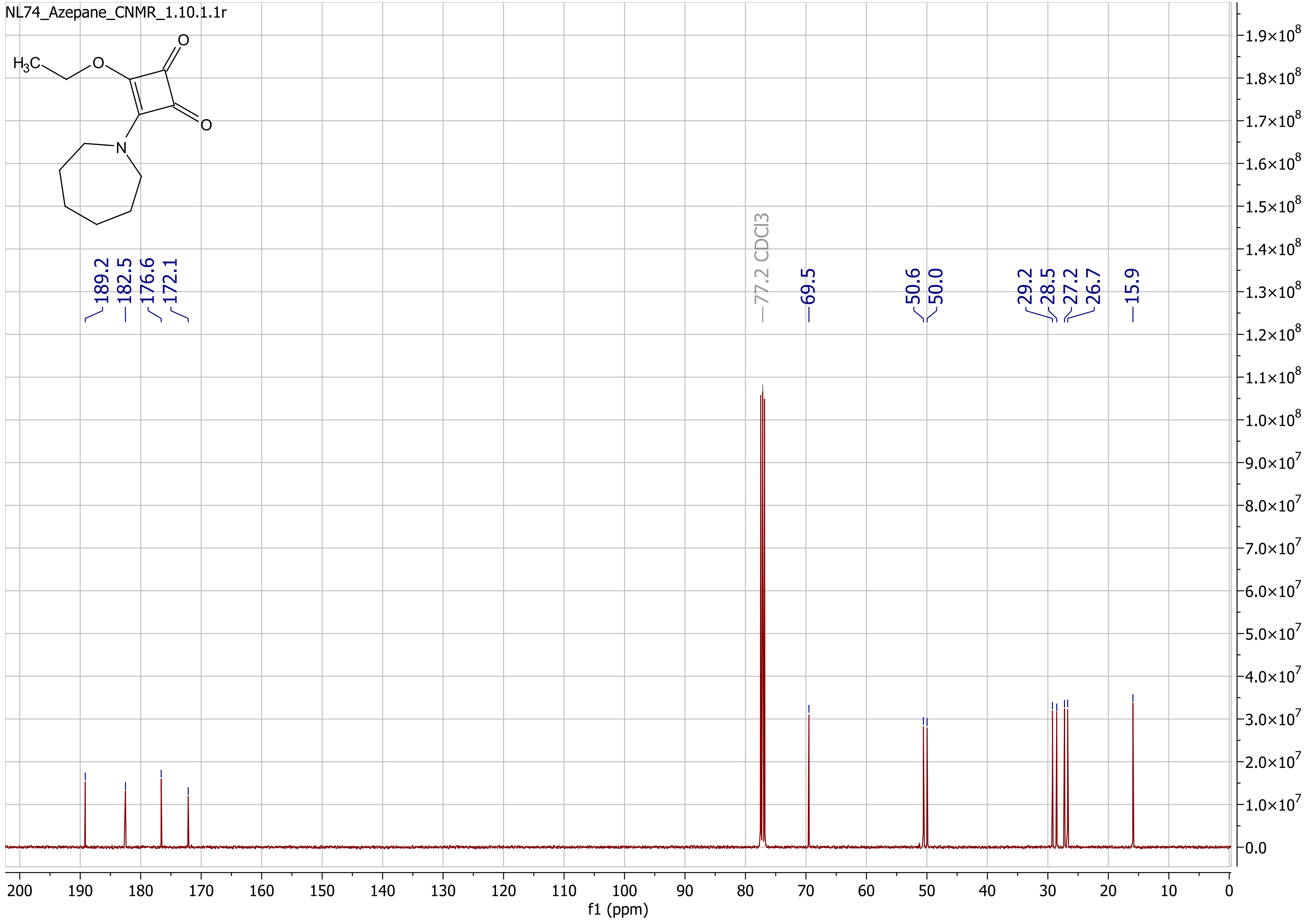

NL74_Azepane_CNMR_1.10.1.1r
H3C
O
O
O
N
189.2
182.5
176.6
172.1
77.2 CDCl3
69.5
50.6
50.0
29.2
28.5
27.2
26.7
15.9
f1 (ppm)

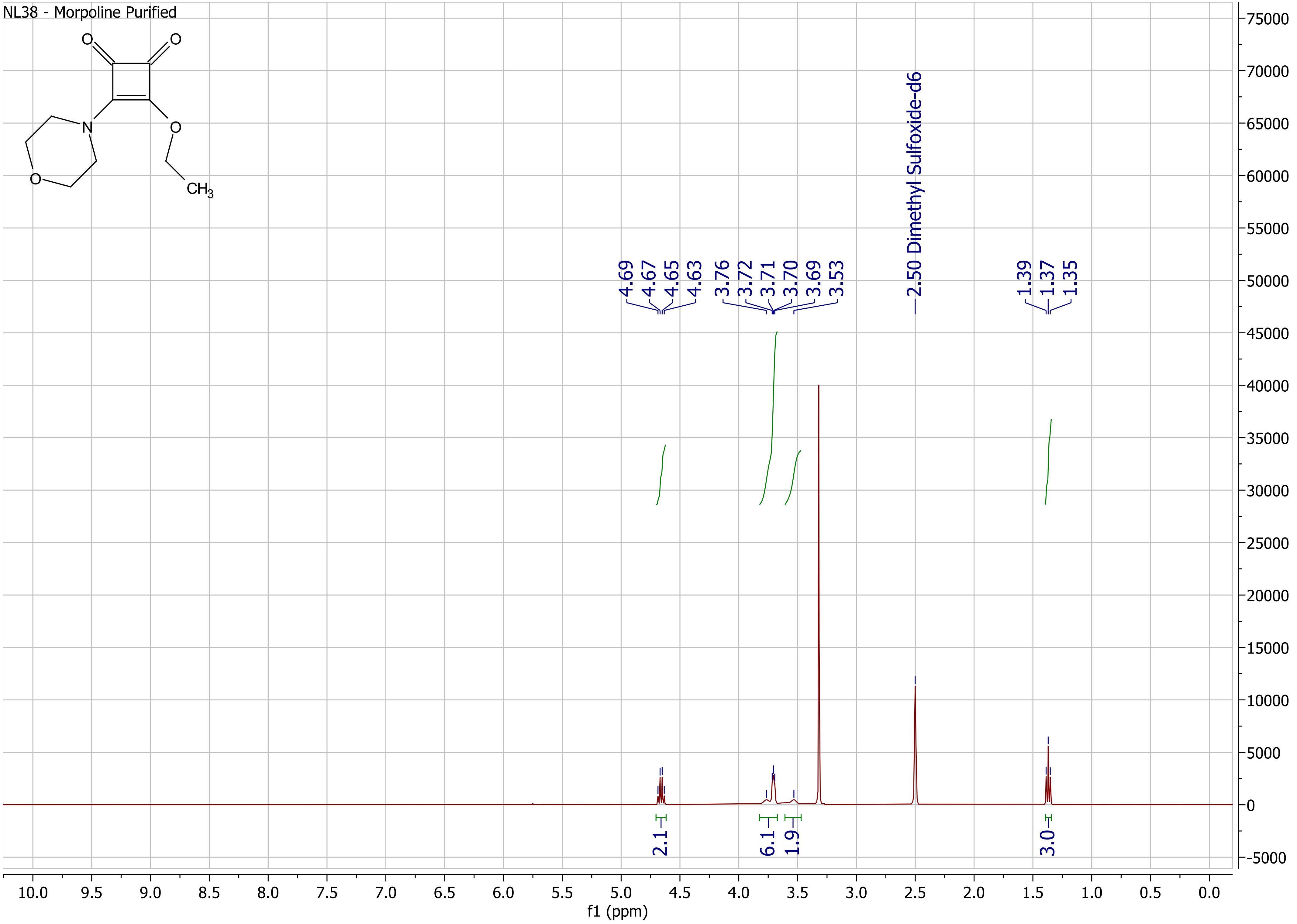
NL38 - Morpoline Purified
O
O
N
O
O
CH3
4.69
4.67
4.65
4.63
3.76
3.72
3.71
3.70
3.69
3.53
2.50 Dimethyl Sulfoxide-d6
1.39
1.37
1.35
2.1
6.1
1.9
3.0
75000
70000
65000
60000
55000
50000
45000
40000
35000
30000
25000
20000
15000
10000
5000
0
-5000
10.0
9.5
9.0
8.5
8.0
7.5
7.0
6.5
6.0
5.5
5.0
4.5
4.0
3.5
3.0
2.5
2.0
1.5
1.0
0.5
0.0
f1 (ppm)

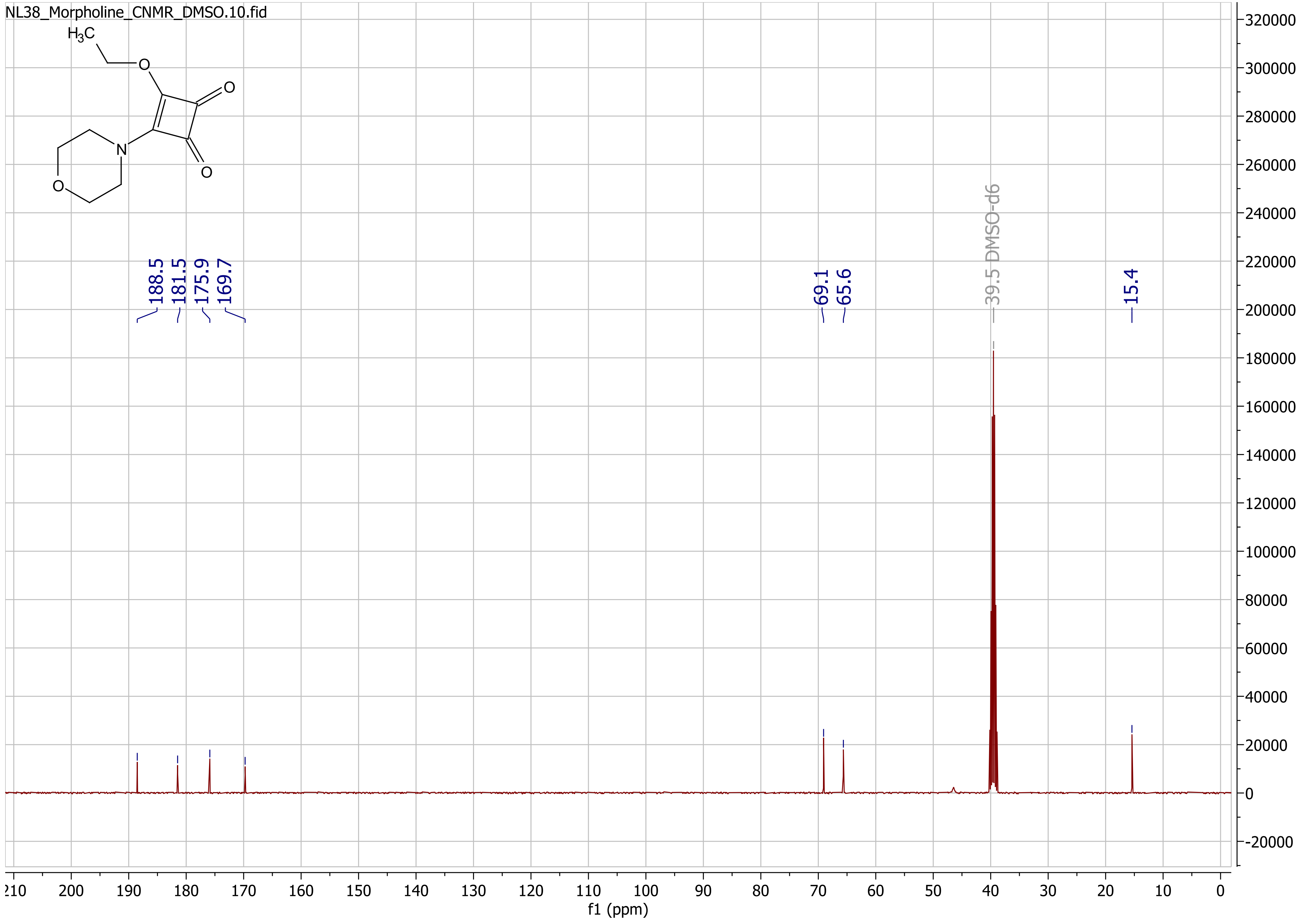
NL38_Morpholine_CNMR_DMSO.10.fid
H3C
O
O
N
O
O
188.5
181.5
175.9
169.7
69.1
65.6
39.5 DMSO-d6
15.4
f1 (ppm)

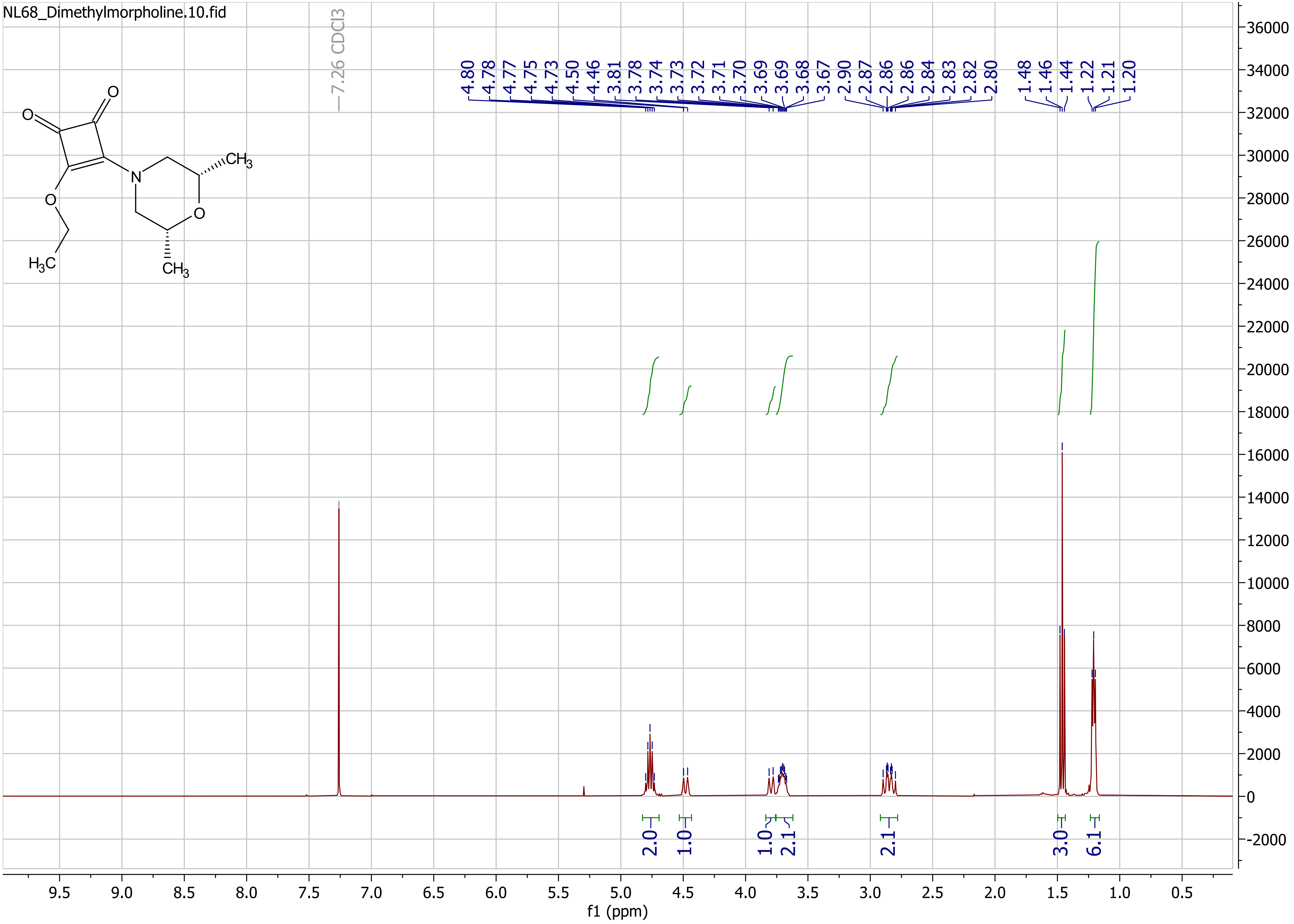

NL68_Dimethylmorpholine.10.fid
7.26 CDCl3
4.80 4.78 4.77 4.75 4.73 4.50 4.46 3.81 3.78 3.74 3.73 3.72 3.71 3.70 3.69 3.69 3.68 3.67 2.90 2.87 2.86 2.86 2.84 2.83 2.82 2.80 1.48 1.46 1.44 1.22 1.21 1.20
2.0 1.0 1.0 2.1 2.1 3.0 6.1
f1 (ppm)

NL68 - Dimethylmorpholine CNMR.10.fid

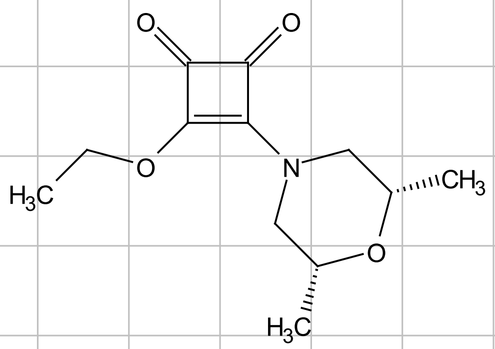

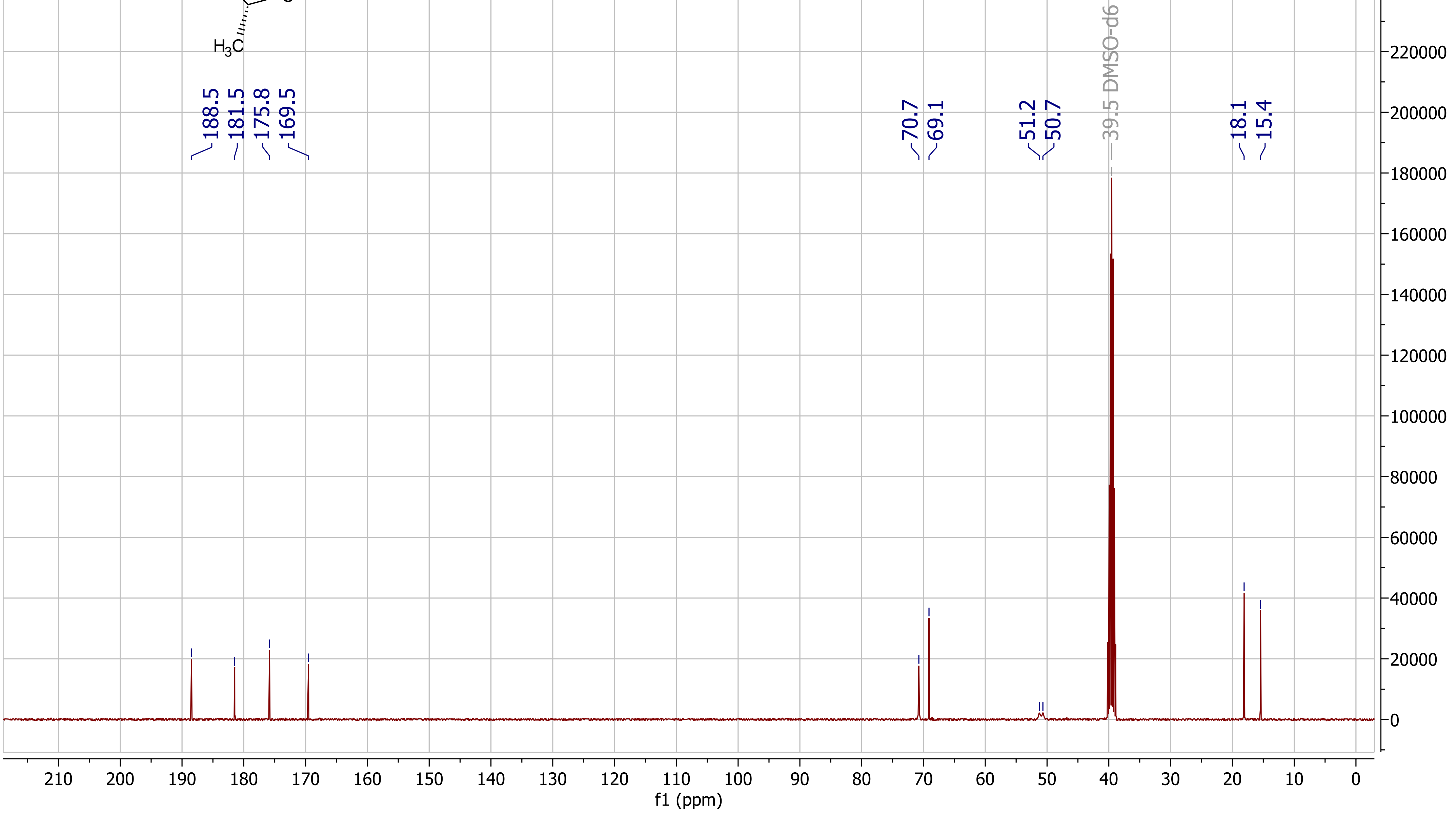

NL70_Thiomorpholine_Purified_HNMR.10.fid

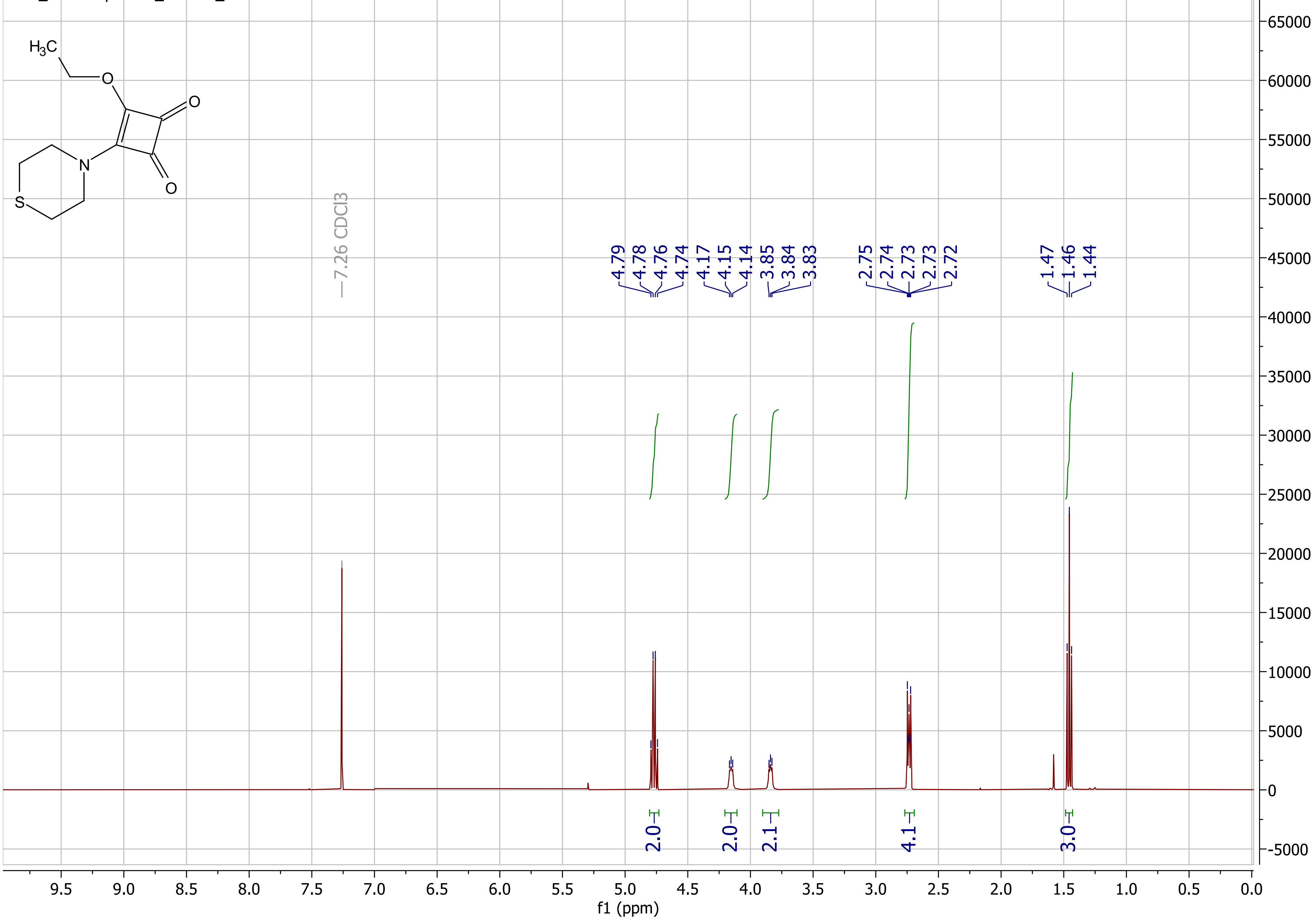

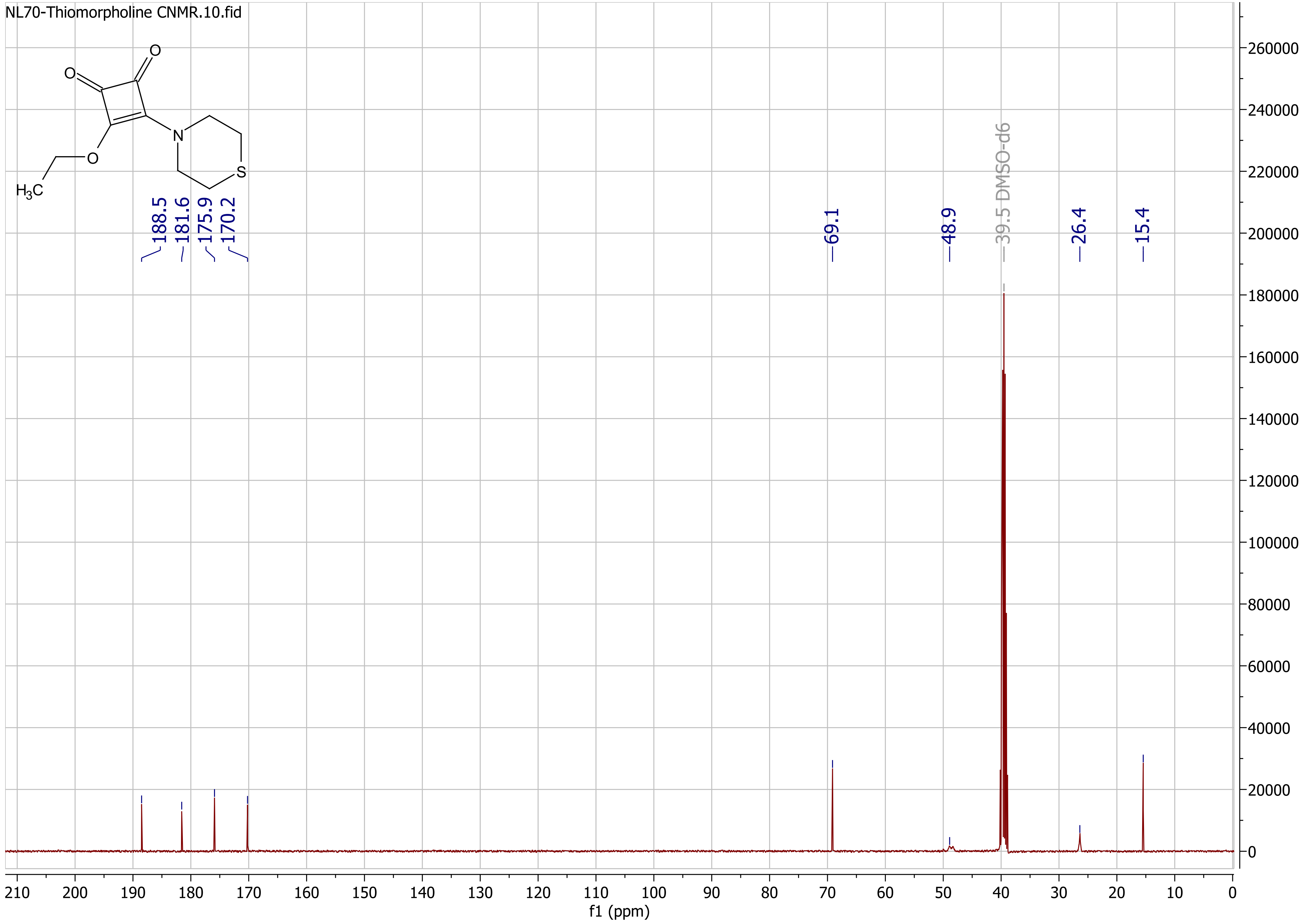
NL70-Thiomorpholine CNMR.10.fid
O
O
N
S
O
H3C
188.5
181.6
175.9
170.2
69.1
48.9
39.5 DMSO-d6
26.4
15.4
f1 (ppm)

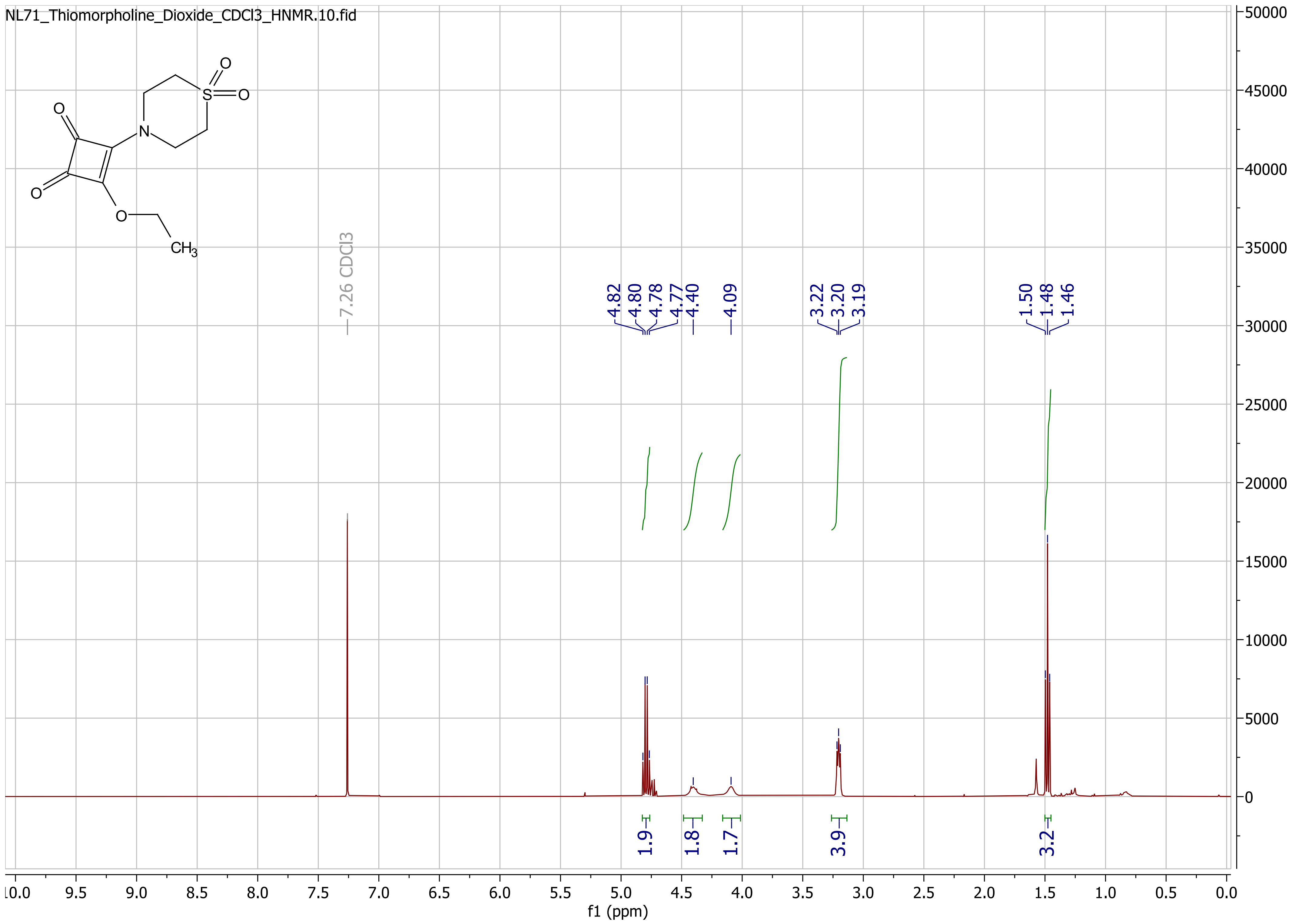

NL71_Thiomorpholine_Dioxide_CDCl3_HNMR.10.fid
7.26 CDCl3
4.82
4.80
4.78
4.77
4.40
4.09
3.22
3.20
3.19
1.50
1.48
1.46
1.9
1.8
1.7
3.9
3.2
f1 (ppm)

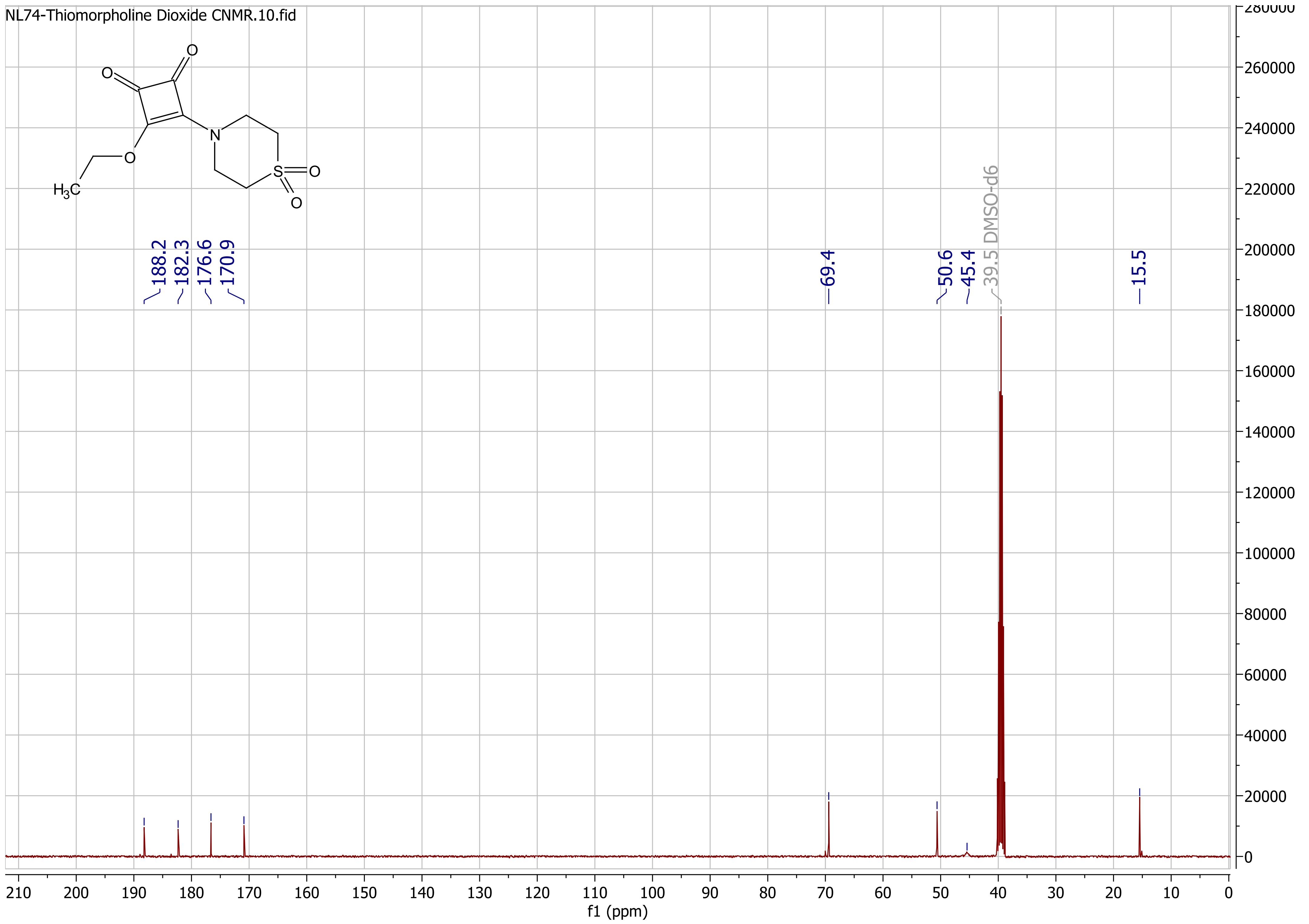
NL74-Thiomorpholine Dioxide CNMR.10.fid
O
O
N
O
S
O
O
H_3C
188.2
182.3
176.6
170.9
69.4
50.6
45.4
39.5 DMSO-d6
15.5
f1 (ppm)
210
200
190
180
170
160
150
140
130
120
110
100
90
80
70
60
50
40
30
20
10
0
280000
260000
240000
220000
200000
180000
160000
140000
120000
100000
80000
60000
40000
20000
0

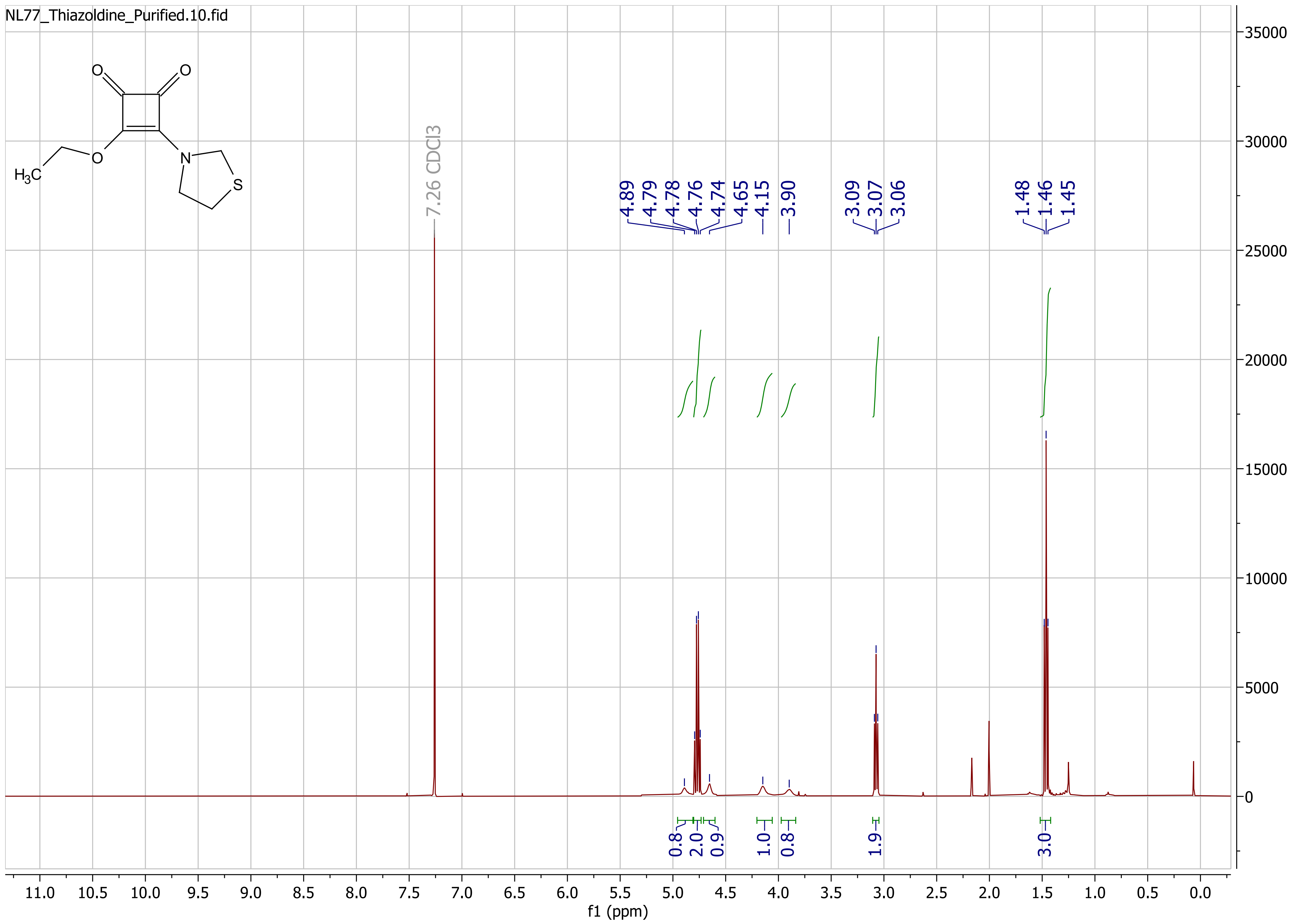

NL77_Thiazoldine_Purified.10.fid
O
O
H3C
O
N
S
7.26 CDCl3
4.89
4.79
4.78
4.76
4.74
4.65
4.15
3.90
3.09
3.07
3.06
1.48
1.46
1.45
0.8
2.0
0.9
1.0
0.8
1.9
3.0
35000
30000
25000
20000
15000
10000
5000
0
11.0
10.5
10.0
9.5
9.0
8.5
8.0
7.5
7.0
6.5
6.0
5.5
5.0
4.5
4.0
3.5
3.0
2.5
2.0
1.5
1.0
0.5
0.0
f1 (ppm)

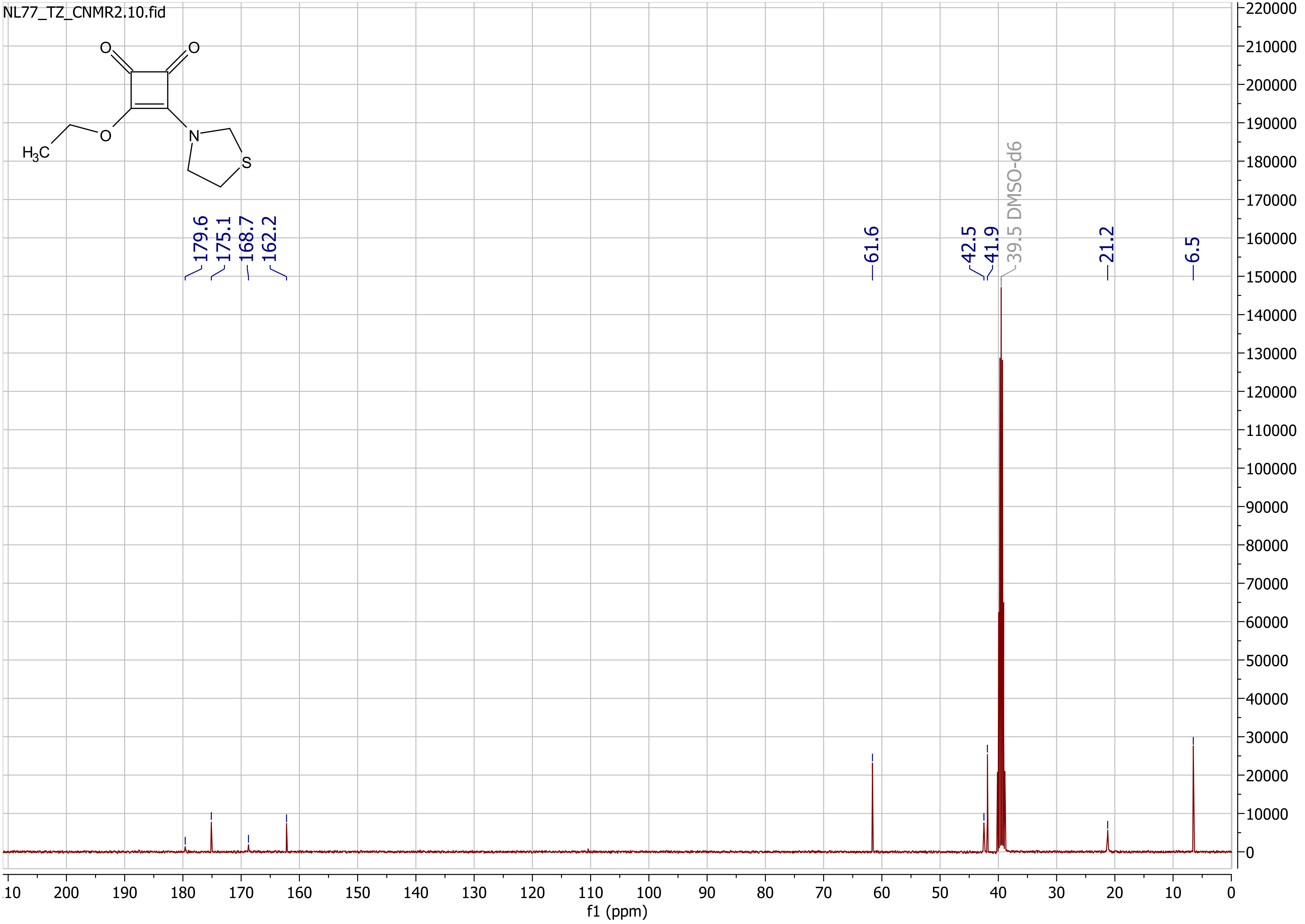
NL77_TZ_CNMR2.10.fid
H3C
O
O
O
N
S
179.6
175.1
168.7
162.2
61.6
42.5
41.9
39.5 DMSO-d6
21.2
6.5
f1 (ppm)

NL73_BocPiperazine_HNMR.10.fid

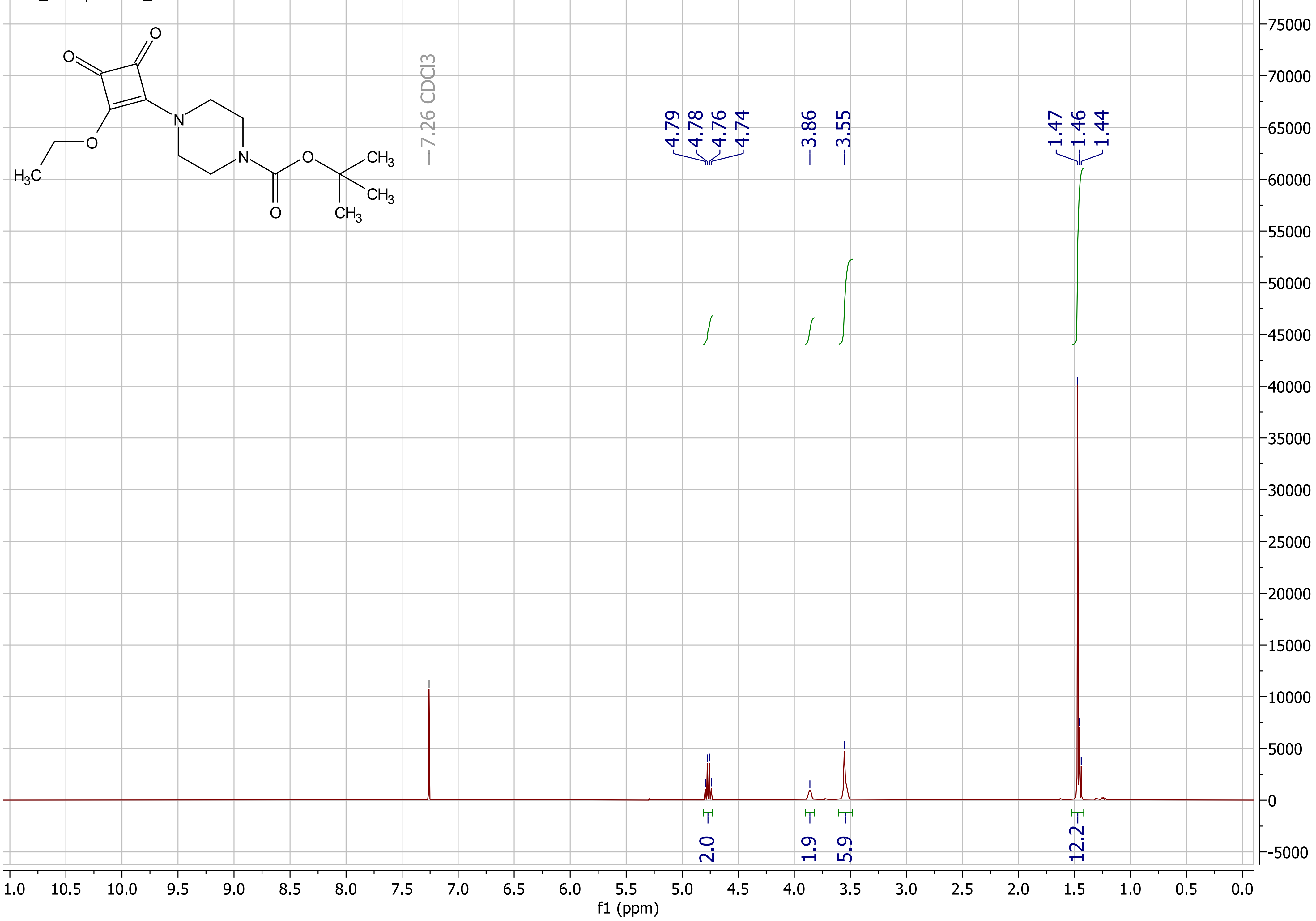

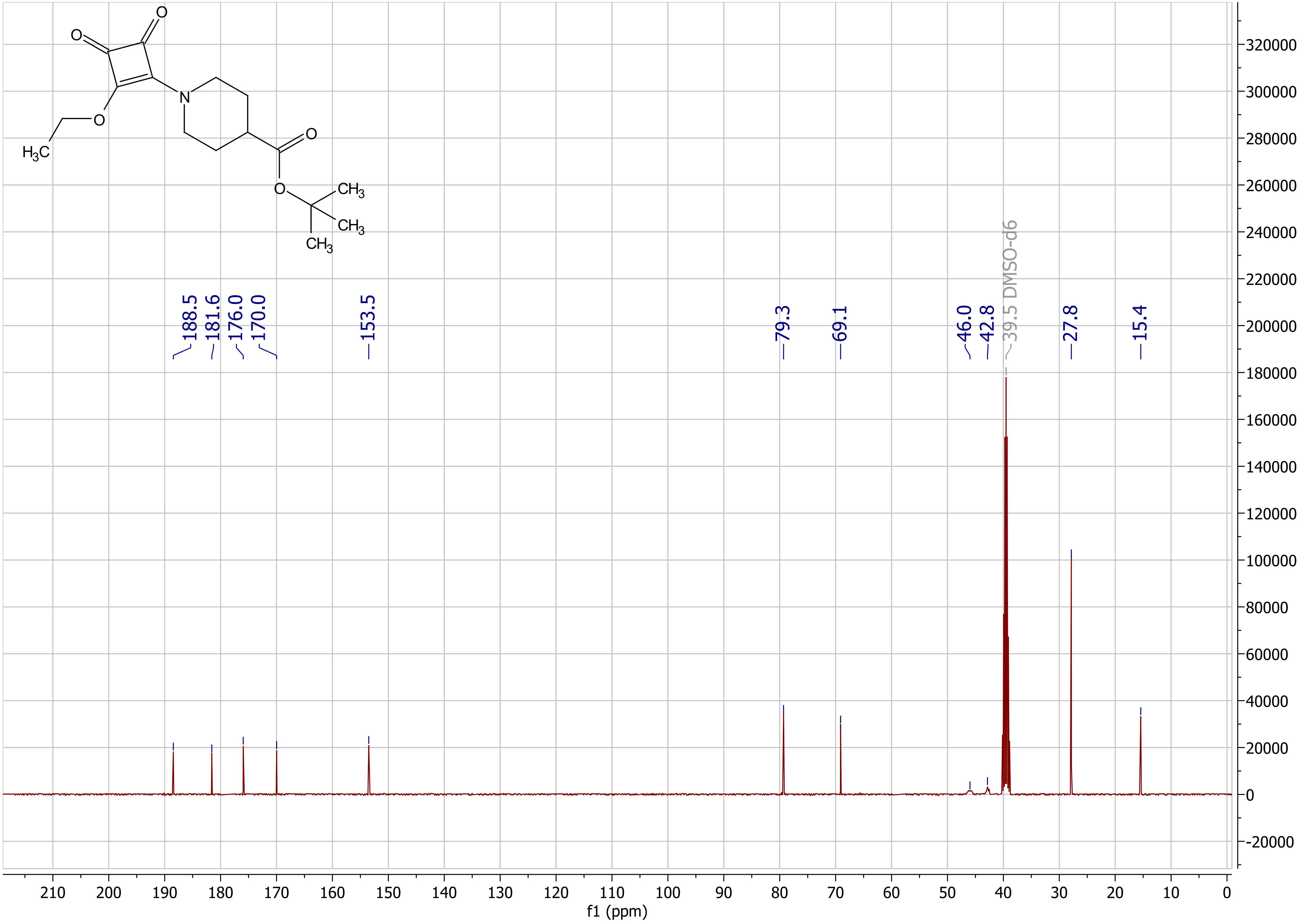

O
O
O
O
O
N
H3C
CH3
CH3
CH3
188.5
181.6
176.0
170.0
153.5
79.3
69.1
46.0
42.8
39.5 DMSO-d6
27.8
15.4
f1 (ppm)

NL67_4_CD2Cl2.10.fid

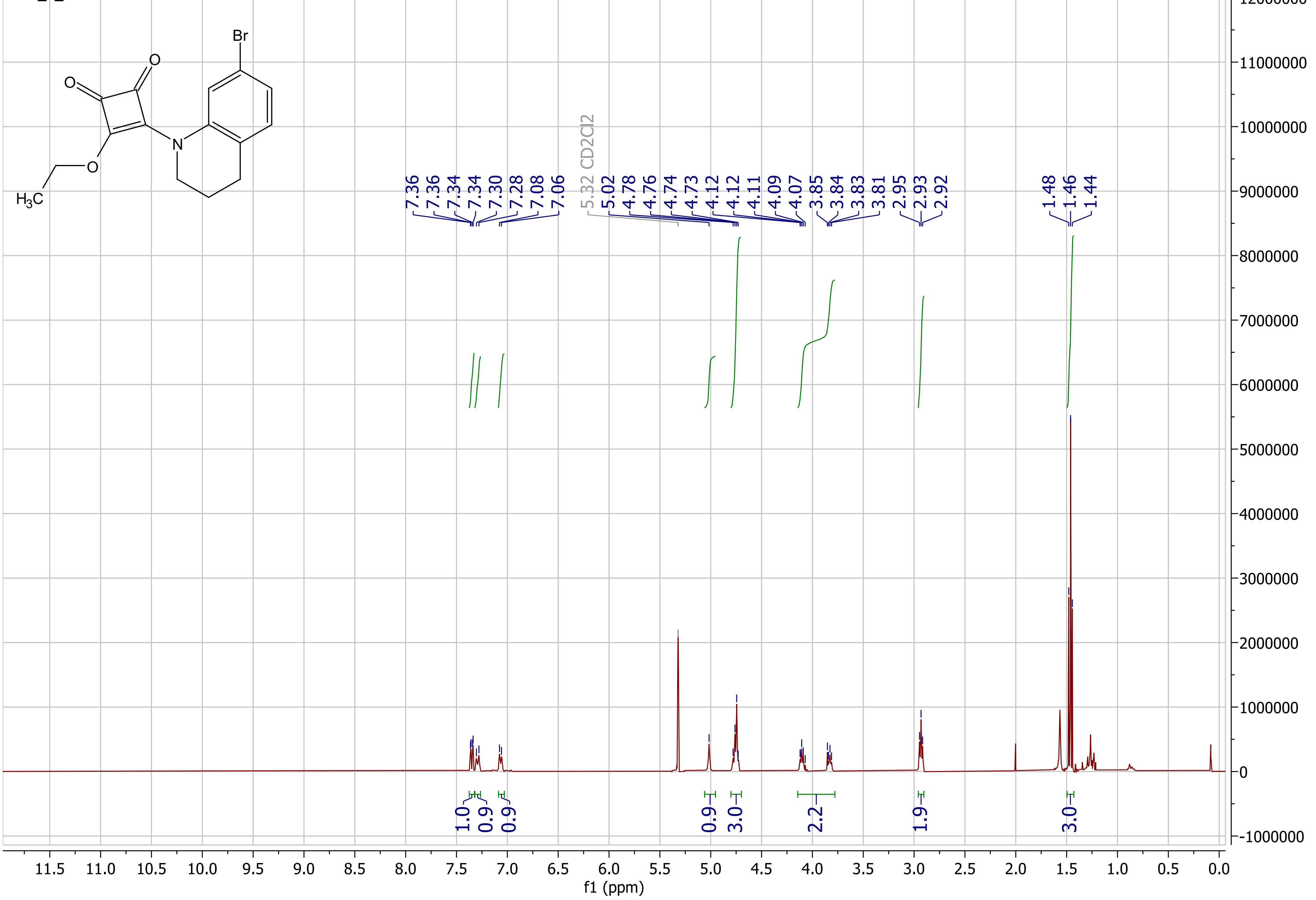

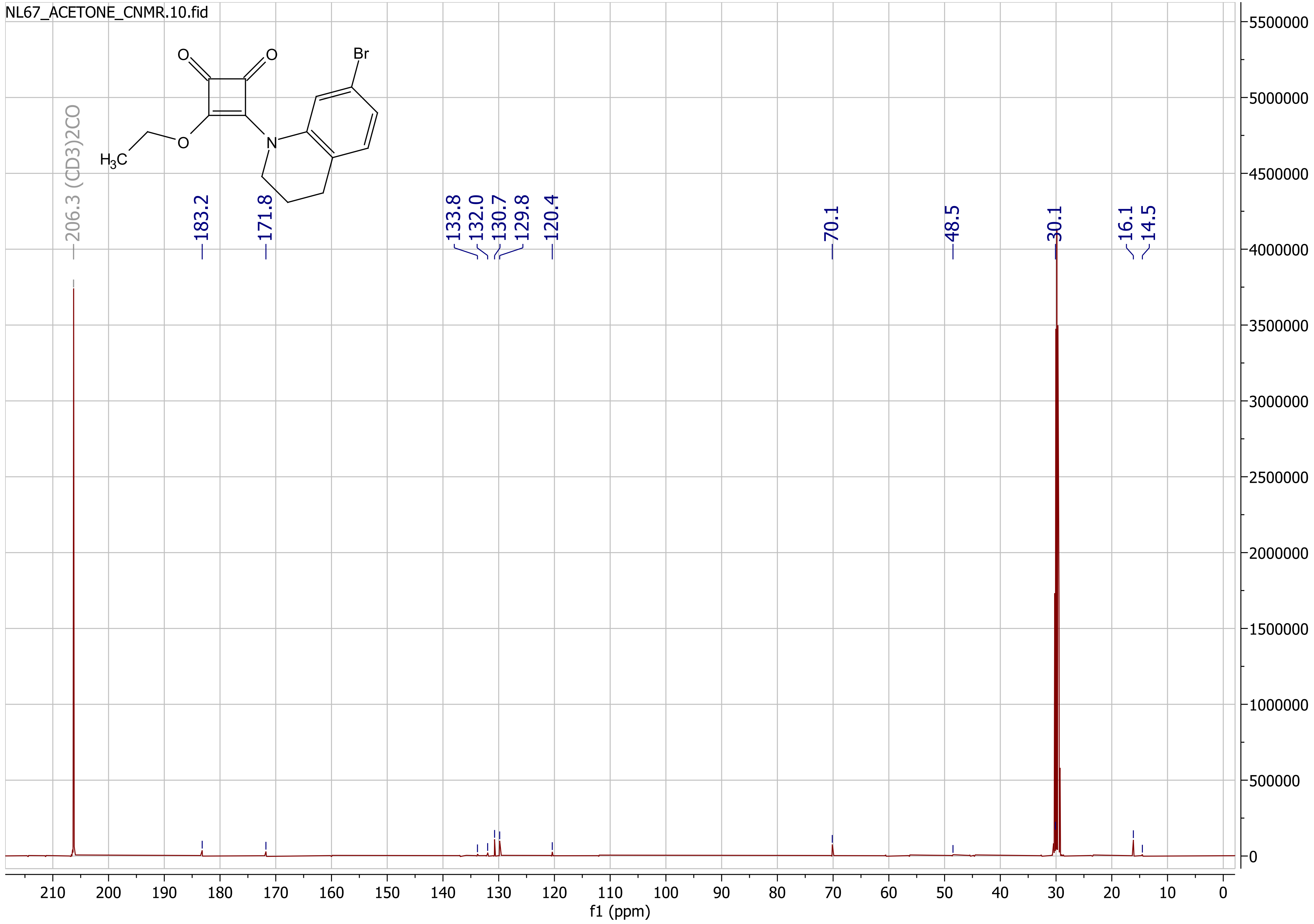
NL67_ACETONE_CNMR.10.fid
O
O
Br
H3C
O
N
206.3 (CD3)2CO
183.2
171.8
133.8
132.0
130.7
129.8
120.4
70.1
48.5
30.1
16.1
14.5
5500000
5000000
4500000
4000000
3500000
3000000
2500000
2000000
1500000
1000000
500000
0
210
200
190
180
170
160
150
140
130
120
110
100
90
80
70
60
50
40
30
20
10
0
f1 (ppm)

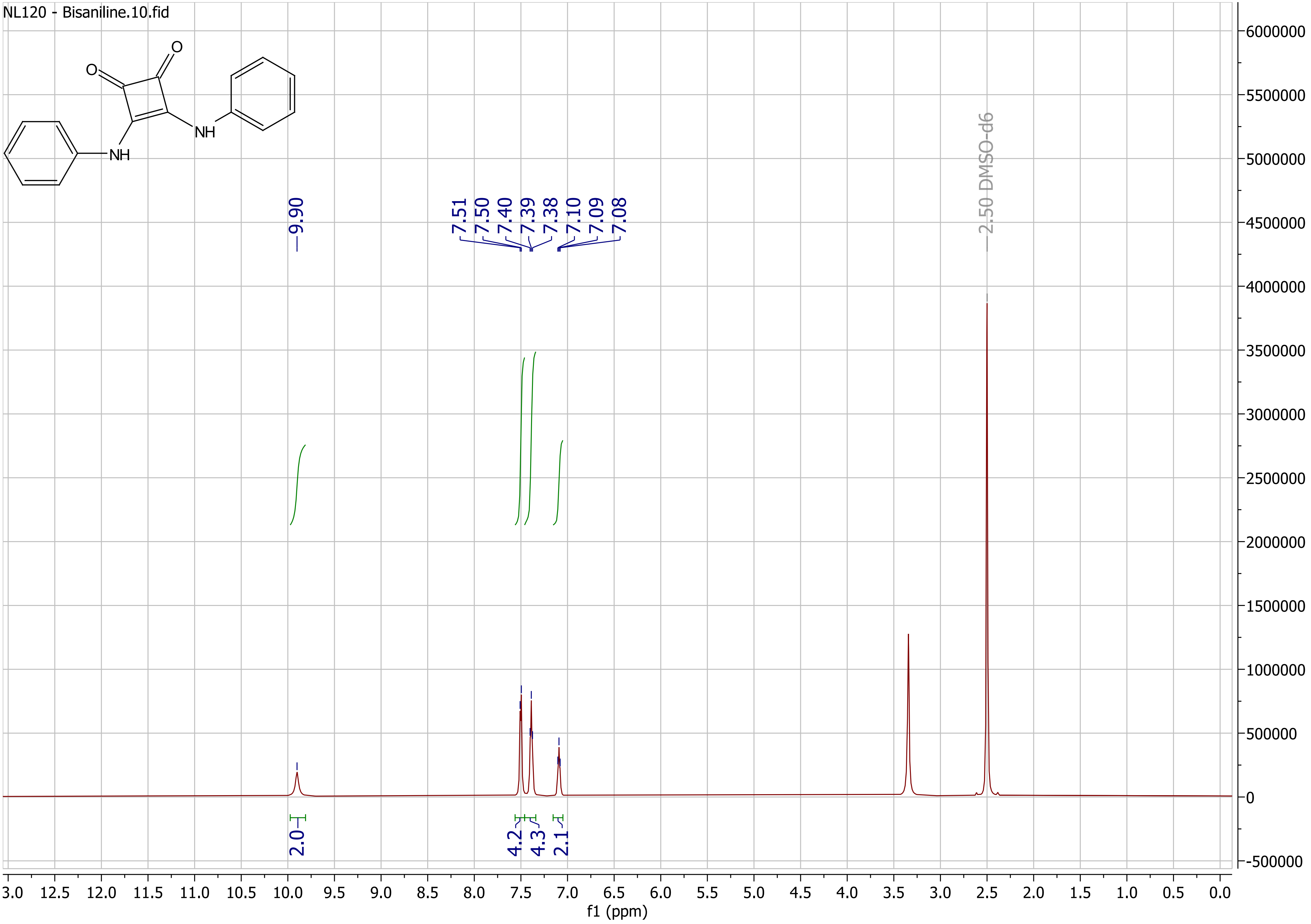
NL120 - Bisaniline.10.fid
O
O
NH
NH
9.90
7.51
7.50
7.40
7.39
7.38
7.10
7.09
7.08
2.50 DMSO-d6
2.0
4.2
4.3
2.1
f1 (ppm)
6000000
5500000
5000000
4500000
4000000
3500000
3000000
2500000
2000000
1500000
1000000
500000
0
-500000
13.0 12.5 12.0 11.5 11.0 10.5 10.0 9.5 9.0 8.5 8.0 7.5 7.0 6.5 6.0 5.5 5.0 4.5 4.0 3.5 3.0 2.5 2.0 1.5 1.0 0.5 0.0

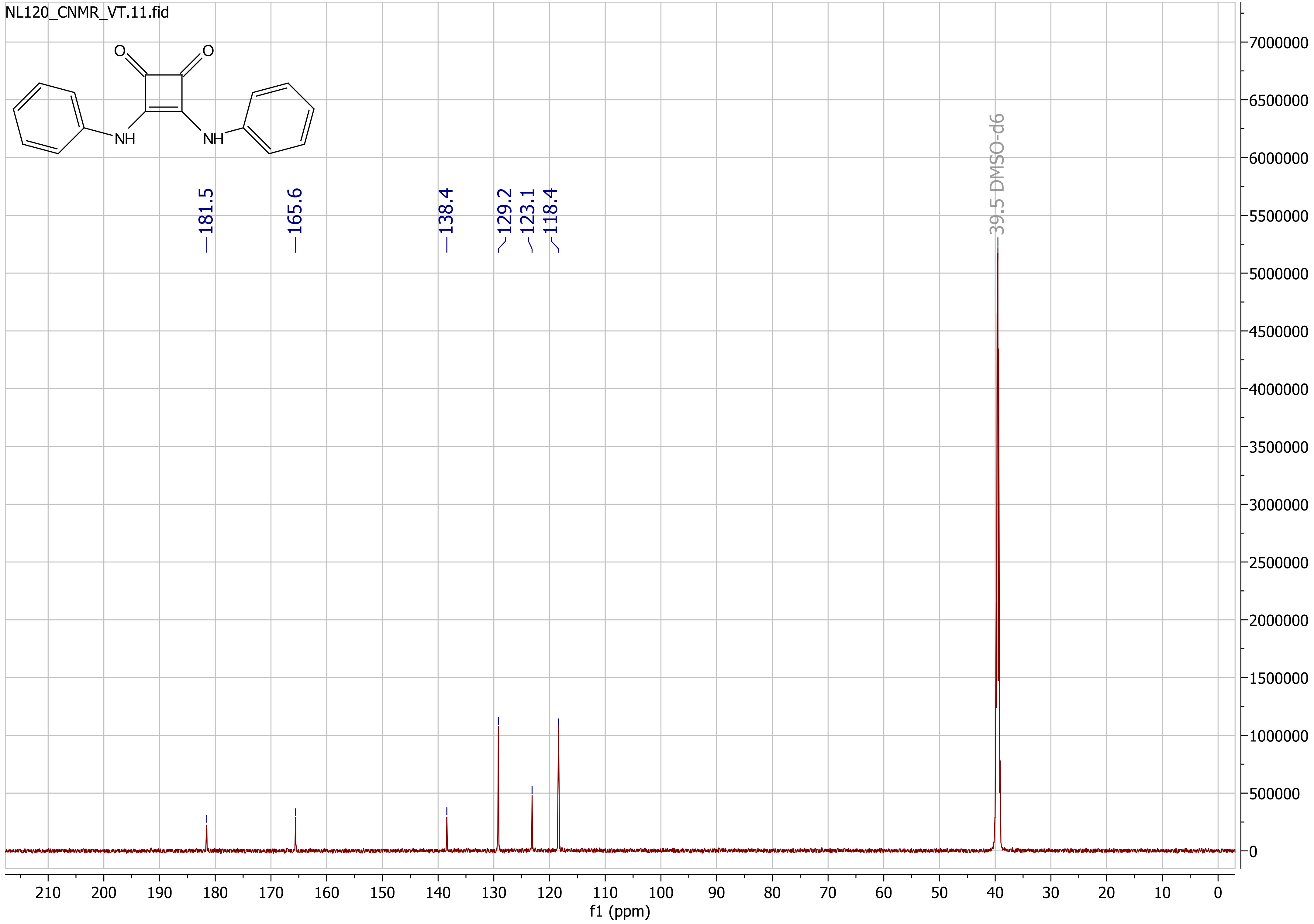
NL120_CNMR_VT.11.fid
O
O
NH
NH
181.5
165.6
138.4
129.2
123.1
118.4
39.5 DMSO-d6
f1 (ppm)

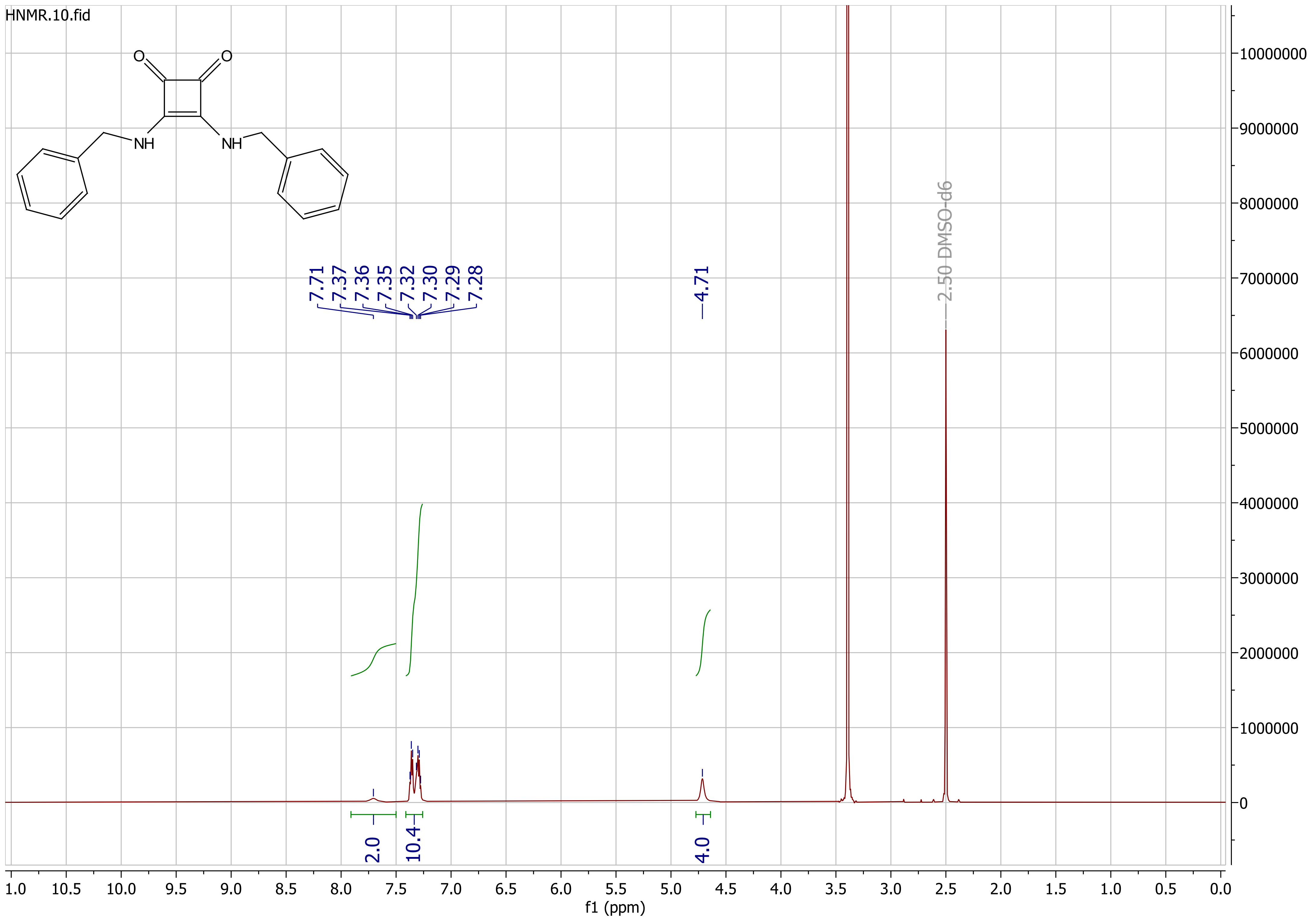

HNMR.10.fid
O
O
NH
NH
7.71
7.37
7.36
7.35
7.32
7.30
7.29
7.28
4.71
2.50 DMSO-d6
2.0
10.4
4.0
f1 (ppm)

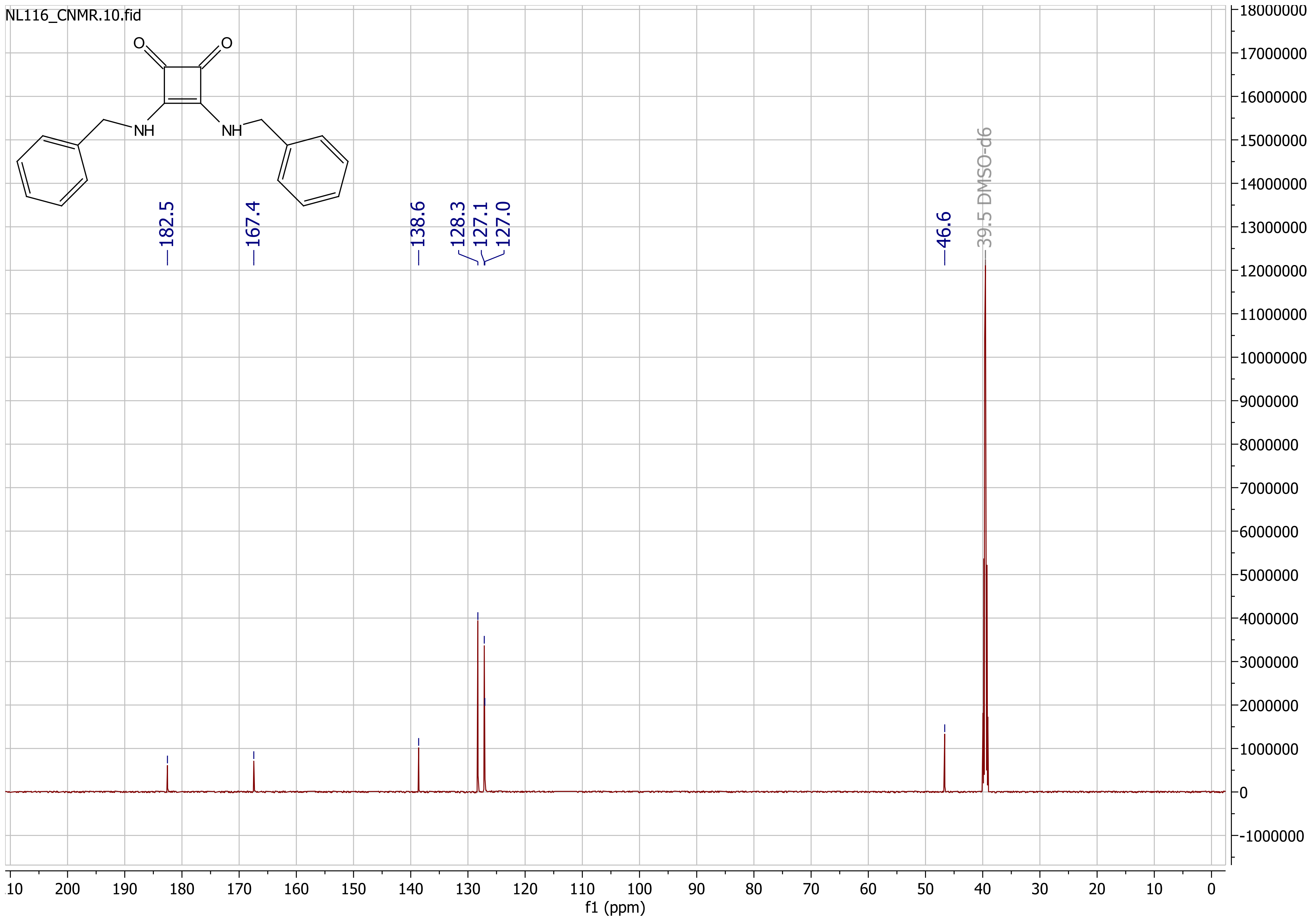
NL116_CNMR.10.fid
O
O
NH
NH
182.5
167.4
138.6
128.3
127.1
127.0
46.6
39.5 DMSO-d6
f1 (ppm)

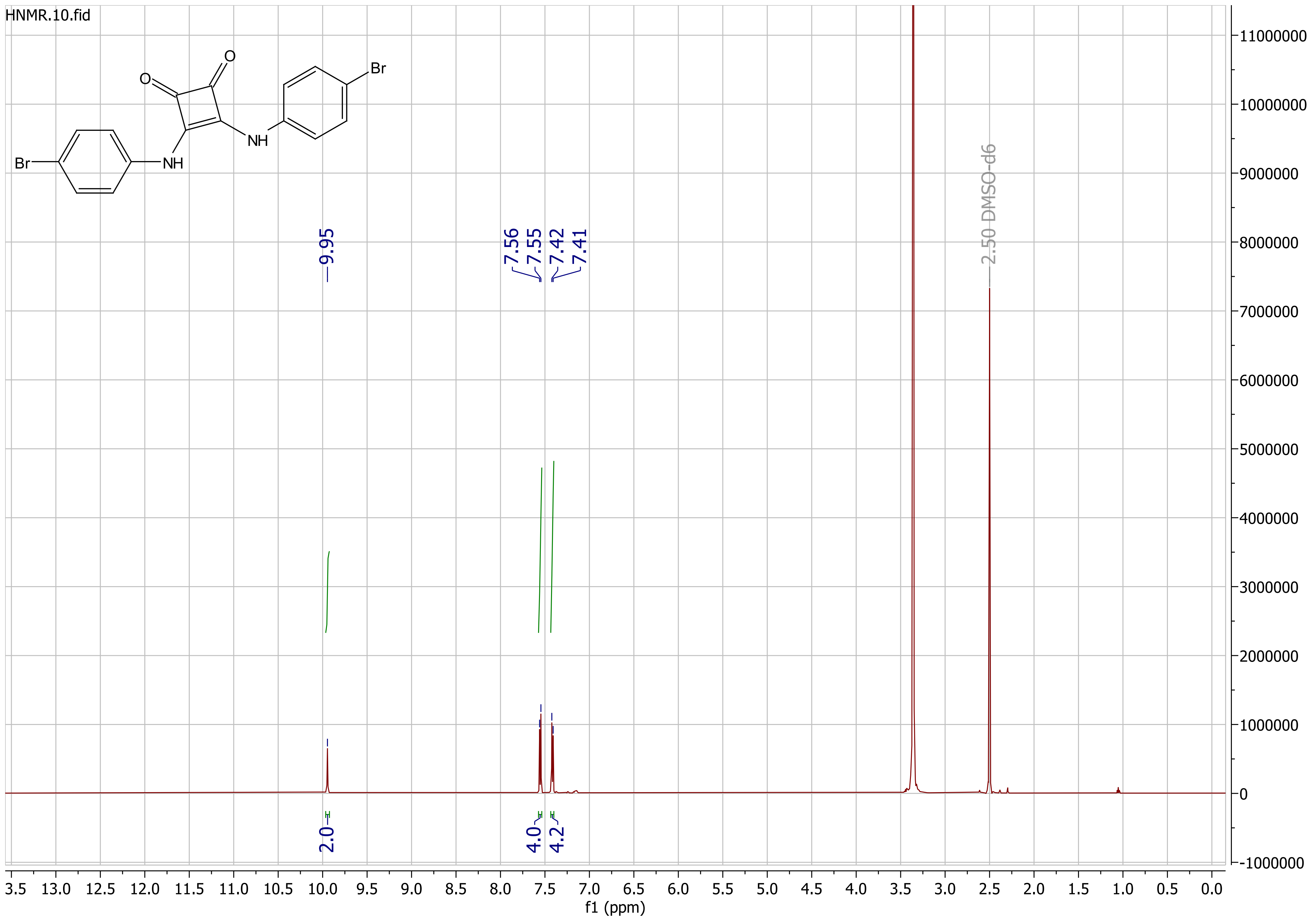

HNMR.10.fid
O
O
Br
NH
Br
NH
9.95
7.56
7.55
7.42
7.41
2.50 DMSO-d6
2.0
4.0
4.2
f1 (ppm)

NL135_CNMR.10.fid

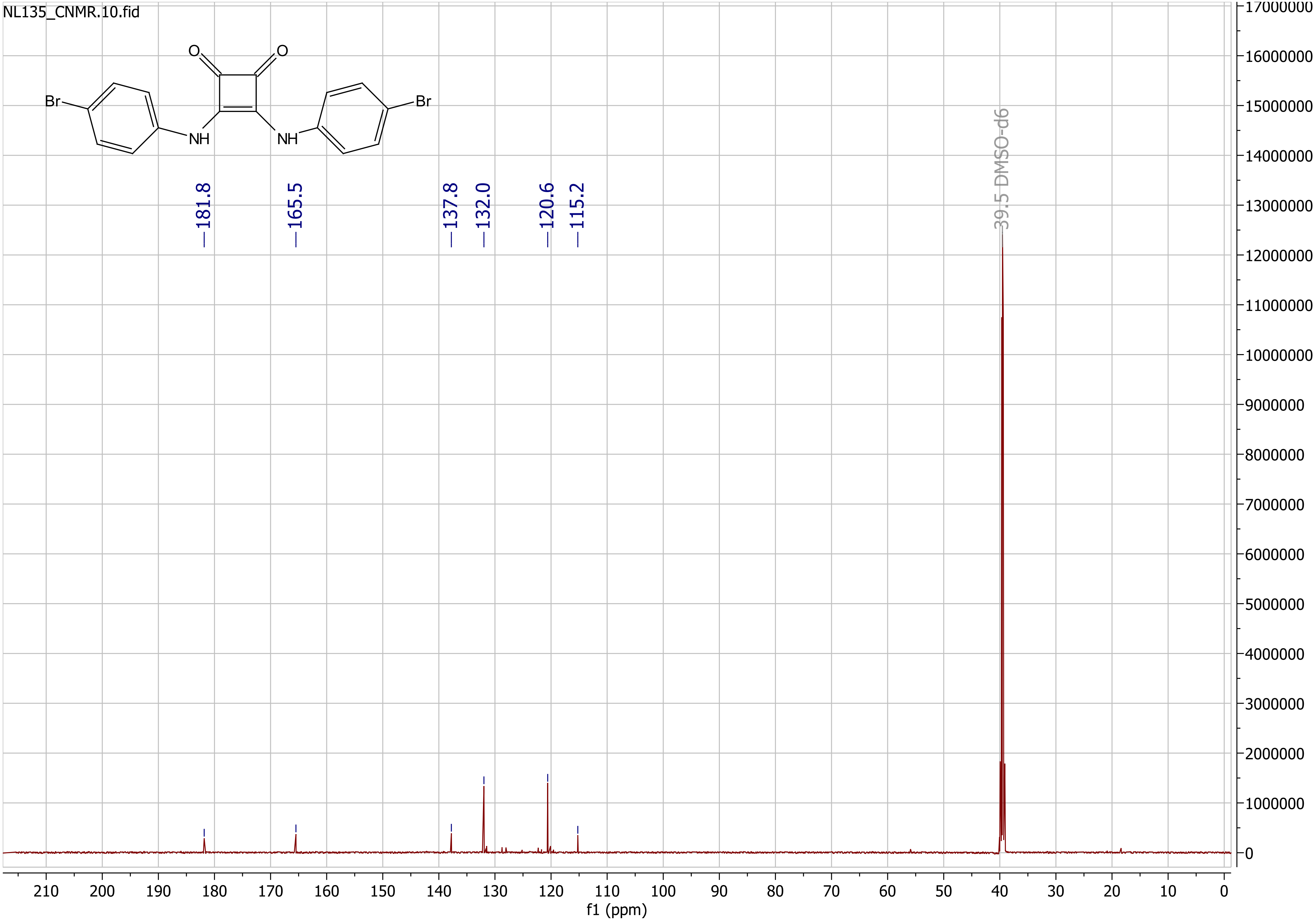

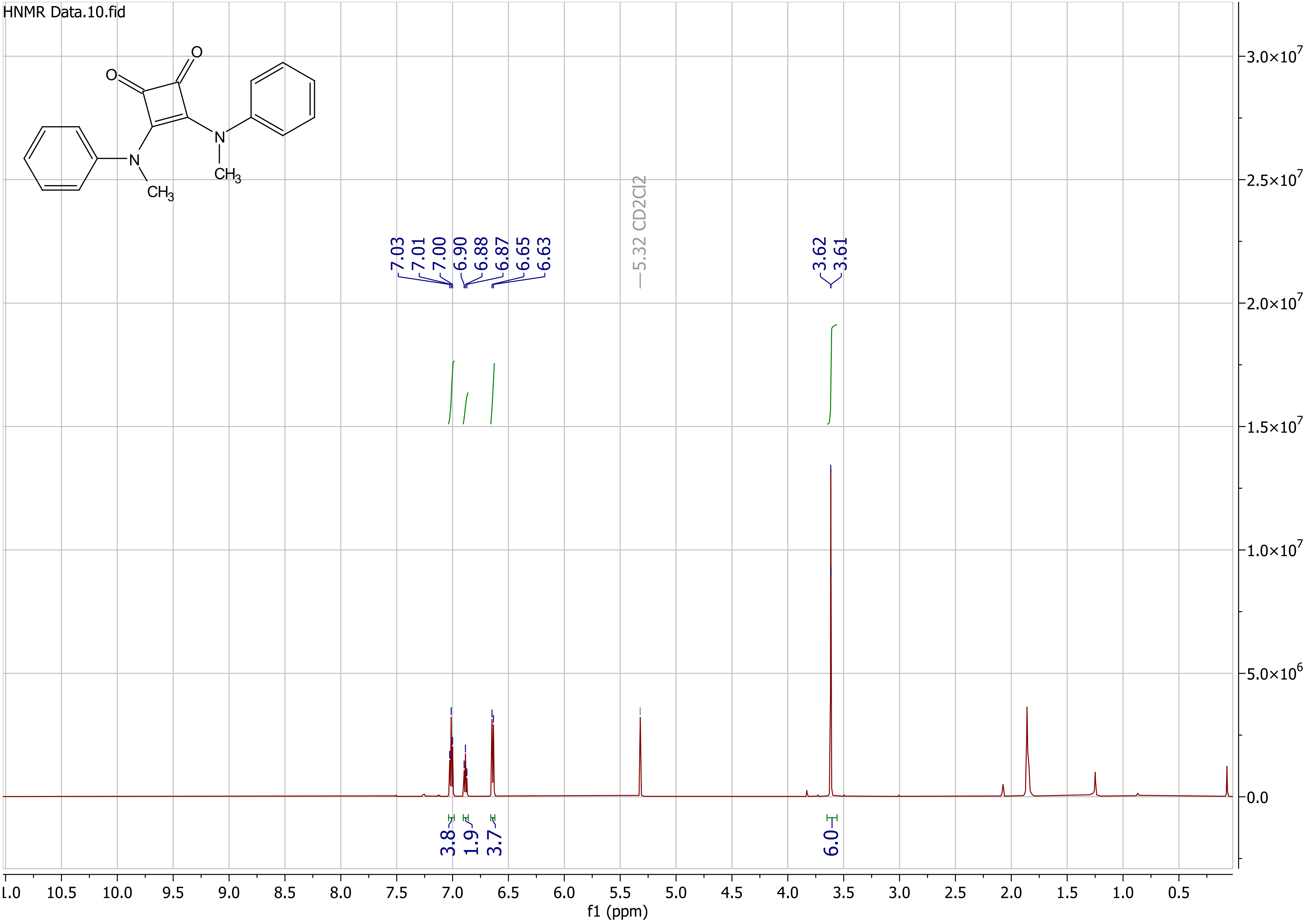
HNMR Data.10.fid
O
O
N
CH3
N
CH3
7.03
7.01
7.00
6.90
6.88
6.87
6.65
6.63
5.32 CD2Cl2
3.62
3.61
3.8
1.9
3.7
6.0
3.0×10^7
2.5×10^7
2.0×10^7
1.5×10^7
1.0×10^7
5.0×10^6
0.0
10.5
10.0
9.5
9.0
8.5
8.0
7.5
7.0
6.5
6.0
5.5
5.0
4.5
4.0
3.5
3.0
2.5
2.0
1.5
1.0
0.5
f1 (ppm)

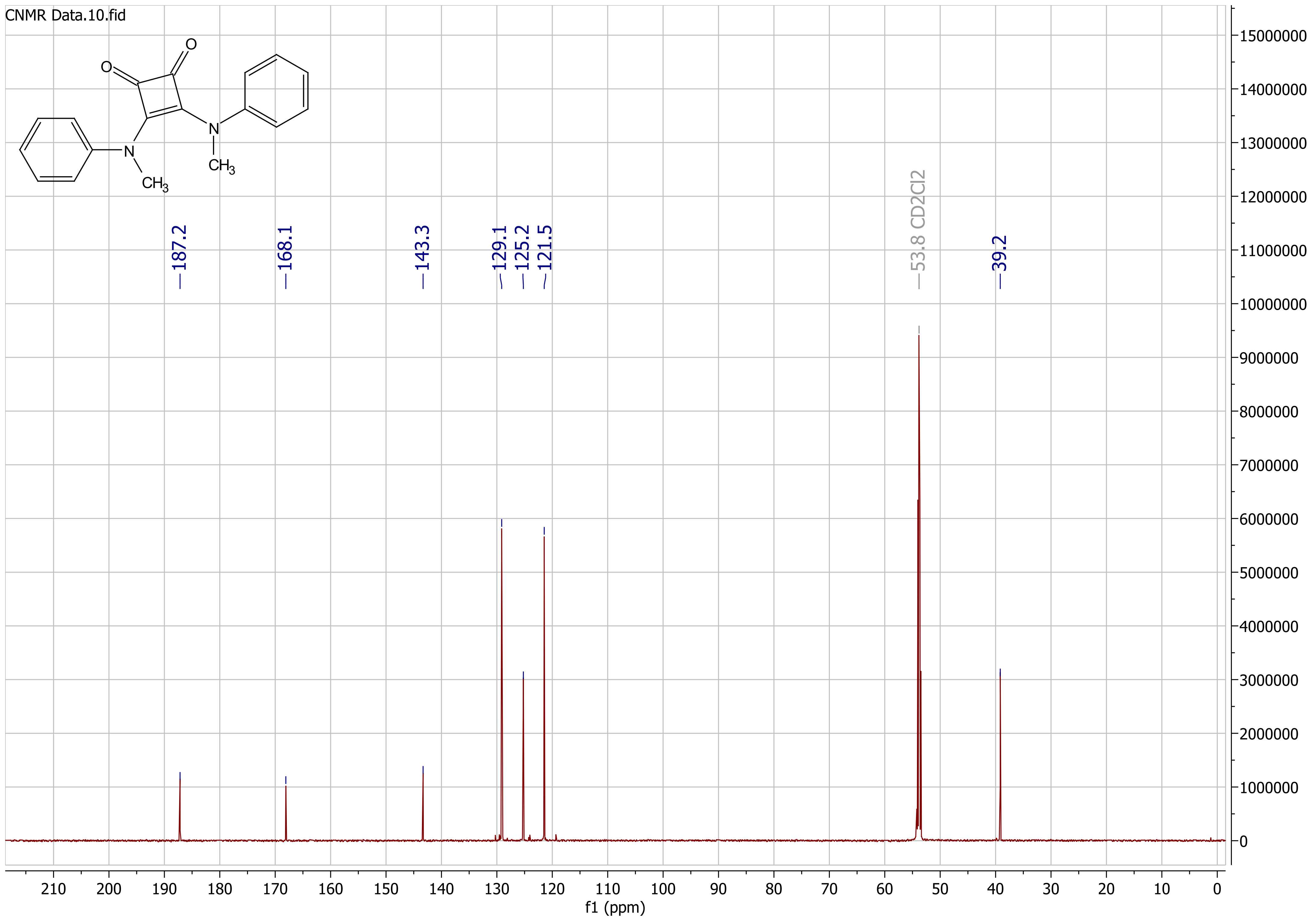

CNMR Data.10.fid
O
O
N
CH3
N
CH3
187.2
168.1
143.3
129.1
125.2
121.5
53.8 CD2Cl2
39.2
f1 (ppm)

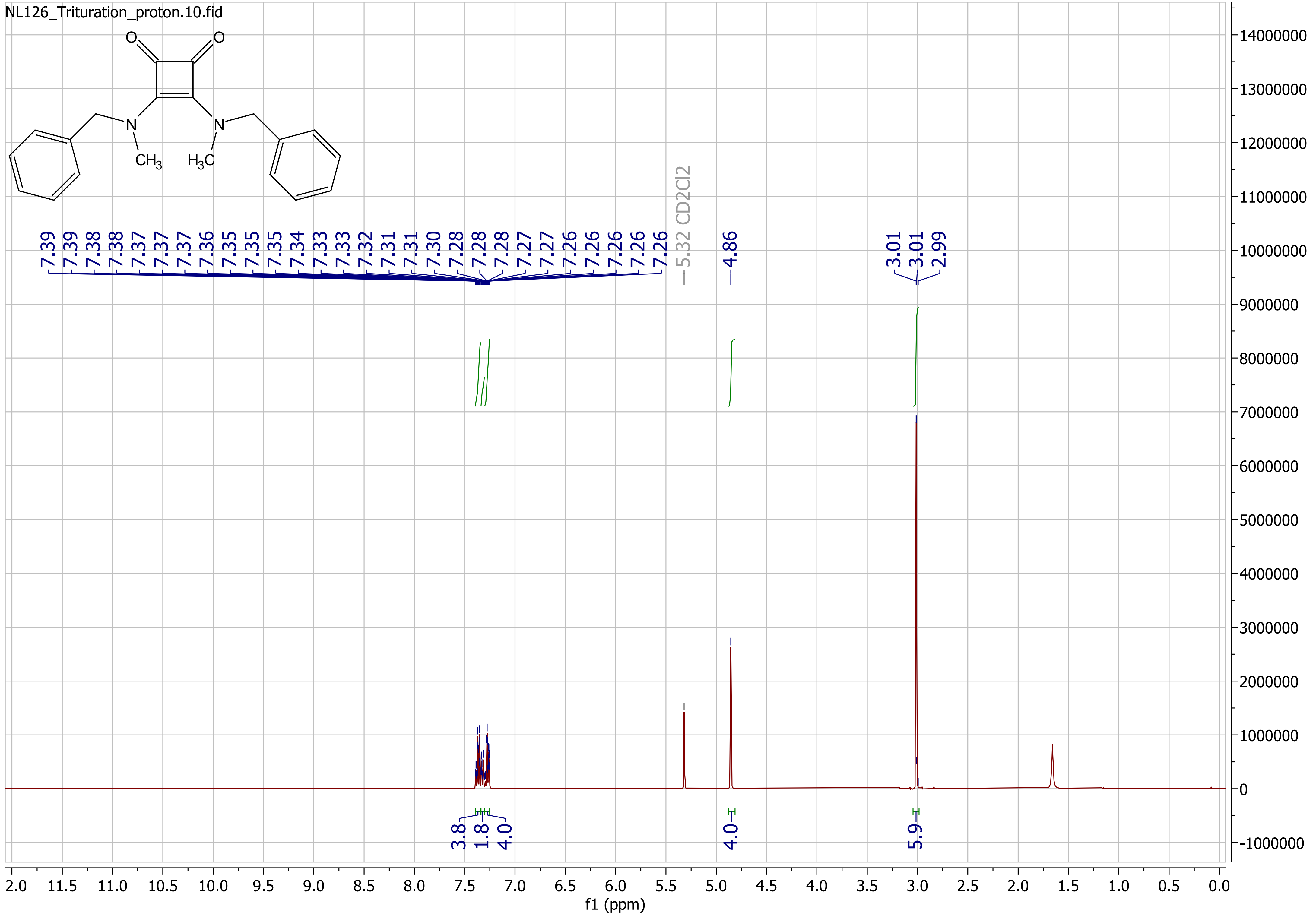

NL126_Trituration_proton.10.fid
O
O
N
N
CH3
H3C
7.39 7.39 7.38 7.38 7.37 7.37 7.37 7.36 7.35 7.35 7.35 7.34 7.33 7.33 7.32 7.31 7.31 7.30 7.28 7.28 7.28 7.27 7.27 7.26 7.26 7.26 7.26 7.26
5.32 CD2Cl2
4.86
3.01 3.01 2.99
3.8 1.8 4.0
4.0
5.9
14000000 13000000 12000000 11000000 10000000 9000000 8000000 7000000 6000000 5000000 4000000 3000000 2000000 1000000 0 -1000000
2.0 11.5 11.0 10.5 10.0 9.5 9.0 8.5 8.0 7.5 7.0 6.5 6.0 5.5 5.0 4.5 4.0 3.5 3.0 2.5 2.0 1.5 1.0 0.5 0.0
f1 (ppm)

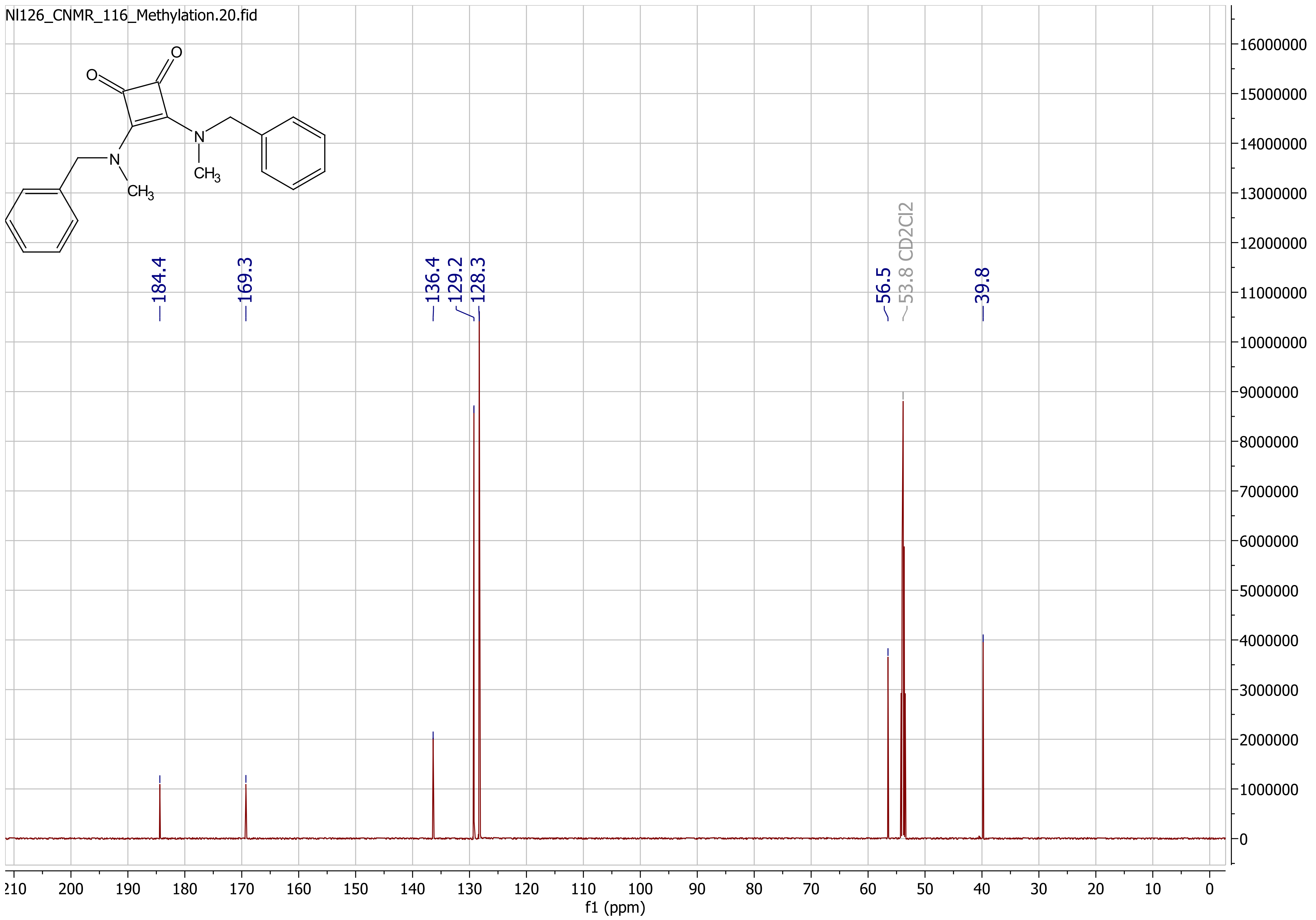

NI126_CNMR_116_Methylation.20.fid
O
O
N
CH3
N
CH3
184.4
169.3
136.4
129.2
128.3
56.5
53.8 CD2Cl2
39.8
f1 (ppm)
210
200
190
180
170
160
150
140
130
120
110
100
90
80
70
60
50
40
30
20
10
0
16000000
15000000
14000000
13000000
12000000
11000000
10000000
9000000
8000000
7000000
6000000
5000000
4000000
3000000
2000000
1000000
0

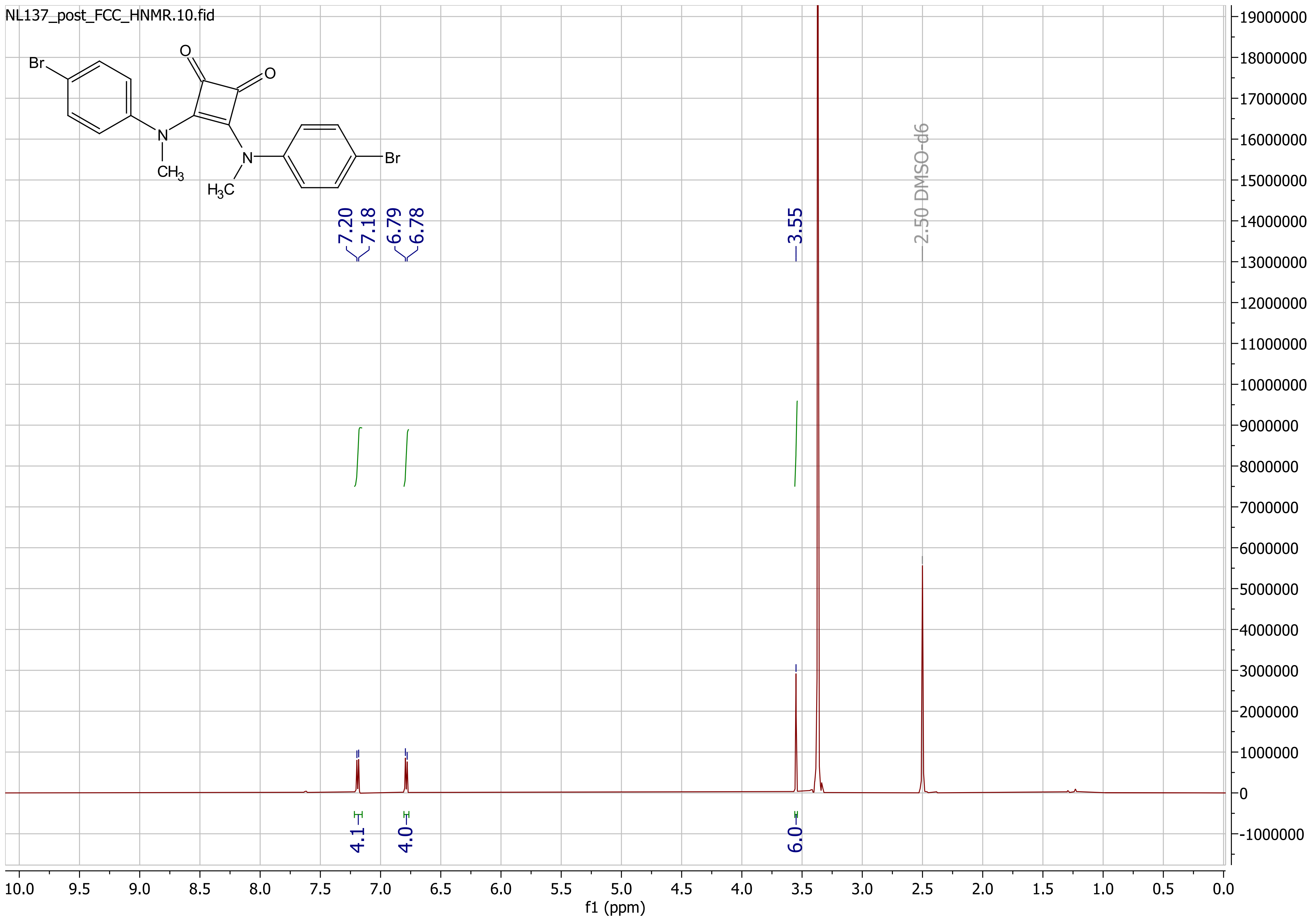
NL137_post_FCC_HNMR.10.fid
Br
O
O
N
CH3
N
Br
H3C
7.20
7.18
6.79
6.78
3.55
2.50 DMSO-d6
4.1
4.0
6.0
f1 (ppm)

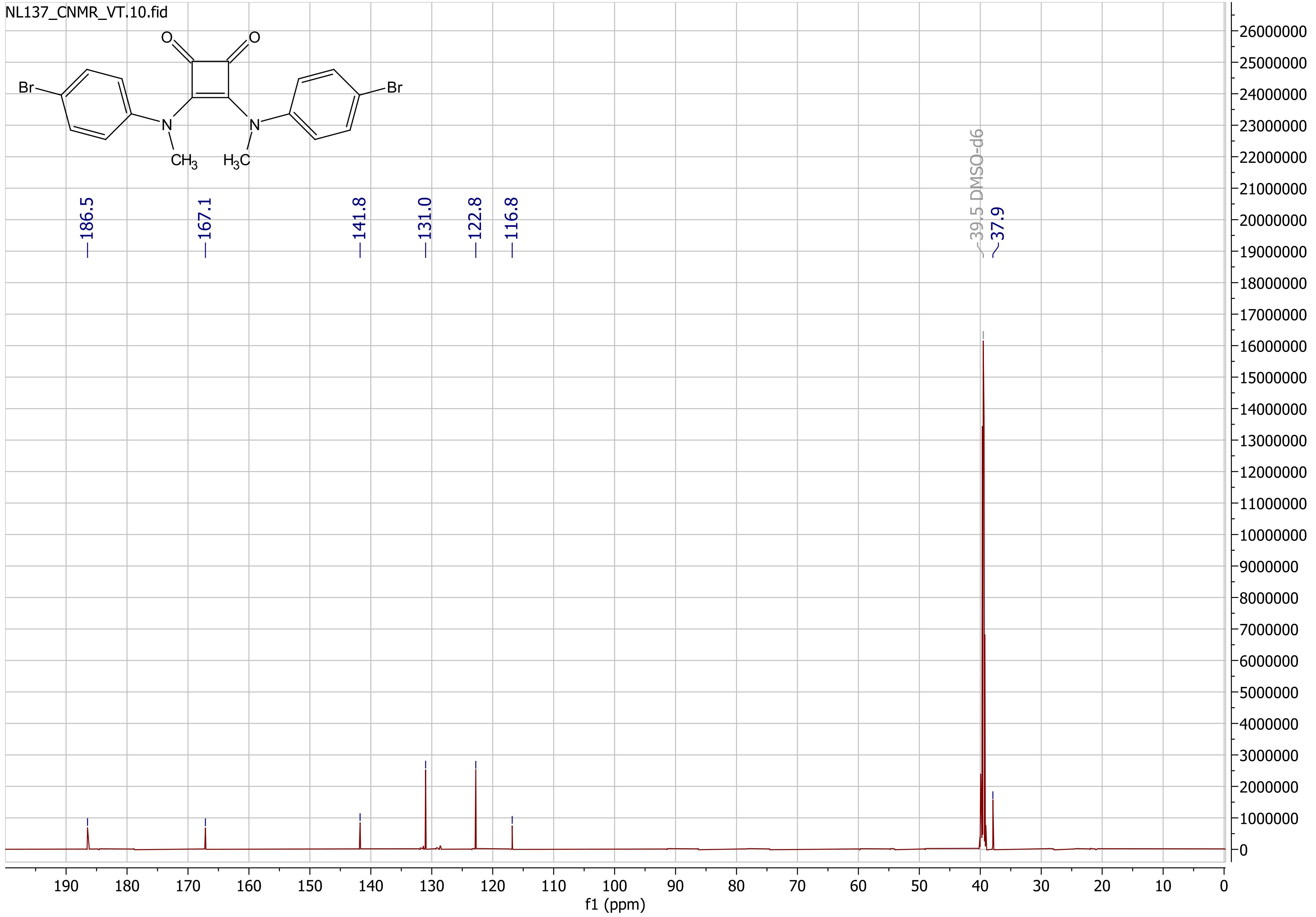
NL137_CNMR_VT.10.fid
186.5
167.1
141.8
131.0
122.8
116.8
39.5 DMSO-d6
37.9
f1 (ppm)

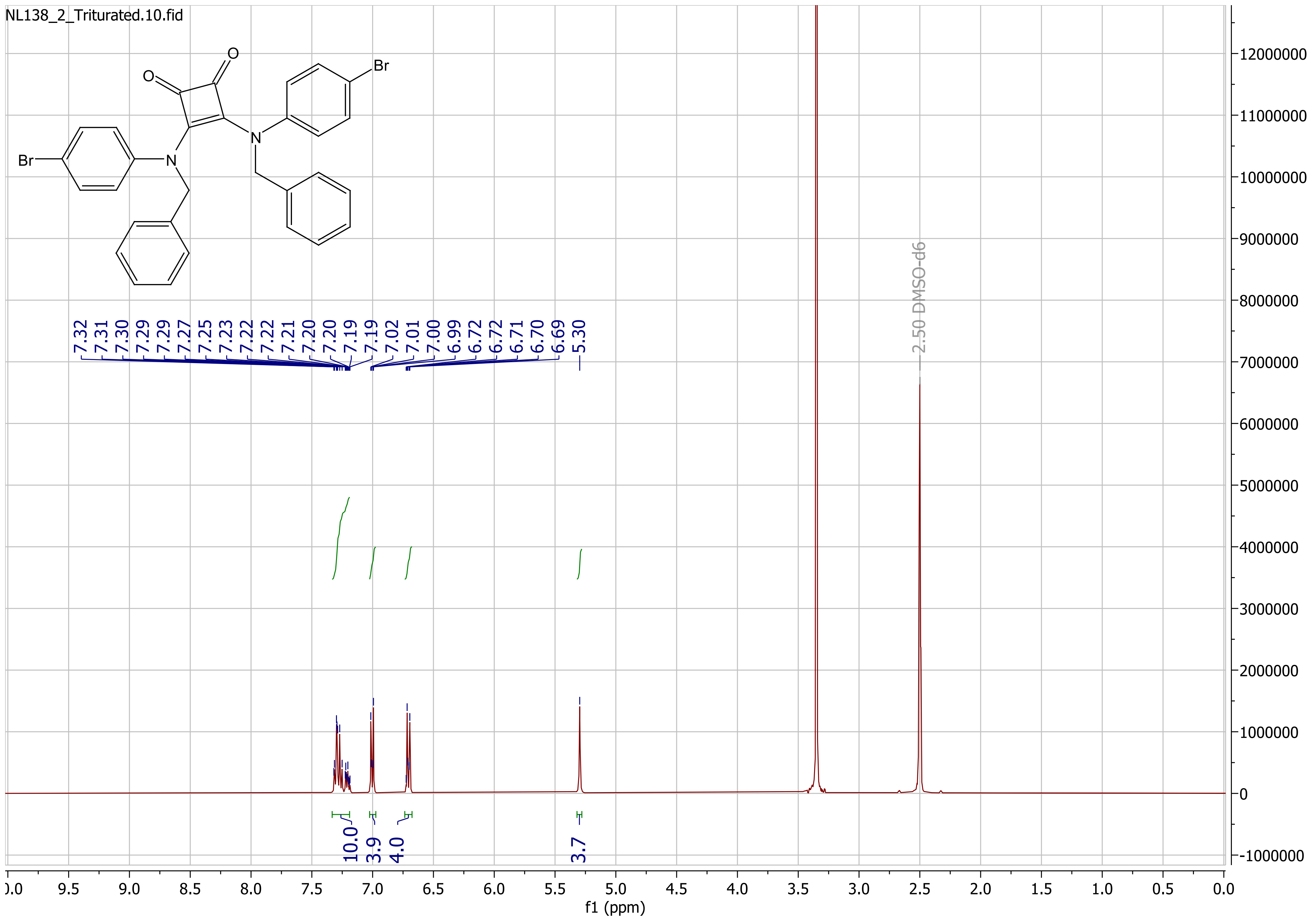
NL138_2_Triturated.10.fid
O
O
Br
Br
N
N
7.32
7.31
7.30
7.29
7.29
7.27
7.25
7.23
7.22
7.22
7.21
7.20
7.20
7.19
7.19
7.02
7.01
7.00
6.99
6.72
6.72
6.71
6.70
6.69
5.30
2.50 DMSO-d6
10.0
3.9
4.0
3.7
f1 (ppm)

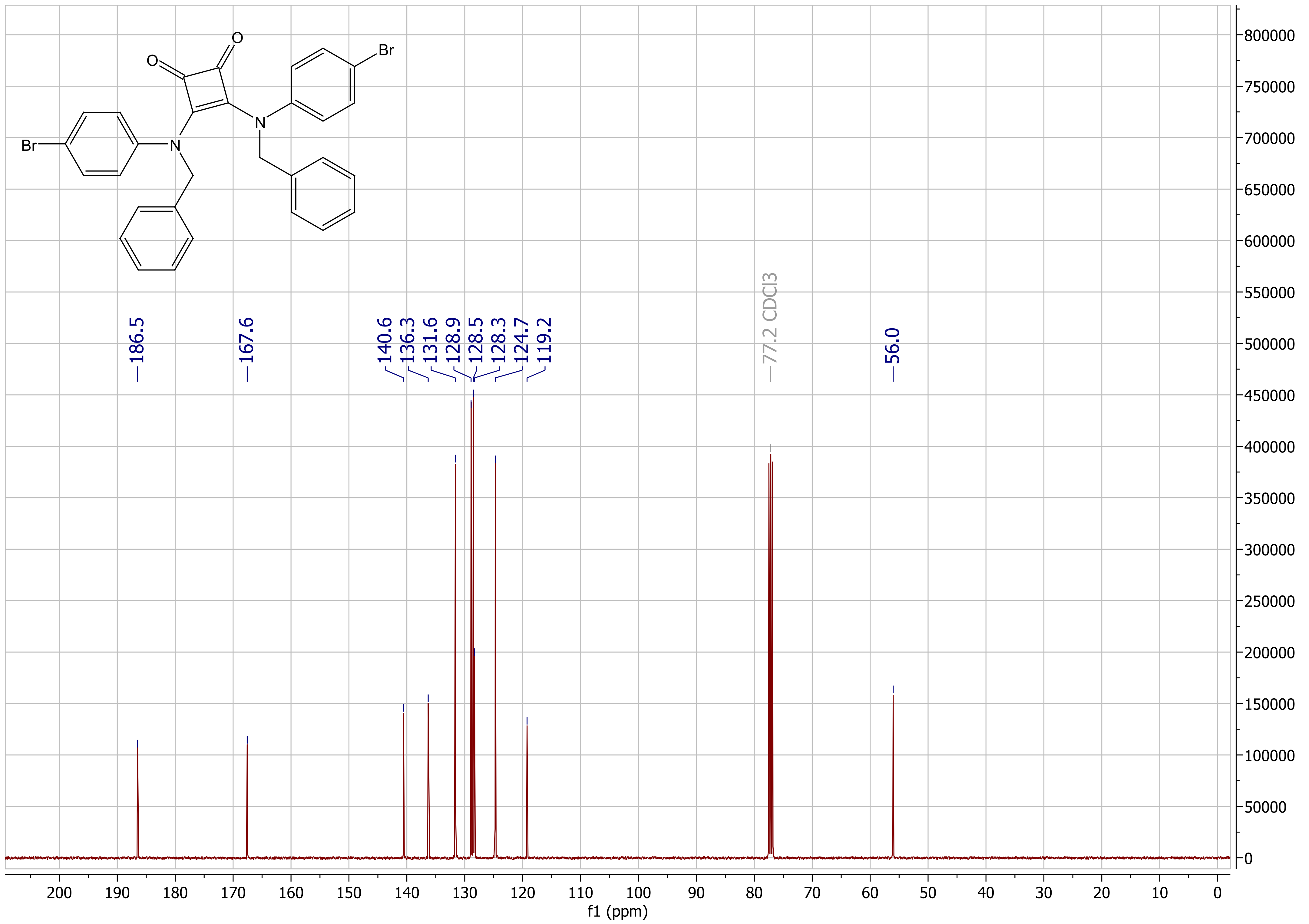

Br
N
O
O
N
Br
186.5
167.6
140.6
136.3
131.6
128.9
128.5
128.3
124.7
119.2
77.2 CDCl3
56.0
f1 (ppm)

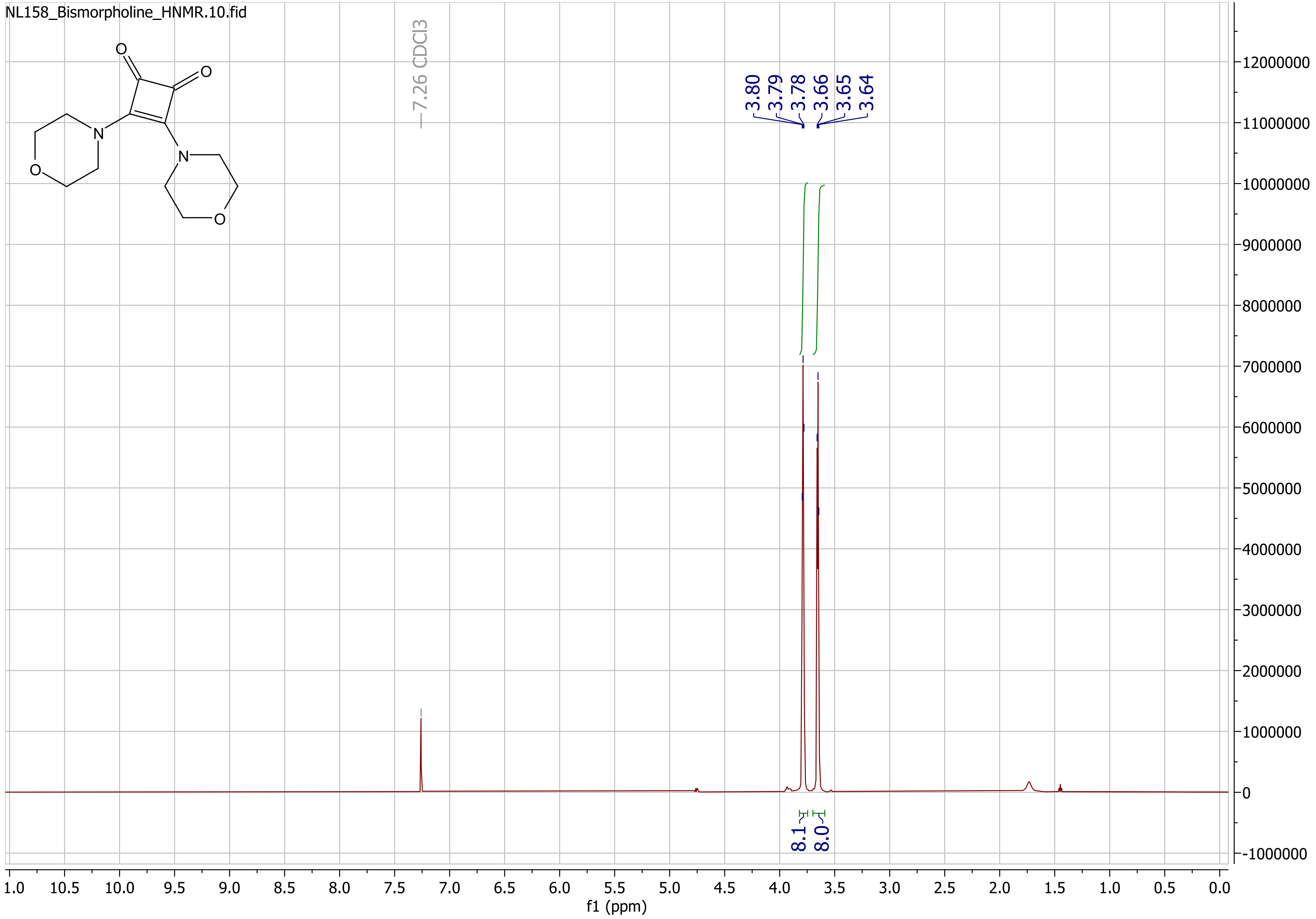
NL158_Bismorpholine_HNMR.10.fid
7.26 CDCl3
3.80
3.79
3.78
3.66
3.65
3.64
8.1
8.0
f1 (ppm)

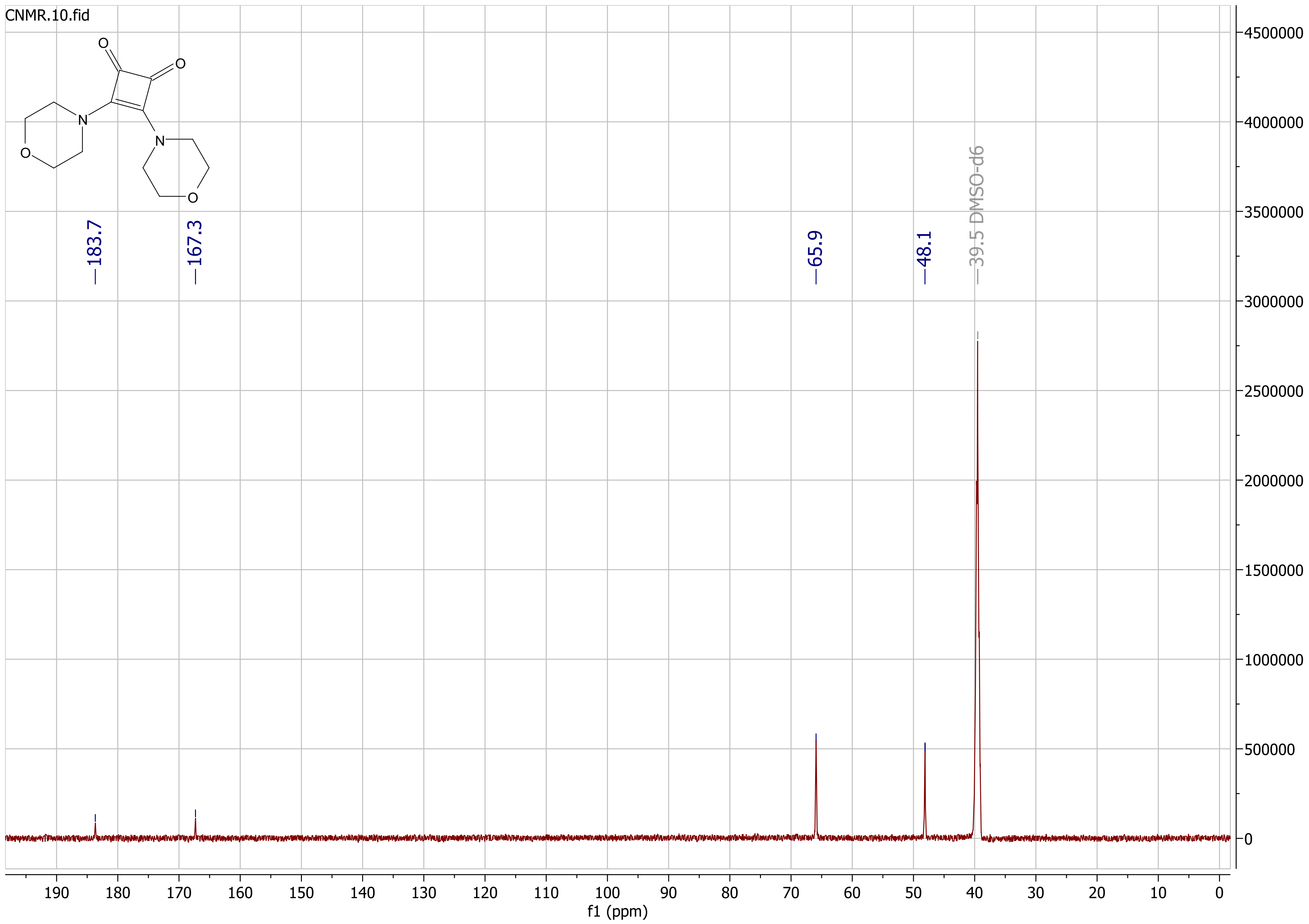
CNMR.10.fid
O
O
N
O
N
O
183.7
167.3
65.9
48.1
39.5 DMSO-d6
4500000
4000000
3500000
3000000
2500000
2000000
1500000
1000000
500000
0
190 180 170 160 150 140 130 120 110 100 90 80 70 60 50 40 30 20 10 0
f1 (ppm)

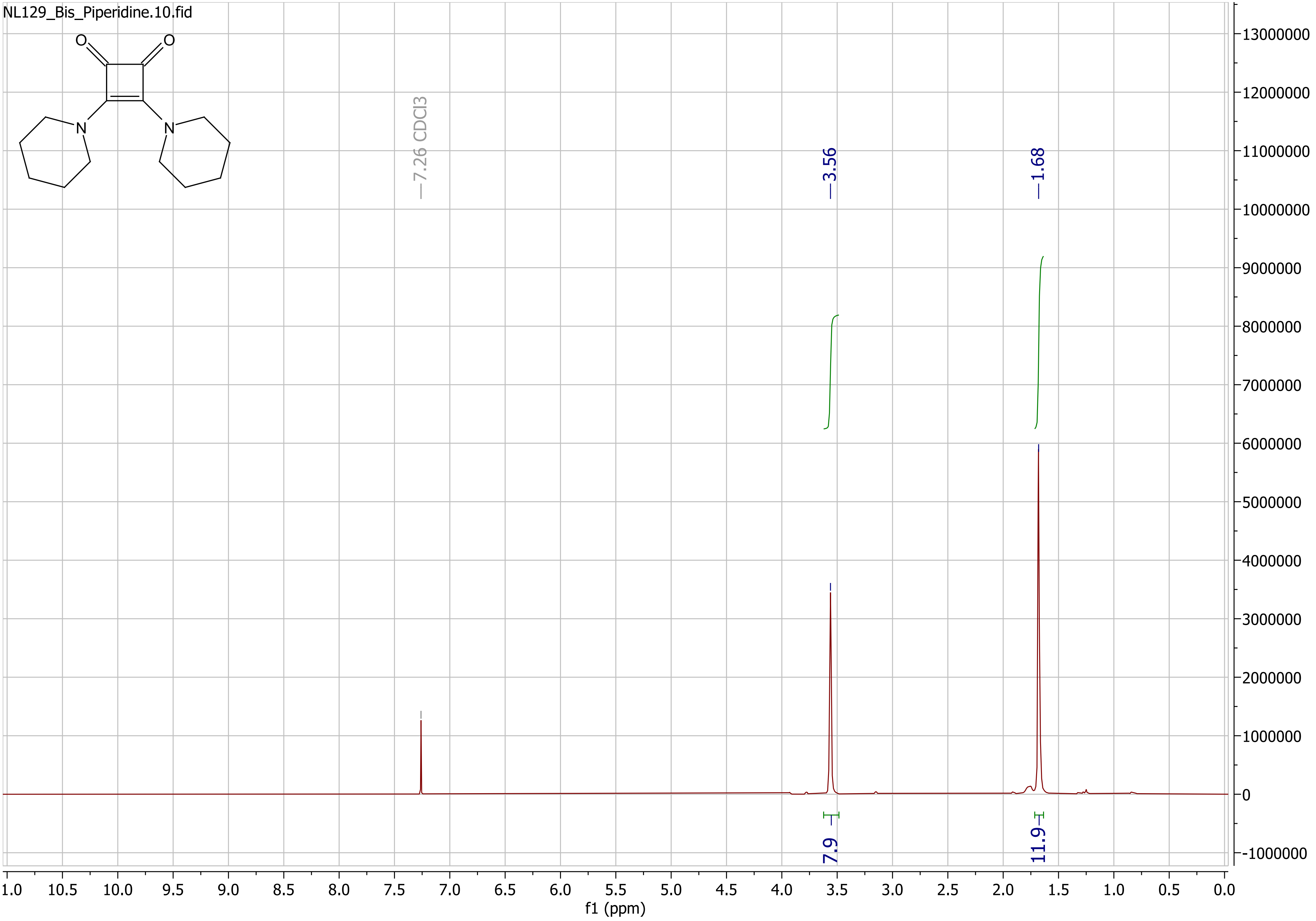
NL129_Bis_Piperidine.10.fid
O
O
N
N
7.26 CDCl3
3.56
1.68
7.9
11.9
13000000
12000000
11000000
10000000
9000000
8000000
7000000
6000000
5000000
4000000
3000000
2000000
1000000
0
-1000000
1.0
10.5
10.0
9.5
9.0
8.5
8.0
7.5
7.0
6.5
6.0
5.5
5.0
4.5
4.0
3.5
3.0
2.5
2.0
1.5
1.0
0.5
0.0
f1 (ppm)

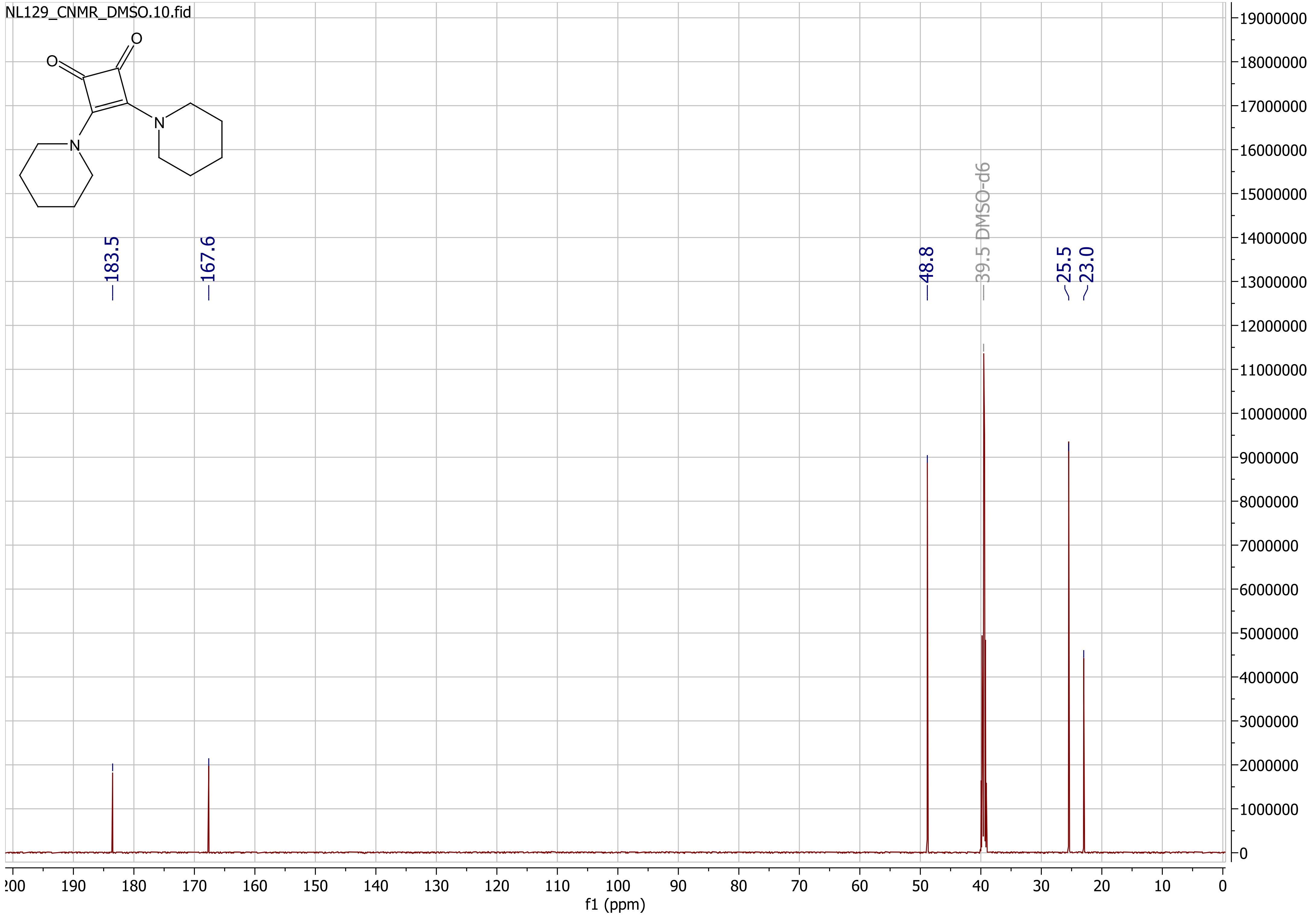

NL129_CNMR_DMSO.10.fid
O
O
N
N
183.5
167.6
48.8
39.5 DMSO-d6
25.5
23.0
19000000
18000000
17000000
16000000
15000000
14000000
13000000
12000000
11000000
10000000
9000000
8000000
7000000
6000000
5000000
4000000
3000000
2000000
1000000
0
200
190
180
170
160
150
140
130
120
110
100
90
80
70
60
50
40
30
20
10
0
f1 (ppm)

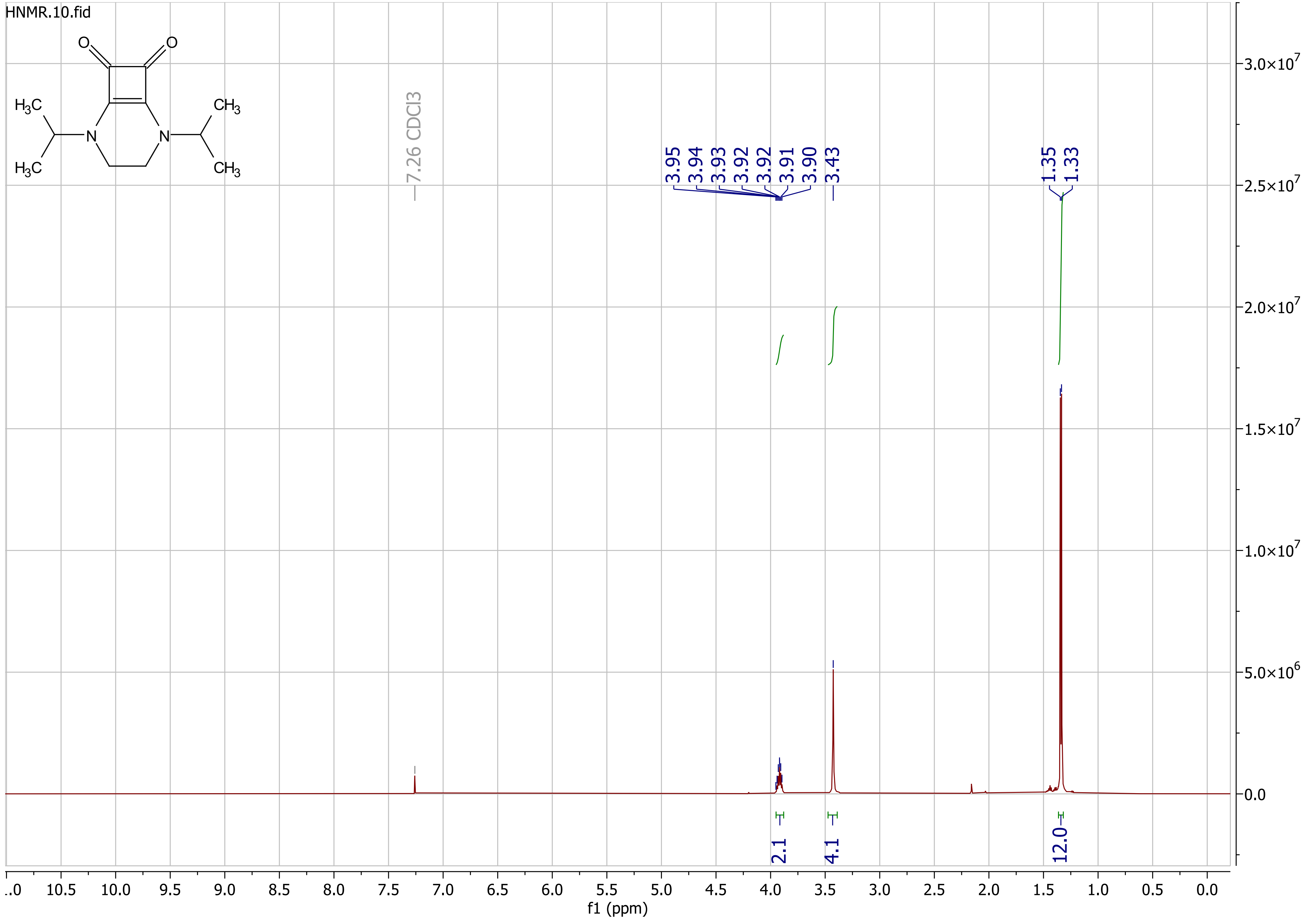
HNMR.10.fid
7.26 CDCl3
3.95
3.94
3.93
3.92
3.92
3.91
3.90
3.43
1.35
1.33
2.1
4.1
12.0
3.0×10^7
2.5×10^7
2.0×10^7
1.5×10^7
1.0×10^7
5.0×10^6
0.0
..0 10.5 10.0 9.5 9.0 8.5 8.0 7.5 7.0 6.5 6.0 5.5 5.0 4.5 4.0 3.5 3.0 2.5 2.0 1.5 1.0 0.5 0.0
f1 (ppm)

NL156_CNMR_CDCl3.10.fid

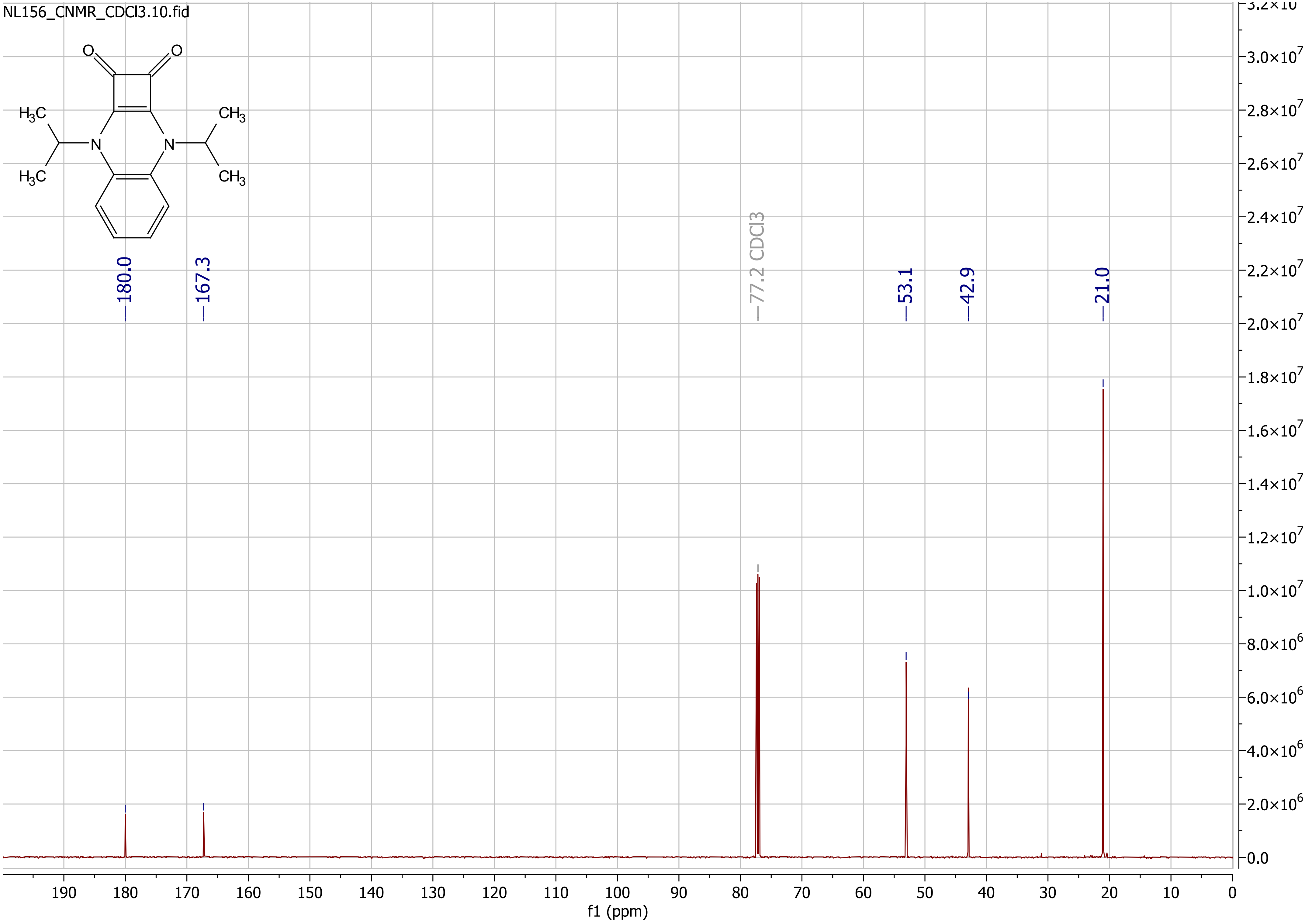

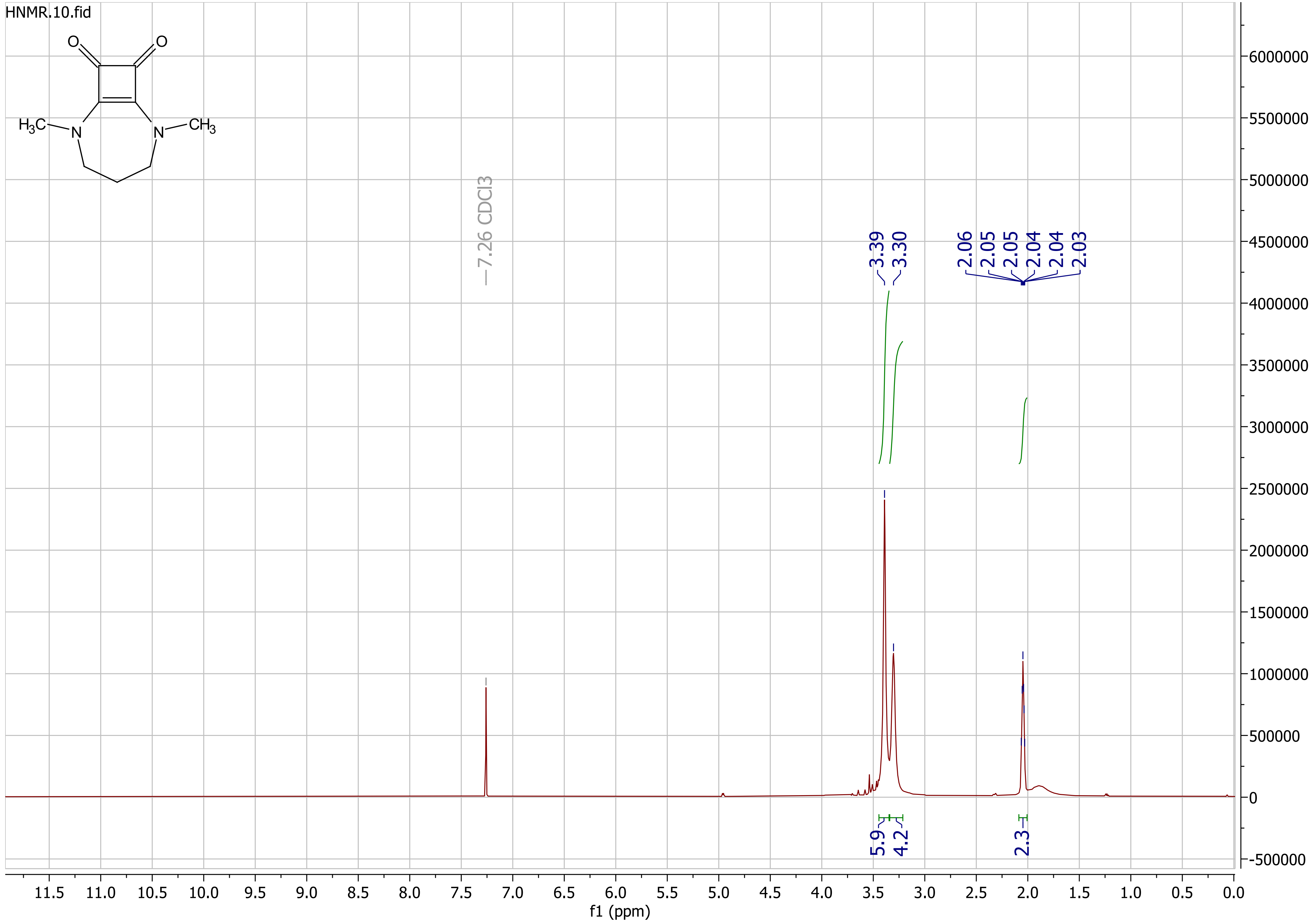

HNMR.10.fid
7.26 CDCl3
3.39
3.30
2.06
2.05
2.05
2.04
2.04
2.03
5.9
4.2
2.3
f1 (ppm)

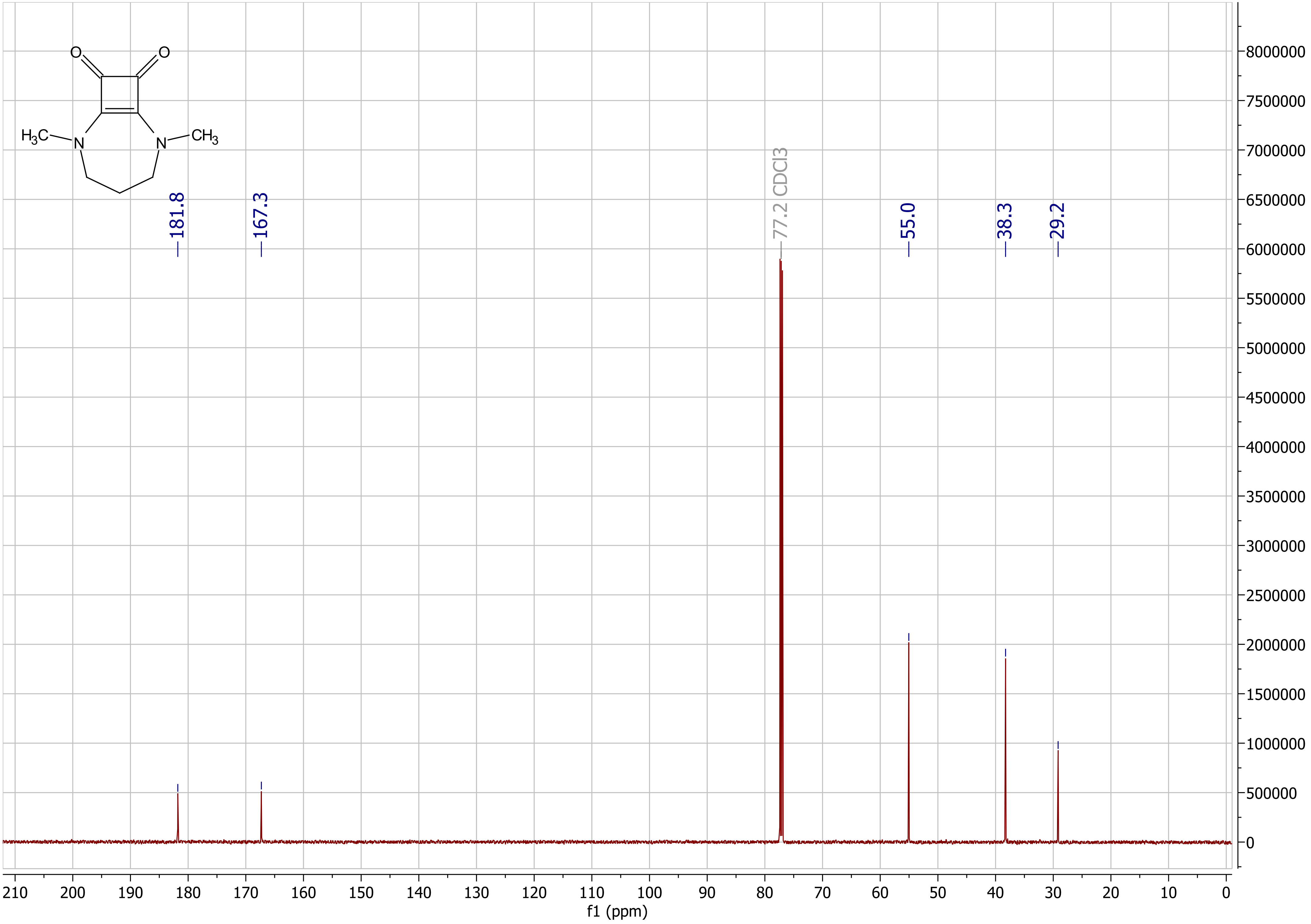

O
O
H3C
N
N
CH3
181.8
167.3
77.2 CDCl3
55.0
38.3
29.2
8000000
7500000
7000000
6500000
6000000
5500000
5000000
4500000
4000000
3500000
3000000
2500000
2000000
1500000
1000000
500000
0
210 200 190 180 170 160 150 140 130 120 110 100 90 80 70 60 50 40 30 20 10 0
f1 (ppm)
